# Tylosin polyketide synthase module 3: stereospecificity, stereoselectivity and steady-state kinetic analysis of β-processing domains *via* diffusible, synthetic substrates†
†Electronic supplementary information (ESI) available. See DOI: 10.1039/c5sc01505g



**DOI:** 10.1039/c5sc01505g

**Published:** 2015-06-12

**Authors:** William D. Fiers, Greg J. Dodge, Yang Li, Janet L. Smith, Robert A. Fecik, Courtney C. Aldrich

**Affiliations:** a Department of Medicinal Chemistry , College of Pharmacy , University of Minnesota , Minneapolis , Minnesota 55455 , USA . Email: aldri015@umn.edu ; Email: fecik001@umn.edu; b Department of Biological Chemistry and Life Sciences Institute , University of Michigan , Ann Arbor , Michigan 48109 , USA

## Abstract

Polyketide synthase (PKS) β-processing domains are responsible for much of the stereochemical complexity of polyketide natural products. Although the importance of β-processing domains has been well noted and significantly explored, key stereochemical details pertaining to cryptic stereochemistry and the impact of remote stereogenic centers have yet to be fully discerned. To uncover the inner workings of ketoreductases (KR) and dehydratases (DH) from the tylosin pathway a didomain composed of TylDH3-KR3 was recombinantly expressed and interrogated with full-length tetraketide substrates to probe the impact of vicinal and distal stereochemistry. *In vitro* product isolation analysis revealed the products of the cryptic KR as d-alcohols and of the DH as *trans*-olefins. Steady-state kinetic analysis of the dehydration reaction demonstrated a strict stereochemical tolerance at the β-position as d-configured substrates were processed more than 100 times more efficiently than l-alcohols. Unexpectedly, the *k*_cat_/*K*_M_ values were diminished 14- to 45-fold upon inversion of remote ε- and ζ-stereocenters. This stereochemical discrimination is predicted to be driven by a combination of allylic A^1,3^ strain that likely disfavors binding of the ε-epimer and a loss of electrostatic interactions with the ζ-epimer. Our results strongly suggest that dehydratases may play a role in refining the stereochemical outcomes of preceding modules through their substrate stereospecificity, honing the configurational purity of the final PKS product.

## Abstract 2

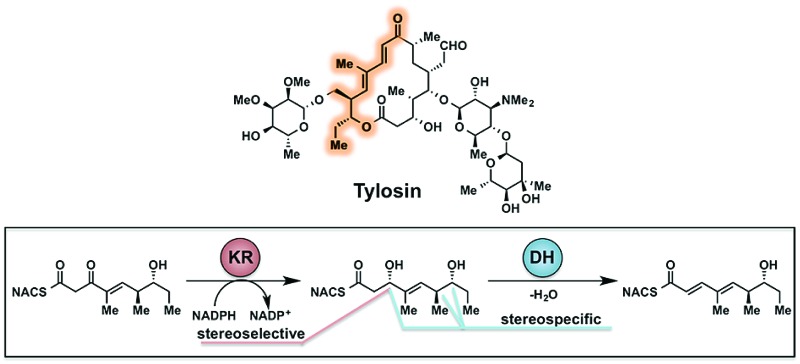
Natural and modified substrates coupled with LC-MS/MS analysis of products revealed the stereospecificity and stereoselectivity of a polyketide didomain.

## Introduction

Polyketides, polyoxygenated secondary metabolites isolated from fungal, plant and bacterial producing organisms, represent an incredibly diverse natural product family with manifold bioactivites.^1–3^ Constituents of this natural product class are thought to serve as defensive and cell–cell signaling agents arising from billions of years of evolution. The complex and varied structural characteristics of polyketides are derived from their highly tunable, assembly line-like biosynthesis. Modular type I polyketide synthases (PKSs) are characterized by multi-functional proteins equipped with numerous catalytic domains, each responsible for a unique enzymatic reaction in the biosynthetic pathway.^4^ A minimal module consists of acyl carrier protein (ACP), ketosynthase (KS) and acyl transferase (AT) domains. Additionally, polyketide modules often carry out varying degrees of β-carbon processing by successive action of ketoreductase (KR), dehydratase (DH), and enoyl reductase (ER) domains. These catalytic domains transform the β-keto moiety produced from the ACP, KS, and AT domains into hydroxyl, olefin, and saturated alkane products depending on their presence in the biosynthetic pathway. The β-processing domains create stereogenic centers and set the olefin geometry present in the final natural product with extremely stringent fidelity.^5–8^

The stereochemical and/or geometric outcome of β-processing domains is often concealed or obscured through subsequent, downstream catalytic events. These instances of hidden domain action can fall into two broad categories: cryptic ketoreductase stereochemistry and cryptic dehydratase geometry. Cryptic KR reductions arise from presence of a subsequent DH domain, catalytically eliminating water and, in so doing, removing both α- and β-stereogenic centers. *trans*-Olefin configuration arises from the elimination of d-alcohols while *cis*-olefins emanate from enzyme-mediated isomerization events.^9,10^ Despite recent, compelling evidence suggesting that, in phoslactomycin biosynthesis, PKS DH domain-catalyzed generation of *Z*-olefins occurs from l-alcohols, *in vitro* validation has yet to be obtained.^11,12^ Olefin geometry may be rendered cryptic through successive reduction by an ER domain, potentially yielding a new α-stereogenic center in the process. Cryptic reductions have received a significant amount of attention over the last decade resulting in several novel approaches to their study.^13,14^ Bioinformatic analysis has shown promise in predicting stereogenic centers based on amino acid sequence of the KR domain in question.^9,15,16^

**Fig. 1:**
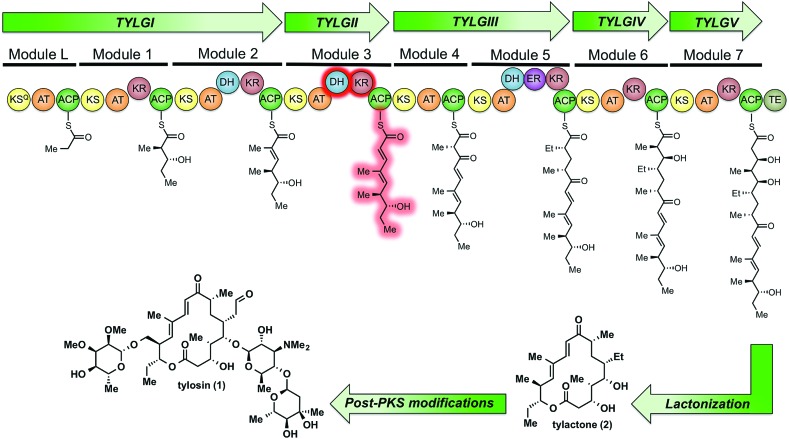
The modular PKS of tylactone (**1**). The module 3 β-processing domains and their postulated product are highlighted in red.

Tylosin (**1**), a 16-membered macrolactone product of *Streptomyces fradiae*, was chosen as a model system for our initial cryptic domain studies. The tylosin polyketide synthase includes one loading module and seven extension modules terminating in a thioesterase (TE) domain affording the aglycone tylactone (**2**) (Fig. 1).^17^ By virtue of their DH domains, modules 2, 3, and 5 have cryptic KR stereochemistry. Additionally, module 5 housing an ER domain constitutes a complete reductive sequence further obscuring the geometry of the precursor olefin. Prior methods to study cryptic KRs and DHs using synthetic substrates have generally been limited to diketides. Truncated substrates, while synthetically more accessible, are often poorly tolerated resulting in low conversion and exhibit loose stereochemical discrimination.^18,8^ As a result, the inferred substrate specificity obtained using truncated substrates remains dubious given their significant deviation from the native chain intermediates. Cane *et al.* overcame this limitation through the *in situ* chemoenzymatic synthesis of a triketide from a diketide substrate using a KS-AT didomain and excised ACP domain from the DEBS pathway.^19^ However, we anticipated this strategy would be difficult to implement for tetraketides as this would require the use of two complete modules in tandem.

**Fig. 2:**
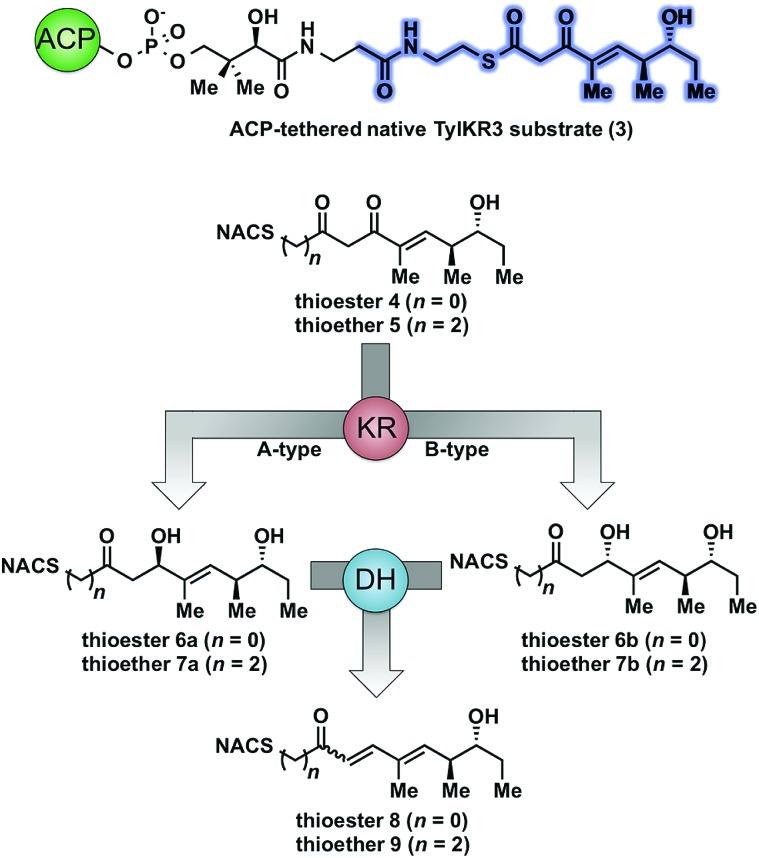
Native and synthetic TylKR3 substrates with their possible β-processing products. The truncated region of the native substrate serving as the basis of substrates **4** and **5** is highlighted in blue.

As part of ongoing studies in our laboratories, we are interested in the development and use of small molecule tools for exploring innate reactivity within polyketide synthase modules. In the present study we sought to probe the enzyme catalyzed turnover of full-length tetraketide substrates **4**, **6a** and **6b** by TylKR3 and TylDH3 *via* LC-MS/MS detection (Fig. 2). One virtue of our chosen tetraketides is that they uniformly lack a δ-hydroxyl moiety which has been shown to spontaneously lactonize onto the thioester.^18–20^ Our previous strategy utilized stable full-length polyketide intermediate mimics that are resistant to spontaneous intramolecular lactonization through replacement of the labile thioester linkage with a stable thioether moiety.^14,21^ In light of these results, we sought to validate the use of thioether analogs **5**, **7a** and **7b** for direct comparison with the aforementioned thioester substrates. This would constitute the first steady-state analysis of a polyketide dehydratase domain using native substrates, uncover the cryptic stereochemistry of TylKR3, and would offer a unique, more native context to discover innate substrate biases. The use of a natural full-length tetraketide chain intermediates and epimers at each stereogenic center would also allow us to evaluate the impact of vicinal and distal stereochemistry on KR and DH substrate processing.

## Results and discussion

### Thioether substrate syntheses

**Scheme 1:**
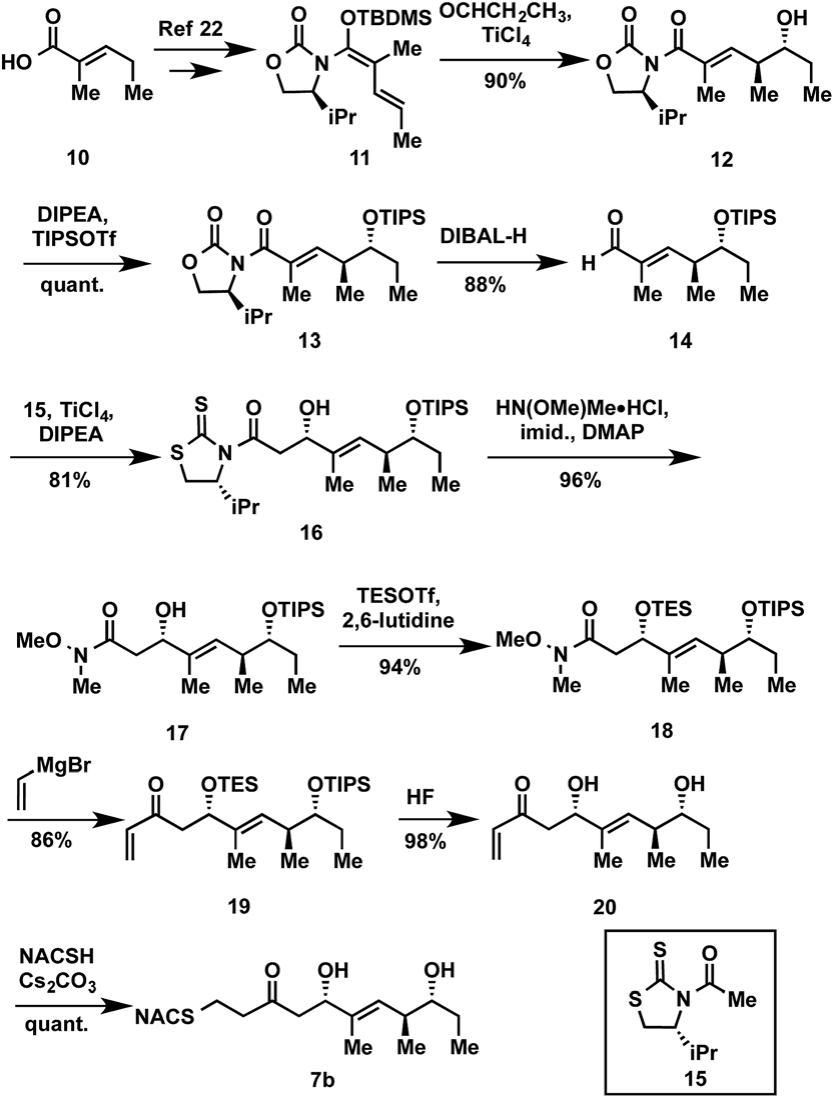
Exemplary synthesis of thioether **7b**.

The synthesis of tetraketide substrate mimic **7b** for TylDH3 began with known vinylketene silyl *N*,*O*-acetal **11**, obtained in two steps from commercially available *trans*-2-methyl-2-pentenoic acid **10** (Scheme 1). The vinylogous Mukaiyama aldol reaction of **11** with propionaldehyde set the two distal stereogenic centers with excellent yield and diastereoselectivity (92%, >98 : 2 dr), illustrating the power of Kobayashi's methodology for synthesis of this triketide building block.^22^ The relative and absolute stereochemistry was confirmed by comparison of its NMR spectral data and optical rotation value to the reported enantiomer.^23^ The vinylogous aldol adduct **12** was subsequently protected as the triisopropylsilyl (TIPS) ether **13** in quantitative yield and reductive removal of the oxazolidinone auxiliary with diisobutylaluminum hydride (DIBAL-H) provided aldehyde **14**.

With the enal **14** in hand, we were poised to set the unknown stereochemistry of the TylKR3 reduction product. Utilization of Nagao's *N*-acetylthiazolidinethione **15** under titanium-catalyzed conditions developed by Vilarrassa, Urpí and coworkers furnished the d-alcohol as the only detectable diastereomer in 81% yield.^24,25^ The thiazolidinethione chiral auxiliary of **16** was displaced with methyl(methoxy)amine to afford the corresponding Weinreb amide **17**.^26^ The newly formed β-hydroxyl group was protected as triethylsilyl (TES) ether **18**. Due to susceptibility to α,β-elimination, the strength of base was crucial, as tertiary amines (TEA, DIPEA) yielded exclusively the conjugated dienamide, whereas 2,6-lutidine afforded the desired TES ether. The precise order of this two-step sequence (**16** → **18**) was critical as reversal led to a sterically encumbered, hydroxyl-protected thiazolidine resistant to displacement. Grignard addition of vinylmagnesium bromide to Weinreb amide **18** provided **19** that was globally deprotected with HF to afford **20**. Regioselective Michael addition of *N*-acetylcysteamine (NAC) to the terminal enone of **20** produced TylDH3 substrate mimic **7b** containing a two-carbon spacer. The l-alcohol diastereomer **7a** was prepared in an analogous fashion from **14** employing the antipode of **15** (ESI, Schemes S1 and S2†).

**Scheme 2:**
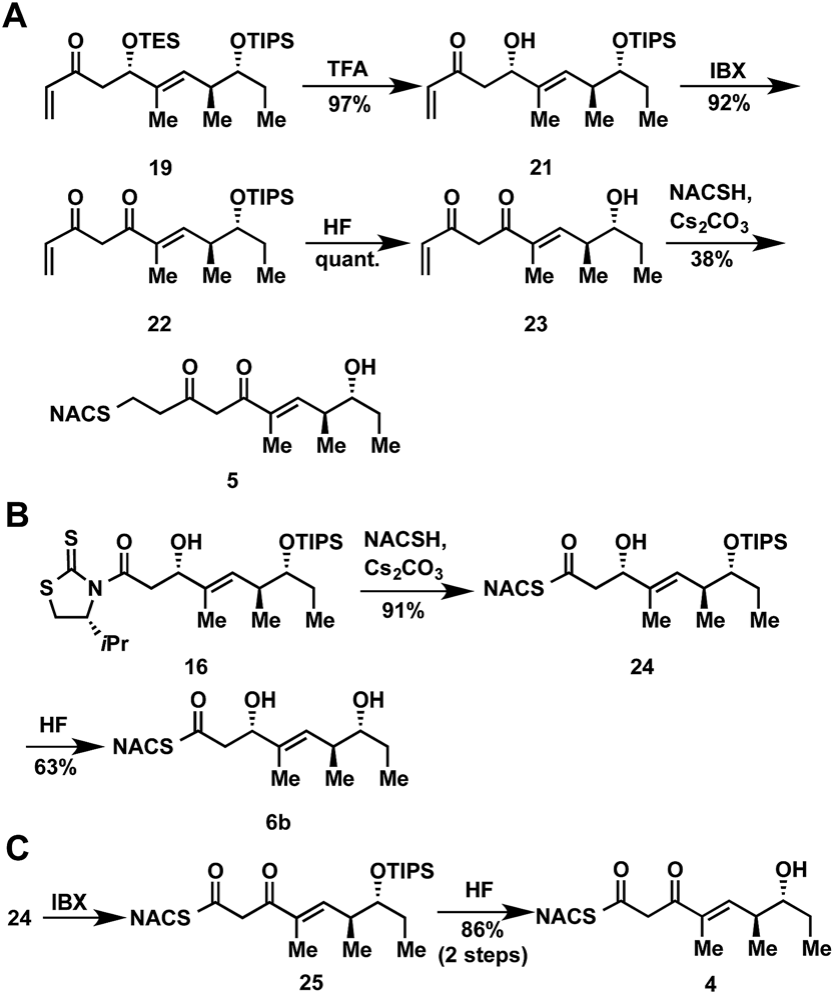
Synthetic route to ketoreductase and thioester substrates **4**, **5**, and **6b**.

At the onset of the project we had planned to prepare the TylKR3 substrate mimic **5** from the corresponding TylDH3 substrate **7b** through regioselective oxidation of the allylic alcohol over the distal secondary alcohol. Unfortunately **7b** and its precursor **20** proved recalcitrant to a variety of oxidants (MnO_2_, BaMnO_4_, Pd(OAc)_2_/O_2_, *etc.*), returning starting material or dehydration products under more forcing conditions.^27–29^ In light of these results, we decided to chemoselectively remove the TES protecting group in **19** to provide **21** (Scheme 2A). A variety of common oxidants were then screened to affect the transformation of alcohol **21** to the desired β-diketone **22** including the Dess–Martin periodinane, TPAP/NMO, and SO_3_•pyr. Surprisingly, most common reagents led to quick decomposition of the starting material or unwanted hetero-Michael additions to afford a tetrahydropyranone. A recently described β-hydroxyketone oxidation using iodoxybenzoic acid (IBX) as the oxidant was employed as a mild, neutral method.^30^ This procedure afforded β-diketone **22** in near quantitative yields after simple filtration of the sparingly soluble oxidant from the products. Facile TIPS deprotection with aqueous HF provided **23**, which was reacted with NAC to afford TylKR3 substrate **5**.

### Thioester product and substrate syntheses

The NAC thioester TylKR3 and TylDH3 substrates **4** and **6b** were synthesized in a straightforward approach from intermediates **16** and **24**, respectively, prepared in Scheme 1. The thiazolidinethione in **16** was directly displaced with NAC yielding the β-hydroxythioester **24** (Scheme 2B). TIPS deprotection with aqueous HF furnished the TylDH3 NAC thioester substrate mimic **6b**. As anticipated, this compound displayed reasonable stability at room temperature and was stable for several months at 4 °C. The C-2 epimeric compound **6a** could be synthesized in a similar manner (ESI, Scheme S1†). Oxidation of β-hydroxythioester **24** would provide the required β-ketothioester. Several reaction conditions were studied to effect this transformation and it was found, once again, that IBX afforded near quantitative yield of **25** (Scheme 2C). TIPS deprotection promoted by aqueous HF yielded TylKR3 NAC thioester substrate mimic **4**.

### Expression of tylosin module 3 β-processing domains

With TylKR3 substrate mimics **4** and **5** in hand, we sought to purify the KR and DH domains. By sequence alignment to structurally characterized domains,^16,31–33^ the sequence boundaries of the KR and DH were determined. The TylKR3 was recalcitrant to purification, so we constructed a plasmid encoding the TylDH3-KR3 didomain including a portion of linker between the KR and ACP domains (residues 957-1682 of tylosin PKS module 3). The didomain was stable upon purification, and used in further analysis. The molecular weight of the recombinant proteins determined by SDS-PAGE was 76 kDa and found to be 76 265 Da by mass spectrometry, both consistent with the calculated value (76 511 Da).

### Enzymatic analysis of TylDH3-KR3

**Fig. 3:**
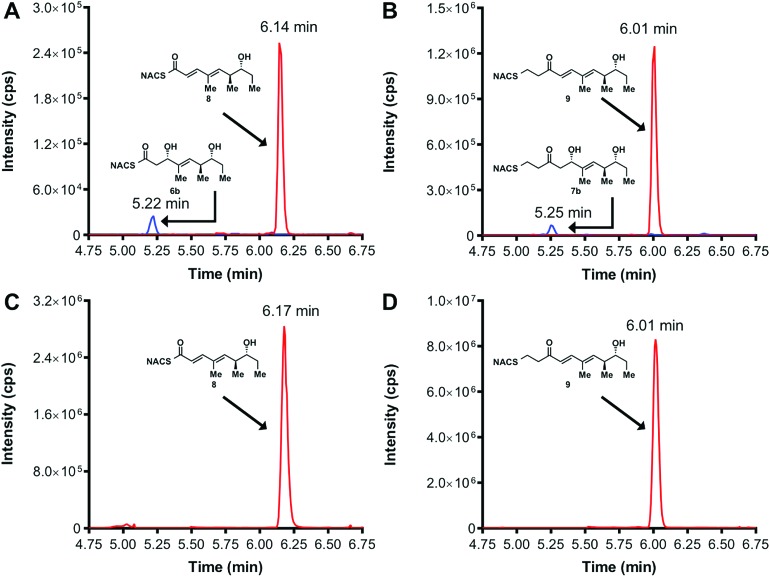
LC-MS/MS traces of *in vitro* ketoreduction and dehydration reactions. Overnight incubation conducted with KR substrates **4** (panel A) and **5** (panel B) and TylDH3-KR3 in the presence of NADPH. The identity of the β-hydroxy products (shown in blue) was confirmed by co-injection with authentic standards. Incubation with synthetic **7** (panel C) and **9** (panel D) resulted in sole formation of dehydration products **10** and **11** (trace shown in red), respectively. Panels A and C blue trace represents MRM (*m*/*z* 340 → 184) and red trace represents MRM (*m*/*z* 300 → 181). Panels B and D blue trace represents MRM (*m*/*z* 368 → 212) red trace represents MRM (*m*/*z* 328 → 151).

We initially attempted to characterize the activity of the TylKR3 domain with substrates **4** and **5** in the presence of NADPH using LC-MS/MS analysis with an internal standard for rigorous quantitation and synthetic standards for product identification. Overnight incubation of the TylDH3-KR3 didomain with **4** and **5** afforded the d-configured reduction products **6b** and **7b** in relatively minor amounts, consistent with the B-type KR domain, along with the dehydration products **8** and **9** as the major species. NAC thioester **4** provided **6b** : **8** in ratio of 1 : 22 while NAC thioether **5** furnished **7b** : **9** in a ratio of 1 : 102 (Fig. 3A and B). However, the KR acted very slowly, as the total conversion in each case was less than 2% of input substrate. The combination of slow KR conversion and low KR : DH product ratio suggests that the ketoreductase product can shuttle to the dehydratase in absence of ACP tethering. It further suggests that the chemically reversible dehydration reaction is unidirectional in the TylDH3 since an unexpectedly high amount of dehydration product was formed from a freely diffusible reduction product. Unfortunately, we were unable to kinetically characterize TylKR3 due to the slow substrate turnover.

We next examined the ability of TylDH3-KR3 to process DH substrates **6a**, **6b**, **7a**, and **7b**. Each substrate (1 mM) was individually incubated overnight with 10 μM TylDH3-KR3 and the reactions were analyzed by LC-MS/MS as before. The d-alcohols **6b** and **7b** led to nearly quantitative formation of *trans*-olefin products **8** and **9**, respectively (Fig. 3C and D) whereas l-alcohols **6a** and **7a** were not turned over by the enzyme. This further corroborates the bioinformatic prediction that the preceding B-type TylKR3 should produce d-alcohols and is consistent with empirical observations that d-alcohols yield *trans*-olefins.^7,34^ Based on the enhanced activity of TylDH3 relative to TylKR3 we performed a large-scale incubation of **6b** and **7b** and isolated **8** and **9** in 67 and 98% yield, respectively after flash chromatography. The product identities were unequivocally confirmed by NMR spectroscopy and exhibited nearly identical diagnostic ^13^C chemical shifts of ∼147 and ∼133 ppm and a ^3^ *J*_HH_ coupling constant of ∼16 Hz.

The enhanced activity of TylDH3 domain enabled characterization by steady-state kinetic analysis. The velocity remained linear up to 10 minutes reaction time and was also linear with respect to TylDH3-KR3 concentration from 0.25 to 1 μM. The initial rates, *v*_0_ at a given [*S*] were thus determined by single-time point stopped-time incubations at 8 minutes with 0.5 μM TylDH3-KR3. Due to the limited solubility of substrates **6b** and **7b** we were unable to reach saturation, consequently the plots of initial velocity *versus* [*S*] were fit by linear regression analysis to determine the specificity constants (*k*_cat_/*K*_M_). Thioester **6b** and thioether **7b** displayed specificity constants of 980 ± 30 and 410 ± 20 min^–1^ M^–1^, respectively (Table 1 and Fig. S2†). The modest 2.5-fold difference in *k*_cat_/*K*_M_ indicates thioethers are well tolerated, validating their use as stabilized forms of substrates otherwise prone to nonproductive, intramolecular cyclization.

**Table 1:** Steady-state kinetic analysis of TylDH3 substrates

Cmpd#	DH substrate	*k*_cat_/*K*_M_, min^–1^ M^–1^
**6a**	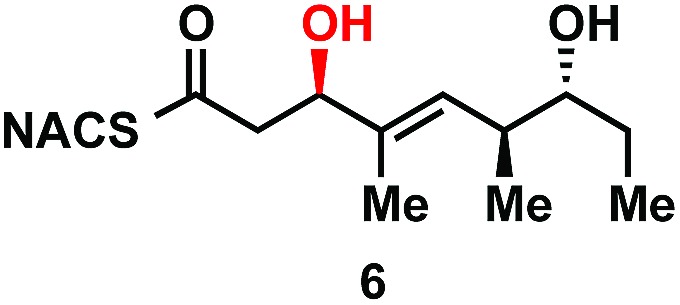	<10^*a*^
**6b**	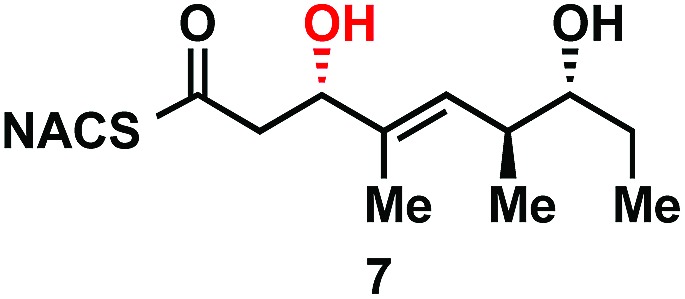	980 ± 30
**7a**	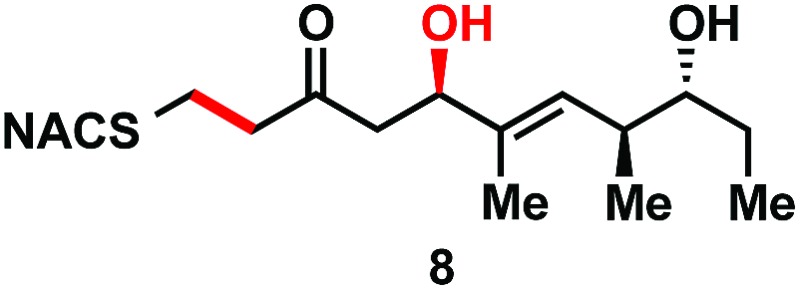	<10^*a*^
**7b**	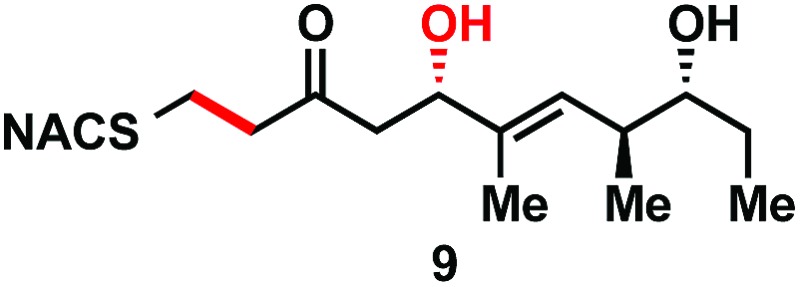	410 ± 20
**6c**	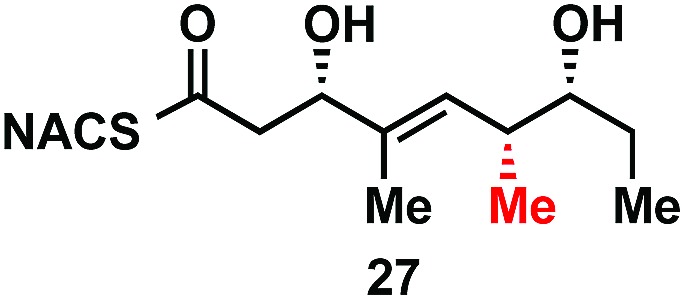	22 ± 2
**6d**	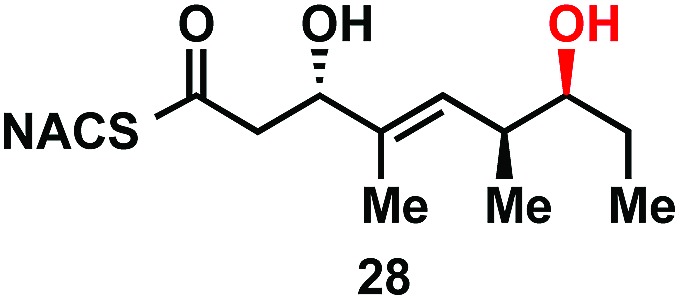	72 ± 6
**(±)-26**	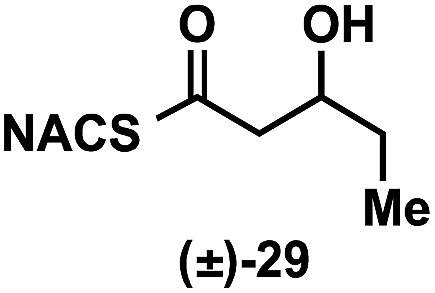	<10^*a*^

^*a*^Below the limit of detection (LOD) of products in LC-MS/MS.

To explore the impact of remote stereocenters on processing by TylDH3 we synthesized full-length tetraketide NAC thioesters **6c** and **6d**, epimeric at the ε- and ζ-stereocenters, respectively (ESI, Schemes S3 and S5†). The specificity constant for **6c** was 22 ± 2 min^–1^ M^–1^, which is 45-fold less than **6b** (Table 1). Although **6c** only differs from **6b** *via* inversion of the ε-methyl group, we expect the trisubstituted olefin may enhance the 1,3-allylic (A^1,3^) strain and more severely impact the side chain conformation, potentially contributing to the drastic attenuation in *k*_cat_/*K*_M_. Boddy and co-workers also invoked A^1,3^ strain to rationalize substrate tolerance in their work on PKS thioesterases.^35^ We next evaluated the ζ-epimer **6d**, whose specificity constant was 72 ± 6 min^–1^ M^–1^, approximately 14-fold less than **6b**. Since inversion of the ζ-stereocenter is not expected to significantly alter the substrate conformation, we speculate that the ζ-hydroxyl group may be important for substrate recognition that is otherwise dominated by hydrophobic interactions of this nonpolar substrate. To complete our substrate specificity studies, we also evaluated diketide **(±)-26**, but it was not processed, highlighting the significance of using full-length substrates.

## Conclusion

The use of full-length, diffusible tetraketide probes allowed for systematic analysis of the module 3 processing domains of tylosin: TylKR3 and TylDH3. The TylKR3 domain was weakly active and produced d-alcohols stereoselectively, further confirming the accuracy of existing bioinformatic approaches.^9,15^ In contrast, the TylDH3 domain proved robust in its production of *trans*-olefins allowing for the chemoenzymatic synthesis of dehydration products. Dehydratase substrate specificity in relation to each stereocenter was independently determined through steady-state kinetic analysis *via* LC-MS/MS detection revealing unpredicted biases for the native substrate. TylDH3 did not tolerate β-stereochemistry inversion, and epimerization of distal stereocenters attenuated the activity by 14–45 fold when compared to the native substrate. This finding was rationalized through recognition of allylic A^1,3^ strain within the molecule and the potential electrostatic and/or hydrogen bonding interactions of the distal hydroxyl moiety. The distant elements of the substrate were necessary for activity as truncated substrate **(±)-26** was not dehydrated by TylDH3. Additionally, thioethers proved to be stable thioester surrogates.

This work highlights the *in vitro* use of didomains in the study of cryptic processes as potential solutions for insoluble and/or unreactive domains. The ACP domain has been largely assumed to control the flow of intermediates throughout the reductive progression of domains towards the ultimate module product.^36–38^ Unexpectedly, we discovered that tylosin module 3 funnels diffusible substrates from ketoreductase to dehydratase independent of ACP tethering. The exact mechanism of this observed phenomenon remains to be determined and could involve the proximity of the KR product exit and DH substrate entrance, thereby increasing the local concentration of DH substrate. Alternatively, a conformational change prior to or upon substrate release from the ketoreductase may draw the two catalytic sites together, leading to the observed shuttling process. Our results are consistent with structures of the pikromycin module 5 in which the ACP localization was determined by the tethered acyl group.^39,40^

The finding that the distal stereochemical fidelity of preceding modules can be closely regulated by dehydratase activity *via* a stringently stereospecific process may have far-reaching implications in the fields of natural product isolation and synthetic biology. Specifically, this research directly supports the hypothesis that tightly controlled relative and absolute polyketide stereochemistry may not necessitate the action of exquisitely stereoselective domains but, instead, be the consequence of iterative, stereospecific checkpoints or gatekeeper domains. Stalled chain intermediates have been shown previously to be hydrolyzed by downstream TE domains, freeing a non-productive, immature polyketide acid and phosphopantetheine-ACP arm for productive product formation.^41–43^ Interestingly, our work suggests that the dehydratase domain, which eliminates stereochemical information, can additionally enrich the final product optical purity. As the dehydratase-catalyzed syn-elimination of water is the only β-processing domain to require a specific, two-centered tetrahedral substrate conformation, it may be naturally sensitive to the local stereochemical features of the substrate.

## Acknowledgements

The authors thank B. Witthuhn (Center for Mass Spectrometry and Proteomics, University of Minnesota) for assistance with LC-MS/MS. Financial support from NIH GM081544 and DK042303 (both to J.L.S.), the Department of Medicinal Chemistry of the University of Minnesota (to Y.L. and W.D.F.) and the NIH Chemical Biology Training Grant (GM008700 for W.D.F.) is gratefully acknowledged.
