# Supplementary material for: Tylosin polyketide synthase module 3: stereospecificity, stereoselectivity and steady-state kinetic analysis of β-processing domains via diffusible, synthetic substrates
Source: Chem Sci. 2015 Jun 12;6(8):5027–33. doi: 10.1039/c5sc01505g (PMC4540058; doi:10.1039/c5sc01505g)
Supplement: Supplementary file 1 [file SC-006-C5SC01505G-s001.pdf]

Electronic Supplementary Material (ESI) for Chemical Science.
This journal is © The Royal Society of Chemistry 2015

SUPPORTING INFORMATION

For

Tylosin Polyketide Synthase Module 3: Stereospecificity, Stereoselectivity and Steady-State Kinetic Analysis of β-Processing Domains via Diffusible, Synthetic Substrates

William D. Fiers,[1] Gregory J. Dodge,[2] Yang Li,[1] Janet L. Smith,[2] Robert A. Fecik,[1]* and Courtney C. Aldrich[1]*

[1]Department of Medicinal Chemistry, College of Pharmacy, University of Minnesota, Minneapolis, Minnesota 55455, United States

[2]Department of Biological Chemistry and Life Sciences Institute, University of Michigan, Ann Arbor, Michigan 48109, United States

Table of Contents

General Chemistry Procedures--S3

Scheme S1--- S12

Scheme S2--- S14

Scheme S3--- S17

Scheme S4--- S21

Scheme S5--- S22

Scheme S6--- S24

General Biology Procedures---S25

Figure S1---S26

Figure S2---S27

Table S1-- S27

Figure S3---S29

Figure S4---S29

References-- S30

^{1}H-NMR and ^{13}C-NMR Spectra---S31

General Chemistry Procedures. All chemical reagents were used as provided unless explicitly indicated otherwise. Tetrahydrofuran (THF) and dichloromethane (CH_2Cl_2) were purified via passage through alumina columns. All reactions were performed under anhydrous argon atmosphere using oven-dried (150 °C) glassware. Reaction mixtures were stirred magnetically using oven-dried magnetic stir bars. Compound purification via flash chromatography utilized silica gel (300–400 mesh) in the indicated solvent system. TLC was performed using 250 μm, F_{254} silica gel plates and visualized UV and *para*-anisaldehyde stain. A Rudolph Autopol III polarimeter at the indicated temperature using the sodium D line (λ = 589 nm) unless otherwise specified and reported as follows: $[\alpha]^{temp}_{D}$= rotation (*c* g/100 mL, solvent). ^1H and ^{13}C NMR spectra were recorded on a Bruker 400 spectrometer at 400 Hz for ^1H NMR and at 100 Hz for ^{13}C NMR. Chemical shifts are reported in ppm based on an internal standard of residual $CHCl_3$ (7.26 ppm in ^1H NMR and 77.16 in ^{13}C NMR). Proton chemical data are reported in the following format: chemical shift (ppm) (multiplicity (s = singlet, d= doublet, t = triplet, q = quartet, quin = quintet, sextet = sextet, sept = septet, m = multiplet, br = broad peak), *J* = coupling constant (Hz), integration). High-resolution mass spectra (HRMS) were obtained on a Bruker BioTOF II ESI-TOF/MS using either PEG or PPG standards as high resolution calibrants.

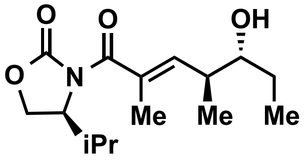

(*S*)-3-((4*S*,5*R*,*E*)-5-hydroxy-2,4-dimethylhept-2-enoyl)-4-isopropyloxazolidin-2-one (12). An oven-dried round bottom flask (250 mL) equipped with magnetic stir bar charged with freshly distilled propionaldehyde[1] (4.93 mL, 68.3 mmol, 2 equiv.) in anhydrous dichloromethane (120 mL) was placed in dry ice-acetone bath (-78 °C) and allowed to equilibrate. To the cooled solution was added titanium(IV) chloride (3.77 mL, 34.2 mmol, 1 equiv.). To the resulting yellow reaction mixture was slowly added the vinylketene silyl *N*,*O*-acetal **11**[2] (11.60 g, 34.2 mmol, 1 equiv.) as a solution in CH_2Cl_2 (45 mL, anhydrous). The reaction quickly adopted a dark red hue. Following completion of addition the reaction mixture was transferred to dry ice-acetonitrile bath (-40 °C) and stirred for 21 hours. The reaction mixture was quenched upon treatment with saturated Rochelle's salt (aq.) followed by saturated sodium bicarbonate (aq.) solutions at -40 °C. The cloudy, white solution was allowed to warm to room temperature and stirred for 1 hour. The resulting biphasic solution was separated and the aqueous layer was extracted with dichloromethane (3 x 75 mL) and ethyl acetate (3 x 75 mL). The combined organic solutions were dried over anhydrous Na_2SO_4, filtered, and concentrated under vacuum. The resulting crude oil was dry-loaded onto silica column and purified via flash chromatography (30% EtOAc/hexanes) yielding the title compound (**12**) (8.76 g, 30.9 mmol, 90%) as a viscous, slightly yellow oil. R_f = 0.20 (20% EtOAc/hexanes); $[\alpha]^{23}_{D}$= 28.3 (*c* 1.00, $CHCl_3$); ^1H NMR ($CDCl_3$, 400 MHz) δ 5.82 (d, *J* = 10.4 Hz, 1H), 4.58 (dt, *J* = 8.8, 4.8 Hz, 1H), 4.34 (t, *J* = 8.8 Hz, 1H), 4.18 (dd, *J* = 7.2, 7.6 Hz, 1H), 3.32–3.25 (m, 1H), 3.08 (br, 1H), 2.63–2.52 (m, 1H), 2.38–2.28 (m, 1H), 1.94 (s, 3H), 1.76–1.64 (m, 1H), 1.44 (app sept, *J* = 7.6 Hz, 1H), 1.01 (t, *J* = 7.2 Hz, 3H), 0.98 (d, *J* = 7.2 Hz, 3H), 0.95–0.89 (m, 6H); ^{13}C NMR ($CDCl_3$, 100 MHz) δ 171.7, 154.7, 142.5, 131.2, 76.7, 63.6, 58.3, 39.9, 28.6, 26.8,

18.0, 16.3, 15.4, 14.1, 10.1; HRMS (ESI-TOF) *m/z*: $[M + Na]^+$ Calcd for $C_{15}H_{24}NO_4Na$ 306.1676; Found 306.1673.

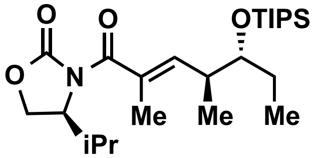

(*S*)-3-((4*S*,5*R*,*E*)-2,4-dimethyl-5-((triisopropylsilyl)oxy)-hept-2-enoyl)-4-isopropyloxazolidin-2-one (13). A solution containing the aldol adduct **12** (889 mg, 3.14 mmol, 1 equiv.) in dichloromethane (24 mL, anhydrous) was cooled via ice-water bath. To the chilled, colorless solution was added diisopropylethylamine (0.655 mL, 3.76 mmol, 1.2 equiv.) followed by triisopropylsilyl trifluoromethylsulfonate (1.01 mL, 3.76 mmol, 1.2 equiv.). The resulting solution was stirred at 0° C (1 h) and transferred to refrigerator (3-5° C, 24 h). The yellow, clear reaction solution was quenched upon addition of saturated sodium bicarbonate (aq.) solution at 0° C and allowed to warm to room temperature (20 min). The biphasic mixture was separated and the aqueous layer was extracted with dichloromethane (4 x 20 mL). The combined organic layers were dried over Na_2SO_4, filtered, and concentrated under vacuum. The product residue was purified by flash chromatography (10% EtOAc/hexanes) affording the silyl ether **13** (1.3805 g, 3.14 mmol, quant.) as a transparent, colorless oil. R_f = 0.68 (20% EtOAc/hexanes); $[\alpha]_D^{21}$ = 29.5 (*c* 1.00, $CHCl_3$); ^1H NMR ($CDCl_3$, 400 MHz) δ 6.19 (dd, *J* = 9.6, 1.2 Hz, 1H), 4.50 (ddd, *J* = 9.2, 5.6, 4.4, 1H), 4.29 (t, *J* = 8.8), 4.14 (dd, *J* = 6.0, 9.2 Hz, 1H), 3.73(ddd, *J* = 5.2, 2.4, 7.6, 1H), 2.72 (ddq, 9.6, 6.8, 2.4 Hz, 1H), 2.42-2.30 (m, 1H), 1.92 (d, *J* = 1.2 Hz, 3H), 1.63-1.48 (m, 2H), 1.10-1.04 (m, 24H), 0.90 (t, *J* = 7.2 Hz, 6H), 0.86 (t, *J* = 7.6 Hz, 3H); ^{13}C NMR ($CDCl_3$, 100 MHz) δ 172.4, 153.8, 141.1, 130.6, 77.8, 63.4, 58.4, 37.0, 28.3, 18.5, 18.4, 18.0, 16.7, 15.1, 13.7, 13.2, 10.5; HRMS (ESI-TOF) *m/z*: $[M + Na]^+$ Calcd for $C_{24}H_{45}NO_4Na$ 462.3010; Found 462.3019.

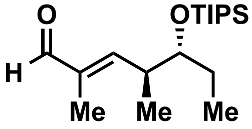

(4*S*,5*R*,*E*)-2,4-dimethyl-5-((triisopropylsilyl)oxy)hept-2-enal (14). A solution of imide **13** (3.185 g, 7.24 mmol, 1 equiv.) in anhydrous dichloromethane (229 mL) was equilibrated in dry ice-acetone bath (-78 °C) under argon atmosphere. To the stirred solution was added diisobutylaluminum hydride (1.49 M in toluene, 9.73 mL, 14.5 mmol, 2 equiv.) in a dropwise fashion over two minutes. The reaction mixture was allowed to stir at -78 °C for 14 minutes then quenched upon successive addition of methanol (15 mL) and saturated, aqueous sodium potassium tartrate (15 mL). The resulting mixture was allowed to warm to room temperature and stirred vigorously overnight (14 h). The biphasic solution was separated and the aqueous layer was extracted with dichloromethane (3 x 100 mL). Combined organic extracts were dried over sodium sulfate, filtered and concentrated under vacuum. The resulting toluene solution was directly loaded onto flash silica column and purified (10% EtOAc/hexanes) furnishing enal **14** (1.983 g, 6.34 mmol, 88%) as a clear, colorless oil. R_f = 0.52 (10% EtOAc/hexanes); $[\alpha]_D^{22}$ = -4.3 (*c* 1.00, $CHCl_3$); ^1H NMR ($CDCl_3$, 400 MHz) δ 9.40 (s, 1H), 6.59 (d, *J* = 10.0 Hz, 1H), 3.78 (app. quin, *J* = 4 Hz, 1H), 2.89 (ddq, *J* = 10.0, 6.8, 3.2 Hz, 1H), 1.76 (s, 3H), 1.65-1.54 (m, 1H), 1.41 (app. sept, *J* = 7.6, 1H), 1.12 (d, *J* = 7.2 Hz, 3H), 1.08 (s, 21H), 0.86 (t, *J* = 7.6 Hz, 3H); ^{13}C NMR ($CDCl_3$, 100 MHz) δ

195.8, 156.8, 139.1, 77.4, 37.7, 28.6, 18.4, 16.9, 13.1, 9.9, 9.6; HRMS (ESI-TOF) *m/z*: $[M + Na]^+$ Calcd for $C_{18}H_{36}O_2SiNa$ 335.2377; Found 335.2372.

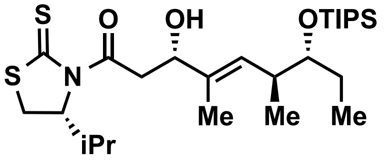

(3*S*,6*S*,7*R*,*E*)-3-hydroxy-1-((*R*)-4-isopropyl-2-thioxo-thiazolidin-3-yl)-4,6-dimethyl-7-((triisopropylsilyl)-oxy)non-4-en-1-one (16). A dichloromethane (anhydrous, 19.56 mL) solution of acetyl thiazolidinone **15** (1.07 g, 5.26 mmol, 1.7 equiv.) was cooled to -40 °C (dry ice-acetonitrile bath) under inert, argon atmosphere. To the chilled, yellow solution was added titanium (IV) chloride (0.611 mL, 5.57 mmol, 1.8 equiv.) resulting in a sudden color change to bright orange. The reaction mixture was stirred for 30 minutes. Slow addition of freshly distilled diisopropylethylamine[1] (0.970 mL, 5.57 mmol, 1.8 equiv.) over three minutes caused a dramatic color change to blood red. The enolate solution was stirred for 2h 12 m while maintaining an external temperature of -40 °C. The reaction was cooled to -78 °C and aldehyde **14** (0.967 g, 3.09 mmol, 1 equiv.) was slowly added over the course of one minute as a solution in dichloromethane (anhydrous, 3.87 mL) and stirred for 5 h. The resulting red-orange mixture was quenched via addition of saturated, aqueous ammonium chloride (10 mL) at -78 °C. The biphasic solution was allowed to warm to room temperature and the layers were separated. The aqueous layer was extracted with dichloromethane (3 x 15 mL) and the combined organic layers were dried over sodium sulfate, filtered and concentrated under vacuum. The resulting clear, golden oil was wet-loaded onto silica column and purified via flash chromatography (20% EtOAc/hexanes) affording the title compound **16** as a bright yellow, viscous oil (1.29 g, 2.50 mmol, 81%). R_f = 0.29 (20% EtOAc/hexanes); $[\alpha]^{22}_{D}$ = -219.0 (*c* 1.00, $CHCl_3$); ^1H NMR (400 MHz, $CDCl_3$) δ 5.51 (d, *J* = 9.6 Hz, 1H), 5.15 (app. t, *J* = 6.8 Hz, 1H), 4.58 (dd, *J* = 8.8, 3.2 Hz, 1H), 3.66 (td, *J* = 6.4, 3.2 Hz, 1H), 3.56–3.44 (m, 3H), 3.03 (d, *J* = 11.6 Hz, 1H), 2.63–2.55 (m, 1H), 2.38 (app. sextet, *J* = 7.2 Hz, 1H), 1.68 (s, 3H), 1.52–1.40 (m, 2H), 1.07 (s, 24H), 0.99 (d, *J* = 2.4, 3H), 0.98 (d, *J* = 2.4, 3H), 0.85 (t, *J* = 7.2 Hz, 3H); ^{13}C NMR ($CDCl_3$, 100 MHz) δ 203.1, 173.0, 135.2, 129.1, 77.7, 73.4, 71.7, 44.3, 36.6, 31.0, 30.8, 27.6, 19.3, 18.5, 17.9, 16.9, 13.2, 12.6, 10.5; HRMS (ESI-TOF) *m/z*: $[M + Na]^+$ Calcd for $C_{26}H_{49}NO_3S_2SiNa$ 538.2815; Found 538.2815.

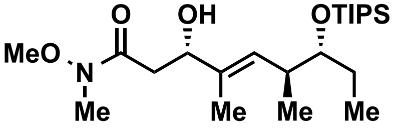

(3*S*,6*S*,7*R*,*E*)-3-hydroxy-*N*-methoxy-*N*,4,6-trimethyl-7-((triisopropylsilyl)oxy)non-4-enamide (17). To a bright yellow dichloromethane (anhydrous, 2 mL) solution of acyl thiazolidinone **16** (203 mg, 0.39 mmol, 1 equiv.) was added imidazole (133 mg, 1.95 mmol, 5 equiv.) followed by methylmethoxyamine hydrochloride (95.0 mg, 0.98 mmol, 2.50 equiv). A catalytic amount of 4-dimethylaminopyridine (one crystal) was added to the final mixture. The reaction was stirred 20 h at room temperature and quenched via addition of saturated aqueous ammonium chloride (2 mL). The biphasic mixture was separated and the aqueous layer was extracted with dichloromethane (4 x 10 mL). The combined organic layers were dried over sodium sulfate, filtered and concentrated under vacuum. The crude residue was purified by flash chromatography (30% EtOAc/hexanes) providing a slightly yellow, clear, viscous oil (155 mg, 0.372 mmol, 96%). R_f = 0.34 (40% EtOAc/hexanes); $[\alpha]^{21}_{D}$ = -35.1 (*c* 1.00, $CHCl_3$); ^1H NMR (400 MHz, $CDCl_3$) δ 5.49 (d, *J* = 9.6 Hz, 1H),

4.45 (dd, J = 8.0, 4.4 Hz, 1H), 3.69 (s, 3H), 3.68–3.63 (m, 1H), 3.20 (s, 3H), 2.68 – 2.56 (m, 3H), 1.69 (s, 1H), 1.47 (dq, J = 7.6, 6.8 Hz, 2H), 1.07 (s, 21H), 0.99 (d, J = 6.8 Hz, 3H), 0.85 (t, J = 7.6 Hz, 3H); ^{13}C NMR ($CDCl_3$, 100 MHz) δ 173.9, 135.4, 128.5, 77.8, 73.4, 61.4, 37.2, 36.6, 32.0, 27.4, 18.4, 16.8, 13.1, 12.6, 10.5; HRMS (ESI-TOF) *m/z*: $[M + Na]^+$ Calcd for $C_{22}H_{45}NO_4SiNa$ 438.3010; Found 438.3009.

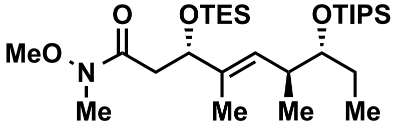

(3*S*,6*S*,7*R*,*E*)-*N*-methoxy-*N*,4,6-trimethyl-3-((triethylsilyl)oxy)-7-((triisopropylsilyl)oxy)non-4-enamide (18). To a reaction flask containing β-hydroxy amide **17** (345 mg, 0.829 mmol, 1.00 equiv.) dissolved in anhydrous dichloromethane (15 mL) in ice-water bath (0 °C) was added 2,6-lutidine (0.387 mL, 3.32 mmol, 4.00 equiv.). The resulting basic solution was supplemented with triethylsilyl trifluoromethanesulfonate (0.375 mL, 1.66 mmol, 2.00 equiv.) and stirred for 1.5 h. The reaction was terminated via the sequential addition of methanol (3 mL) followed by saturated aqueous sodium bicarbonate (5 mL) at 0 °C. The biphasic mixture was warmed to room temperature, separated, and the aqueous fractions were extracted with dichloromethane (4 x 10 mL). The combined organic fractions were dried over anhydrous sodium sulfate, filtered and concentrated under vacuum. The resulting crude oil was wet-loaded onto silica column and purified via flash chromatography (10% EtOAc/hexanes) affording a clear, slightly yellow oil (410.6 mg, 0.776 mmol, 94%). R_f = 0.61 (20% EtOAc/hexanes); $[\alpha]_D^{23}$ = -20.6 (*c* 1.00, $CHCl_3$); ^{1}H NMR (400 MHz) δ 5.45 (d, J = 9.6 Hz, 1H), 4.58 (dd, J = 8.8, 4.4 Hz, 1H), 3.70 (s, 3H), 3.65 (td, J = 6.8, 2.8 Hz, 1H), 3.17 (s, 3H), 2.94–2.80 (m, 1H), 2.56 (dqd, J = 9.6, 6.8, 2.8 Hz, 1H), 2.31 (dd, J = 14.0, 4.4 Hz, 1H), 1.64 (s, 3H), 1.52 – 1.39 (m, 2H), 1.08 (s, 21H), 0.96 (d, J = 6.8 Hz, 3H), 0.91 (t, J = 8.0 Hz, 9H), 0.85 (t, J = 7.6 Hz, 3H), 0.56 (q, J = 8.0 Hz, 6H); ^{13}C NMR ($CDCl_3$, 100 MHz) δ 172.3, 136.8, 128.0, 78.0, 75.5, 61.5, 39.7, 36.6, 32.1, 27.6, 18.4, 16.7, 13.1, 11.7, 10.8, 7.0, 4.9; HRMS (ESI-TOF) *m/z*: $[M + Na]^+$ Calcd for $C_{28}H_{59}NO_4Si_2Na$ 552.3875; Found 552.3882.

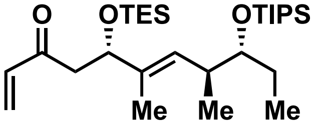

(5*S*,8*S*,9*R*,*E*)-6,8-dimethyl-5-((triethylsilyl)oxy)-9-((triisopropylsilyl)oxy)undeca-1,6-dien-3-one (19). A reaction vessel containing weinreb amide **18** (237 mg, 0.448 mmol, 1 equiv.) in anhydrous tetrahydrofuran (32 mL) was placed in ice-water bath (0 °C) and allowed to equilibrate. To the chilled, clear solution was added solution of vinylmagnesium bromide (1.0 M in THF, 1.44 mL, 1.44 mmol, 3.20 equiv.). The reaction mixture was stirred at 0 °C for 4 h. An additional aliquot of vinylmagnesium bromide (0.450 mL, 0.450 mmol, 1.00 equiv.) was added and the reaction mixture was stirred for 1 h prior to quenching with saturated, aqueous ammonium chloride (10 mL). The biphasic mixture was allowed to warm to room temperature, separated and the aqueous layer was extracted with dichloromethane (3 x 15 mL). The combined organic layers were dried over sodium sulfate, filtered and concentrated under vacuum. The resulting crude oil was wet-loaded onto silica column and purified via flash chromatography (5% EtOAc/hexanes) yielding the desired enone as a clear, colorless oil (192 mg, 0.387 mmol, 86%). R_f = 0.38 (5% EtOAc/hexanes); $[\alpha]_D^{22}$ = -20.7 (*c* 0.978, $CHCl_3$); ^{1}H NMR (400 MHz) δ 6.36 (dd, J = 17.6, 10.8 Hz, 1H), 6.20 (d, J = 17.6 Hz, 1H), 5.82 (d, J = 10.8 Hz, 1H), 5.43 (d, J = 9.2 Hz, 1H), 4.54 (dd, J = 8.4, 4.4 Hz, 1H),

3.64 (td, J = 6.9, 2.6 Hz, 1H), 2.94 (dd, J = 14.0, 8.5 Hz, 1H), 2.63–2.42 (m, 2H), 1.63 (s, 3H), 1.52–1.35 (m, 2H), 1.08 (s, 21H), 0.95 (d, J = 6.8, 3H), 0.89 (t, J = 8.0 Hz, 9H), 0.85, (t, J = 7.4 Hz, 3H), 0.54 (q, J = 8.0 Hz, 6H); ^{13}C NMR ($CDCl_3$, 100 MHz) δ 199.6, 137.6, 136.5, 128.5, 128.1, 77.9, 75.6, 47.0, 36.6, 27.6, 18.4, 13.1, 11.6, 10.8, 7.0, 6.0, 4.9; HRMS (ESI-TOF) *m/z*: $[M + Na]^+$ Calcd for $C_{28}H_{56}O_3Si_2Na$ 519.3660; Found 519.3647.

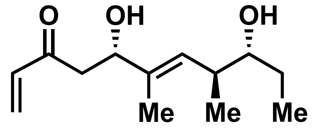

(5*S*,8*S*,9*R*,*E*)-5,9-dihydroxy-6,8-dimethylundeca-1,6-dien-3-one (20). To a polypropylene tube (15 mL, BD Falcon™) containing silylether **19** (29.8 mg, 0.0600 mmol, 1 equiv.) was added acetonitrile (3.0 mL) and magnetic stir bar. The mixture was stirred in ice-water bath (0 °C) until a homogenous solution was obtained. A solution of 48% aqueous hydrofluoric acid and acetonitrile (11:89, 8.36 mL) was slowly added to the reaction vessel. The resulting clear solution was stirred vigorously for 1 h and placed in a refrigerator (4 °C) for 15 h. The reaction was quenched at 0 °C via dropwise addition of a saturated aqueous sodium bicarbonate solution until the pH was 7. The neutralized solution was extracted with ethyl acetate (4 x 15 mL). All organic fractions were combined, dried over sodium sulfate, filtered and concentrated under vacuum. The crude residue was wet-loaded onto silica column and purified via flash column chromatography (5% MeOH/dichloromethane) affording the diol as a clear, colorless oil (13.3 mg, 0.586 mmol, 98%). R_f = 0.30 (5% MeOH/dichloromethane); $[\alpha]_D^{21}$ = -37.2 (*c* 0.26, $CHCl_3$); ^{1}H NMR (400 MHz) δ 6.37 (dd, J = 17.6, 10.3 Hz, 1H), 6.26 (dd, J = 17.7, 1.0 Hz, 1H), 5.90 (dd, J = 11.4, 1.0 Hz, 1H), 5.38 (d, J = 9.9 Hz, 1H), 4.52 (dd, J = 7.3, 4.7 Hz, 1H), 3.32 (ddd, J = 8.4, 6.4, 3.6 Hz, 1H), 2.99 (br, 1H), 2.86–2.80 (m, 2H), 2.53– 2.41(m, 1H), 1.69 (d, J = 1.2 Hz, 3H), 1.62–1.53 (m, 2H), 1.45–1.33 (m, 1H), 0.97 (t, J = 7.4 Hz, 3H), 0.96 (d, J = 6.8 Hz, 3H); ^{13}C NMR ($CDCl_3$, 100 MHz) δ 201.0, 137.6, 136.9, 129.5, 128.4, 77.0, 73.0, 44.6, 38.1, 27.1, 17.2, 13.0, 10.2; HRMS (ESI-TOF) *m/z*: $[M + Na]^+$ Calcd for $C_{13}H_{22}O_3Na$ 249.1461; Found 249.1466.

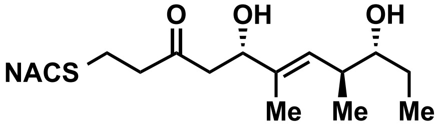

N-(2-(((5S,8S,9R,E)-5,9-dihydroxy-6,8-dimethyl-3-oxoundec-6-en-1-yl)thio)ethyl)acetamide (7b). A reaction flask containing vinyl ketone **20** (3.9 mg) in anhydrous tetrahydrofuran (5 mL) was supplemented with *N*-acetylcysteamine (2.0 μL, 0.019 mmol, 1.1 equiv.). To the resulting clear solution was added a catalytic amount of cesium carbonate and the resulting reaction mixture was stirred for 5 h at ambient temperature under argon atmosphere and quenched upon addition of a saturated, aqueous ammonium chloride solution (5 mL). The biphasic mixture was separated and the aqueous layer was extracted with ethyl acetate (8 x 10 mL). The combined organic fractions were dried over sodium sulfate, filtered and concentrated under vacuum. The crude residue was wet-loaded onto silica flash column with a small copper(II) sulfate-impregnated silica gel plug (2 cm thick) and purified (eluent = 10% MeOH/dichloromethane) furnishing the desired thioether as a colorless, translucent oil (5.9 mg, 0.017 mmol, quant.). R_f = 0.13 (5% MeOH/dichloromethane); $[\alpha]_D^{22}$ = -24.9 (*c* 0.38, $CHCl_3$); ^{1}H NMR (400 MHz) δ 6.11 (s, 1H), 5.36 (d, J = 9.9 Hz, 1H), 4.48 (dd, J = 8.5, 3.4 Hz, 1H), 3.43 (app. q, J = 6.1 Hz, 2H), 3.37–3.24 (m, 1H), 2.86–2.58 (m, 8H), 2.49–2.39 (m, 1H), 1.99 (s, 3H), 1.66 (d, J = 1.0 Hz, 3H), 1.61–1.52

(m, 1H), 1.44–1.32 (m, 1H), 0.99–0.93 (m, 6H); ^{13}C NMR ($CDCl_3$, 100 MHz) δ 209.4, 170.5, 137.5, 128.4, 77.1, 73.1, 48.2, 43.8, 38.7, 38.0, 32.2, 27.2, 25.2, 23.4, 17.2, 13.0, 10.2; HRMS (ESI-TOF) *m/z*: [M + Na]$^+$ Calcd for $C_{17}H_{31}NO_4SNa$ 368.1866; Found 368.1872.

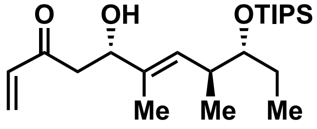

(5*S*,8*S*,9*R*,*E*)-5-hydroxy-6,8-dimethyl-9-((triisopropylsilyl)-oxy)undeca-1,6-dien-3-one (21). To a reaction vessel containing disilylether **19** (39.3 mg, 0.0792 mmol) in a mixture of tetrahydrofuran (1.98 mL) and deionized water (0.296 mL) was added trifluoroacetic acid (15.6 μL, 0.210 mmol, 2.65 equiv.). The acidic solution was stirred for 2.5 h at ambient temperature and carefully neutralized upon addition of a saturated, aqueous sodium bicarbonate solution. The biphasic solution was separated and the aqueous layer repeatedly extracted with ethyl acetate (4 x 7 mL). The combined organic layers were dried over sodium sulfate, filtered and concentrated under vacuum. The crude product was wet-loaded onto silica column and purified via flash column chromatography (10% EtOAc/hexanes) producing a light yellow, clear oil (29.5 mg, 0.0771 mmol, 97%). R_f = 0.25 (10% EtOAc/hexanes); $[\alpha]^{24}_{D}$ = -23.0 (*c* 1.00, $CHCl_3$); ^{1}H NMR (400 MHz) δ 6.37 (dd, *J* = 17.7, 10.4 Hz, 1H), 6.25 (d, *J* = 16.6 Hz, 1H), 5.89 (d, *J* = 10.4 Hz, 1H), 5.48 (d, *J* = 9.6 Hz, 1H), 4.52 (dd, *J* = 9.1, 2.8 Hz, 1H), 3.65 (td, *J* = 6.2, 3.3 Hz, 1H), 2.87 (dd, *J* = 16.8, 9.2 Hz, 1H), 2.75 (dd, *J* = 16.8, 3.1 Hz, 1H), 2.62–2.54 (m, 1H), 1.68 (d, *J* = 1.3 Hz, 3H), 1.54 – 1.40 (m, 2H), 1.07 (s, 21H), 0.97 (d, *J* = 6.9 Hz, 3H), 0.85 (t, *J* = 7.5 Hz, 3H); ^{13}C NMR ($CDCl_3$, 100 MHz) δ 201.1, 136.9, 135.3, 129.2, 128.9, 77.8, 73.3, 44.8, 36.6, 27.6, 18.5, 16.9, 13.2, 12.5, 10.5; HRMS (ESI-TOF) *m/z*: [M + Na]$^+$ Calcd for $C_{22}H_{42}O_3SiNa$ 405.2795, Found 405.2795.

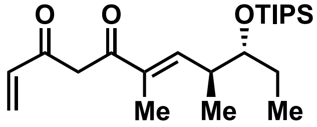

(8*S*,9*R*,*E*)-6,8-dimethyl-9-((triisopropylsilyl)oxy)undeca-1,6-diene-3,5-dione (22). To a small flask containing *β*-hydroxy ketone **21** (80.0 mg, 0.209 mmol) in ethyl acetate (10 mL) was added 2-iodoxybenzoic acid (390 mg, 0.627 mmol, 3.00 equiv., 45% wt/wt). The white, cloudy solution was rapidly stirred and heated at reflux for 2 h. The slightly yellow solution was cooled to ambient temperature and concentrated under vacuum. The resulting concentrated solution was directly loaded onto silica flash column and purified (5% EtOAc/hexanes) yielding a red-orange oil (72.4 mg, 0.190 mmol, 92%). R_f = 0.36 (5% EtOAc/hexanes); $[\alpha]^{21}_{D}$ = -47.2 (*c* 1.00, $CHCl_3$); ^{1}H NMR (400 MHz) δ 15.46 (s, 1H), 6.71 (dd, *J* = 9.9, 1.0 Hz, 1H), 6.25 (dd, *J* = 17.2, 2.0 Hz, 1H), 6.17 (dd, *J* = 9.9, 17.2 Hz, 1H), 5.89 (s, 1H), 5.64 (dd, *J* = 9.9, 2.0 Hz, 1H), 3.75 (ddd, *J* = 7.8, 4.6, 3.6 Hz, 1H), 2.81–2.72 (m, 1H), 1.87 (d, *J* = 1.1 Hz, 3H), 1.63–1.52 (m, 1H), 1.49–1.37 (m, *J* = 14.9, 7.4 Hz, 1H), 1.09–1.07 (m, 24H), 0.86 (t, *J* = 7.5 Hz, 3H); ^{13}C NMR ($CDCl_3$, 100 MHz) δ 193.1, 176.5, 143.0, 134.4, 133.3, 124.5, 96.6, 77.6, 37.8, 28.4, 18.4, 16.9, 13.2, 11.9, 10.0; HRMS (ESI-TOF) *m/z*: [M + Na]$^+$ Calcd for $C_{22}H_{40}O_3SiNa$ 403.2639, Found 403.2646.

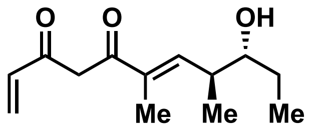

(8*S*,9*R*,*E*)-9-hydroxy-6,8-dimethylundeca-1,6-diene-3,5-dione (23). To a polypropylene tube (15 mL, BD Falcon™) containing silylether **22** (72.4 mg, 0.140 mmol) was added acetonitrile (1.23 mL). The clear solution was equilibrated in

ice-water bath (0 °C) for 10 minutes. A pre-chilled solution of 48% aqueous hydrogen fluoride and acetonitrile (11:89, 9.84 mL) was slowly added to the rapidly stirred solution. The resulting slightly yellow solution was placed in refrigerator (4 °C) for 40 h. The reaction mixture was quenched via slow neutralization with aqueous, saturated sodium bicarbonate solution at 0 °C. The aqueous layer was extracted with ethyl acetate (5 x 20 mL) and the combined organic layers were concentrated under vacuum. The product was placed under high vacuum for 2 h furnishing the desired alcohol **23** as a viscous, yellow oil (42.6 mg, 0.190 mmol, quant.). R_f = 0.20 (20% EtOAc/hexanes); $[\alpha]_D^{22}$ = -51.2 (*c* 1.00, $CHCl_3$); (Compound **23** was found to exist entirely in the enol form (not drawn)) ^{1}H NMR (400 MHz) δ 15.54 (br, 1H), 6.59 (dd, *J* = 10.0, 0.96 Hz, 1H), 6.28 (dd, *J* = 17.2, 2.4 Hz, 1H), 6.21 (dd, *J* = 17.2, 9.5 Hz, 1H) 5.90 (s, 1H), 5.66 (dd, *J* = 9.5, 2.4 Hz, 1H), 3.49 (dt, *J* = 8.8, 4.6 Hz, 1H), 2.73–2.60 (m, 1H), 1.89 (d, *J* = 1.0 Hz, 3H), 1.60–1.49 (m, 1H), 1.43 (dq, *J* = 14.2, 7.5 Hz, 1H), 1.08 (d, *J* = 6.7 Hz, 3H), 0.97 (t, *J* = 7.4 Hz, 3H); ^{13}C NMR ($CDCl_3$, 100 MHz) δ 192.1, 177.7, 141.6, 135.1, 133.3, 125.0, 96.9, 76.9, 39.2, 27.9, 16.8, 12.2, 10.2; HRMS (ESI-TOF) *m/z*: $[M + Na]^+$ Calcd for $C_{13}H_{20}O_3Na$ 247.1305, Found 247.1310.

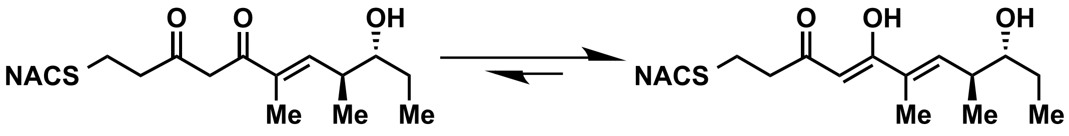

A B

***N*-(2-(((8*S*,9*R*,*E*)-9-hydroxy-6,8-dimethyl-3,5-dioxoundec-6-en-1-yl)thio)ethyl)acetamide (5).** To a round bottom flask containing vinyl ketone (12.3 mg, 0.055 mmol) in anhydrous tetrahydrofuran (10 mL) was added *N*-acetylcysteamine (6.4 μL, 0.060 mmol, 1.1 equiv.) as a solution in anhydrous THF (64 μL). The reaction mixture was supplemented with a catalytic amount of cesium carbonate and stirred at ambient temperature for 4.5 h. The bright yellow, transparent solution was quenched via addition of aqueous, saturated ammonium chloride solution (5 mL) and the resulting biphasic solution was separated. The aqueous layer was repeatedly extracted with ethyl acetate (5 x 10 mL). Combined organic fractions were dried over anhydrous sodium sulfate, filtered and concentrated under vacuum. The crude residue was purified by via flash column chromatography (5% MeOH/dichloromethane) providing the desired thioether **5** as a viscous, bright orange oil (7.2 mg, 0.021 mmol, 38%). R_f = 0.30 (5% MeOH/dichloromethane); $[\alpha]_D^{21}$ = -30.0 (*c* 0.46, $CHCl_3$); (Compound **5** existed in a ~3:1 mixture of enol (**B**) and keto (**A**) tautomers, respectively) ^{1}H NMR (400 MHz) δ 15.58 (s, 0.75H), 6.66–6.56 (m, 1H), 6.09 (br, 0.25H), 5.96 br, 0.75H), 5.80 (br, 0.75H), 3.96 (d, *J* = 14.7 Hz, 0.25H), 3.79 (d, *J* = 14.7 Hz, 0.25H), 3.61–3.36 (m, 3H), 2.87–2.80 (m, 2H), 2.80 – 2.74 (m, 0.50H), 2.72–2.62 (m, 4.5H), 1.98–2.02 (m, 3H), 1.85 (d, *J* = 1.2 Hz, 2.25H), 1.81 (d, *J* = 1.2 Hz, 0.75H), 1.60–1.48 (m, 1H), 1.47–1.37 (m, 1H), 1.09 (d, *J* = 6.9 Hz, 0.75H), 1.06 (dd, *J* = 6.8 Hz, 2.25H), 1.00–0.93 (m, 3H); (Only the major (enol) tautomer **B** carbon shifts are recorded) ^{13}C NMR ($CDCl_3$, 100 MHz) δ 194.8, 183.7, 170.4, 141.2, 132.2, 96.6, 76.9, 39.7, 39.1, 38.6, 32.2, 27.9, 27.2, 23.4, 16.8, 12.4, 10.2; HRMS (ESI-TOF) *m/z*: $[M + Na]^+$ Calcd for $C_{17}H_{29}NO_4SSi$ 366.1710, Found 366.1716.

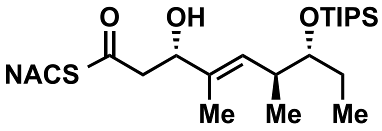

S-(2-acetamidoethyl) (3S,6S,7R,E)-3-hydroxy-4,6-dimethyl-7-((triisopropylsilyl)oxy)non-4-enethioate (24). A reaction vessel containing thiazoldinethione **16** (0.142 g, 0.276 mmol) in anhydrous dichloromethane (2 mL) under argon atmosphere was added imidazole (56.4 mg, 0.828 mmol, 3.00 equiv.). To the clear, yellow solution was added *N*-acetylcysteamine (32.3 µL, 0.304 mmol, 1.10 equiv.) and the resulting reaction mixture was stirred for 14 h at ambient temperature. The crude reaction mixture was wet-loaded onto silica column and purified by flash chromatography (5% MeOH/dichloromethane) affording the desired thioester **24** as a transparent, slightly yellow oil (0.119 g, 0.251 mmol, 91%). R_f = 0.32 (5% MeOH/dichloromethane); $[\alpha]_D^{22}$ = -15.0 (*c* 1.00, $CHCl_3$); ^{1}H NMR (400 MHz) δ 5.82 (s, 1H), 5.49 (d, *J* = 9.7 Hz, 1H), 4.49 (d, *J* = 8.4 Hz, 1H), 3.65 (br, 1H), 3.45 (q, *J* = 5.9 Hz, 2H), 3.05 (t, *J* = 6.2 Hz, 2H), 2.85 (dd, *J* = 15.0, 9.2 Hz, 1H), 2.79 – 2.64 (m, 1H), 2.63–2.51 (m, 1H), 2.34 (br, 1H), 1.97 (s, 3H), 1.65 (s, 3H), 1.54–1.36 (m, 2H), 1.07 (s, 21H), 0.96 (d, *J* = 6.9 Hz, 3H), 0.84 (t, *J* = 7.3 Hz, 3H); ^{13}C NMR ($CDCl_3$, 100 MHz) δ 199.1, 170.5, 134.9, 129.6, 77.7, 74.4, 49.8, 39.6, 36.6, 29.0, 27.6, 23.4, 18.5, 17.0, 13.2, 12.2, 10.4; HRMS (ESI-TOF) *m/z*: [M + Na]$^+$ Calcd for $C_{24}H_{47}NO_4SSiNa$ 496.2887, Found 494.2896.

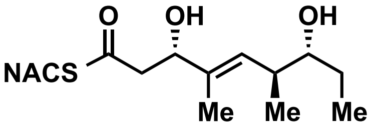

S-(2-acetamidoethyl) (3S,6S,7R,E)-3,7-dihydroxy-4,6-dimethylnon-4-enethioate (6b). A solution of silylether **25** (33.4 mg, 0.0706 mmol) in acetonitrile (3.55 mL) in a polypropylene tube (15 mL, BD Falcon™) was cooled in ice-water bath (0 °C). To the reaction mixture was added a chilled solution of 48% hydrofluoric acid in acetonitrile (11:89, 10.6 mL) and the resulting combined solution was transferred to the refrigerator overnight (15 h). The reaction mixture was neutralized via slow addition of a saturated, aqueous sodium bicarbonate solution at 0 °C. The clear solution was extracted with ethyl acetate (3 x 25 mL). The combined organic layers were dried over anhydrous sodium sulfate, filtered and concentrated under vacuum. Flash column chromatography (10% MeOH/dichloromethane) of the crude residue furnished the title diol as a colorless, clear oil (14.1 mg, 0.0445 mmol, 63%). R_f = 0.44 (10% MeOH/dichloromethane); $[\alpha]_{365}^{22}$ = -6.0 (*c* 0.51, $CHCl_3$); ^{1}H NMR (400 MHz) δ 6.16 (br, 1H), 5.38 (d, *J* = 9.9 Hz, 1H), 4.46 (br, 1H), 3.52–3.35 (m, 2H), 3.33–3.22 (m, 1H), 3.11–2.96 (m, 3H), 2.87 (dd, *J* = 14.7, 7.5 Hz 1H), 2.79 (dd, *J* = 14.5, 4.0 Hz 1H), 2.49–2.39 (m, 1H), 1.96 (s, 3H), 1.67 (s, 3H), 1.62–1.53 (m, 1H), 1.45–1.31 (m, 1H), 1.01–0.88 (m, 6H); ^{13}C NMR ($CDCl_3$, 100 MHz) δ 199.0, 170.7, 137.2, 128.9, 77.1, 74.0, 49.3, 39.3, 38.1, 29.3, 27.2, 23.3, 17.2, 13.1, 10.1; HRMS (ESI-TOF) *m/z*: [M + Na]$^+$ Calcd for $C_{15}H_{27}NO_4SNa$ 340.1553, Found 340.1565.

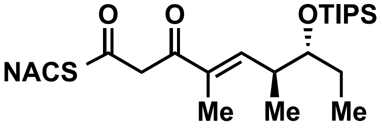

S-(2-acetamidoethyl) (6S,7R,E)-4,6-dimethyl-3-oxo-7-((triisopropylsilyl)oxy)non-4-enethioate (25). To a small round bottom flask was added β-hydroxythioester **24** (20.7 mmol, 0.0437 mmol) as an ethyl acetate solution (2 mL). The colorless solution was diluted with additional ethyl acetate (3 mL) and a superstoichiometric amount of 2-iodoxybenzoic acid (81.8 mg, 0.131 mmol, 3.00 equiv., 45% wt/wt) was added. The white suspension was refluxed for 1 h. After cooling

to ambient temperature the reaction mixture was filtered through celite pad (3 cm thick) and concentrated under vacuum affording the crude β-ketothioester **25** as a yellow oil which was taken to the next step without further purification. R_f = 0.45 (5% MeOH/dichloromethane).

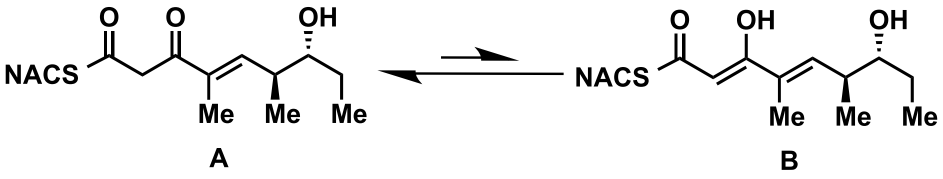

***S*-(2-acetamidoethyl) (6*S*,7*R*,*E*)-7-hydroxy-4,6-dimethyl-3-oxonon-4-enethioate (4).** The crude silylether **25** (20.6 mg, 0.0437 mmol) was transferred as an acetonitrile solution (0.200 mL) to a polypropylene tube (15 mL, BD Falcon™) and cooled via ice-water bath (0 °C). To the pre-chilled solution was added a 48% hydrofluoric acid solution diluted in acetonitrile (11:89, 2.50 mL). The reaction mixture was placed in refrigerator (4 °C) for 24 h. TLC analysis of the reaction mixture indicated incomplete conversion and an addition portion of hydrofluoric acid solution (freshly prepared and identical to above, 1 mL) was added to the reaction mixture at 0 °C. After an additional 12 h at 4 °C, the reaction mixture was neutralized at 0 °C via addition of aqueous, saturated sodium bicarbonate solution. The reaction mixture was extracted with ethyl acetate (4 x 25 mL). The combined organic fractions were dried over anhydrous sodium sulfate, filtered and concentrated under vacuum. Crude product was purified by flash column chromatography (5% MeOH/dichloromethane) yielding a slightly yellow oil (11.8 mg, 0.0374 mmol, 86% from **24**). R_f = 0.25 (5% MeOH/dichloromethane); $[\alpha]_D^{21}$ = -35.8 (*c* 0.79, $CHCl_3$); (Compound **26** exists as in equilibrium of **A** and **B** (~3:1)) ^{1}H NMR (400 MHz) δ 12.71 (br, 0.25H), 6.65 (d, *J* = 9.8 Hz, 0.75H), 6.58 (d, *J* = 10.1 Hz, 0.25H), 6.24 (s, 0.75H), 6.05 (s, 0.25H), 5.66 (s, 0.25H), 4.08 (d, *J* = 15.0 Hz, 0.75H), 3.90 (d, *J* = 15.0 Hz, 0.75H), 3.59–3.33 (m, 3H), 3.13–3.00 (m, 2H), 2.74–2.59 (m, 1H), 2.02–1.91 (m, 3H), 1.85–1.78 (s, 3H), 1.58–1.34 (m, 2H), 1.09 (d, *J* = 6.8 Hz, 2.25H), 1.04 (d, *J* = 6.8 Hz, 0.75H), 1.00–0.90 (m, 3H); (Carbon shifts only given for major tautomer) ^{13}C NMR ($CDCl_3$, 100 MHz) δ 193.8, 193.5, 170.9, 147.7, 137.3, 76.9, 53.2, 39.5, 39.2, 29.5, 28.3, 23.2, 16.8, 11.7, 10.3; HRMS (ESI-TOF) *m/z*: $[M + Na]^+$ Calcd for $C_{15}H_{25}NO_4SSi$ 338.1397, Found 338.1398.

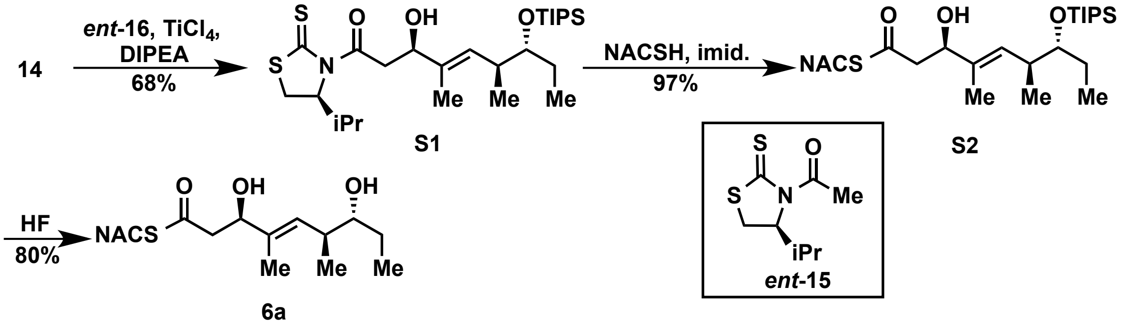

Scheme S1. Synthetic route towards L-alcohol substrate **6a**. An asymmetric aldol reaction between key aldehyde **14** and Nagao's chiral auxiliary *ent*-**15** and displacement of the thiazoldinone with *N*-acetylcysteamine furnished thioester **S2**. Final deprotection with hydrogen fluoride afforded the desired diol substrate **6a**.

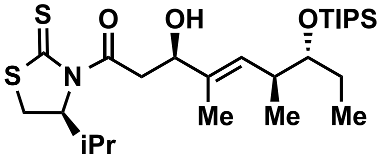

(3*R*,6*S*,7*R*,*E*)-3-hydroxy-1-((*S*)-4-isopropyl-2-thioxothiazolidin-3-yl)-4,6-dimethyl-7-((triisopropylsilyl)oxy)non-4-en-1-one (S1). A reaction vessel containing thiazolidinethione *ent*-**15** (91.9 mg, 0.452 mmol, 1.50 equiv.) in anhydrous dichloromethane (3.00 mL) was cooled in ice-water bath (0 °C). To the chilled, bright yellow solution was added titanium(IV) chloride (55.0 µL, 0.503 mmol, 1.67 equiv.) resulting in a color change to red orange. The reaction was stirred for 5 min, then placed in dry ice-acetone bath (-78 °C) and allowed to equilibrate. To the chilled reaction mixture was added freshly distilled diisopropylethylamine[1] (87.6 µL, 0.503 mmol, 1.67 equiv.) dropwise over 4 min causing a sudden color change to blood red. The solution was stirred at -78 °C for 2 h. To the enolate solution was added aldehyde **14** (94 mg, 0.301 mmol, 1.00 equiv.) as a solution in anhydrous dichloromethane (1 mL) over the course of 6 min. The reaction mixture was stirred at -78 °C for an additional 6 hours and quenched upon addition of saturated, aqueous ammonium chloride solution (5 mL). The biphasic solution was warmed to ambient temperature, separated and the aqueous layer extracted with dichloromethane (4 x 10 mL). The combined, organic fractions were dried over anhydrous sodium sulfate, filtered and concentrated under vacuum. Purification by silicia gel chromatography (20% EtOAc/hexanes) provided the desired aldol adduct as a yellow, viscous oil (0.106 mg, 0.205 mmol, 68%). R_f = 0.31 (20% EtOAc/hexanes); $[\alpha]_D^{22}$ = 217.2 (*c* 1.00, $CHCl_3$); ^1H NMR (400 MHz) δ 5.51 (d, *J* = 9.5 Hz, 1H), 5.15 (t, *J* = 6.5 Hz, 1H), 4.58 (d, *J* = 9.3 Hz, 1H), 3.65 (br, 1H), 3.56 – 3.38 (m, 3H), 3.03 (d, *J* = 11.5 Hz, 1H), 2.58 (br, 1H), 2.45 – 2.32 (m, 1H), 1.67 (s, 3H), 1.54–1.35 (m, 2H), 1.07 (app. s, 24H), 0.97 (t, *J* = 6.0 Hz, 6H), 0.84 (t, *J* = 7.0 Hz, 3H); ^{13}C NMR ($CDCl_3$, 100 MHz) δ 203.1, 173.0, 135.1, 129.3, 77.7, 77.4, 73.5, 71.7, 44.2, 36.6, 31.0, 27.7, 19.3, 18.5, 18.0, 17.0, 13.2, 12.4, 10.4; HRMS (ESI-TOF) *m/z*: $[M + Na]^+$ Calcd for $C_{26}H_{49}NO_3S_2SiNa$ 538.2815, Found 538.2821.

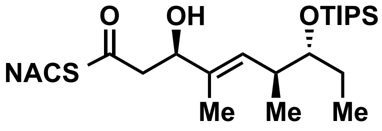

***S*-(2-acetamidoethyl) (3*R*,6*S*,7*R*,*E*)-3-hydroxy-4,6-dimethyl-7-((triisopropylsilyl)oxy)non-4-enethioate (S2).** To a reaction vessel containing acyl thiazolidinethione **S1** (66.0 mg, 0.128 mmol) in anhydrous dichloromethane

(2 mL) was sequentially added imidazole (26.0 mg, 0.384 mmol, 3.00 equiv.) and *N*-acetylcysteamine (16.0 µL, 0.154 mmol, 1.20 equiv.). The transparent, yellow solution was vigorously stirred under argon atmosphere for 15 h at ambient temperature. The reaction was quenched upon addition of an aqueous, saturated ammonium chloride solution (3 mL) and the biphasic solution was separated. The aqueous layer was extracted with dichloromethane (3 x 10 mL). The combined organic layers were dried over anhydrous sodium sulfate, filtered, concentrated under vacuum and purified via silica column flash chromatography (5% MeOH/dichloromethane) using a small plug of copper(II) sulfate impregnated silica gel (1 cm on top) furnished the title compound as a colorless oil (59.0 mg, 0.125 mmol, 97%). R_f = 0.35 (5% MeOH/dichloromethane); $[\alpha]_D^{22}$ = -7.4 (*c* 1.00, $CHCl_3$); ^{1}H NMR (400 MHz) δ 5.94 (br, 1H), 5.48 (d, *J* = 9.7 Hz, 1H), 4.49 (dd, *J* = 9.2, 3.4 Hz, 1H), 3.71–3.56 (m, 1H), 3.44 (q, *J* = 6.1 Hz, 2H), 3.13–2.94 (m, 2H), 2.85 (dd, *J* = 15.0, 9.2 Hz, 1H), 2.71 (dd, *J* = 14.9, 3.6 Hz, 1H), 2.55 (ddd, *J* = 9.9, 6.8, 3.2 Hz, 1H), 2.47 (br, 1H), 1.96 (s, 3H), 1.64 (d, *J* = 1.1 Hz, 3H), 1.52–1.30 (m, 2H), 1.06 (s, 21H), 0.98 (d, *J* = 6.9 Hz, 3H), 0.82 (t, *J* = 7.4 Hz, 3H); ^{13}C NMR ($CDCl_3$, 100 MHz) δ 199.0, 170.6, 134.9, 129.7, 77.7, 74.5, 49.8, 39.5, 36.5, 28.9, 27.7, 23.3, 18.4, 17.0, 13.1, 12.0, 10.4; HRMS (ESI-TOF) *m/z*: $[M + Na]^+$ Calcd for $C_{24}H_{47}NO_4SSiNa$ 496.2887, Found 496.2881.

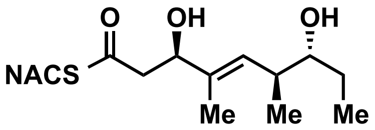

***S*-(2-acetamidoethyl) (3*R*,6*S*,7*R*,*E*)-3,7-dihydroxy-4,6-dimethylnon-4-enethioate (6a).** The silylether **S2** (40.0 mg, 0.0845 mmol) was deprotected in a manner analogous to the production of diol **6b** affording the desired product as a clear, colorless oil (21.5 mg, 0.678 mmol, 80%). R_f = 0.43 (10% MeOH/dichloromethane); $[\alpha]_D^{22}$ = 18.2 (*c* 1.00, $CHCl_3$); ^{1}H NMR (400 MHz) δ 6.19 (br, 1H), 5.34 (d, *J* = 10.0 Hz, 1H), 4.50 (t, *J* = 6.6 Hz, 1H), 3.48 (app. dq, *J* = 12.4, 6.2 Hz, 1H), 3.42–3.31 (m, 1H), 3.30–3.24 (m, 1H), 3.04 (t, *J* = 6.2 Hz, 2H), 2.83 (dd, *J* = 6.6, 3.1 Hz, 2H), 2.62 (br, 1H), 2.49–2.39 (m, 1H), 1.96 (s, 3H), 1.69 (d, *J* = 1.1 Hz 3H), 1.63–1.52 (m, 1H), 1.43–1.31 (m, 1H), 0.96 (t, *J* = 7.3 Hz, 3H), 0.96 (d, *J* = 6.7 Hz, 3H); ^{13}C NMR ($CDCl_3$, 100 MHz) δ 198.6, 170.7, 137.2, 129.9, 77.1, 74.5, 49.7, 39.4, 38.2, 29.3, 27.2, 23.3, 17.2, 12.2, 10.1; HRMS (ESI-TOF) *m/z*: $[M + Na]^+$ Calcd for $C_{15}H_{27}NO_4SiNa$ 340.1553, Found 340.1554.

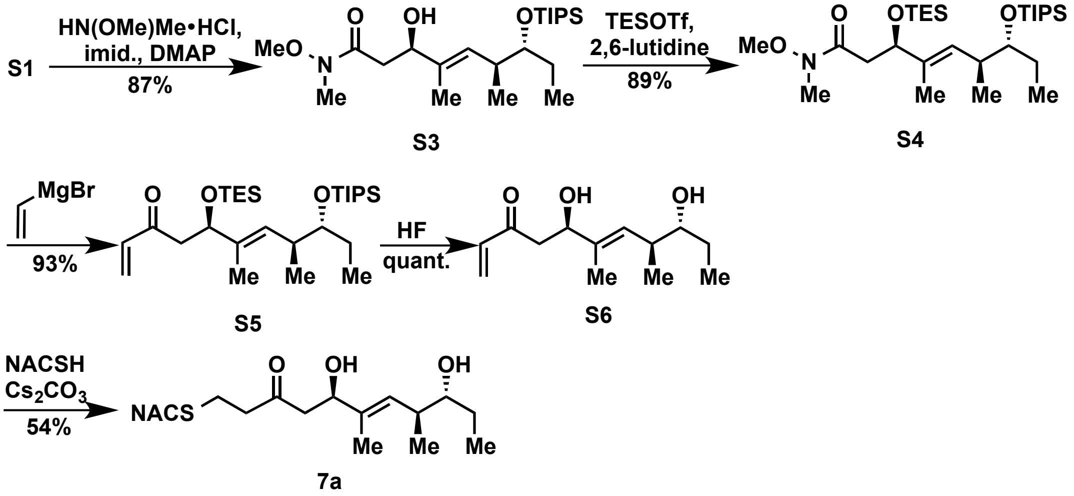

Scheme S2. Chemical synthesis of thioether substrate **7a**. Aldol adduct **S1** was sequentially converted to the corresponding weinreb amide (**S3**) followed by protection with triethylsilyl trifluromethanesulfonate providing disilylether **S4**. Grignard addition and deprotection followed by Michael addition afforded thioether **7a**.

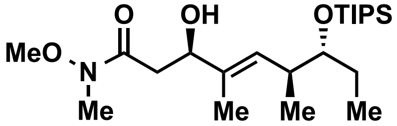

(3*R*,6*S*,7*R*,*E*)-3-hydroxy-*N*-methoxy-*N*,4,6-trimethyl-7-((triisopropylsilyl)oxy)non-4-enamide (S3). To a reaction vessel containing acyl thiazolidinethione **S1** (0.298 g, 0.578 mmol) in anhydrous dichloromethane (3 mL) was sequentially added imidazole (0.197 g, 2.89 mmol, 5.00 equiv.) and *N*,*O*-dimethylhydroxylamine hydrochloride (0.141 g, 1.45 mmol, 2.50 equiv.). The reaction mixture was stirred at ambient temperature under argon atmosphere for 16 h and quenched upon addition of a saturated, aqueous ammonium chloride solution (4 mL). The biphasic solution was separated and the aqueous layer was extracted with dichloromethane. Combined organic fractions were dried over anhydrous sodium sulfate, filtered, concentrated under vacuum and purified via silica flash column chromatography (40% EtOAc/hexanes) yielding the desired amide as a slightly yellow, clear oil (0.208 g, 0.501 mmol, 87%). R_f = 0.37 (40% EtOAc/hexanes); $[\alpha]_D^{23}$ = 26.3 (*c* 1.00, $CHCl_3$); ^1H NMR (400 MHz) δ 5.48 (d, *J* = 9.6 Hz, 1H), 4.47–4.41 (m, 1H), 3.67 (s, 3H), 3.66–3.60 (m, 2H), 3.19 (s, 3H), 2.69–2.51 (m, 3H), 1.67 (s, 3H), 1.52–1.36 (m, 2H), 1.06 (s, 21H), 0.99 (d, *J* = 6.9 Hz, 3H), 0.84 (t, *J* = 7.4 Hz, 3H); ^{13}C NMR ($CDCl_3$, 100 MHz) δ 173.9, 135.4, 129.8, 77.8, 73.6, 61.4, 37.3, 36.6, 32.0, 27.6, 18.4, 17.0, 13.1, 12.3, 10.4; HRMS (ESI-TOF) *m/z*: $[M + Na]^+$ Calcd for $C_{22}H_{45}NO_4SiNa$ 438.3010, Found 438.3010.

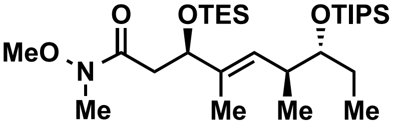

(3*R*,6*S*,7*R*,*E*)-*N*-methoxy-*N*,4,6-trimethyl-3-((triethylsilyl)oxy)-7-((triisopropylsilyl)oxy)non-4-enamide (S4). To a small, round bottom flask containing β-hydroxy amide **S3** (79.0 mg, 0.190 mmol) under argon atmosphere in an ice-water bath (0 °C) was added anhydrous dichloromethane (3.5 mL). The resulting clear solution was supplemented with 2,6-lutidine (89.0 μL, 0.761 mmol, 4.00 equiv.) and triethylsilyl trifluoromethanesulfonate (86.0 μL, 0.380 mmol, 2.00 equiv.). The reaction mixture was stirred at 0 °C for 2.5 h and quenched upon sequential addition of methanol (2 mL) and saturated, aqueous sodium bicarbonate

solution (2 mL). The biphasic solution was warmed to ambient temperature and the layers were separated. The aqueous layer was extracted with dichloromethane (3 x 10 mL) and the combined organic fractions were dried over anhydrous sodium sulfate, filtered and concentrated under vacuum. The crude residue was purified by silica flash column chromatography (10-20% EtOAc/hexanes) producing the disilylether **S4** as a transparent, clear oil (89.3 mg, 0.169 mmol, 89%). R_f = 0.58 (20% EtOAc/hexanes); $[\alpha]_D^{22}$ = 9.7 (*c* 1.00, $CHCl_3$); ^{1}H NMR (400 MHz) δ 5.45 (d, *J* = 9.5 Hz, 1H), 4.59 (dd, *J* = 8.8, 4.5 Hz, 1H), 3.70 (s, 3H), 3.61 (td, *J* = 6.5, 2.8 Hz, 1H), 3.16 (s, 3H), 2.99 – 2.80 (m, 1H), 2.60–2.50 (m, 1H), 2.31 (dd, *J* = 13.9, 4.3 Hz, 1H), 1.64 (d, *J* = 1.1 Hz, 3H), 1.49–1.34 (m, 2H), 1.07 (s, 21H), 1.00 (d, *J* = 6.9 Hz, 3H), 0.91 (t, *J* = 7.9 Hz, 9H), 0.82 (t, *J* = 7.4 Hz, 3H), 0.56 (q, *J* = 7.9 Hz, 6H); ^{13}C NMR ($CDCl_3$, 100 MHz) δ 136.7, 128.2, 77.8, 75.6, 61.4, 39.5, 36.7, 32.1, 27.5, 18.5, 16.5, 13.1. 11.4, 10.7, 6.9, 4.9 (missing amide carbonyl carbon); HRMS (ESI-TOF) *m/z*: $[M + Na]^+$ Calcd for $C_{28}H_{59}NO_4Si_2Na$ 552.3875, Found 552.3905.

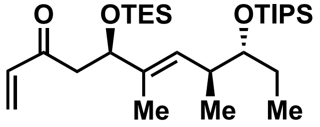

(5*R*,8*S*,9*R*,*E*)-6,8-dimethyl-5-((triethylsilyl)oxy)-9-((triisopropylsilyl)oxy)undeca-1,6-dien-3-one (S5). To a reaction vessel containing Weinreb amide **S4** (37.6 mg, 0.0710 mmol) under argon atmosphere was added anhydrous tetrahydrofuran (5 mL). The resulting clear solution was cooled with ice-water bath (0 °C). A solution of vinylmagnesium bromide (1.00 M) (0.224 mL, 0.224 mmol, 3.16 equiv.) was added to the stirred solution of starting material. After 3 h the reaction was quenched at 0 °C via addition of saturated, aqueous ammonium chloride solution (5 mL) and allowed to warm to ambient temperature. The biphasic solution was separated and the aqueous layer was repeatedly extracted with ethyl acetate (3x 10 mL). The combined organic layers were dried over anhydrous sodium sulfate, filtered and concentrated under vacuum. Purification of the crude residue by silica flash column chromatography (5% EtOAc/hexanes) furnished the vinyl ketone as a clear, light yellow oil (32.7 mg, 0.658 mmol, 93%). R_f = 0.45 (5% EtOAc/hexanes); $[\alpha]_D^{22}$ = 7.6 (*c* 1.00, $CHCl_3$); ^{1}H NMR (400 MHz) δ 6.35 (dd, *J* = 17.6, 10.5 Hz, 1H), 6.19 (d, *J* = 17.6 Hz, 1H), 5.81 (d, *J* = 10.5 Hz, 1H), 5.42 (d, *J* = 9.6 Hz, 1H), 4.55 (dd, *J* = 8.3, 4.7 Hz, 1H), 3.69 – 3.52 (m, 1H), 2.94 (dd, *J* = 14.0, 8.4 Hz, 1H), 2.58–2.47 (m, 2H), 1.62 (s, 3H), 1.50–1.30 (m, 2H), 1.07 (s, 21H), 0.99 (d, *J* = 6.9 Hz, 3H), 0.89 (t, *J* = 7.9 Hz, 9H), 0.81 (t, *J* = 7.4 Hz, 3H), 0.54 (q, *J* = 7.9 Hz, 6H); ^{13}C NMR ($CDCl_3$, 100 MHz) δ 199.4, 137.6, 136.4, 128.4 (2), 77.8, 75.8, 46.9, 36.6, 27.6, 18.4, 16.6, 13.2, 11.3, 10.6, 7.0, 4.9; HRMS (ESI-TOF) *m/z*: $[M + Na]^+$ Calcd for $C_{28}H_{56}NO_3Si_2Na$ 519.3660, Found 519.3674.

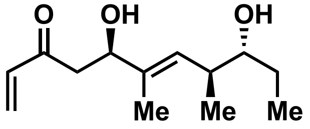

(5*R*,8*S*,9*R*,*E*)-5,9-dihydroxy-6,8-dimethylundeca-1,6-dien-3-one (S6). Disilylether **S5** (10.9 mg, 0.220 mmol) was transferred to a small polypropylene tube (15 mL, BD Falcon™) and cooled via ice-water bath (0 °C). The diol was dissolved in acetonitrile (1 mL) and to the resulting clear solution was added a solution of 48% hydrofluoric acid in acetonitrile (11:89, 3.2 mL). The reaction mixture was placed in a refrigerator (4 °C) for 19 h and quenched via addition of an aqueous, saturated sodium bicarbonate solution until the mixture was neutralized (pH = 7). Extraction of the reaction mixture with ethyl acetate (4 x 15 mL), drying over anhydrous sodium sulfate, filtration

and concentration under vacuum gave a crude product residue. Purification via flash column chromatography (50% EtoAc/hexanes) provided the desired diol as a cloudy, colorless oil (4.97 mg, 0.220 mmol, quant.). R_f = 0.33 (50% EtOAc/hexanes); $[\alpha]_D^{21}$ = 25.6 (*c* 0.87, $CHCl_3$); ^{1}H NMR (400 MHz) δ 6.37 (dd, *J* = 17.6, 10.4 Hz, 1H), 6.26 (d, *J* = 17.6 Hz, 1H), 5.90 (d, *J* = 10.4 Hz, 1H), 5.37 (d, *J* = 10.0 Hz, 1H), 4.53 (dd, *J* = 8.4, 4.0 Hz, 1H), 3.34–3.27 (m, 1H), 2.88 (dd, *J* = 8.4, 16.8 Hz, 1H), 2.80 (dd, *J* = 16.8, 4.4 Hz, 1H) 2.52 – 2.38 (m, 1H), 1.69 (s, 3H), 1.61–1.51 (m, 1H), 1.43– 1.31(m, 1H), 0.97 (d, *J* = 6.8 Hz, 3H), 0.97 (t, *J* = 7.6 Hz, 3H); ^{13}C NMR ($CDCl_3$, 100 MHz) δ 200.8, 137.4, 136.8, 129.5, 129.0, 77.1, 73.2, 44.7, 38.1, 27.2, 17.2, 12.6, 10.2; HRMS (ESI-TOF) *m/z*: [M + Na]$^+$ Calcd for $C_{13}H_{22}O_3Na$ 249.1461, Found 249.1463.

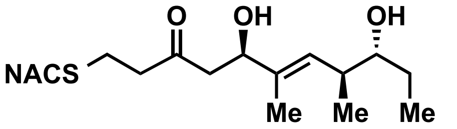

***N*-(2-(((5*R*,8*S*,9*R*,*E*)-5,9-dihydroxy-6,8-dimethyl-3-oxoundec-6-en-1-yl)thio)ethyl)acetamide (7a).** To a small round bottom flask containing vinyl ketone **S6** (26.0 mg, 0.115 mmol) in anhydrous tetrahydrofuran (9 mL) under argon atmosphere was added *N*-acetylcysteamine (14.6 μL, 0.138 mmol, 1.20 equiv.). A catalytic amount of cesium carbonate was added to the reaction mixture and the solution was stirred at ambient temperature for 11 h. The reaction was concentrated under vacuum and purified via silica flash column chromatography (10% MeOH/dichloromethane, 1 cm plug of Cu(II)SO_4-silica on top) yielding the title compound as a colorless, cloudy oil (21.4 mg, 0.0620 mmol, 54%). R_f = 0.46 (10% MeOH/dichloromethane); $[\alpha]_D^{22}$ = 5.9 (*c* 0.59, $CHCl_3$); ^{1}H NMR (400 MHz) δ 6.17 (s, 1H), 5.36 (d, *J* = 10.0 Hz, 1H), 4.48 (dd, *J* = 8.1, 3.5 Hz, 1H), 3.43 (q, *J* = 6.3 Hz, 2H), 3.33–3.26 (m, 1H), 2.87 (br, 1H), 2.80 – 2.74 (m, 4H), 2.74 – 2.58 (m, 4H), 2.49 – 2.38 (m, 1H), 1.99 (s, 3H), 1.81 (s, 1H), 1.65 (s, 3H), 1.61–1.50 (m, 1H), 1.36 (dq, *J* = 14.1, 7.4 Hz, 1H), 0.96 (d, *J* = 7.4 Hz, 3H), 0.95 (t, *J* = 7.7 Hz, 3H); ^{13}C NMR ($CDCl_3$, 100 MHz) δ 209.1, 170.5, 137.4, 128.9, 77.4, 73.2, 48.4, 43.6, 38.7, 38.0, 32.2, 27.3, 25.2, 23.4, 17.3, 12.6, 10.2; HRMS (ESI-TOF) *m/z*: [M + Na]$^+$ Calcd for $C_{17}H_{31}NO_4SNa$ 368.1866, Found 368.1873.

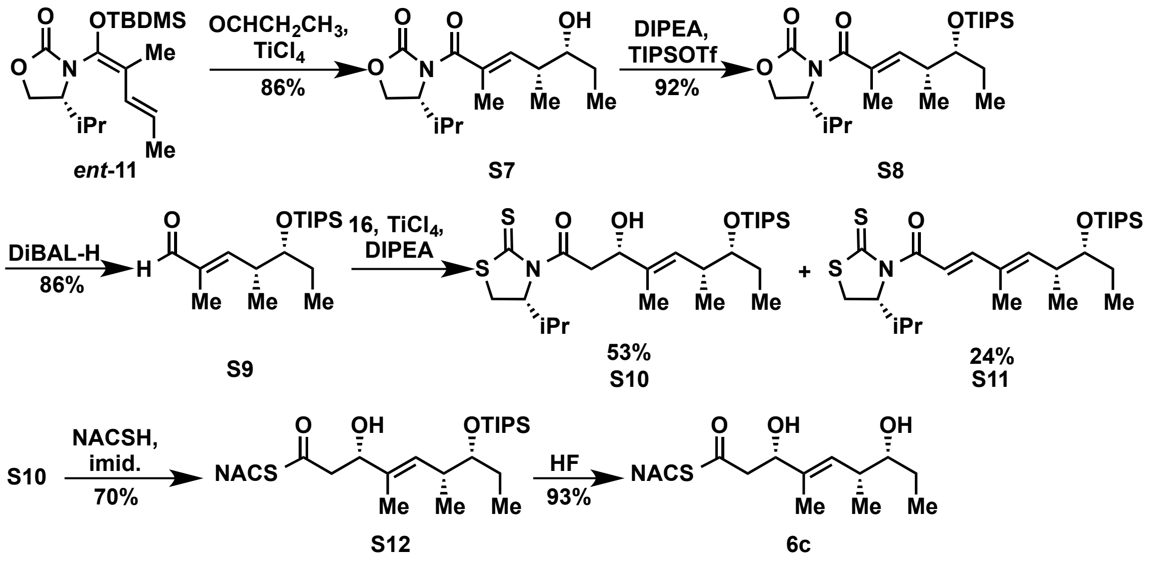

Scheme S3. Synthesis of TylDH3 substrate **6c**. The outlined route closely parallels that of thioester **6b** (**Schemes 1** and **2** in manuscript). Notable differences include the use of the enantiomer of **11** (ent-**11**), the *syn*-selective vinylogous aldol promoted by excess titanium(IV) chloride, and the production of dehydration product **S12** due to an increased sensitivity of diastereomer **S11** to prolonged aldol conditions.

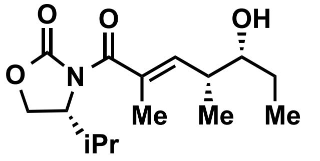

(*R*)-3-((4*R*,5*R*,*E*)-5-hydroxy-2,4-dimethylhept-2-enoyl)-4-isopropyloxazolidin-2-one (S7). This procedure is adapted from that of Hosokawa and coworkers.[3] To a reaction vessel containing *ent*-**11**[4] (0.720 g, 2.12 mmol, 1.50 equiv.) in anhydrous dichloromethane (42 mL) under argon atmosphere was added freshly distilled propionaldehyde[1] (0.102 mL, 1.41 mmol, 1.00 equiv.). The resulting solution was cooled to -78 °C (acetone-dry ice). Titanium(IV) chloride (0.621 mL, 5.65 mmol, 4.00 equiv.) was added, in one portion, to the chilled solution. The reaction mixture immediately developed a dark blue hue. The reaction was stirred at -78 °C for 17 h and quenched via addition saturated aqueous sodium bicarbonate solution (10 mL) and saturated aqueous potassium sodium tartrate (10 mL). The biphasic solution was warmed to ambient temperature, separated and the aqueous layer was extracted with dichloromethane (3 x 20 mL). The combined organic layers were dried over anhydrous sodium sulfate, filtered and concentrated under vacuum. Purification via flash column chromatography (30% EtOAc/hexanes) furnished aldol adduct **S7** as a colorless, clear oil (0.344 g, 1.21 mmol, 86%). R_f = 0.26 (30% EtOAc/hexanes); $[\alpha]_D^{22}$ = -65.2 (*c* 1.00, $CHCl_3$); ^1H NMR (400 MHz) δ 5.92 (d, *J* = 10.8 Hz, 1H), 4.56 (dt, *J* = 9.6, 5.0 Hz, 1H), 4.33 (t, *J* = 9.0 Hz, 1H), 4.18 (dd, *J* = 8.9, 5.6 Hz, 1H), 3.55 (dt, *J* = 8.8, 4.2 Hz, 1H), 2.76 – 2.64 (m, 1H), 2.40 – 2.27 (m, 1H), 1.94 (d, *J* = 1.3 Hz, 3H), 1.63–1.51 (m, 1H), 1.45 – 1.31 (m, 1H), 1.03 (d, *J* = 6.8 Hz, 3H), 0.98 (t, *J* = 7.3 Hz, 3H), 0.93 (d, 5.5 Hz, 3H), 0.91 (d, 5.7 Hz, 3H); ^{13}C NMR ($CDCl_3$, 100 MHz) δ 171.9, 154.3, 142.5, 130.6, 77.4, 63.6, 58.3, 38.6, 28.5, 26.5, 18.0, 15.3, 14.3, 13.9, 10.8; HRMS (ESI-TOF) *m/z*: $[M + Na]^+$ Calcd for $C_{15}H_{25}NO_4Na$ 306.1678, Found 306.1680.

(*S*)-3-((4*S*,5*S*,*E*)-5-hydroxy-2,4-dimethylhept-2-enoyl)-4-isopropyloxazolidin-2-one (*ent*-S7). The title compound was synthesized in an analogous matter to its enantiomer and was identical with respect to ^{1}H-NMR and ^{13}C-NMR spectra. $[\alpha]_D^{22}$ = 66.0 (*c* 1.00, $CHCl_3$); HRMS (ESI-TOF) *m*/*z*: $[M + Na]^+$ Calcd for $C_{15}H_{25}NO_4Na$ 306.1678, Found 306.1682.

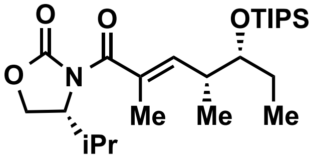

(*R*)-3-((4*R*,5*R*,*E*)-2,4-dimethyl-5-((triisopropylsilyl)oxy)-hept-2-enoyl)-4-isopropyloxazolidin-2-one (S8). To a reaction flask containing aldol adduct **S7** (0.260 g, 0.918 mmol) in anhydrous dichloromethane (10 mL) under argon atmosphere in ice-water bath (0 °C) was added diisopropylethylamine (0.192 mL, 1.10 mmol, 1.20 equiv.) followed by triisopropylsilyl trifluoromethanesulfonate (0.296 mL, 1.10 mmol, 1.20 equiv.). The reaction was stirred at 0 °C for 19 h and quenched upon addition of an aqueous, saturated sodium bicarbonate solution (5 mL). The biphasic mixture was allowed to warm to ambient temperature and separated. The aqueous layer was extracted with dichloromethane (3x 20 mL) and the combined organic fractions were dried over anhydrous sodium sulfate, filtered and concentrated under vacuum. The crude residue was purified by flash column chromatography on silica gel (10% EtOAc/hexanes) affording the desired silyl ether as a colorless oil (0.373 mg, 0.848 mmol, 92%). R_f = 0.32 (10% EtOAc/hexanes); $[\alpha]_D^{22}$ = -42.6 (*c* 1.00, $CHCl_3$); ^{1}H NMR (400 MHz) δ 6.04 (dd, *J* = 10.0, 1.4 Hz, 1H), 4.53 – 4.43 (m, 1H), 4.30 (t, *J* = 8.8 Hz, 1H), 4.17 (dd, *J* = 8.9, 5.4 Hz, 1H), 3.75 (q, *J* = 5.2 Hz, 1H), 2.72 – 2.59 (m, 1H), 2.46–2.34 (m, 1H), 1.92 (d, *J* = 1.4 Hz, 3H), 1.66 – 1.54 (m, 2H), 1.07 (s, 21H), 1.01 (d, *J* = 6.8 Hz, 3H), 0.92 (d, *J* = 7.2 Hz, 3H), 0.90 (d, *J* = 7.0 Hz, 3H), 0.88 (t, 7.5 Hz, 3H); ^{13}C NMR ($CDCl_3$, 100 MHz) δ 172.2, 153.7, 142.3, 129.7, 76.2, 63.5, 58.6, 36.7, 28.3, 28.0, 18.4, 18.1, 15.1, 14.1, 13.8, 13.1, 9.1; HRMS (ESI-TOF) *m*/*z*: $[M + Na]^+$ Calcd for $C_{24}H_{45}NO_4SiNa$ 462.3010, Found 462.2986.

(*S*)-3-((4*S*,5*S*,*E*)-2,4-dimethyl-5-((triisopropylsilyl)oxy)hept-2-enoyl)-4-isopropyloxa-zolidin-2-one (*ent*-S8). The title compound was synthesized in an analogous matter to its enantiomer and was identical with respect to ^{1}H-NMR and ^{13}C-NMR spectra. $[\alpha]_D^{22}$ = 45.1 (*c* 1.00, $CHCl_3$); HRMS (ESI-TOF) *m*/*z*: $[M + Na]^+$ Calcd for $C_{24}H_{45}NO_4SiNa$ 462.3010, Found 462.3017.

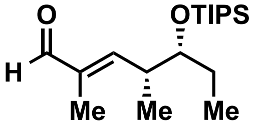

(4*R*,5*R*,*E*)-2,4-dimethyl-5-((triisopropylsilyl)oxy)hept-2-enal (S9). A round bottom flask containing acyl oxazolidinone **S8** (0.373 g, 0.848 mmol) dissolved in anhydrous dichloromethane (27 mL) under argon atmosphere was cooled to – 78 °C (dry ice-acetone). To the chilled reaction mixture was added a toluene solution of diisobutylaluminum hydride (1.49 M, 1.14 mL, 1.70 mmol, 2 equiv.) dropwise over two minutes. The reaction mixture was stirred at – 78 °C for 13 min and quenched upon sequential addition of methanol (10 mL) and saturated, aqueous potassium sodium tartrate solution (10 mL). The biphasic solution was allowed to warm to ambient temperature and separated. The aqueous layer was extracted with dichloromethane (3 x 15 mL) and the combined organic fractions were dried over anhydrous sodium sulfate, filtered and concentrated under vacuum. The crude product was purified via flash column

chromatography (10% EtOAc/hexanes) to provide the aldehyde **S9** as a clear and colorless oil (0.229 g, 0.733 mmol, 86%). R_f = 0.56 (10% EtOAc/hexanes); $[\alpha]_D^{22}$ = -17.7 (*c* 1.00, $CHCl_3$); ^1H NMR (400 MHz) δ 9.39 (s, 1H), 6.55 (d, *J* = 9.8 Hz, 1H), 3.79 (dt, *J* = 8.1, 4.4 Hz, 1H), 2.93 – 2.78 (m, 1H), 1.76 (s, 3H), 1.72 – 1.49 (m, 2H), 1.10–1.04 (s, 24H), 0.89 (t, *J* = 7.5 Hz, 3H); ^{13}C NMR ($CDCl_3$, 100 MHz) δ 195.8, 158.7, 137.9, 76.4, 37.3, 27.9, 18.4, 13.6, 13.1, 9.52, 9.46; HRMS (ESI-TOF) *m/z*: $[M + Na]^+$ Calcd for $C_{18}H_{36}O_2SiNa$ 335.2377, Found 335.2396.

(4*S*,5*S*,*E*)-2,4-dimethyl-5-((triisopropylsilyl)oxy)hept-2-enal (*ent*-S9). The title compound was synthesized in an analogous matter to its enantiomer and was identical with respect to ^1H-NMR and ^{13}C-NMR spectra. $[\alpha]_D^{23}$ = 18.5 (*c* 1.00, $CHCl_3$); HRMS (ESI-TOF) *m/z*: $[M + Na]^+$ Calcd for $C_{18}H_{36}O_2SiNa$ 335.2377, Found 335.2375.

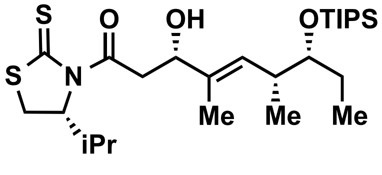

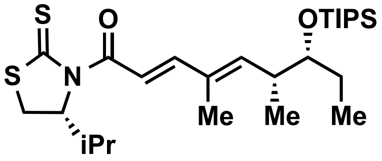

(3*S*,6*R*,7*R*,*E*)-3-hydroxy-1-((*R*)-4-isopropyl-2-thioxothiazolidin-3-yl)-4,6-dimethyl-7-((triisopropylsilyl)oxy)non-4-en-1-one (S10).

(2*E*,4*E*,6*R*,7*R*)-1-((*R*)-4-isopropyl-2-thioxothiazolidin-3-yl)-4,6-dimethyl-7-((triisopropylsilyl)oxy)nona-2,4-dien-1-one (S11). A reaction vessel containing acyl thiazolidinethione **16** (0.126 g, 0.620 mmol, 1.70 equiv.) in anhydrous dichloromethane (2.3 mL) under argon atmosphere was cooled to -40 °C (dry ice-acetonitrile bath). The equilibrated solution was supplemented by titanium(IV) chloride (72.0 μL, 0.657 mmol, 1.80 equiv.) and stirred for 30 min. To the reaction was slowly added freshly distilled diisopropylethylamine[1] (0.114 mL, 0.657 mmol, 1.80 equiv.). The blood red reaction mixture was stirred for 2 h and transferred to dry ice-acetone bath (-78 °C). A dichloromethane (1.3 mL) solution of aldehyde **S9** (0.114 g, 0.365 mmol, 1.00 equiv.) was slowly added to the cooled solution over 12 min. After stirring for 4 h at -78 °C the reaction was quenched via addition of ammonium chloride (5 mL). The biphasic mixture was warmed to ambient temperature and separated. The aqueous layer was extracted with dichloromethane (3 x 10 mL). Combined organic layers were dried over anhydrous sodium sulfate, filtered and concentrated under vacuum. The resulting crude oil was purified via flash chromatography (20% EtOAc/hexanes) to give both the aldol adduct **S10** as a thick, bright yellow oil (99.3 mg, 0.192 mmol, 53%) and the dehydration product **S11** as a viscous, yellow oil (44.4 mg, 0.0893 mmol, 24%). For **S10**: R_f = 0.25 (20% EtOAc/hexanes); $[\alpha]_D^{22}$ = -189.2 (*c* 1.00, $CHCl_3$); ^1H NMR (400 MHz) δ 5.49 (d, *J* = 9.6 Hz, 1H), 5.16 (t, *J* = 7.0 Hz, 1H), 4.56 (d, *J* = 9.3 Hz, 1H), 3.65 (dt, *J* = 6.6, 4.7 Hz, 1H), 3.53 (dd, *J* = 7.7, 3.7 Hz, 1H), 3.51–3.47 (m, 1H), 3.40 (dd, *J* = 17.4, 9.6 Hz, 1H), 3.03 (d, *J* = 11.5 Hz, 1H), 2.60–2.48 (m, 2H), 2.38 (app. sextet, *J* = 6.7 Hz, 1H), 1.67 (s, 3H), 1.61 – 1.46 (m, 2H), 1.09–1.03 (m, 24H), 0.99 (d, *J* = 7.0 Hz, 3H), 0.96 (d, *J* = 0.68 Hz, 3H), 0.86 (t, *J* = 7.4 Hz, 3H); ^{13}C NMR ($CDCl_3$, 100 MHz) δ 203.1, 173.1, 134.0, 131.4, 77.3, 73.6, 71.6, 44.2, 36.0, 31.0, 30.8, 27.8, 19.2, 18.5, 18.0, 15.2, 13.2, 12.2, 9.4; HRMS (ESI-TOF) *m/z*: $[M + Na]^+$ Calcd for $C_{26}H_{49}NO_3S_2SiNa$ 538.2815, Found 538.2841.

For **S11**: R_f = 0.70 (20% EtOAc/hexanes); $[\alpha]_D^{22}$ = -207.8 (*c* 0.23, $CHCl_3$); ^{1}H NMR (400 MHz) δ 7.37 (d, *J* = 15.2 Hz, 1H), 7.32 (d, *J* = 15.3 Hz, 1H), 5.97 (d, *J* = 9.9 Hz, 1H), 5.07 (ddd, *J* = 8.2, 5.6, 2.6 Hz, 1H), 3.72 (dt, *J* = 6.9, 4.6 Hz, 1H), 3.50 (dd, *J* = 11.4, 8.1 Hz, 1H), 3.09 (dd, *J* = 11.4, 2.6 Hz, 1H), 2.74 – 2.64 (m, 1H), 2.54 – 2.41 (m, 1H), 1.82 (d, *J* = 1.1 Hz, 3H), 1.64 – 1.47 (m, 2H), 1.09–1.03 (m, 24H), 1.00 (d, *J* = 6.9 Hz, 6H), 0.86 (t, *J* = 7.5 Hz, 3H); ^{13}C NMR ($CDCl_3$, 100 MHz) δ 202.6, 167.5, 150.4, 147.3, 132.5, 118.1, 72.4, 37.4, 30.9, 30.7, 28.0, 19.2, 18.5, 18.4, 17.4, 14.8, 13.2, 12.7, 9.3; HRMS (ESI-TOF) *m/z*: $[M + Na]^+$ Calcd for $C_{26}H_{47}NO_2S_2SiNa$ 520.2710, Found 520.2713.

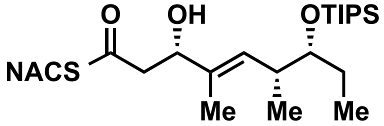

***S*-(2-acetamidoethyl) (3*S*,6*R*,7*R*,*E*)-3-hydroxy-4,6-dimethyl-7-((triisopropylsilyl)oxy)non-4-enethioate (S12).** To a small round bottom flask containing acyl thiazolidinethione **S10** (50.5 mg, 0.0979 mmol) under argon atmosphere was added anhydrous dichloromethane (3 mL). A super-stoichiometric amount of imidazole (20.0 mg, 0.294 mmol, 3.00 equiv.) was added to the rapidly stirred, yellow mixture at ambient temperature. A slight excess of *N*-acetylcysteamine (11.5 µL, 0.108 mmol, 1.10 equiv.) was added to the reaction solution. After stirring for 13.5 h at ambient temperature, the mixture was concentrated under vacuum and purified by flash column chromatography (5% MeOH/dichloromethane, 1 cm thick $CuSO_4$-SiO_2) affording the desired thioester as a clear, colorless wax (32.3 mg, 0.682 mmol, 70%). R_f = 0.30 (5% MeOH/dichloromethane); $[\alpha]_D^{23}$ = -2.5 (*c* 1.00, $CHCl_3$); ^{1}H NMR (400 MHz) δ 5.80 (br, 1H), 5.48 (d, *J* = 9.6 Hz, 1H), 4.48 (dd, *J* = 9.4, 3.0 Hz, 1H), 3.64 (dt, *J* = 6.8, 4.7 Hz, 1H), 3.46 (app. dq, *J* = 6.6, 1.8 Hz, 1H), 3.06 (q, *J* = 6.3 Hz, 1H), 2.84 (dd, *J* = 15.0, 9.5 Hz, 1H), 2.70 (dd, *J* = 15.0, 3.2 Hz, 1H), 2.56–2.46 (m, 1H), 1.97 (s, 3H), 1.65 (d, *J* = 1.2 Hz, 3H), 1.62–1.41 (m, 2H), 1.06 (s, 21H), 0.95 (d, *J* = 6.8 Hz, 3H), 0.85 (t, *J* = 7.4 Hz, 3H); ^{13}C NMR ($CDCl_3$, 100 MHz) δ 199.2, 170.5, 133.8, 131.7, 77.4, 74.5, 49.7, 39.6, 36.0, 29.0, 27.8, 23.4, 18.5, 15.2, 13.2, 12.0, 9.3; HRMS (ESI-TOF) *m/z*: $[M + Na]^+$ Calcd for $C_{24}H_{47}NO_4SSiNa$ 496.2887, Found 496.2887.

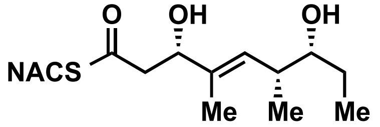

***S*-(2-acetamidoethyl) (3*S*,6*R*,7*R*,*E*)-3,7-dihydroxy-4,6-dimethylnon-4-enethioate (6c).** A polypropylene tube (15.0 mL, BD Falcon™) containing silylether **S12** (32.3 mg, 0.682 mmol) dissolved in acetonitrile (3.40 mL) was placed in ice-water bath (0 °C) and allowed to equilibrate. To the chilled reaction vessel was added a solution of 48% hydrofluoric acid in acetonitrile (11:89, 8.9 mL). The reaction mixture was transferred to a refrigerator (4 °C) for 48 h and quenched at 0 °C via neutralization by saturated aqueous sodium bicarbonate solution. The resulting mixture was extracted with ethyl acetate (4 x 25 mL). The combined organic layers were dried over anhydrous sodium sulfate, filtered and concentrated under vacuum. The crude product residue was purified via silica flash column chromatography (10% MeOH/dichloromethane) furnishing the title compound as a clear, colorless oil (20.2 mg, 0.0630 mmol, 93%). R_f = 0.26 (10% MeOH/dichloromethane); $[\alpha]_{365}^{22}$= 5.0 (*c* 1.47, $CHCl_3$); ^{1}H NMR (400 MHz) δ 6.23 (br, 1H), 5.35 (d, *J* = 9.7 Hz, 1H), 4.45 (dd, *J* = 7.8, 4.8 Hz, 1H), 3.41 (app. hept, *J* = 7.5 Hz, 2H), 3.34–3.28 (m, 1H), 3.08–2.95 (m, 2H), 2.82 (d, *J* = 8.2 Hz, 1H), 2.75 (d, *J* = 4.7 Hz, 1H), (dq, *J* = 12.8, 6.4 Hz, 1H), 1.95 (s, 3H),

1.65 (s, 3H), 1.56–1.43 (m, 1H), 1.35–1.22 (m, 1H), 0.96 (d, J = 6.8 Hz, 3H), 0.92 (t, 7.4 Hz, 3H); ^{13}C NMR ($CDCl_3$, 100 MHz) δ 198.7, 170.8, 135.7, 129.8, 77.2, 74.1, 49.8, 39.4, 37.8, 29.0, 27.1, 23.3, 15.7, 12.4, 10.6; HRMS (ESI-TOF) *m/z*: $[M + Na]^+$ Calcd for $C_{15}H_{27}NO_4SNa$ 340.1553, Found 340.1547.

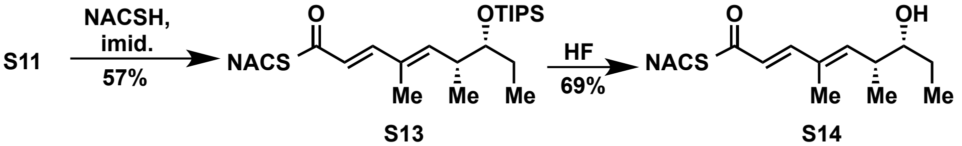

Scheme S4. Synthesis of dehydration product **S14**. By utilizing the serendipitous byproduct **S11**, the result of dehydration during an aldol reaction, we were able to quickly produce the dehydration product **S14**. This dienethioate served as the product standard in LC-MS/MS for the enzymatic dehydration reaction of **27**. Additionally, the same compound was used as the product standard for TylDH3-catalyzed dehydration of **6d**, as it is the enantiomer of the presumed product.

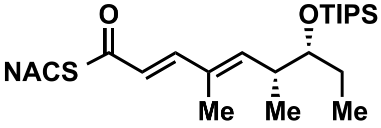

***S*-(2-acetamidoethyl) (2*E*,4*E*,6*R*,7*R*)-4,6-dimethyl-7-((triisopropylsilyl)oxy)nona-2,4-dienethioate (S13).** To a round bottom flask containing acyl thiazolidinethione **S11** (44.2 mg, 0.0889 mmol) under argon atmosphere was added anhydrous dichloromethane (3 mL). To the resulting clear solution was added 4-(dimethylamino)pyridine (32.6 mg, 0.267 mmol, 3.00 equiv.) followed by *N*-acetylcysteamine (10.4 μL, 0.098 mmol, 1.10 equiv.). The resulting mixture was stirred for 10 h at ambient temperature and concentrated under vacuum. Purification of the crude residue via flash column chromatography (5% MeOH/dichloromethane) yielded the desired dienethiolate as a colorless wax (23.1 mg, 0.0507 mmol, 57%). R_f = 0.38 (5% MeOH/dichloromethane); $[\alpha]_D^{23}$= 7.6 (*c* 1.00, $CHCl_3$); ^{1}H NMR (400 MHz) δ 7.25 (d, J = 15.3 Hz, 1H), 6.10 (d, *J* = 15.4 Hz, 1H), 6.01 (d, *J* = 9.8 Hz, 1H), 5.94 (br, 1H), 3.72 (dt, *J* = 7.0, 4.6 Hz, 1H), 3.48 (app. q, *J* = 5.9 Hz, 2H), 3.12 (t, *J* = 6.3 Hz, 2H), 2.76–2.65 (m, 1H), 1.96 (s, 3H), 1.79 (s, 3H), 1.66 –1.46 (m, 2H), 1.08–1.04 (m, 21H), 1.01 (d, *J* = 6.8 Hz, 3H), 0.87 (t, *J* = 7.5 Hz, 3H); ^{13}C NMR ($CDCl_3$, 100 MHz) δ 190.7, 170.4, 148.9, 146.9, 131.4, 122.6, 40.2, 37.5, 28.5, 27.9, 23.4, 18.42, 18.37, 14.7, 13.1, 12.4, 9.3; HRMS (ESI-TOF) *m/z*: $[M + Na]^+$ Calcd for $C_{24}H_{45}NO_3SSiNa$ 478.2781, Found 478.2780.

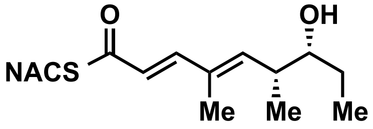

***S*-(2-acetamidoethyl) (2*E*,4*E*,6*R*,7*R*)-7-hydroxy-4,6-dimethylnona-2,4-dienethioate (S14).** A polypropylene tube (15.0 mL, BD Falcon™) containing silylether **S13** (18.0 mg, 0.0395) dissolved in acetonitrile (4.15 mL) was equilibrated in ice-water bath (0 °C). To the chilled starting material was added a solution 48% hydrofluoric acid in acetonitrile (11:89, 5.0 mL). The resulting acidified solution was stored at 4 °C for 48 h and quenched via addition of sodium sulfate at 0 °C. The reaction mixture was extracted with ethyl acetate (4 x 15 mL) and the combined organics were dried over anhydrous sodium sulfate. Filtration and concentration under vacuum gave the crude product residue that was purified by flash column chromatography (5% MeOH/dichloromethane) providing the desired alcohol as a cloudy, colorless oil (8.1 mg,

0.0271 mmol, 69%). R_f = 0.28 (5% MeOH/dichloromethane); $[\alpha]^{22}_{D}$= 37.5 (*c* 0.52, $CHCl_3$); ^{1}H NMR (400 MHz) δ 7.26 (d, *J* = 15.5 Hz, 1H), 6.12 (d, *J* = 15.5 Hz, 1H), 5.94 (s, 1H), 5.87 (d, *J* = 10.1 Hz, 1H), 3.47 (q, *J* = 6.0 Hz, 2H), 3.39 (ddd, *J* = 9.2, 6.5, 3.4 Hz, 1H), 3.11 (t, *J* = 6.3 Hz, 2H), 2.63 (dt, *J* = 10.1, 6.6 Hz, 1H), 1.96 (s, 3H), 1.81 (d, *J* = 0.9 Hz, 3H), 1.61–1.49 (m, 1H), 1.41–1.27 (m, 1H), 1.06 (d, *J* = 6.7 Hz, 3H), 0.95 (t, *J* = 7.4 Hz, 3H); ^{13}C NMR ($CDCl_3$, 100 MHz) δ 190.7, 170.4, 146.8, 146.6, 132.6, 123.1, 77.0, 40.1, 39.6, 28.5, 27.9, 23.4, 15.8, 12.6, 10.4; HRMS (ESI-TOF) *m/z*: $[M + Na]^+$ Calcd for $C_{15}H_{25}NO_3SSiNa$ 332.1447, Found 322.1447. Upon literature analysis we discovered that this compound had been synthesized through a different route by Cane, DE and co-workers.[5] Our analytical data match that of the original report in all respects.

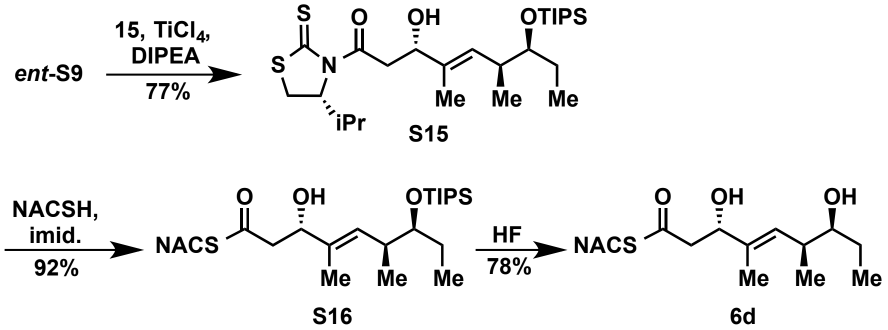

Scheme S5. Chemical preparation of substrate **6d**. The enantiomer of aldehyde **S9** (*ent*-**S9**) was submitted to an aldol reaction with Nagao's acyl chiral auxiliary **15** to afford aldol adduct **S15** as the only product. Interestingly, by decreasing the reaction time from 4 h to 1 h 45 min, we avoided the formation of elimination product with this diastereomer. Thiazolidinethione displacement followed by deprotection furnished the TylDH3 substrate **6d**.

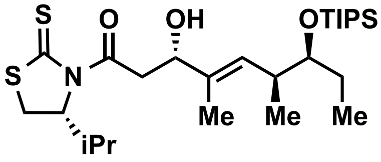

(3*S*,6*S*,7*S*,*E*)-3-hydroxy-1-((*R*)-4-isopropyl-2-thioxo-thiazolidin-3-yl)-4,6-dimethyl-7-((triisopropylsilyl)-oxy)non-4-en-1-one (S15). To a reaction flask containing acyl thiazolidinethione **15** (88.8 mg, 0.437 mmol, 1.70 equiv.) under argon atmosphere was added anhydrous dichloromethane (2.50 mL). The yellow solution was placed in dry ice-acetonitrile bath (- 40 °C) and allowed to equilibrate. To the chilled solution was added titanium(IV) chloride (50.7 μL, 0.463 mmol, 1.80 equiv.) and the resulting orange solution was stirred for 30 min. An aliquot of freshly distilled diisopropylethylamine[1] (80.6 μL, 0.463 mmol, 1.80 equiv.) was slowly added to the reaction mixture and the blood red solution was stirred for an additional 2 h at - 40 °C. The reaction was transferred to a dry ice-acetone bath (-78 °C) and supplemented with a dichloromethane solution (0.400 mL) of aldehyde *ent*-**S9** (80.3 mg, 0.257 mmol, 1.00 equiv.) in a dropwise fashion. The resulting reaction mixture was stirred at -78 °C for 1 h 45 min and quenched upon addition of aqueous saturated ammonium chloride (7.00 mL). The biphasic mixture was warmed to ambient temperature and separated. The aqueous layer was extracted with dichloromethane (3 x 15 mL) and the combined organic layers were dried over anhydrous sodium sulfate, filtered and concentrated under vacuum. The crude product residue was purified by flash column chromatography (20% EtOAc/hexanes) affording the aldol

adduct **S15** (0.102 g, 0.198 mmol, 77%) as a bright yellow oil. R_f = 0.38 (20% EtOAc/hexanes); $[\alpha]_D^{22}$= -199.5 (*c* 1.00, $CHCl_3$); ^{1}H NMR (400 MHz) δ 5.50 (d, *J* = 9.6 Hz, 1H), 5.15 (d, *J* = 6.8 Hz, 1H), 4.55 (dd, *J* = 9.4, 2.2 Hz, 1H), 3.68 (q, *J* = 4.8 Hz, 1H), 3.57–3.51 (m, 1H), 3.51–3.47 (m, 1H), 3.41 (dd, *J* = 17.4, 9.5 Hz, 1H), 3.03 (d, *J* = 11.5 Hz, 1H), 2.58–2.48 (m, 1H), 2.39 (app. sextet, *J* = 6.8 Hz, 1H), 1.67 (s, 3H), 1.61–1.49 (m, 2H), 1.10–1.03 (m, 24H), 0.99 (d, *J* = 6.9 Hz, 3H), 0.94 (d, *J* = 6.8 Hz, 3H), 0.86 (t, *J* = 7.4 Hz, 3H); ^{13}C NMR ($CDCl_3$, 100 MHz) δ 203.1, 173.0, 134.1, 131.1, 77.3, 73.2, 71.7, 44.3, 35.9, 31.0, 30.8, 27.9, 19.3, 18.4, 17.9, 15.1, 13.2, 12.7, 9.4; HRMS (ESI-TOF) *m/z*: $[M + Na]^+$ Calcd for $C_{26}H_{49}NO_3S_2SiNa$ 538.2815, Found 538.2830.

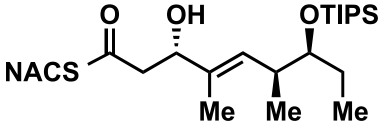

***S*-(2-acetamidoethyl) (3*S*,6*S*,7*S*,*E*)-3-hydroxy-4,6-dimethyl-7-((triisopropylsilyl)oxy)non-4-enethioate (S16).** To a round bottom flask containing aldol adduct **S15** (42.0 mg, 0.0814 mmol) dissolved in anhydrous dichloromethane (3 mL) under argon atmosphere was added imidazole (17.0 mg, 0.250, 3.07 equiv.). To the stirred reaction mixture was added *N*-acetylcysteamine (9.50 µL, 0.0893 mmol, 1.10 equiv.) and the resulting solution was stirred at ambient temperature for 7 h. The reaction mixture was quenched upon addition of a saturated, aqueous ammonium chloride solution (5 mL) and the biphasic solution was separated. The aqueous layer was extracted with dichloromethane (4 x 15 mL) and the combined organic layers were dried over anhydrous sodium sulfate, filtered and concentrated under vacuum. Crude product residues were purified by flash column chromatography (5% MeOH/dichloromethane) providing the title compound as a clear, colorless oil (35.3 mg, 0.746 mmol, 92%). R_f = 0.23 (5% MeOH/dichloromethane); $[\alpha]_D^{22}$= -9.4 (*c* 1.00, $CHCl_3$); ^{1}H NMR (400 MHz) δ 5.85 (br, 1H), 5.48 (d, *J* = 9.6 Hz, 1H), 4.47 (dd, *J* = 9.0, 3.5 Hz, 1H), 3.76–3.60 (m, 1H), 3.45 (q, *J* = 6.2 Hz, 2H), 3.05 (dt, *J* = 5.3, 3.0 Hz, 2H), 2.82 (dd, *J* = 15.0, 9.0 Hz, 1H), 2.73 (dd, *J* = 15.0, 3.7 Hz, 1H), 2.58–2.44 (m, 1H), 1.97 (s, 3H), 1.65 (s, 3H), 1.60–1.46 (m, 2H), 1.06 (s, 21H), 0.92 (d, *J* = 6.8 Hz, 3H), 0.85 (t, *J* = 7.4 Hz, 3H); ^{13}C NMR ($CDCl_3$, 100 MHz) δ 199.1, 170.5, 133.9, 131.4, 77.4, 74.3, 49.8, 39.6, 35.9, 29.0, 27.8, 23.4, 18.4, 15.1, 13.2, 12.2, 9.3; HRMS (ESI-TOF) *m/z*: $[M + Na]^+$ Calcd for $C_{24}H_{47}NO_4SSiNa$ 496.2887, Found 496.2881.

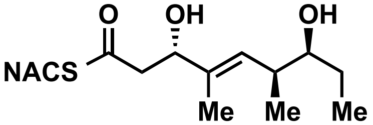

***S*-(2-acetamidoethyl) (3*S*,6*S*,7*S*,*E*)-3,7-dihydroxy-4,6-dimethylnon-4-enethioate (6d).** A large conical polypropylene tube (50 mL, BD Falcon™) containing silylether **S16** (35.3 mg, 0.0746 mmol) dissolved in acetonitrile (3.75 mL) was placed in ice-water (0 °C) bath and allowed to equilibrate. To the chilled reaction vessel was added a solution of 48% hydrofluoric acid diluted in acetonitrile (11:89, 5.52 mL). The resulting clear solution was stored at 4 °C for 23 h and neutralized via addition of an aqueous saturated sodium bicarbonate solution at 0 °C. The reaction mixture was extracted with ethyl acetate (4 x 15 mL) and the combined organic fractions were dried over anhydrous sodium sulfate, filtered and concentrated under vacuum. The crude residue was purified via flash column chromatography (10% MeOH/dichloromethane) yielding the alcohol as a colorless oil (18.5 mg, 0.0583 mmol, 78%). R_f = 0.38 (10% MeOH/dichloromethane); $[\alpha]_D^{23}$= -25.0 (*c* 0.62, $CHCl_3$); ^{1}H NMR (400 MHz) δ 6.19 (br, 1H), 5.36 (d, *J* = 9.7 Hz, 1H), 4.46 (dd, *J* = 8.2, 3.5 Hz, 1H), 3.42

(q, J = 6.1 Hz, 2H), 3.38–3.29 (m, 1H), 3.09–2.95 (m, 2H), 2.83 (dd, J = 14.8, 8.6 Hz, 1H), 2.73 (dd, J = 14.8, 3.8 Hz, 1H), 2.48 (dq, J = 13.3, 6.6 Hz, 1H), 1.95 (s, 3H), 1.65 (s, 3H), 1.58–1.47 (m, 1H), 1.32 (dq, J = 15.1, 7.8 Hz, 1H), 0.94 (d, J = 6.9 Hz, 3H), 0.93 (t, J = 7.2 Hz, 3H); ^{13}C NMR ($CDCl_3$, 100 MHz) δ 198.8, 170.8, 135.8, 129.7, 77.3, 74.2, 49.6, 39.4, 37.8, 29.0, 26.9, 23.3, 15.8, 12.5, 10.7; HRMS (ESI-TOF) *m/z*: $[M + Na]^+$ Calcd for $C_{15}H_{27}NO_4SNa$ 340.1553, Found 340.1556.

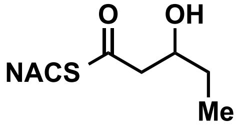

(±)-*S*-(2-acetamidoethyl) 3-hydroxypentanethioate ((±)-26). The details for the synthesis of racemic diketide substrate **26** are currently pending publication in a separate manuscript.[6]

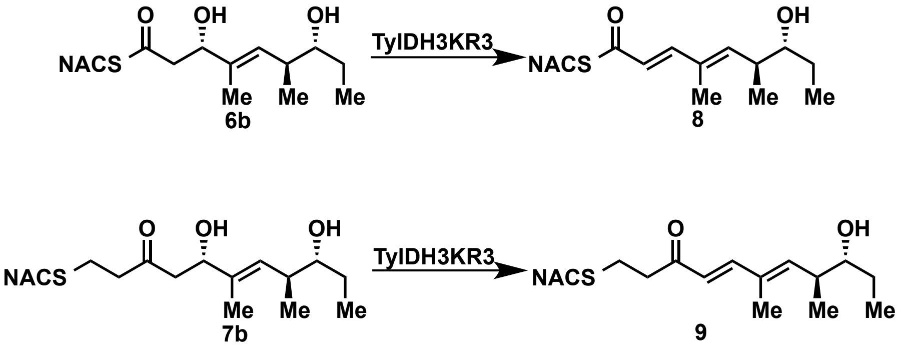

Scheme S6. The chemoenzymatic synthesis of of dienes **8** and **9** via TylDH3KR3-catalyzed dehydration.

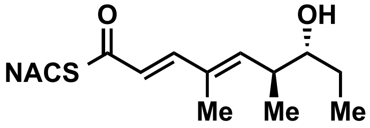

***S*-(2-acetamidoethyl) (2*E*,4*E*,6*S*,7*R*)-7-hydroxy-4,6-dimethylnona-2,4-dienethioate (8).** To a small polypropylene conical tube (15 mL) containing sterile deionized water (0.630 mL) was added a concentrated Tris buffer solution (0.500 M Tris-HCl, 1.50 M NaCl, pH 8.0, 0.500 mL). The clear solution was vortexed to mix and β-hydroxy thioester **6b** (3.17 mg, 0.0100 mmol) was added as a 50:50 DMSO-water stock solution (100 mM, 100 μL) and mixed by inversion. Heterologously expressed TylDH3KR3 (2.53 mg/mL stock solution, 1.52 mL, 3.85 mg, 50.3 nmol). The reaction mixture was capped and incubated at ambient temperature with shaking (250 rpm) for 26 h. The aqueous solution was extracted with ethyl acetate (3 x 10 mL) followed by drying over anhydrous sodium sulfate, filtration and concentration under vacuum. The crude product residue was purified via flash column chromatography (5% MeOH/dichloromethane) affording a cloudy, colorless oil (2.00 mg, 0.00667 mmol, 67%). R_f = 0.32 (5% MeOH/dichloromethane); Due to stability issues, an optical rotation was not obtained; ^{1}H NMR (400 MHz, $CDCl_3$) δ 7.29 (d, J = 15.6 Hz, 1H), 6.12 (d, J = 15.5 Hz, 1H), 5.96 (d, J = 10.1 Hz, 1H), 5.91 (br, 1H), 3.52–3.39 (m, 3H), 3.12 (t, J = 6.3 Hz, 2H), 2.72–2.60 (m, 1H), 1.96 (s, 3H), 1.82 (s, 3H), 1.54–1.49 (m, 1H), 1.45–1.34 (m, 1H), 1.05 (d, J = 6.8 Hz, 3H), 0.96 (t, J = 7.4 Hz, 3H); ^{13}C NMR ($CDCl_3$, 100 MHz) δ 190.7, 170.4, 146.6, 146.0, 133.5, 123.1, 77.0, 40.2, 39.2, 28.5, 27.8, 23.4, 17.0, 12.7, 10.2; HRMS (ESI-TOF) *m/z*: $[M + Na]^+$ Calcd for $C_{15}H_{25}NO_3SNa$ 322.1447, Found 322.1451.

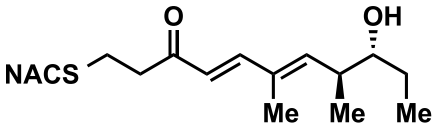

N-(2-(((4E,6E,8S,9R)-9-hydroxy-6,8-dimethyl-3-oxoundeca-4,6-dien-1-yl)thio)ethyl)acetamide (9). β-Hydroxy ketone **7b** (3.45 mg, 0.0100 mmol) was dehydrated in an analogous matter as the generation of dienethioate **8** resulting in dienone **9** (3.17 mg, 0.00969 mmol, 97%). R_f = 0.30 (5% MeOH/dichloromethane); Due to stability issues, an optical rotation was not obtained; ^{1}H NMR (400 MHz) δ 7.17 (d, J = 16.7 Hz, 1H), 6.05 (d, J = 15.9 Hz, 1H), 5.89 (d, J = 10.0 Hz, 1H), 3.43–3.35 (m, 3H), 2.87–2.80 (m, 2H), 2.80–2.74 (m, 3H), 2.66 – 2.54 (m, 3H), 1.94 (s, 3H), 1.76 (s, 3H), 1.52–1.42 (m, 1H), 1.40–1.28 (m, 1H), 0.99 (d, J = 6.8 Hz, 3H), 0.90 (t, J = 7.4 Hz, 3H); ^{13}C NMR ($CDCl_3$, 100 MHz) δ 198.8, 170.4, 148.7, 145.5, 133.9, 124.6, 77.0, 40.2, 39.2, 38.7, 32.3, 27.8, 26.1, 23.4, 17.1, 12.7, 10.2; HRMS (ESI-TOF) m/z: $[M + Na]^+$ Calcd for $C_{17}H_{29}NO_3SNa$ 350.1760, Found 350.1762.

General biology procedures. All chemical reagents were purchased from Sigma-Aldrich and were used directly without further purification. *E. coli* BL21–AI cells were from Life Technologies. IPTG was acquired through Gold Biotechnology. L-(+)-Arabinose (≥99%) was purchased through Sigma Aldrich. His60 Ni Superflow resin was purchased from Clontech Laboratories, Inc. OD_{600} was measured on an Eppendorf BioPhotometer. Sonication was carried out by Branson Sonifier 450. Gel filtration purification was performed on HiLoad 16/600 Superdex 200 pg column (GE). Protein mass spectrometry was carried out using an Agilent 6250 QTOF LC/MS. Kinetic LC–MS/MS was conducted with AB Sciex QTRAP 5500 mass spectrometer and Shimadzu LC system.

The TylKR3-DH3 didomain was ordered as codon-optimized synthetic DNA from Life Technologies. The synthetic DNA encoded the region 957-1682 from the tylactone synthase module 3 polypeptide. The insert was cloned into pMCSG7 using ligation independent cloning (LIC). TylKr3-DH3 synthetic forward primer: 5’-**TACTTCCAATCCAATGCCC**ATCCGCTGCTGAGCG-3’; TylKr3-DH3 synthetic reverse primer: 5’-**TTATCCACTTCCAATG**<u>**TTA**</u>GTTGGTATCTTCCGGTGTACCAGGCG-3’ (LIC-overhangs in **bold**; inserted stop codon <u>underlined</u>). The insert was confirmed via sequencing.

Cloning and Expression of TylDH3-KR3 Construct

Initial efforts to recombinantly express the mono-domain TylKR3 were hampered by with poor expression levels and protein aggregation. Strategies to alleviate these issues included an increase of rare tRNA codons (Rosetta cell line), optimization of codon selection (synthetic TylKR3 gene), toxic protein-compatible expression hosts (pLysS cell line), appending a fusion protein (attempted with SUMO, mOCR, and GST), chaperone coexpression (GroEL-GroES) and truncations of both N- and C-termini. Disappointingly, these techniques failed to improve expression of soluble non-aggregated TylKR3 and forced us to abandon the expression of the mono-domain construct.

The *tylGII* region encoding TylDH3-KR3 didomain comprising residues 957-1682 was cloned into a pMCSG7 vector and transformed into *E. coli* BL21-AI cells containing the pRARE plasmid. A large TB media culture (0.5 L in 2.8 L Fernbach flask) was

inoculated with as small amount of overnight culture (5 mL) and incubated at 37 °C, shaking at 250 RPM until OD600 = 1.00–1.20. The culture was cooled to 20 °C and incubated with shaking (250 RPM) for 1 h. Cells were induced upon addition of IPTG (0.100 mM) and L-arabinose (1.00 g) and allowed to shake (250 RPM) at 20 °C for 19 h. The cell pellet was collected after centrifugation (4 °C, 5,000 x G, 30 min) and resuspended in lysis buffer (50 mM tricine, 50 mM ammonium sulfate, 100 mM urea, pH 8.5, 4 mL/g of pellet). The cells were lysed (3 x 2 min, 50% duty cycle, 40 % power, 4 °C) and centrifuged (4 °C, 28,600 x G, 45 min). The soluble protein was purified by sequential metal-immobilized affinity chromatography and size exclusion chromatography to afford approximately 9 mg of purified protein (18 mg / L) that was greater than 90% pure as judged by SDS-PAGE (**Figure S1**) and to be near the predicted calculated mass by mass spectrometry (**Figure S2**).

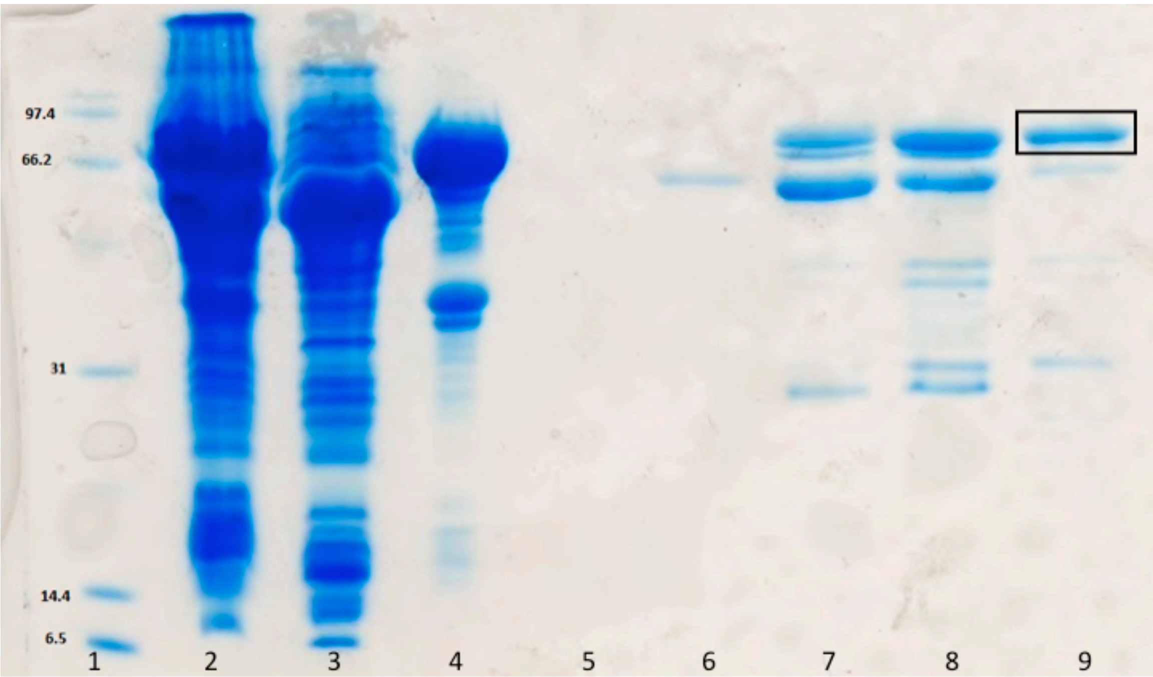

Figure S1. SDS-PAGE image of TylDH3-KR3 purification. The ladder, cell lysate, insoluble pellet, soluble protein and serial nickel elution fractions are shown in lanes 1-9, respectively. The band corresponding to TylDH3-KR3 is boxed.

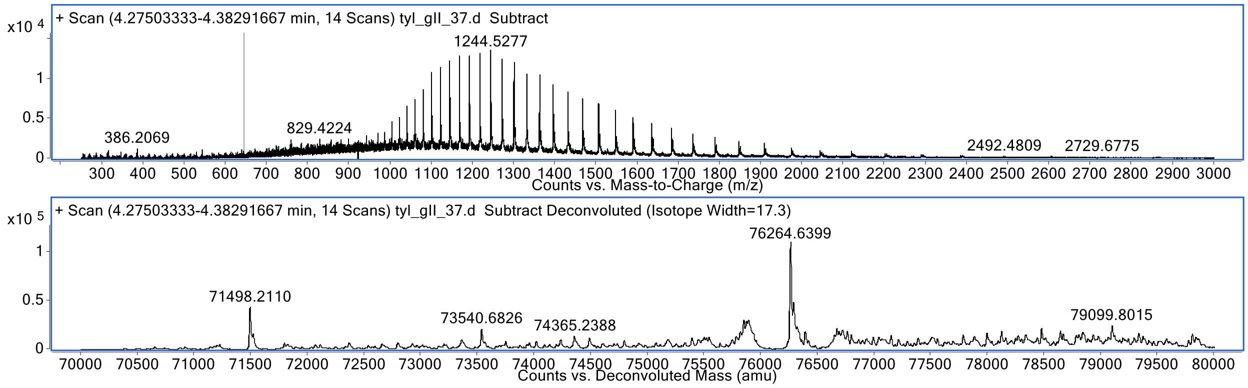

Figure S2- Mass spectrometry analysis of TylDH3-KR3. The convoluted (raw) spectrum and deconvoluted are both displayed. TylDH3-KR3 was found to have a mass of 76,264 Da. The spectrum was obtained using 0.5 mg/mL of recombinant TylDH3-KR3 in 25% formic acid.

Determination of TylDH3-KR3 Ketoreductase Activity

A small eppendorf tube (1.5 mL, 100 μL total volume) containing substrate **4** or **5** (1 mM), heterologously expressed TylDH3-KR3 (5 μM), NADPH (2 mM), Tris (50 mM), NaCl (150 mM) at pH 8.0 was incubated at ambient temperature for 15 h. 60 μL of the diluted reaction solution was added to a HPLC vial and analyzed by LC-MS/MS (Table S4) employing a Kinetix reverse-phase C_{18} column (50 mm × 2.1 mm, 2.6 μm, Phenomenex) operated at 0.4 mL min^{-1} with a gradient between mobile phase A (H_2O) and mobile phase B (MeCN). The gradient program was 0 min, 5% B; 2 min, 5% B; 7 min, 55% B; 8 min, 70% B; 9 min, 70% B; 10.5 min, 5% B; 12 min, 5% B. Co-injection of standards **6a**, **6b**, and **8** for substrate **4** confirmed the identity of product traces. The standards **7a**, **7b**, and **9** were used for the analysis of the incubation of substrate **5**.

Table S1. LC-MS/MS analysis of analytes **6a**, **6b**, **8**, **7a**, **7b**, and **9**.

Analyte	HPLC retention time (min)	Transition
**6a**	5.19	340→184
**6b**	5.27	340→184
**8**	6.17	300→181
**7a**	5.24	368→212
**7b**	5.30	368→212
**9**	6.00	328→151

Analysis of TylDH3-KR3 Dehydratase Activity

Steady-State Analysis.

The enzymatic reactions were carried out in a total volume of 50 μL under initial velocity conditions containing TylDH3-KR3 (1 μM), reaction buffer (50 mM Tris, 150 mM NaCl,

pH 8.0) and substrates **6b** or **7b** at variable concentrations (0.5, 1, 2, 3, 4, 6, 8 mM). The final DMSO concentration was held constant at 4%. After incubation at 25 °C for 8 min (the reaction found to be linear up to 10 min), 5 μL of the reaction mixture was added to 495 μL of 1:1 MeCN–reaction buffer (100-fold dilution). The resulting solution was vortexed, centrifuged and analyzed by 60 μL of the diluted reaction solution was added to a HPLC vial with 10 μL of internal standard **3** (320 nM) and analyzed by LC-MS/MS (Table S4) employing a Kinetix reverse-phase C_{18} column (50 mm × 2.1 mm, 2.6 μm, Phenomenex) operated at 0.4 mL min^{-1} with a gradient between mobile phase A (H_2O) and mobile phase B (MeCN). The gradient program was 0 min, 5% B; 2 min, 5% B; 7 min, 55% B; 8 min, 70% B; 9 min, 70% B; 10.5 min, 5% B; 12 min, 5% B. Standard curves of enzymatic products **8** and **9** were generated by injecting the authentic standard at varying concentrations with a fixed concentration of an internal standard **(8** for the standard curve of **9** and **9** for the standard curve of **8)**. The amount of enzymatic product formation at each time point was calculated by plotting the area ratio (analyte/internal standard) into the standard curve. Control reactions for each concentration of substrate were performed without the addition of enzyme. Each reaction was performed in duplicate. The specificity constants (K_M/k_{cat}) were determined by fitting the normalized v_0 vs [S] plots to linear equations (**Figure S3** panels **A** and **B**).

Substrates **6c** and **6d** were analyzed in an analogous way using synthetic **S14** as the product standard (**Figure S3** panels **C** and **D**). The LC-MS/MS trace of **S14** is provided in **Figure S4**.

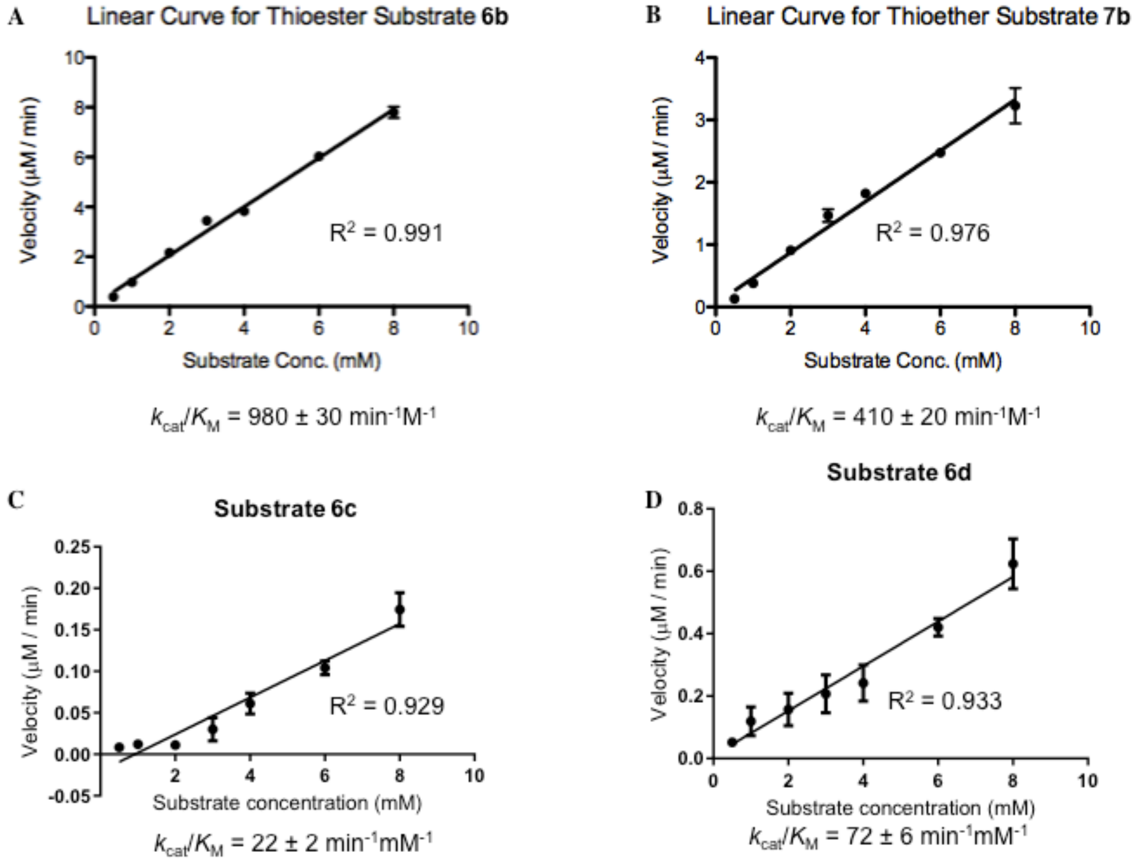

Figure S3. Linear regression analysis of TylDH3KR3 kinetic data with substrates **6b**, **7b**, **6c**, and **6d**. The kinetic plots of thioester **6b**, thioether **7b**, thioester **6c** and thioester **6d** are shown in panels **A**, **B**, **C** and **D**, respectively. Represented data is the result of duplicate LC-MS/MS data normalized with controls lacking enzyme. Specificity constants (k_{cat}/K_M) for each substrate are displayed below the corresponding plot.

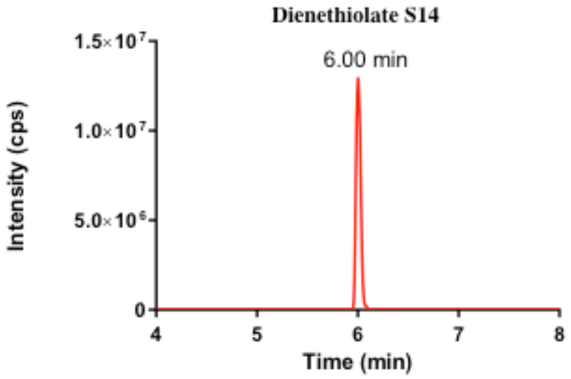

Figure S4. LC-MS/MS trace of dienethiolate **S14**. The synthetic compound **S14** was used to confirm the identity of the dehydration products arising from thioesters **6c** and **6d**. A standard curve for quantitative determination of specificity constants was generated from the synthetic compound.

References:

1. W. L. F. Armarego and D. D. Perrin, *Purification of laboratory chemicals*, Butterworth Heinemann, Oxford ; Boston, 4th edn., 1996.
2. S. Shirokawa, M. Shinoyama, I. Ooi, S. Hosokawa, A. Nakazaki and S. Kobayashi, *Org. Lett.*, 2007, **9**, 849-852.
3. Y. Mukaeda, T. Kato and S. Hosokawa, *Org. Lett.*, 2012, **14**, 5298-5301.
4. A. Schmauder, S. Muller and M. E. Maier, *Tetrahedron*, 2008, **64**, 6263-6269.
5. D. E. Cane, W. Tan and W. R. Ott, *J. Am. Chem. Soc.*, 1993, **115**, 527-535.
6. Y. Li, G. J. Dodge, W. D. Fiers, R. A. Fecik, J. L. Smith and C. C. Aldrich, *J. Am. Chem. Soc.*, 2015, DOI: 10.1021/jacs.5b02325.

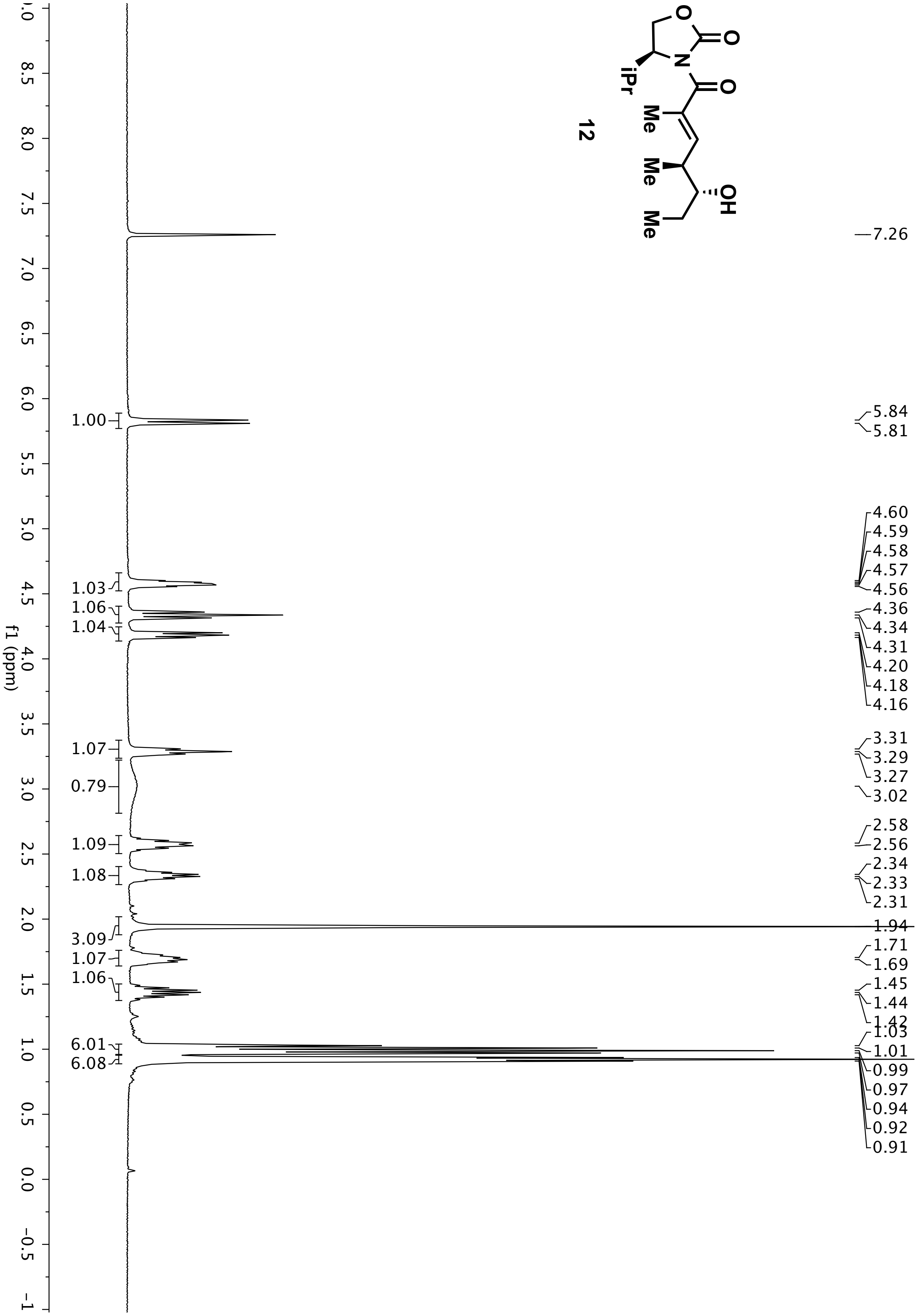

12
iPr
Me
Me
Me
OH
7.26
5.84
5.81
4.60
4.59
4.58
4.57
4.56
4.36
4.34
4.31
4.20
4.18
4.16
3.31
3.29
3.27
3.02
2.58
2.56
2.34
2.33
2.31
1.94
1.71
1.69
1.45
1.44
1.42
1.03
1.01
0.99
0.97
0.94
0.92
0.91
1.00
1.03
1.06
1.04
1.07
0.79
1.09
1.08
3.09
1.07
1.06
6.01
6.08
f1 (ppm)

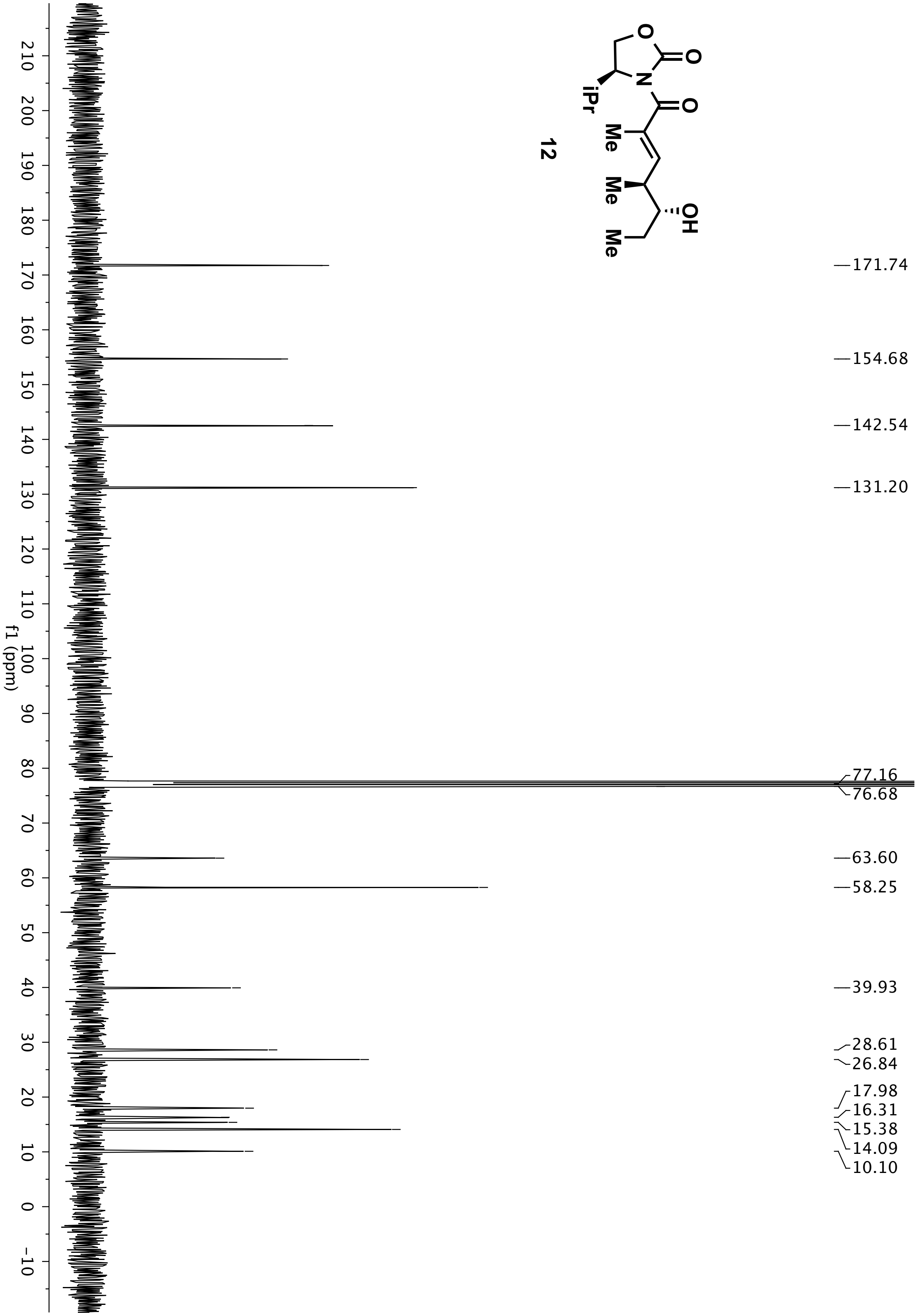
12
171.74
154.68
142.54
131.20
77.16
76.68
63.60
58.25
39.93
28.61
26.84
17.98
16.31
15.38
14.09
10.10
f1 (ppm)

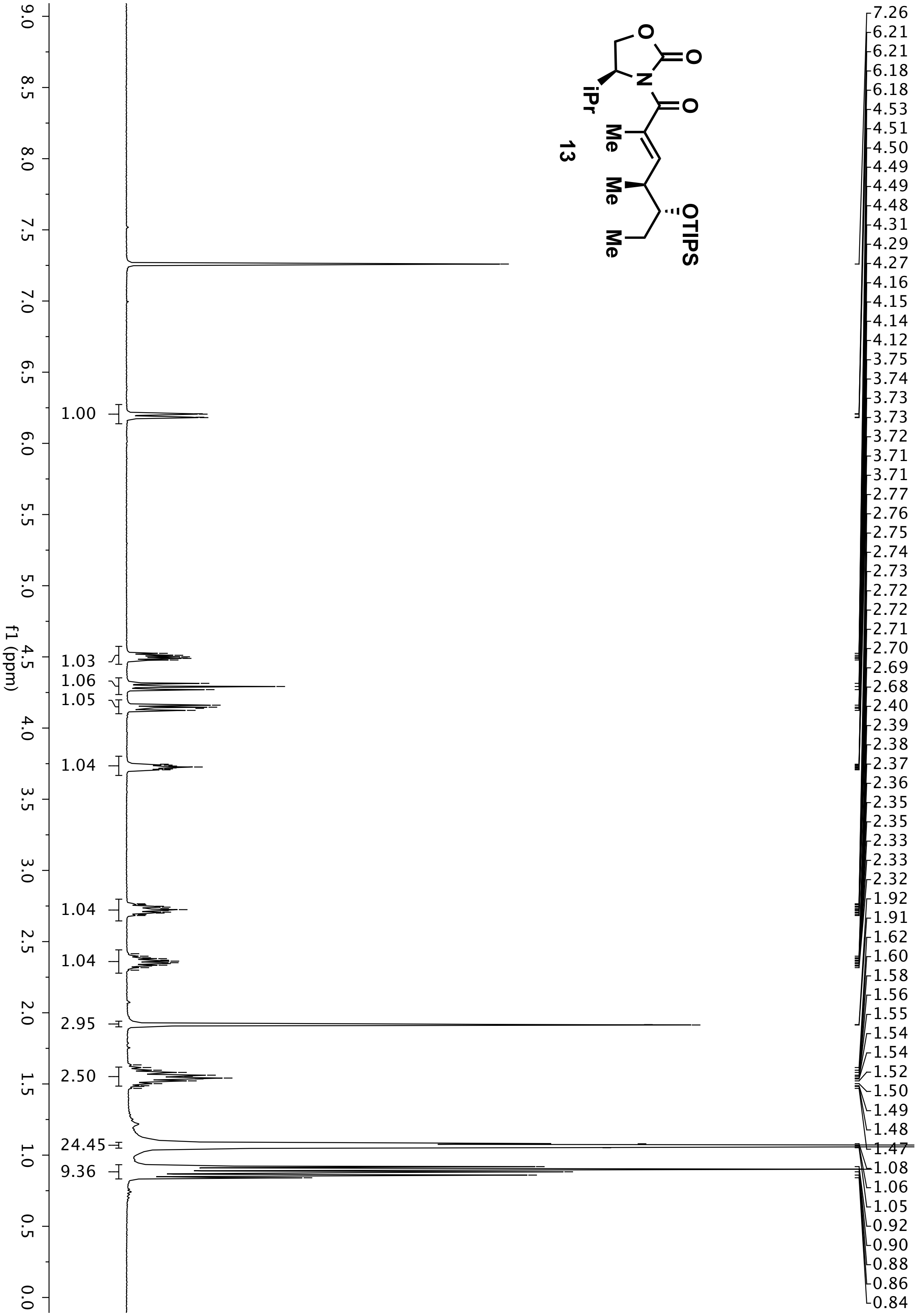
O
N
O
O
iPr
Me
Me
Me
OTIPS
13
7.26
6.21
6.21
6.18
6.18
4.53
4.51
4.50
4.49
4.49
4.48
4.31
4.29
4.27
4.16
4.15
4.14
4.12
3.75
3.74
3.73
3.73
3.72
3.71
3.71
2.77
2.76
2.75
2.74
2.73
2.72
2.72
2.71
2.70
2.69
2.68
2.40
2.39
2.38
2.37
2.36
2.35
2.35
2.33
2.33
2.32
1.92
1.91
1.62
1.60
1.58
1.56
1.55
1.54
1.54
1.52
1.50
1.49
1.48
1.47
1.08
1.06
1.05
0.92
0.90
0.88
0.86
0.84
1.00
1.03
1.06
1.05
1.04
1.04
1.04
2.95
2.50
24.45
9.36
9.0
8.5
8.0
7.5
7.0
6.5
6.0
5.5
5.0
4.5
4.0
3.5
3.0
2.5
2.0
1.5
1.0
0.5
0.0
f1 (ppm)

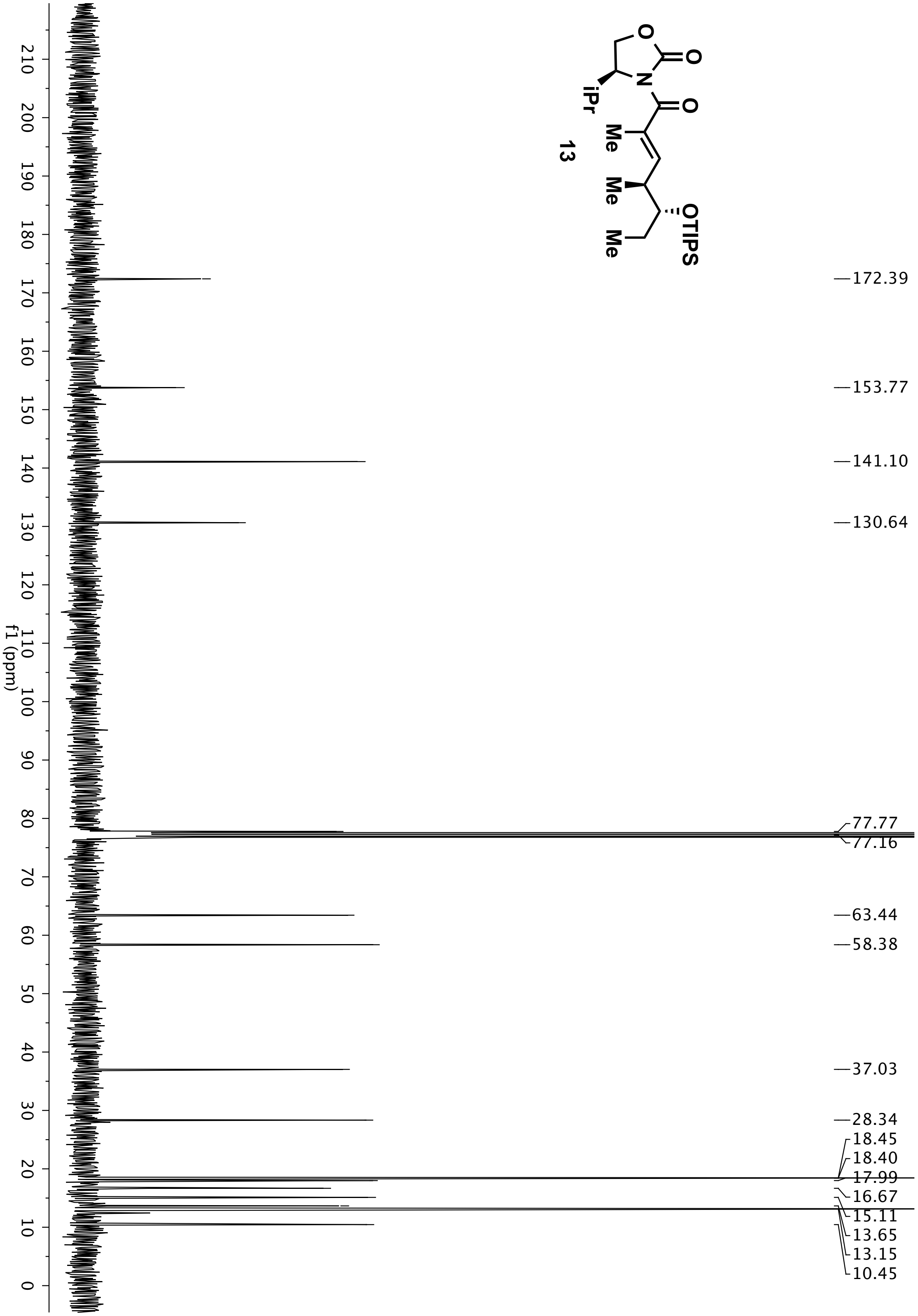
O
O
N
O
iPr
Me
Me
OTIPS
Me
13
172.39
153.77
141.10
130.64
77.77
77.16
63.44
58.38
37.03
28.34
18.45
18.40
17.99
16.67
15.11
13.65
13.15
10.45
f1 (ppm)

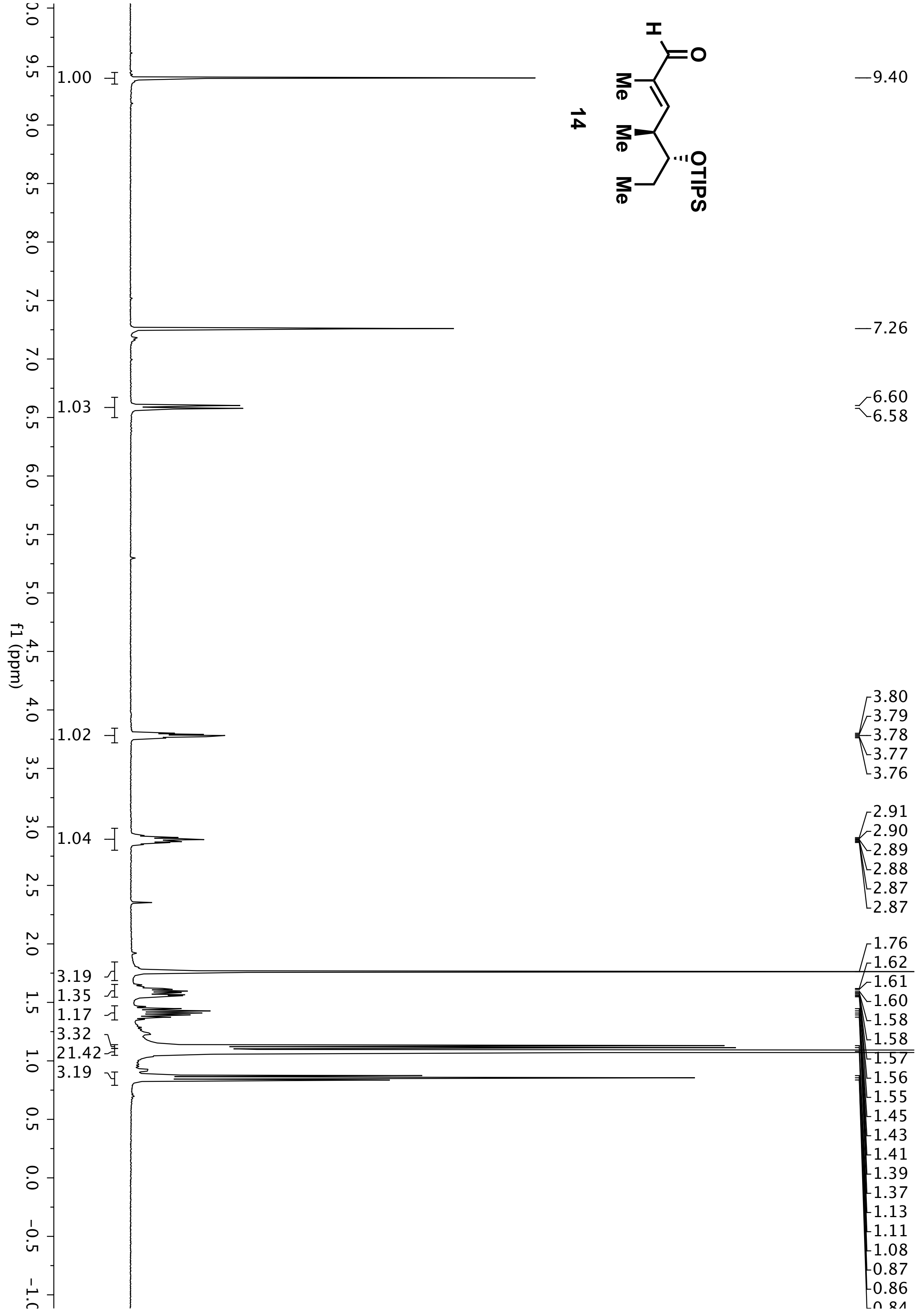
OTIPS
Me
Me
Me
H
O
14
9.40
7.26
6.60
6.58
3.80
3.79
3.78
3.77
3.76
2.91
2.90
2.89
2.88
2.87
2.87
1.76
1.62
1.61
1.60
1.58
1.58
1.57
1.56
1.55
1.45
1.43
1.41
1.39
1.37
1.13
1.11
1.08
0.87
0.86
0.84
1.00
1.03
1.02
1.04
3.19
1.35
1.17
3.32
21.42
3.19
f1 (ppm)

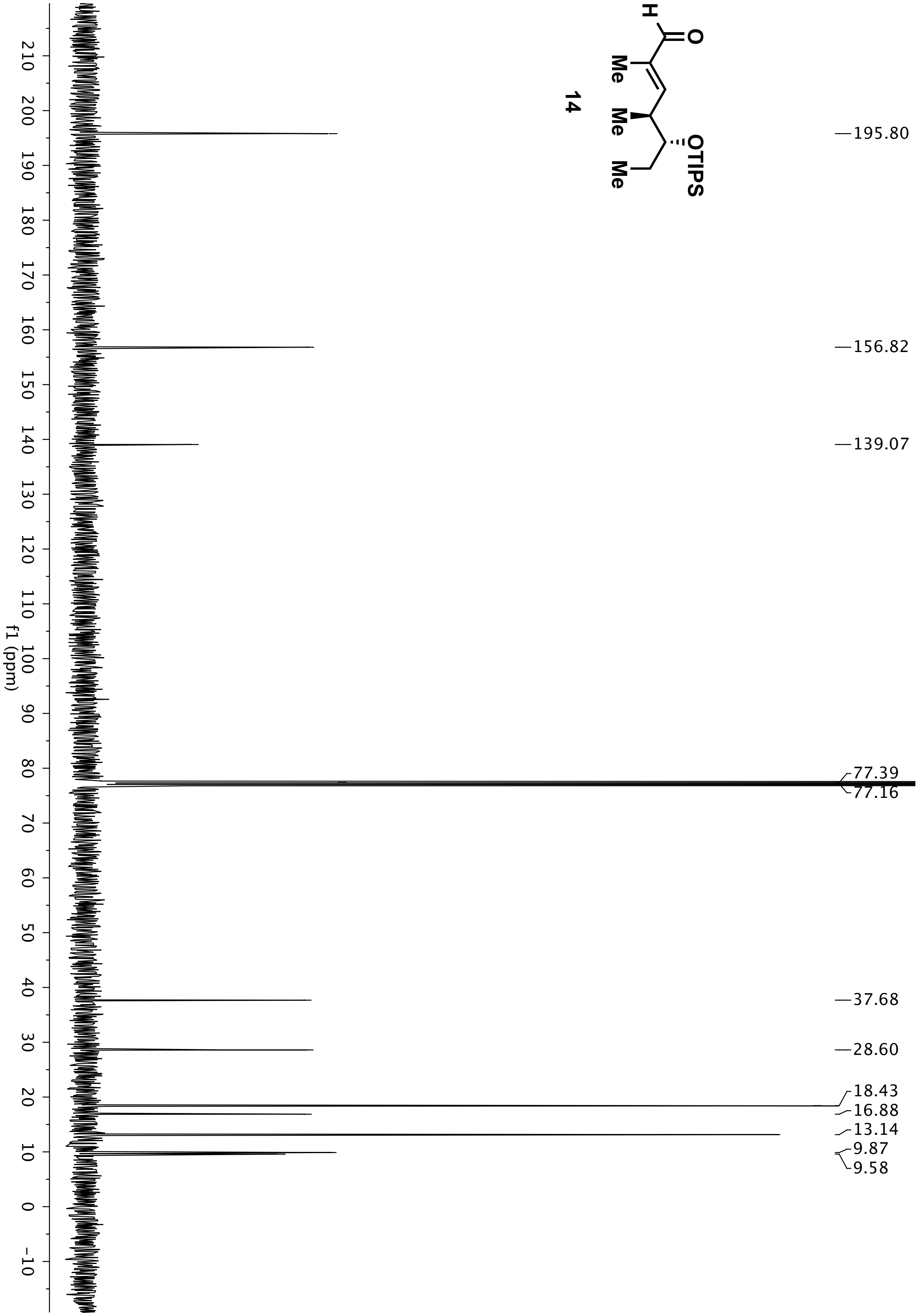

OTIPS
Me
Me
Me
H
O
14
195.80
156.82
139.07
77.39
77.16
37.68
28.60
18.43
16.88
13.14
9.87
9.58
f1 (ppm)

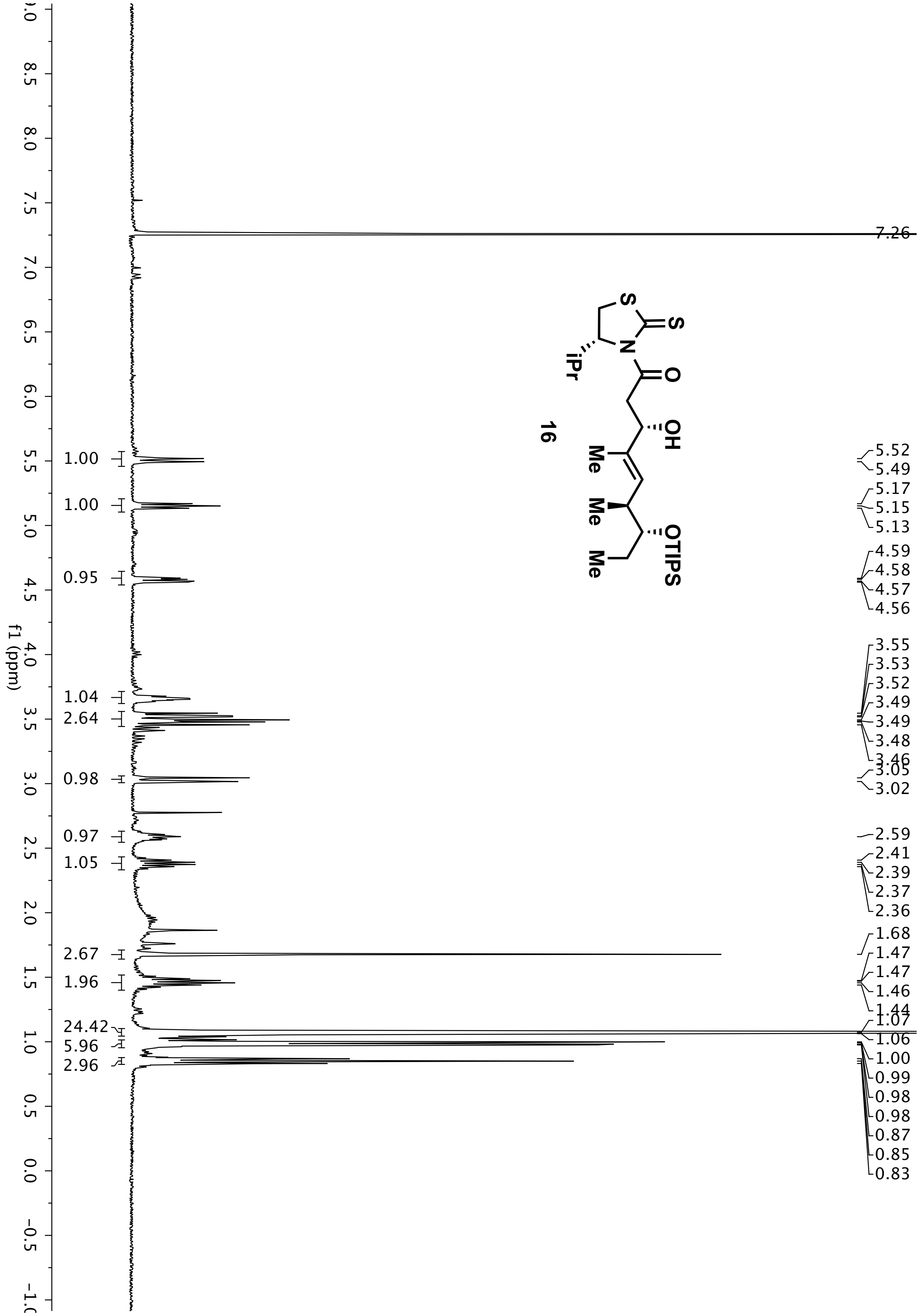
16
f1 (ppm)

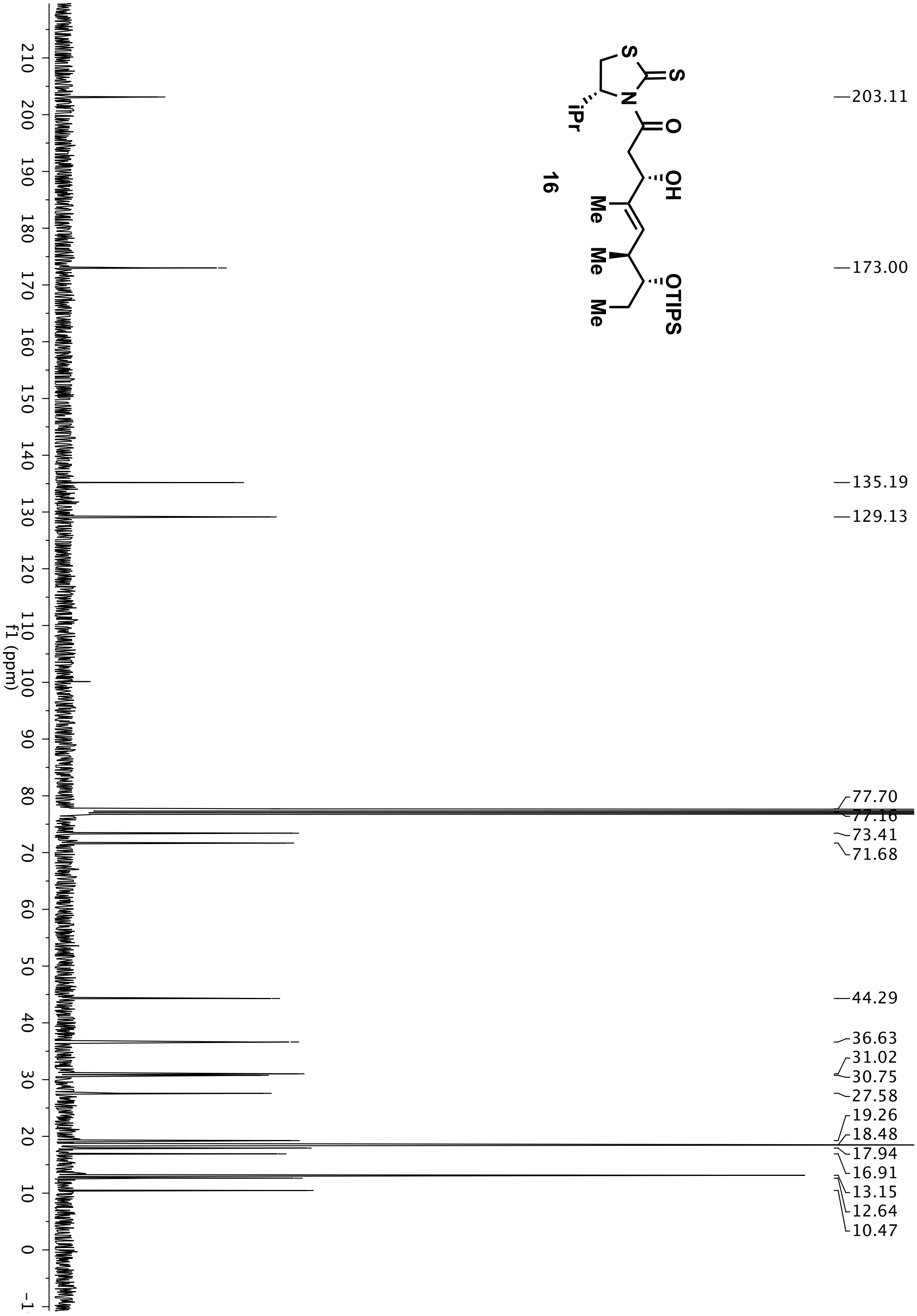

S
N
O
OH
OTIPS
Me
iPr
16
203.11
173.00
135.19
129.13
77.70
77.16
73.41
71.68
44.29
36.63
31.02
30.75
27.58
19.26
18.48
17.94
16.91
13.15
12.64
10.47
f1 (ppm)

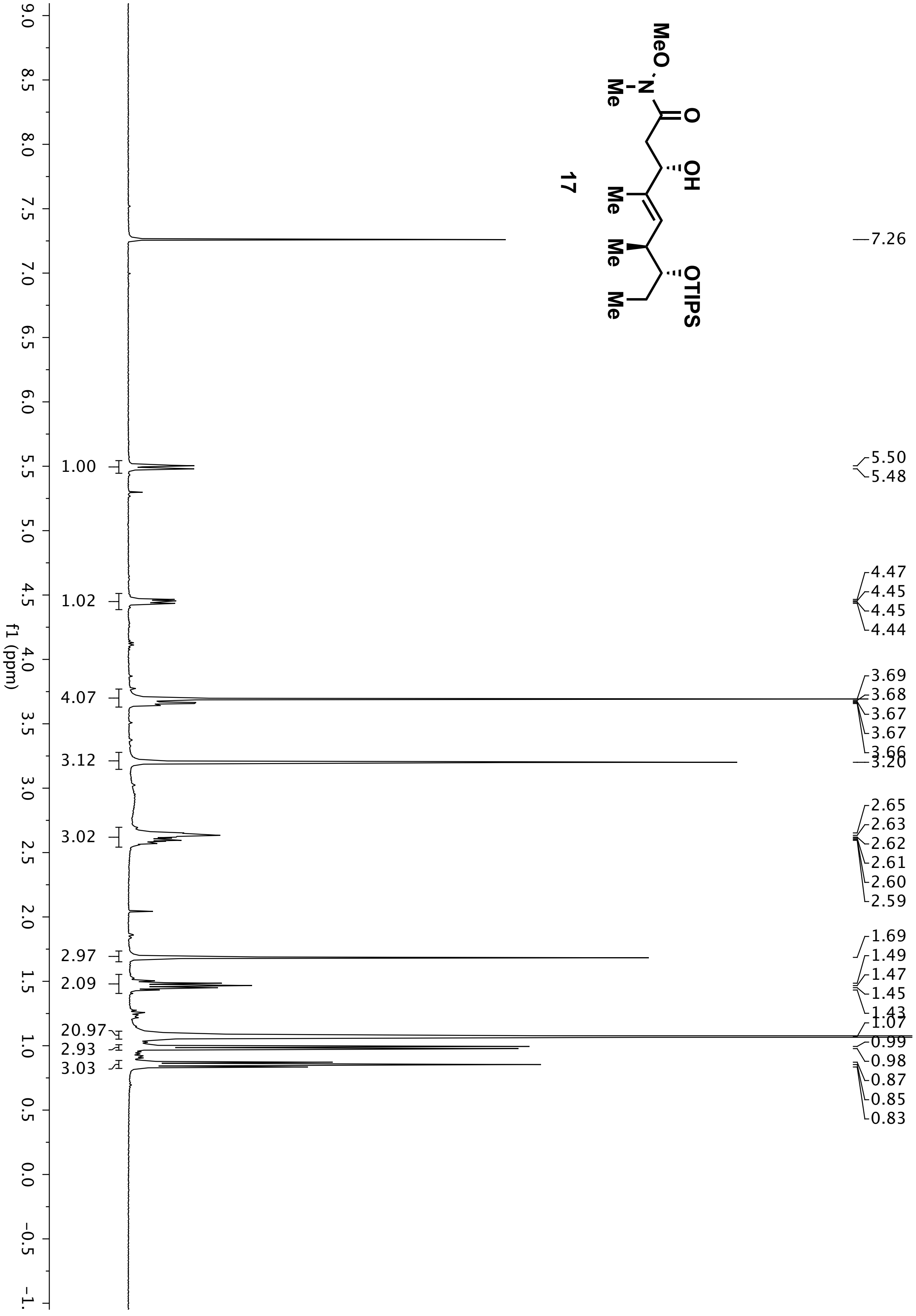
17
f1 (ppm)

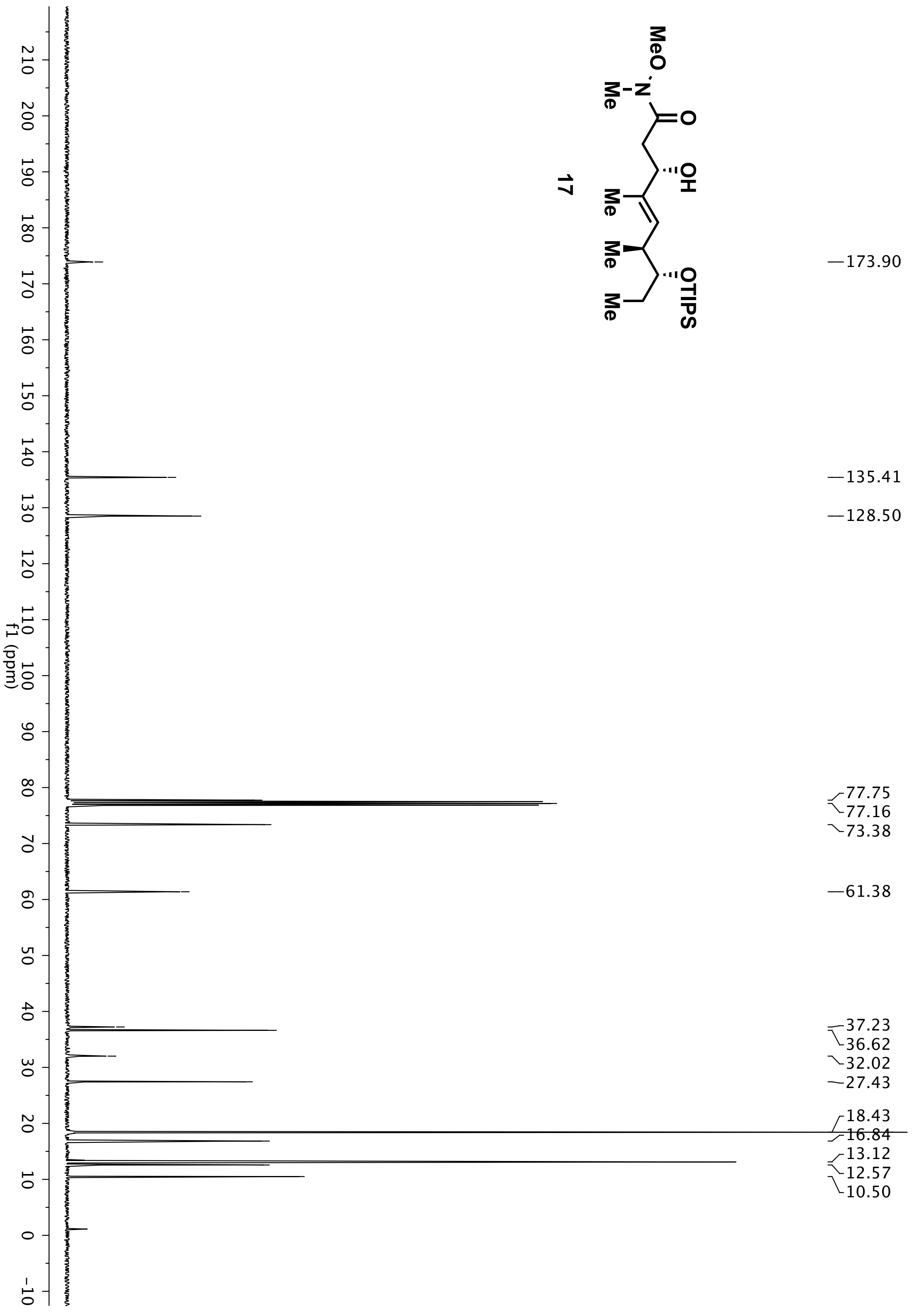
MeO
N
Me
O
OH
Me
Me
OTIPS
Me
17
173.90
135.41
128.50
77.75
77.16
73.38
61.38
37.23
36.62
32.02
27.43
18.43
16.84
13.12
12.57
10.50
f1 (ppm)

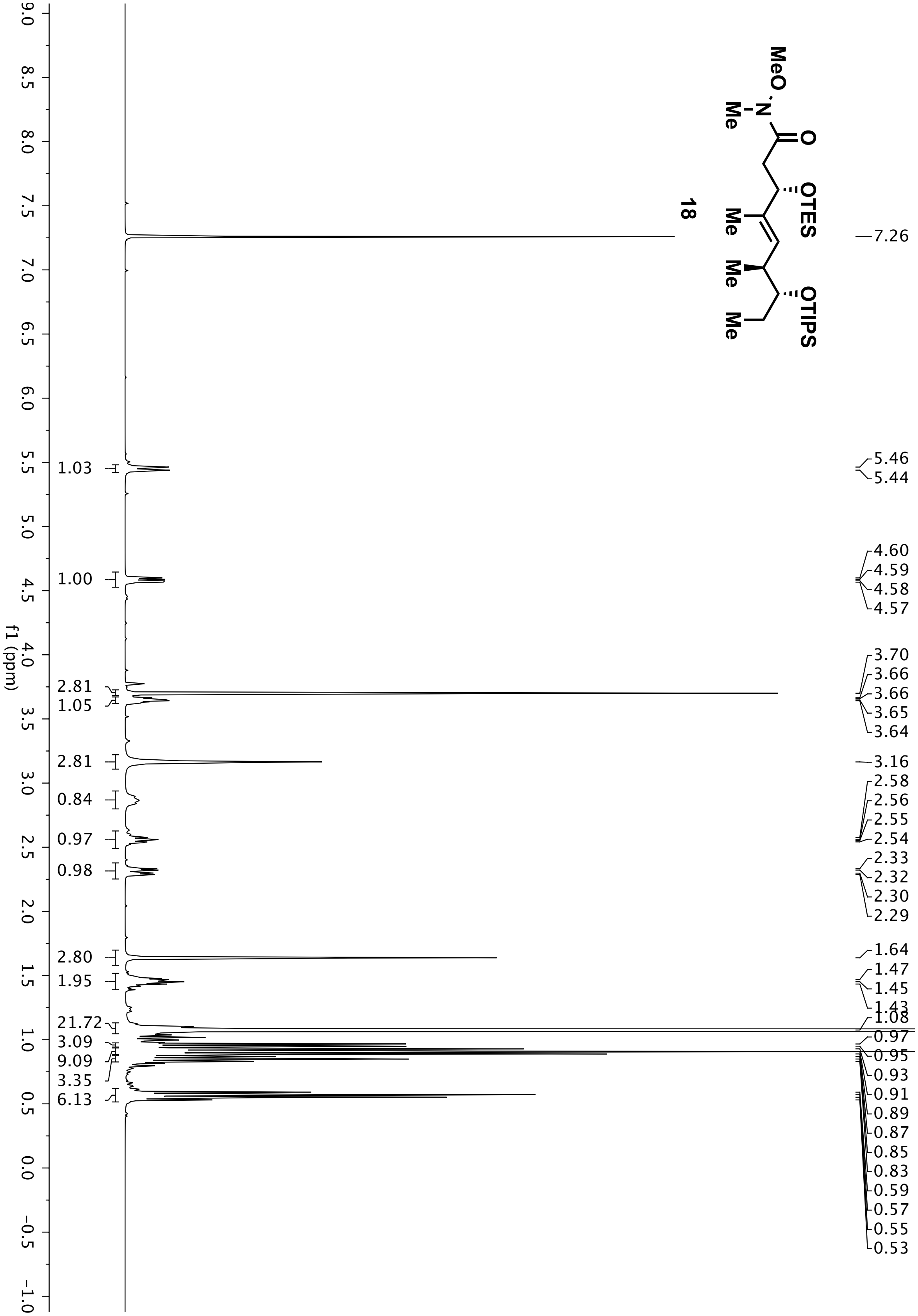

18
OTES
OTIPS
MeO
N
Me
O
f1 (ppm)
7.26
5.46
5.44
4.60
4.59
4.58
4.57
3.70
3.66
3.66
3.65
3.64
3.16
2.58
2.56
2.55
2.54
2.33
2.32
2.30
2.29
1.64
1.47
1.45
1.43
1.08
0.97
0.95
0.93
0.91
0.89
0.87
0.85
0.83
0.59
0.57
0.55
0.53
1.03
1.00
2.81
1.05
2.81
0.84
0.97
0.98
2.80
1.95
21.72
3.09
9.09
3.35
6.13

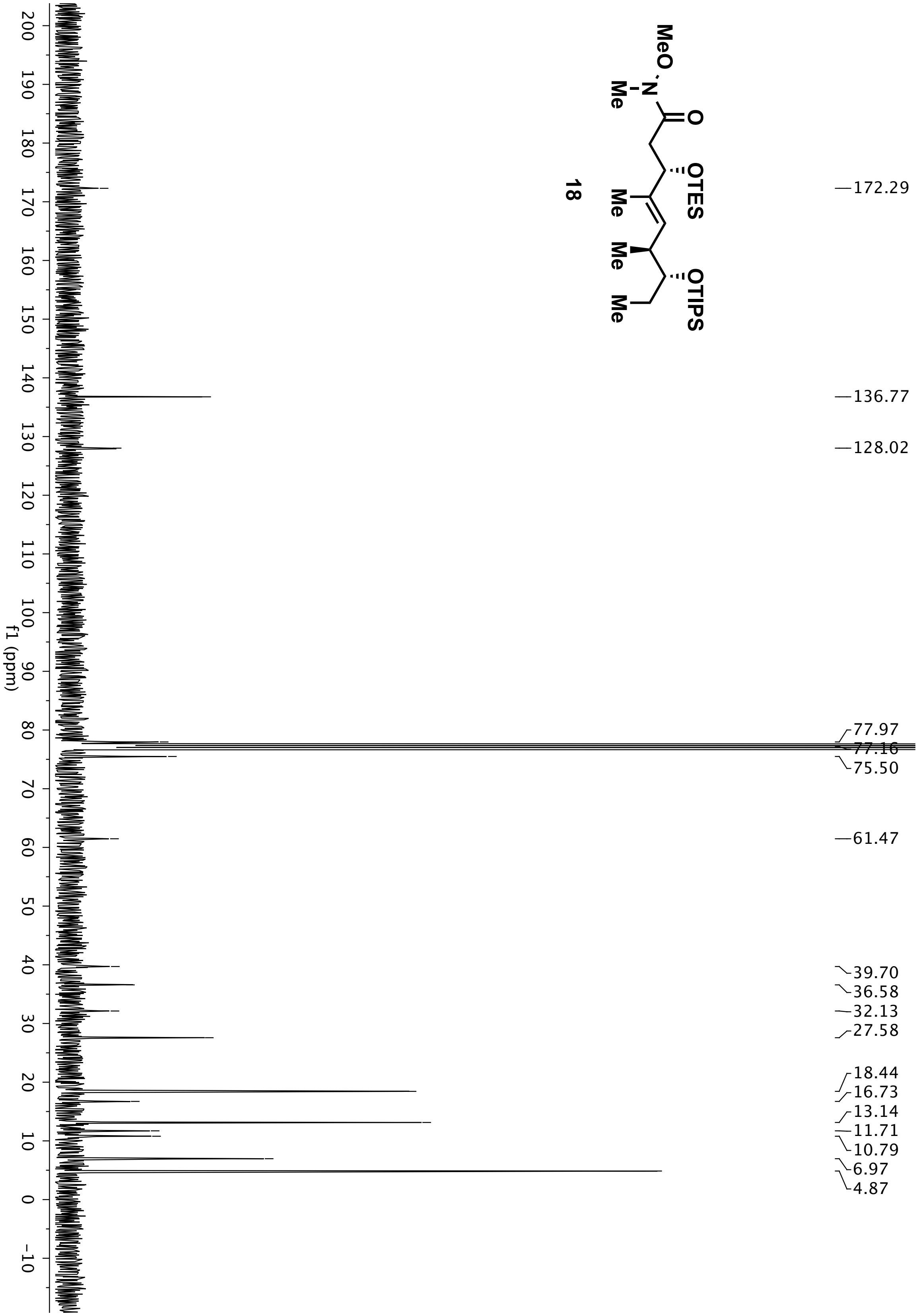

MeO
N
Me
O
OTES
OTIPS
Me
Me
Me
18
172.29
136.77
128.02
77.97
77.16
75.50
61.47
39.70
36.58
32.13
27.58
18.44
16.73
13.14
11.71
10.79
6.97
4.87
f1 (ppm)

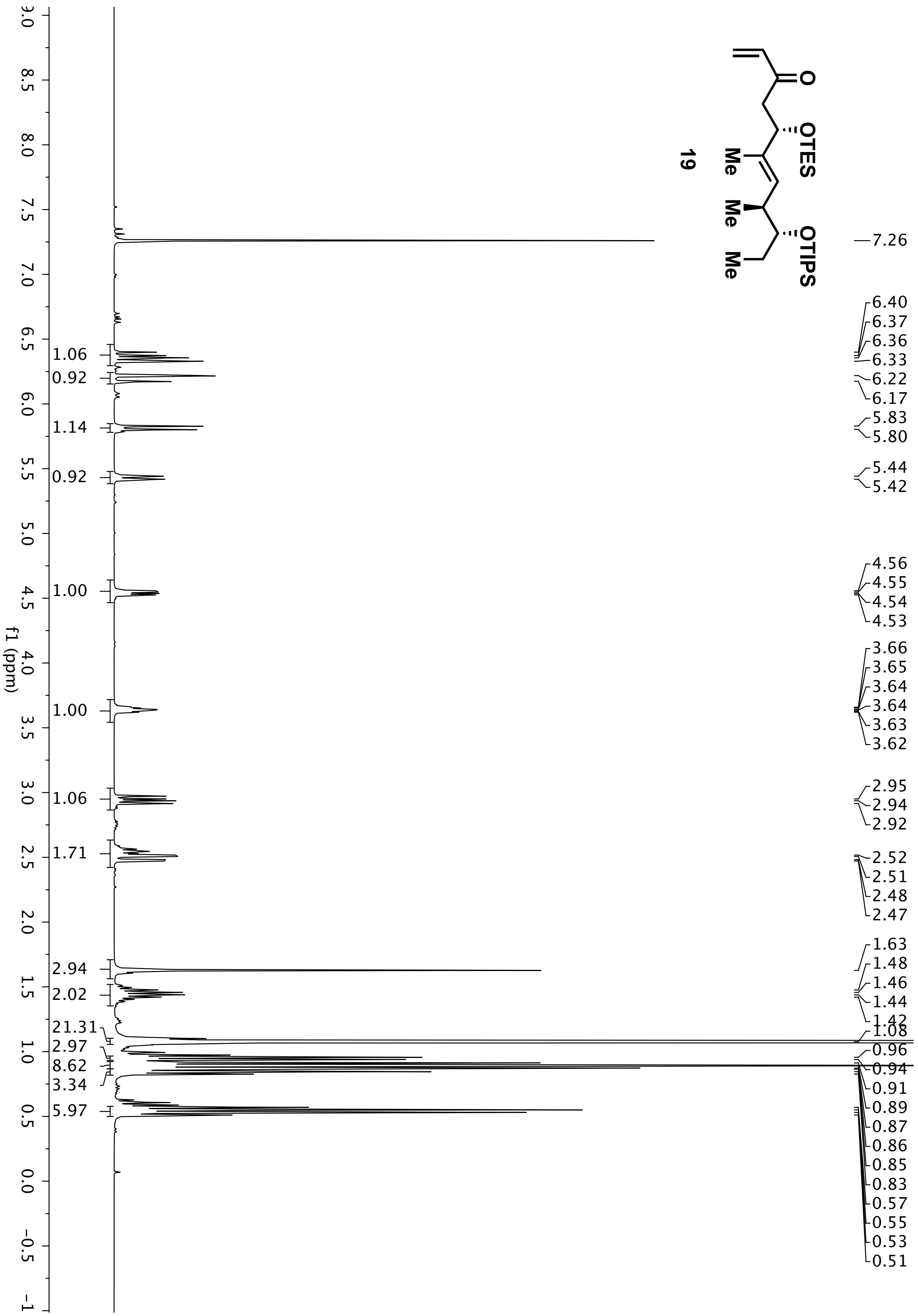
O
OTES
OTIPS
Me
Me
Me
19
7.26
6.40
6.37
6.36
6.33
6.22
6.17
5.83
5.80
5.44
5.42
4.56
4.55
4.54
4.53
3.66
3.65
3.64
3.64
3.63
3.62
2.95
2.94
2.92
2.52
2.51
2.48
2.47
1.63
1.48
1.46
1.44
1.42
1.08
0.96
0.94
0.91
0.89
0.87
0.86
0.85
0.83
0.57
0.55
0.53
0.51
1.06
0.92
1.14
0.92
1.00
1.00
1.06
1.71
2.94
2.02
21.31
2.97
8.62
3.34
5.97
f1 (ppm)

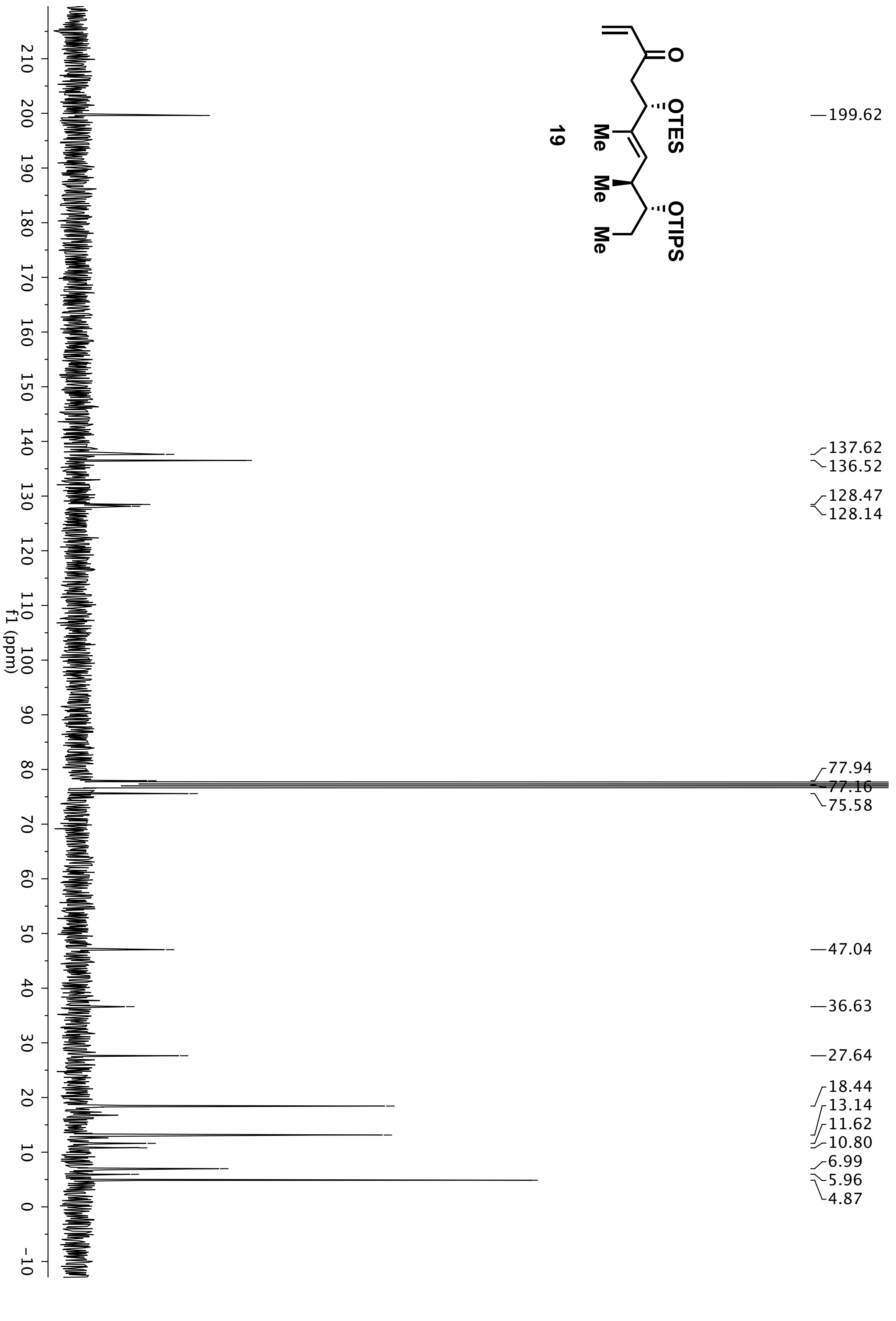
199.62
137.62
136.52
128.47
128.14
77.94
77.16
75.58
47.04
36.63
27.64
18.44
13.14
11.62
10.80
6.99
5.96
4.87
f1 (ppm)
O
OTES
OTIPS
Me
Me
Me
19

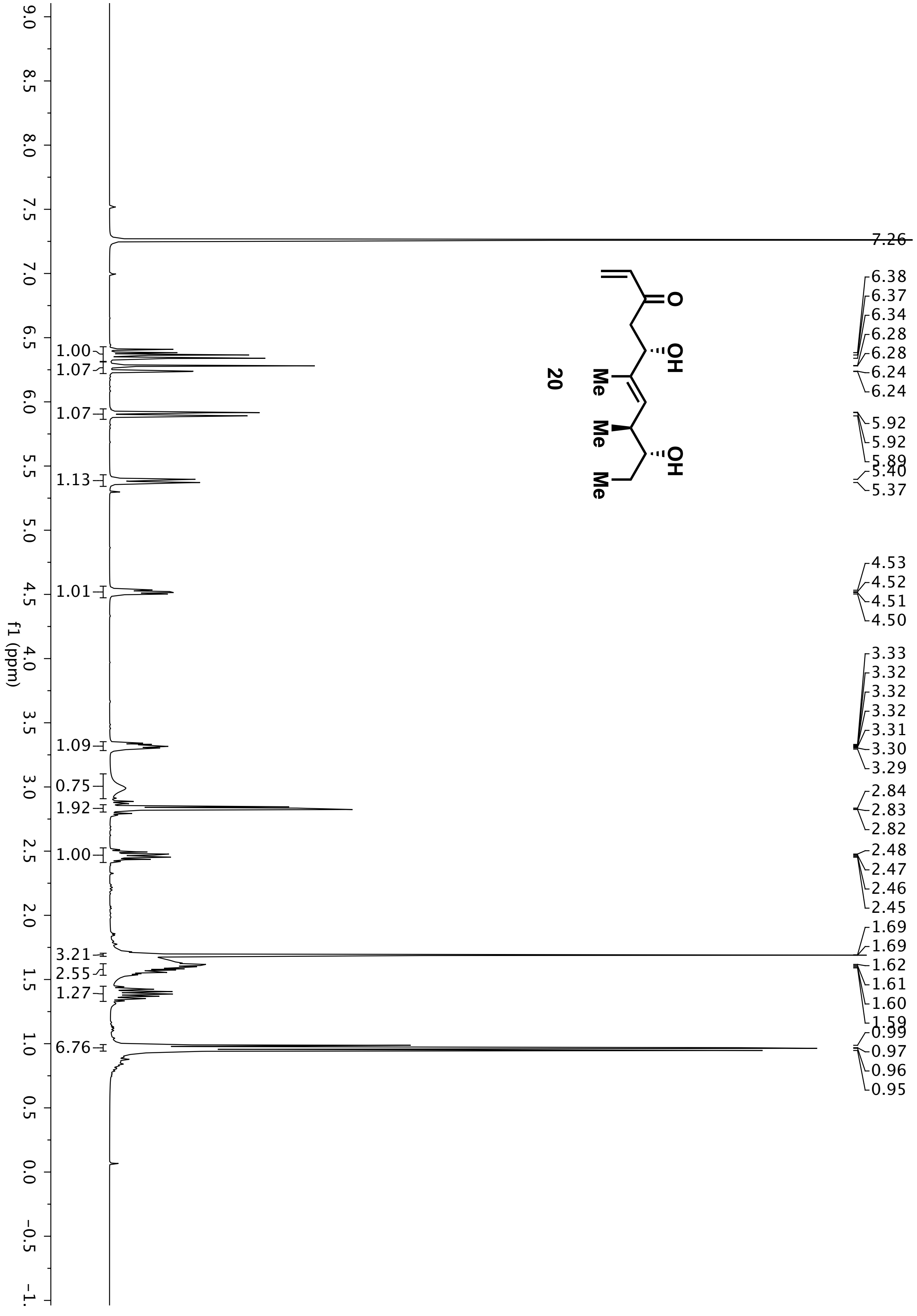
20
f1 (ppm)

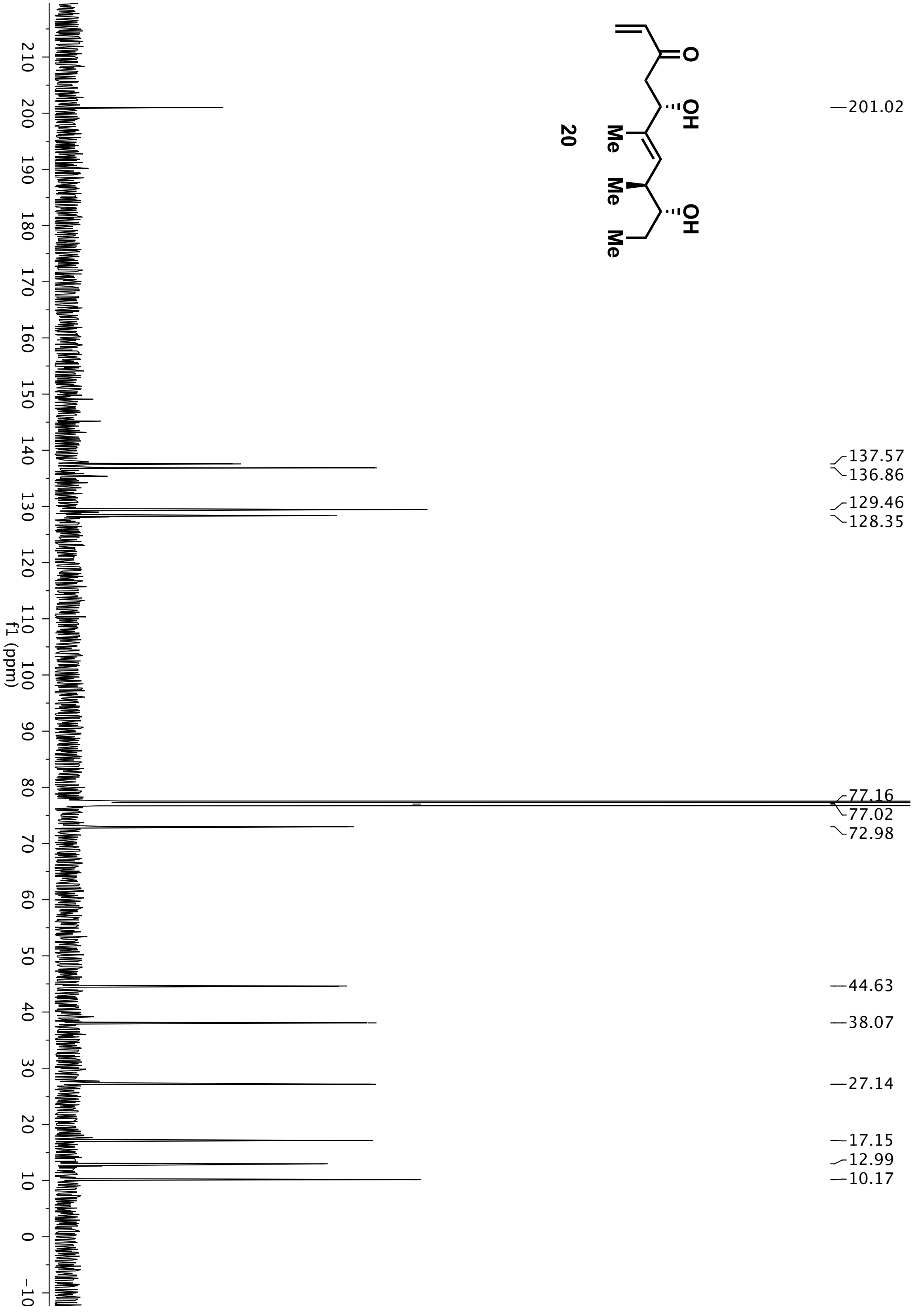
O
OH
OH
Me
Me
Me
20
201.02
137.57
136.86
129.46
128.35
77.16
77.02
72.98
44.63
38.07
27.14
17.15
12.99
10.17
f1 (ppm)

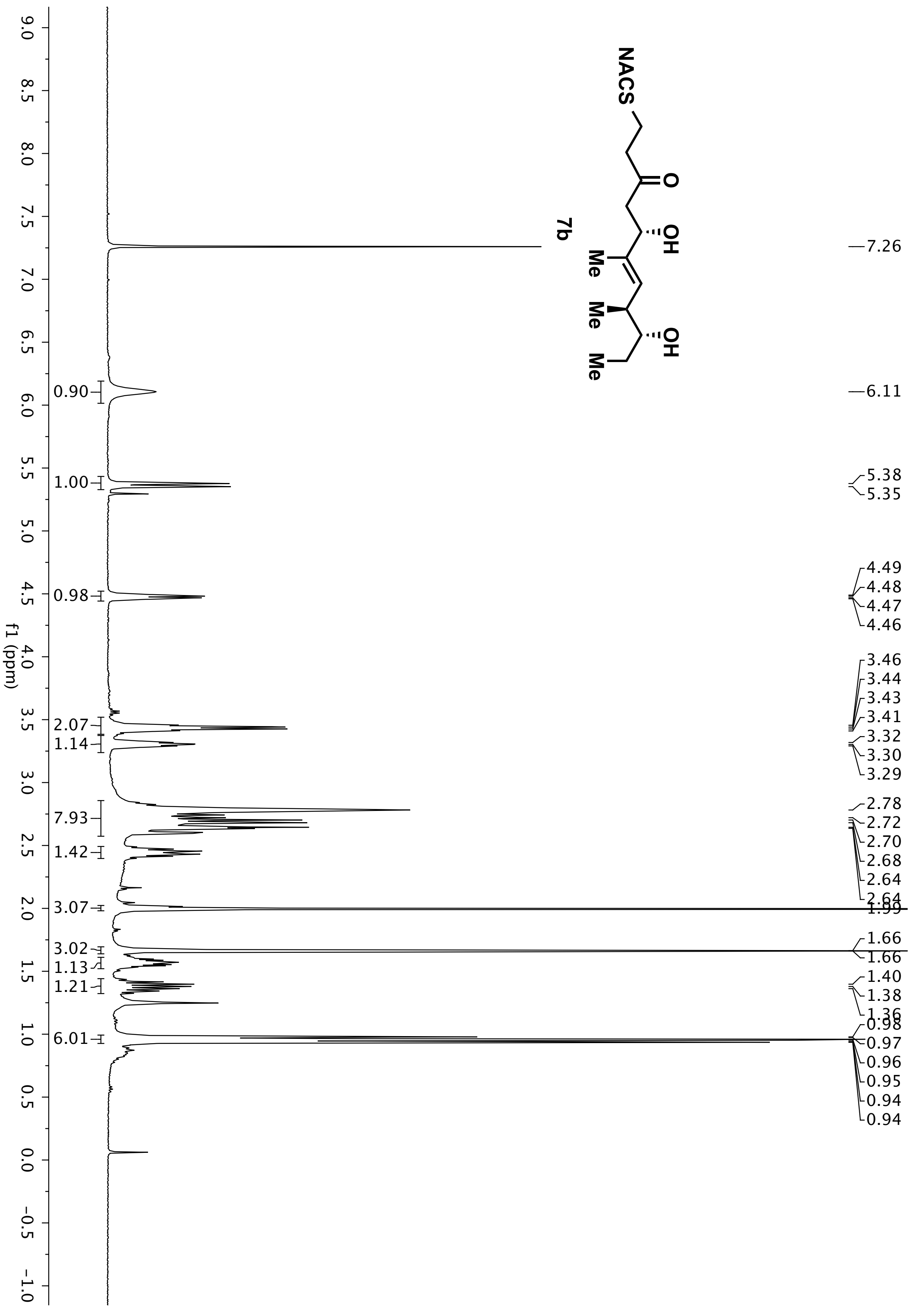
NACS
O
OH
OH
Me
Me
Me
7b
7.26
6.11
5.38
5.35
4.49
4.48
4.47
4.46
3.46
3.44
3.43
3.41
3.32
3.30
3.29
2.78
2.72
2.70
2.68
2.64
2.64
1.99
1.66
1.66
1.40
1.38
1.36
0.98
0.97
0.96
0.95
0.94
0.94
0.90
1.00
0.98
2.07
1.14
7.93
1.42
3.07
3.02
1.13
1.21
6.01
f1 (ppm)

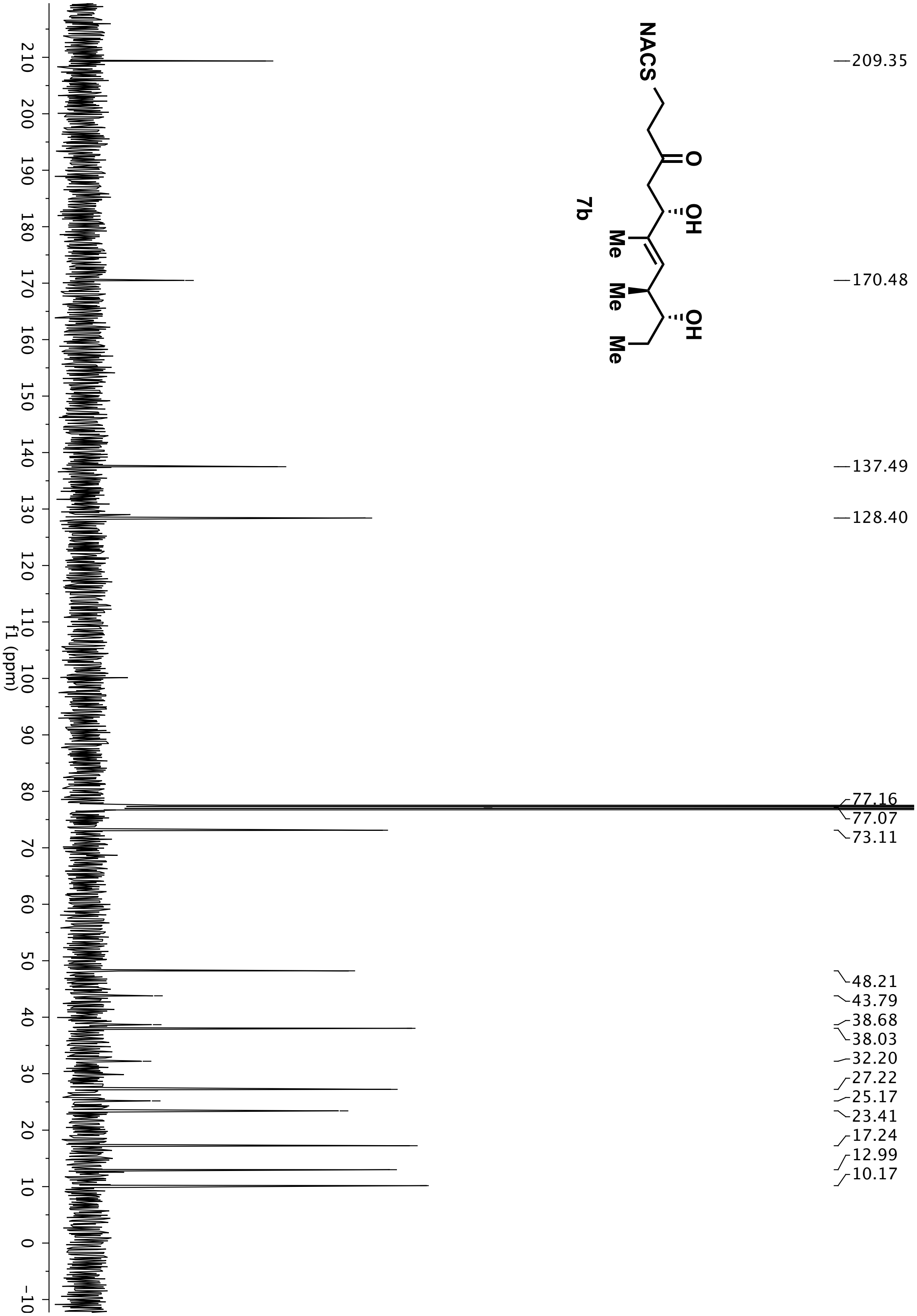

NACS
O
OH
OH
Me
Me
Me
7b
209.35
170.48
137.49
128.40
77.16
77.07
73.11
48.21
43.79
38.68
38.03
32.20
27.22
25.17
23.41
17.24
12.99
10.17
f1 (ppm)

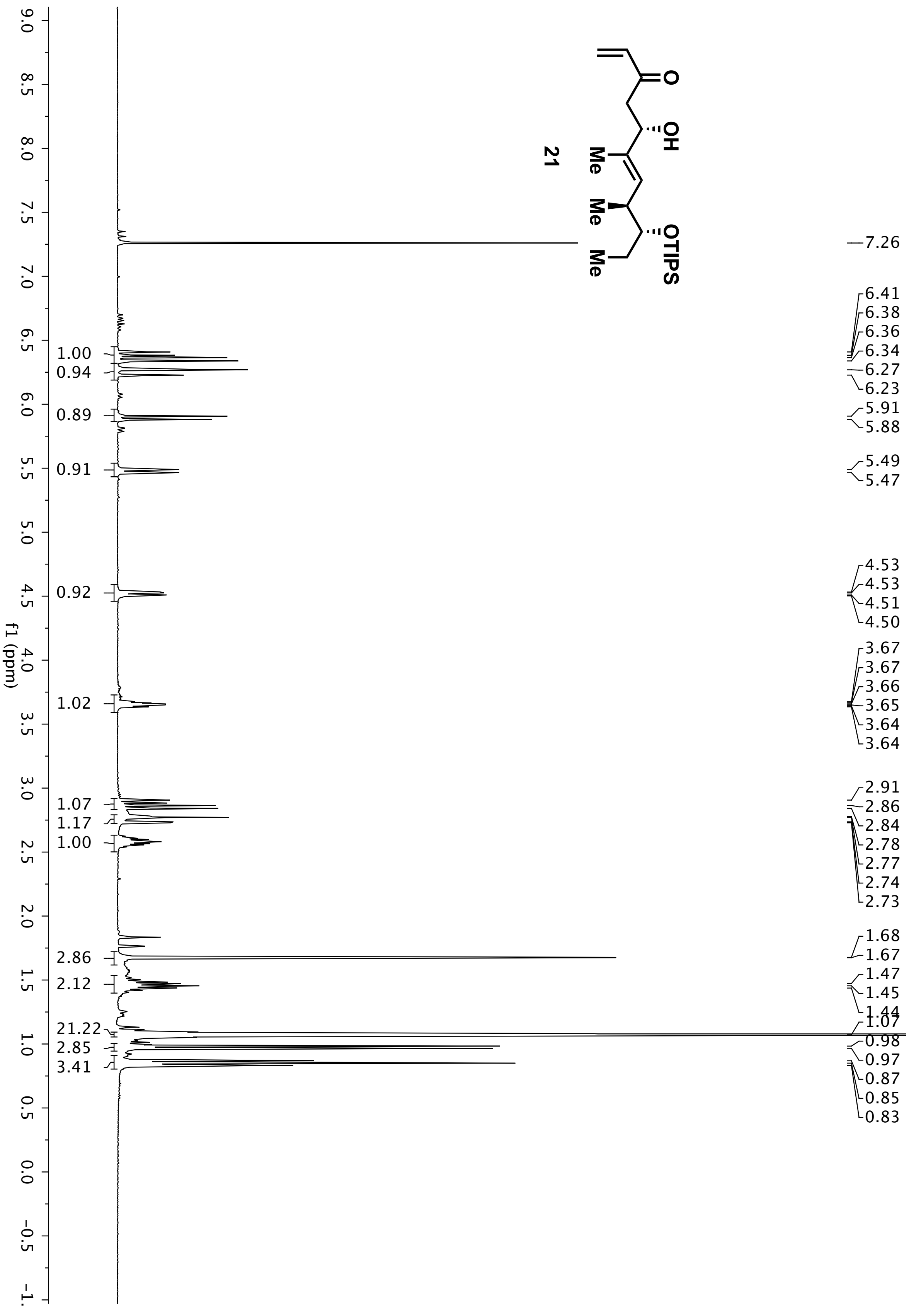

OTIPS
OH
O
Me
Me
Me
21
f1 (ppm)
7.26
6.41
6.38
6.36
6.34
6.27
6.23
5.91
5.88
5.49
5.47
4.53
4.53
4.51
4.50
3.67
3.67
3.66
3.65
3.64
3.64
2.91
2.86
2.84
2.78
2.77
2.74
2.73
1.68
1.67
1.47
1.45
1.44
1.07
0.98
0.97
0.87
0.85
0.83
1.00
0.94
0.89
0.91
0.92
1.02
1.07
1.17
1.00
2.86
2.12
21.22
2.85
3.41

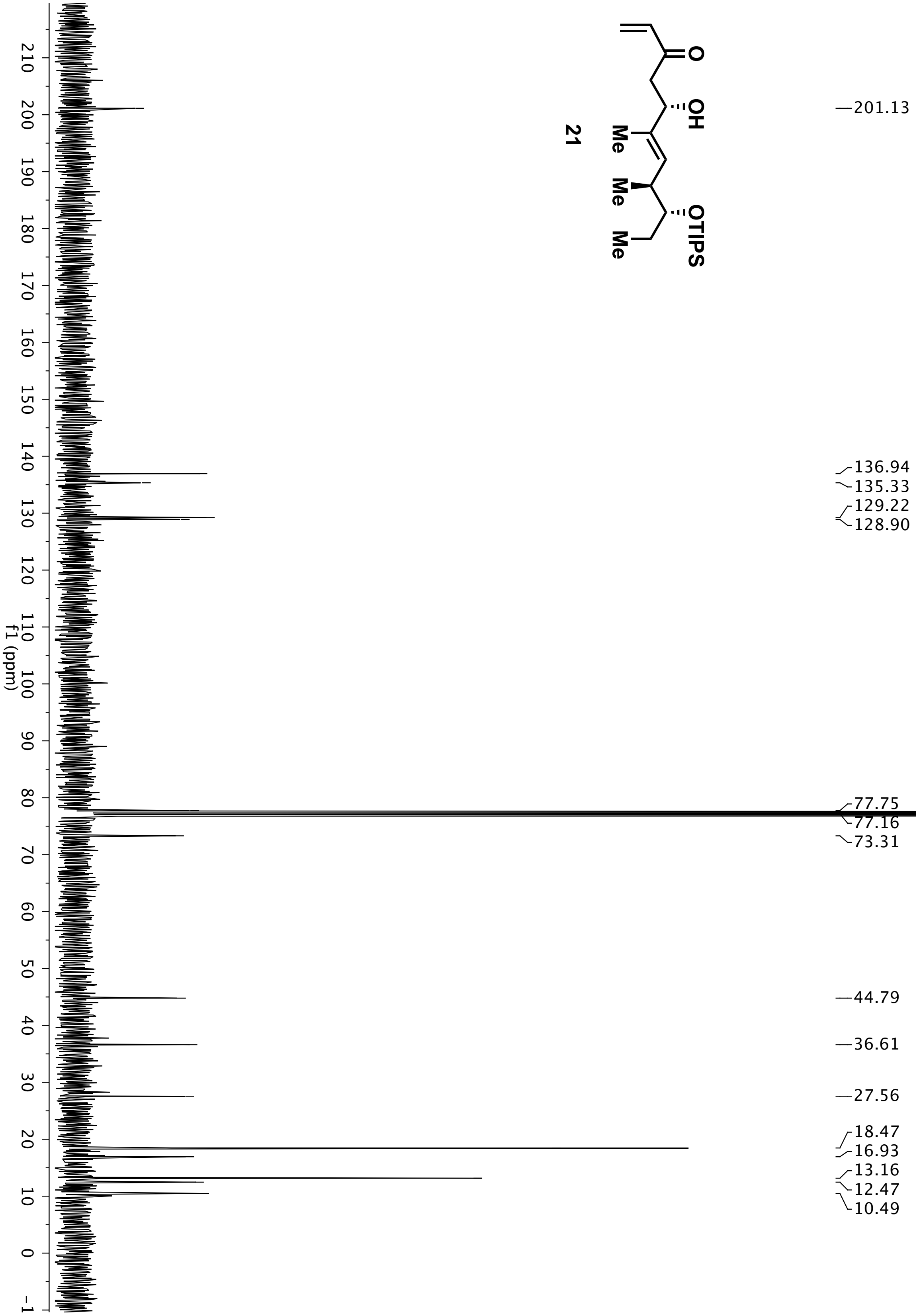

O
OH
OTIPS
Me
Me
Me
21
201.13
136.94
135.33
129.22
128.90
77.75
77.16
73.31
44.79
36.61
27.56
18.47
16.93
13.16
12.47
10.49
f1 (ppm)

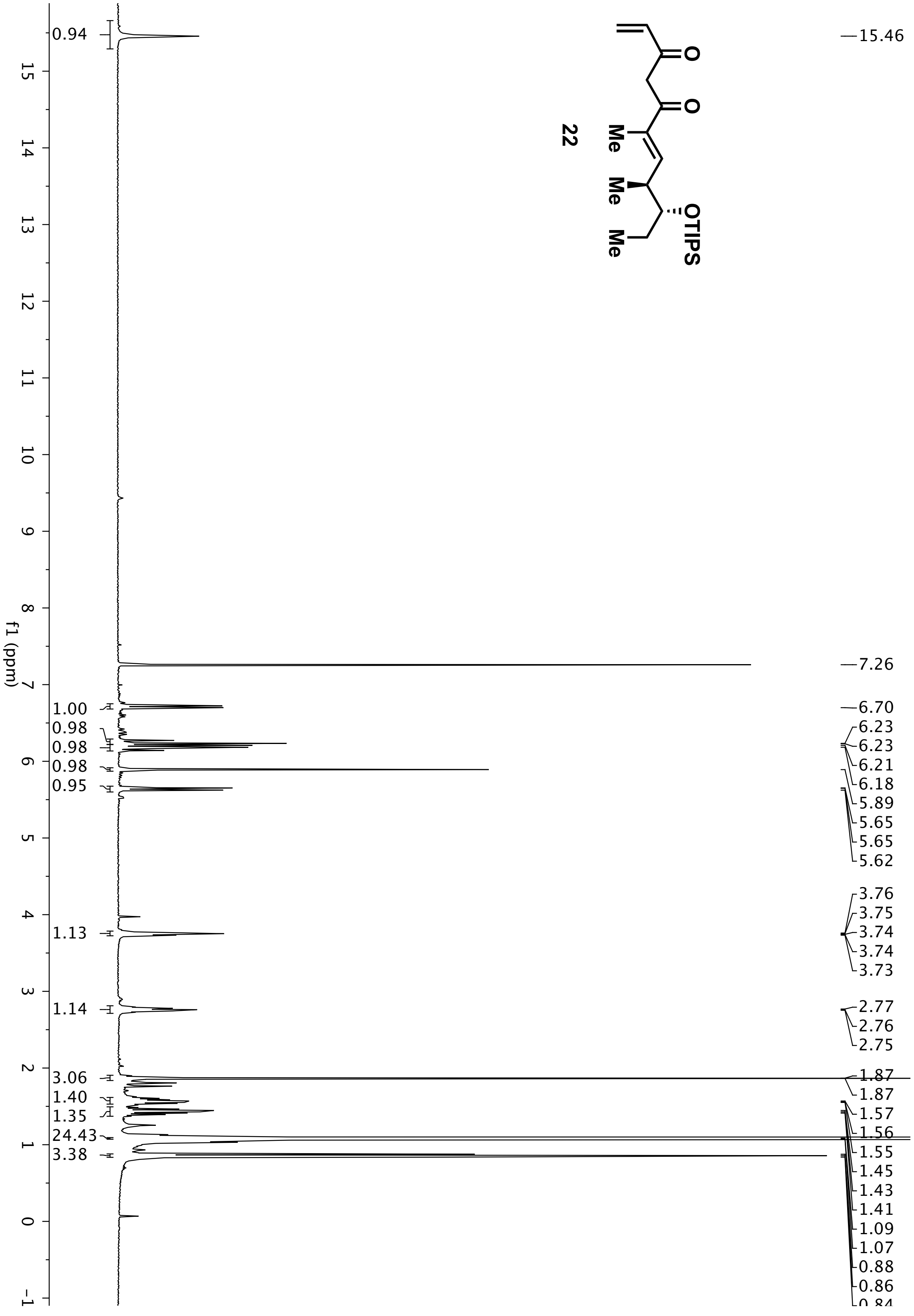

22
OTIPS
Me
Me
Me
O
O
15.46
7.26
6.70
6.23
6.23
6.21
6.18
5.89
5.65
5.65
5.62
3.76
3.75
3.74
3.74
3.73
2.77
2.76
2.75
1.87
1.87
1.57
1.56
1.55
1.45
1.43
1.41
1.09
1.07
0.88
0.86
0.94
1.00
0.98
0.98
0.98
0.95
1.13
1.14
3.06
1.40
1.35
24.43
3.38
f1 (ppm)
15
14
13
12
11
10
9
8
7
6
5
4
3
2
1
0
−1

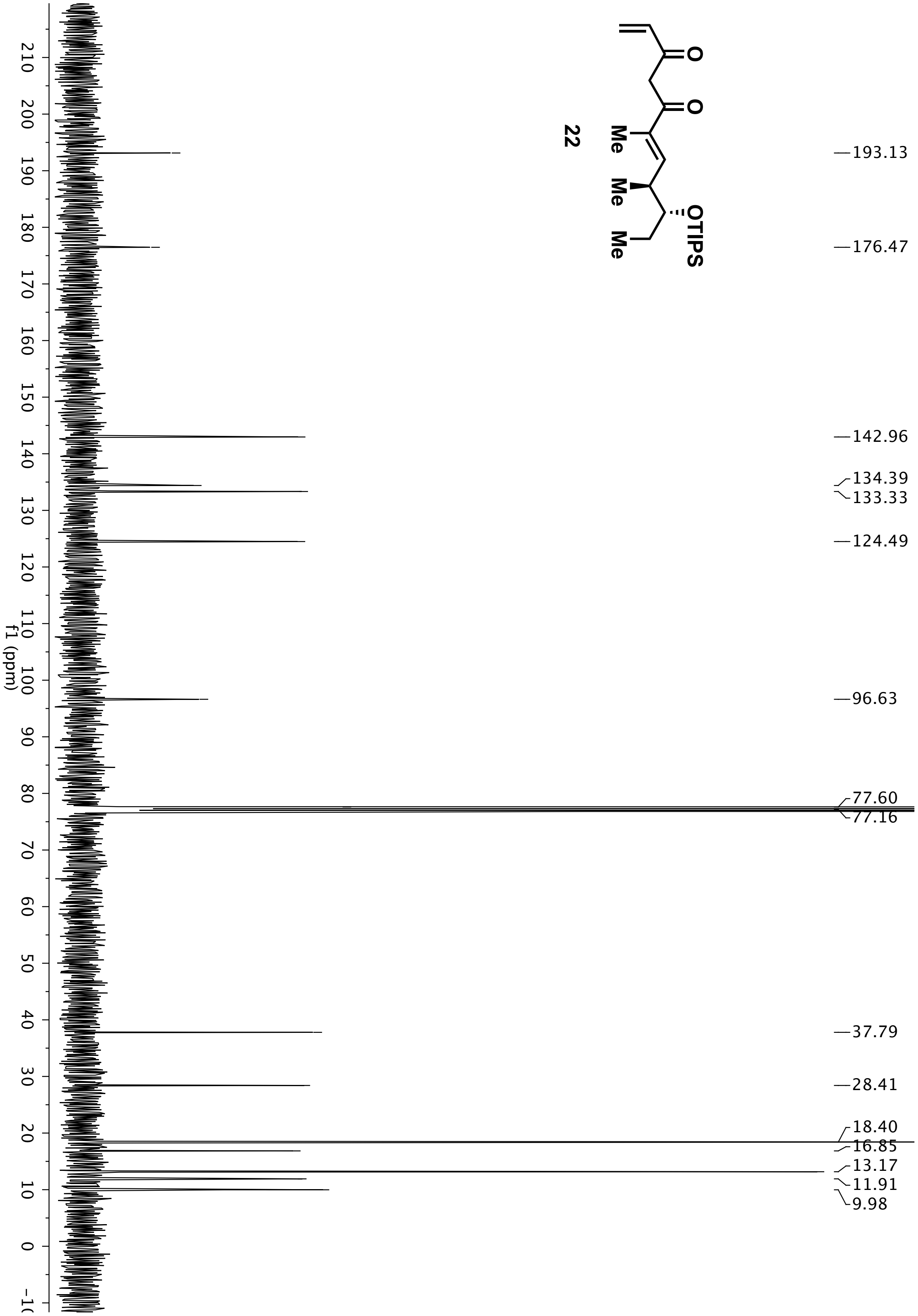

22
OTIPS
Me
Me
Me
O
O
193.13
176.47
142.96
134.39
133.33
124.49
96.63
77.60
77.16
37.79
28.41
18.40
16.85
13.17
11.91
9.98
f1 (ppm)

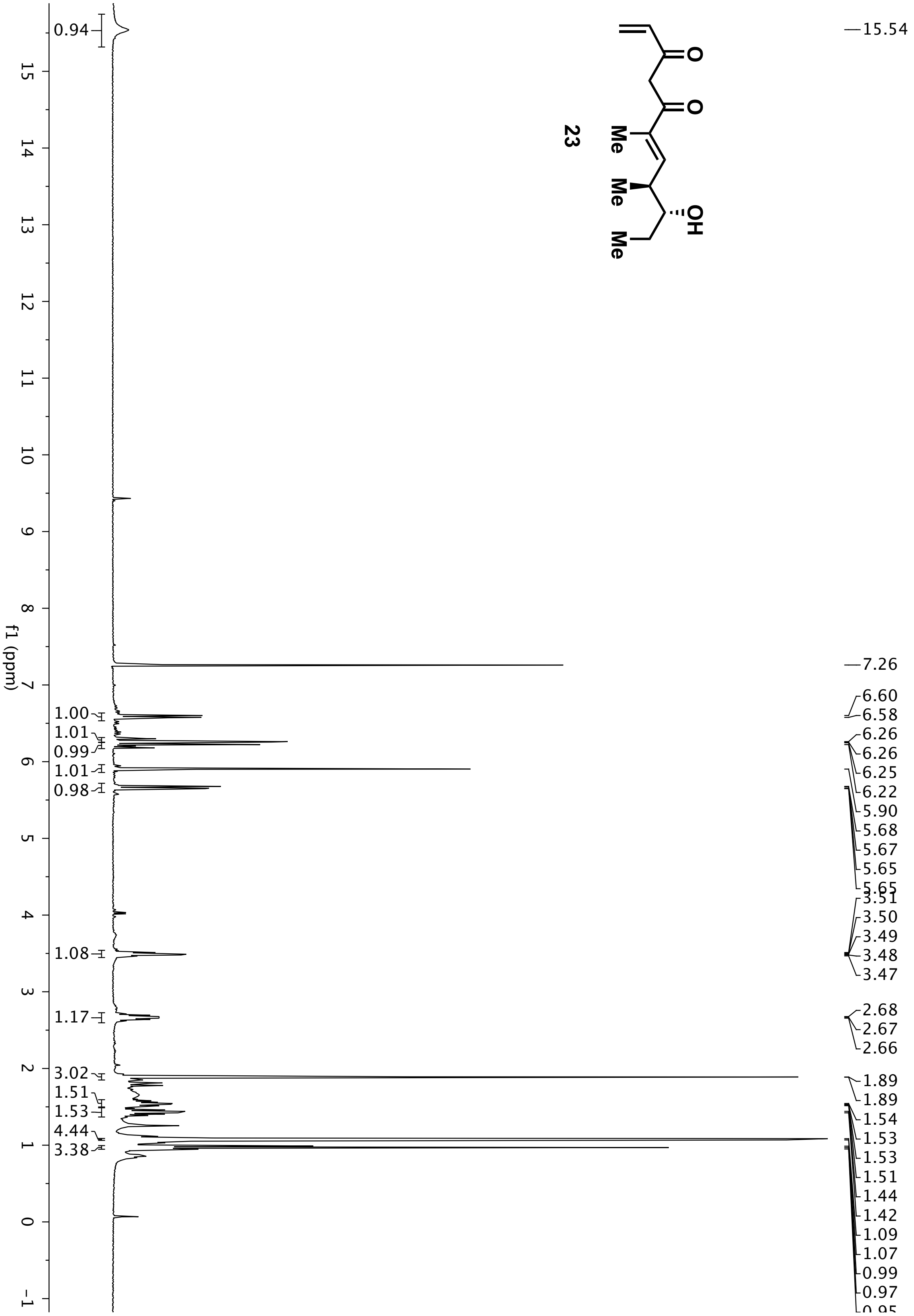
23
f1 (ppm)

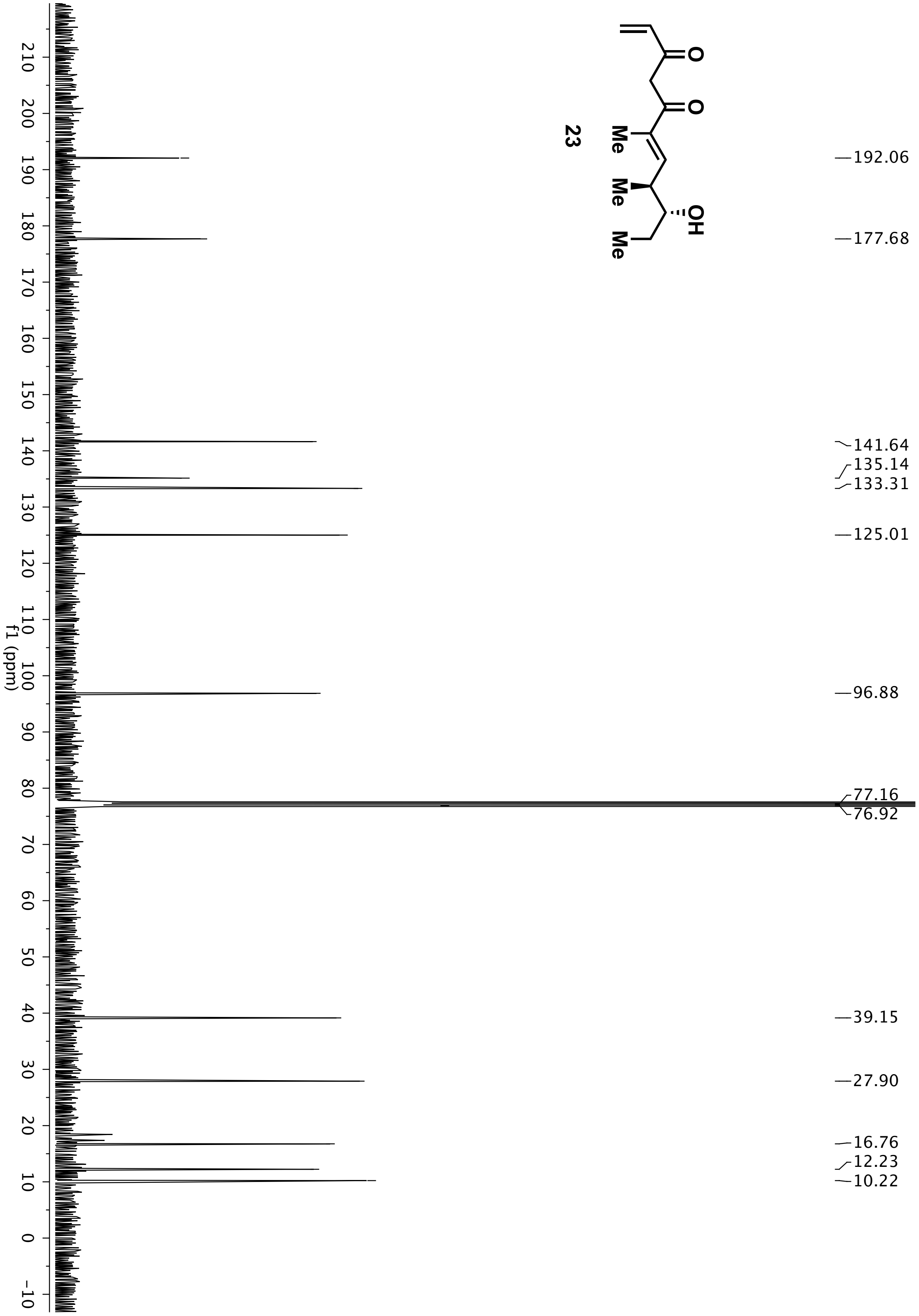
23
Me
Me
Me
OH
O
O
192.06
177.68
141.64
135.14
133.31
125.01
96.88
77.16
76.92
39.15
27.90
16.76
12.23
10.22
f1 (ppm)

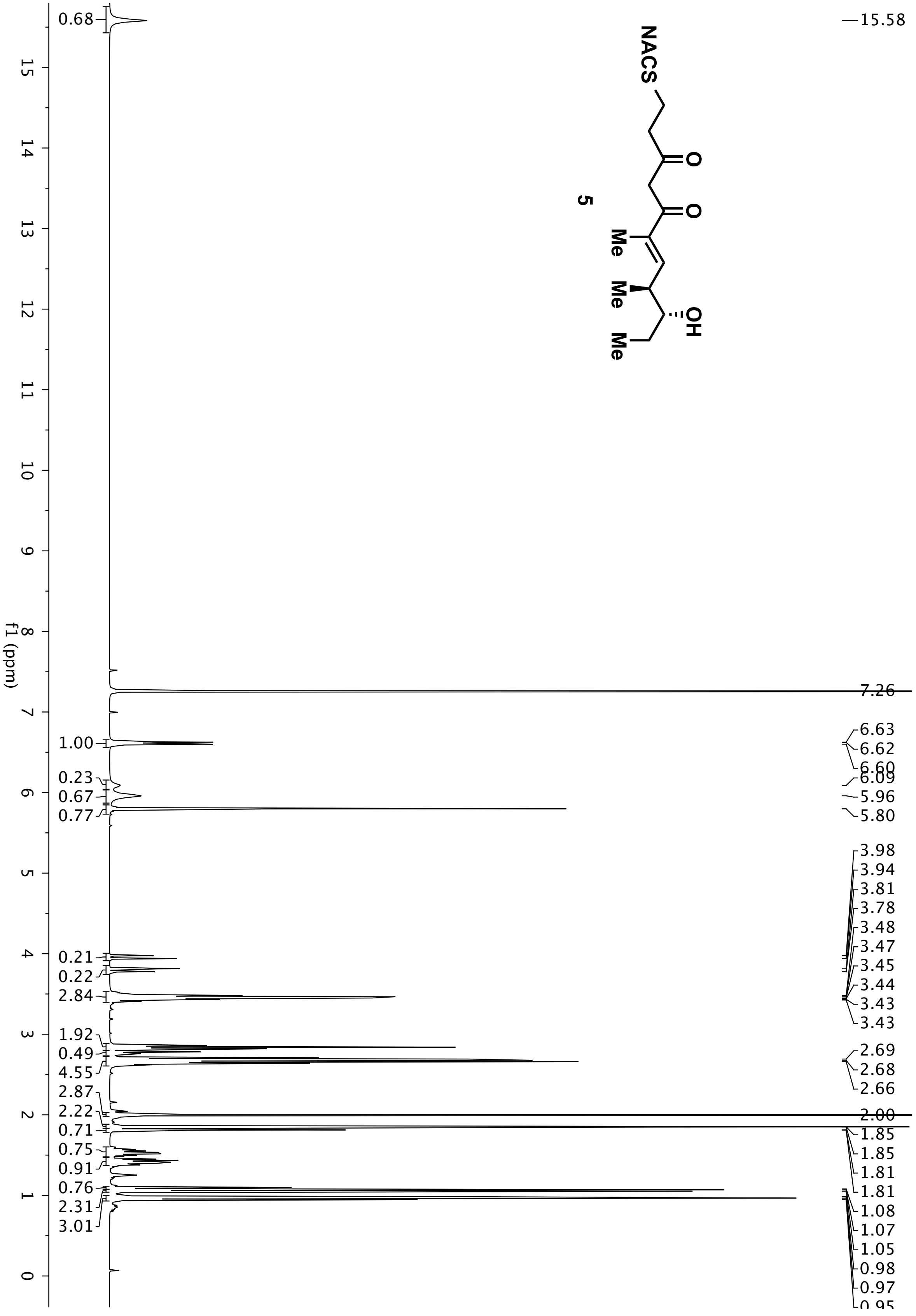

NACS
O
O
OH
Me
Me
Me
5
15.58
7.26
6.63
6.62
6.60
6.09
5.96
5.80
3.98
3.94
3.81
3.78
3.48
3.47
3.45
3.44
3.43
3.43
2.69
2.68
2.66
2.00
1.85
1.85
1.81
1.81
1.08
1.07
1.05
0.98
0.97
0.95
0.68
1.00
0.23
0.67
0.77
0.21
0.22
2.84
1.92
0.49
4.55
2.87
2.22
0.71
0.75
0.91
0.76
2.31
3.01
f1 (ppm)

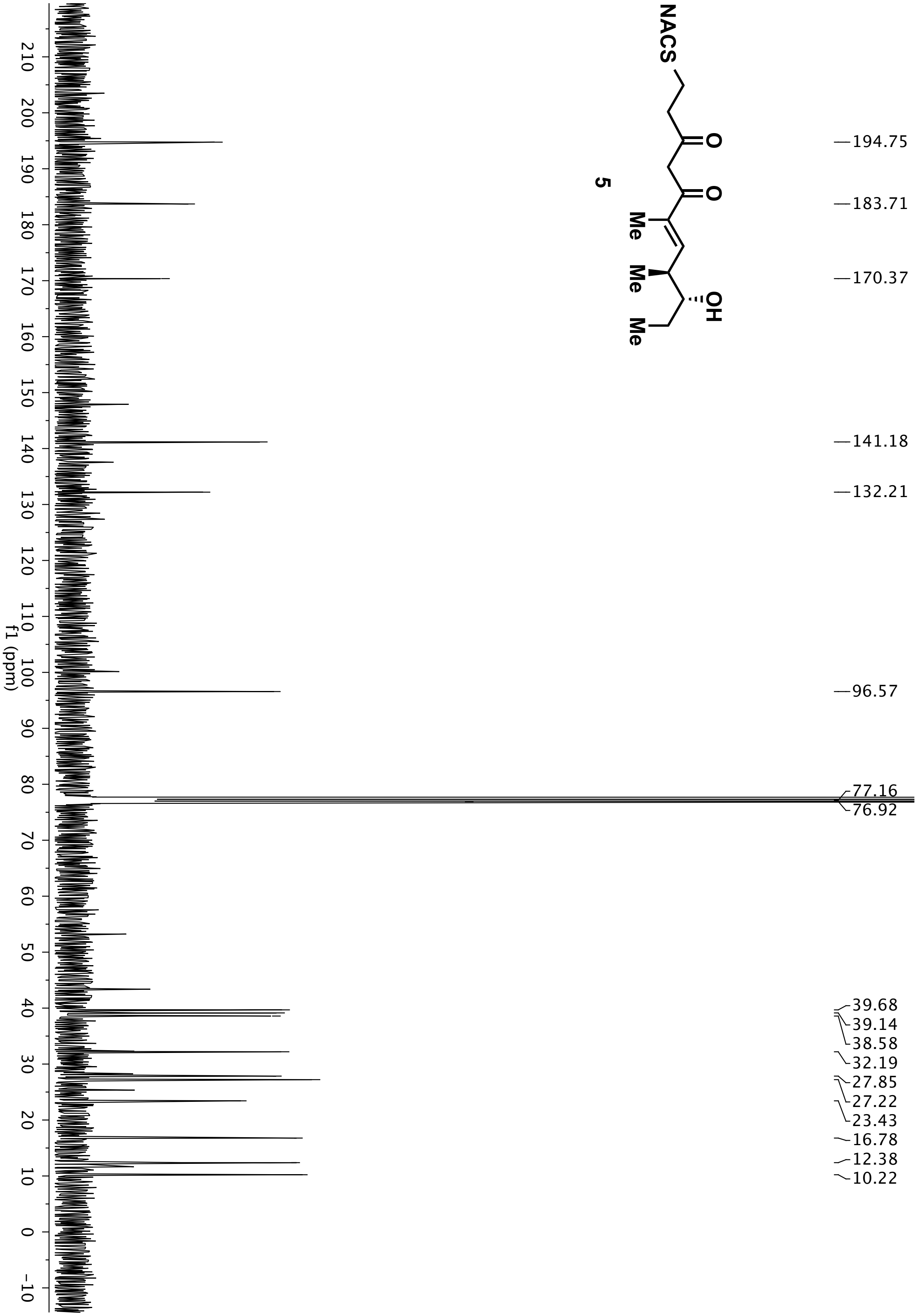
NACS
O
O
Me
Me
OH
Me
5
194.75
183.71
170.37
141.18
132.21
96.57
77.16
76.92
39.68
39.14
38.58
32.19
27.85
27.22
23.43
16.78
12.38
10.22
f1 (ppm)

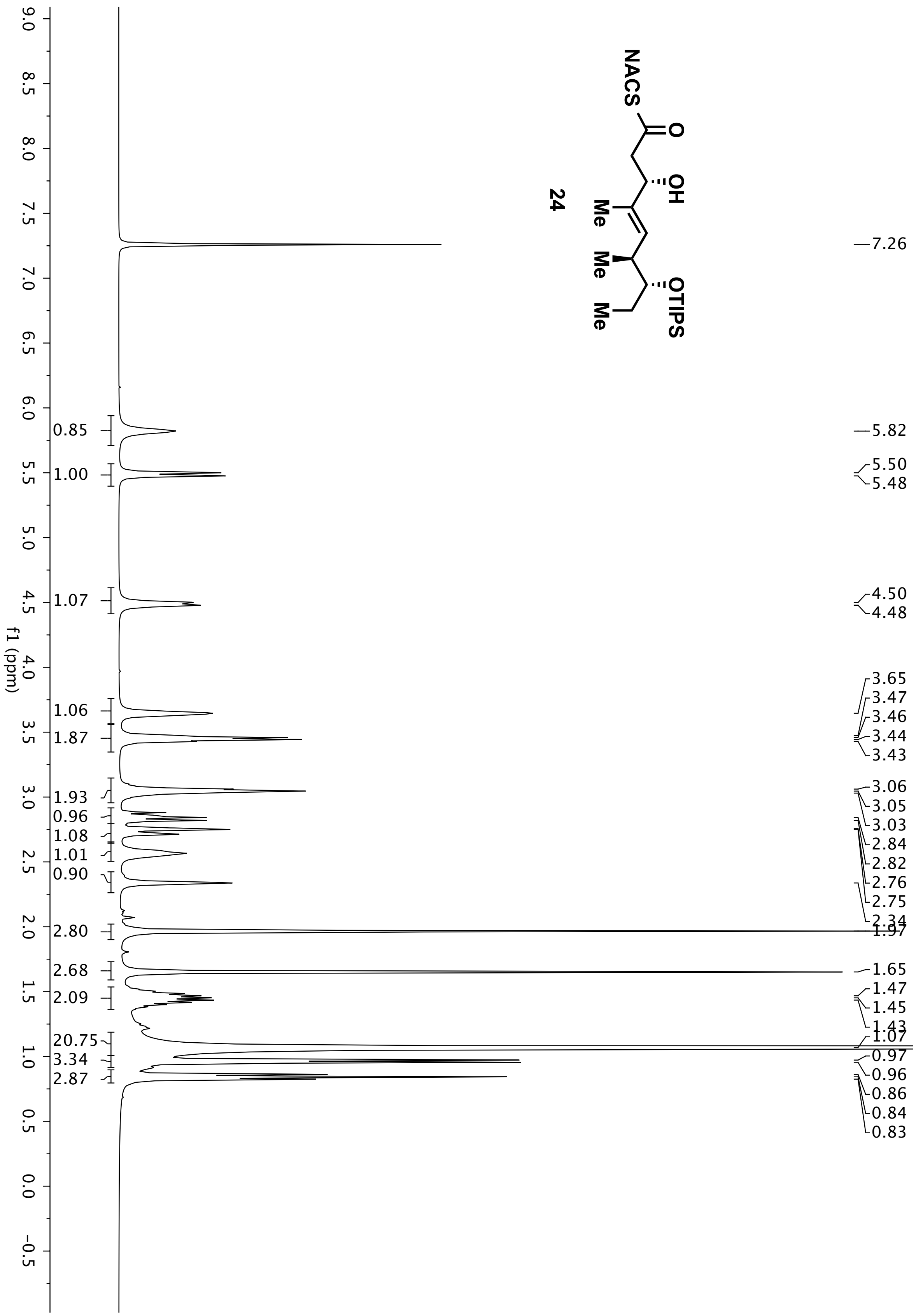
NACS
O
OH
OTIPS
Me
Me
Me
24
f1 (ppm)
7.26
5.82
5.50
5.48
4.50
4.48
3.65
3.47
3.46
3.44
3.43
3.06
3.05
3.03
2.84
2.82
2.76
2.75
2.34
1.97
1.65
1.47
1.45
1.43
1.07
0.97
0.96
0.86
0.84
0.83
0.85
1.00
1.07
1.06
1.87
1.93
0.96
1.08
1.01
0.90
2.80
2.68
2.09
20.75
3.34
2.87

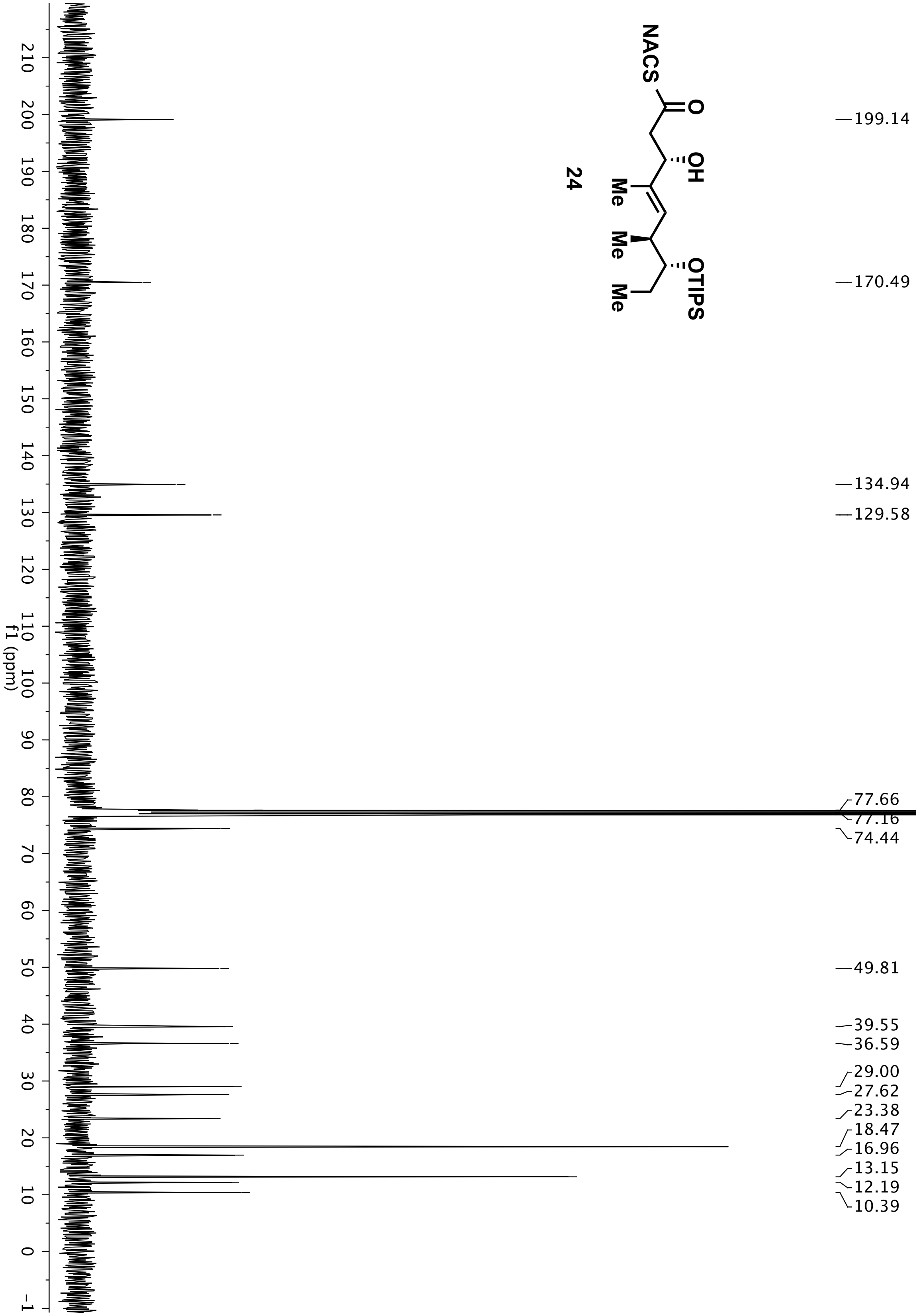

NACS
O
OH
Me
Me
OTIPS
Me
24
199.14
170.49
134.94
129.58
77.66
77.16
74.44
49.81
39.55
36.59
29.00
27.62
23.38
18.47
16.96
13.15
12.19
10.39
f1 (ppm)

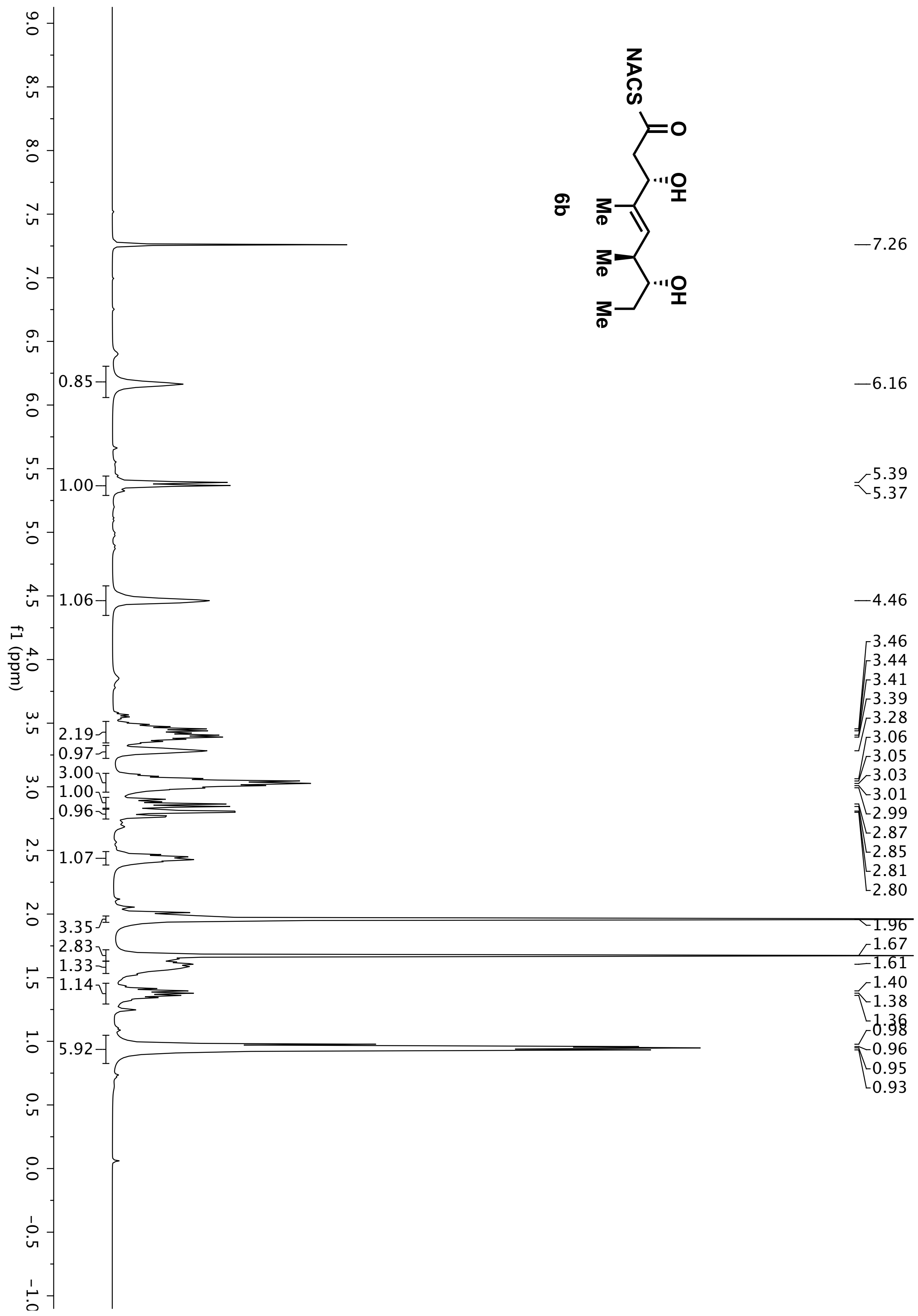
NACS
OH
Me
OH
Me
Me
6b
f1 (ppm)

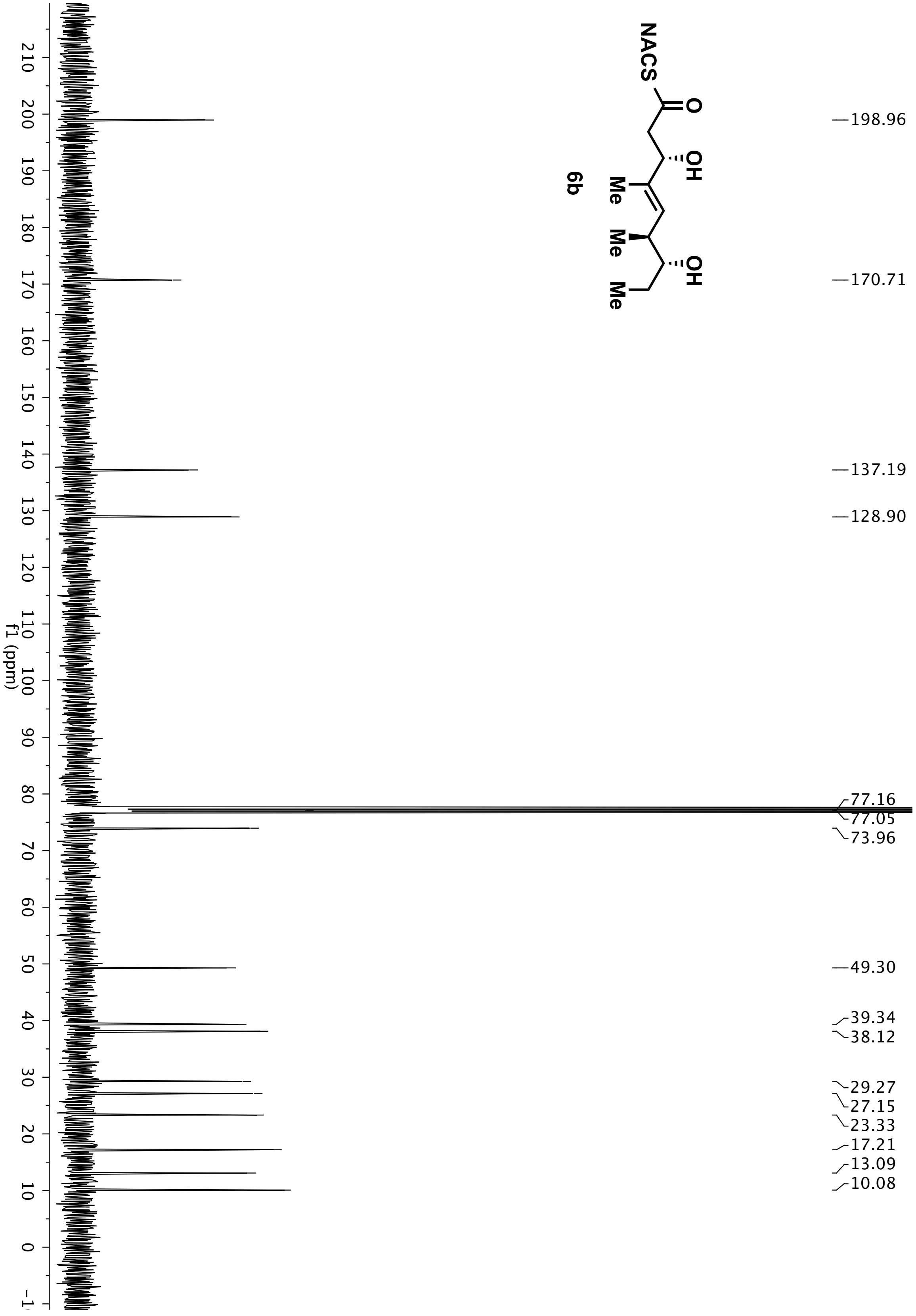
NACS
O
OH
OH
Me
Me
Me
6b
198.96
170.71
137.19
128.90
77.16
77.05
73.96
49.30
39.34
38.12
29.27
27.15
23.33
17.21
13.09
10.08
f1 (ppm)

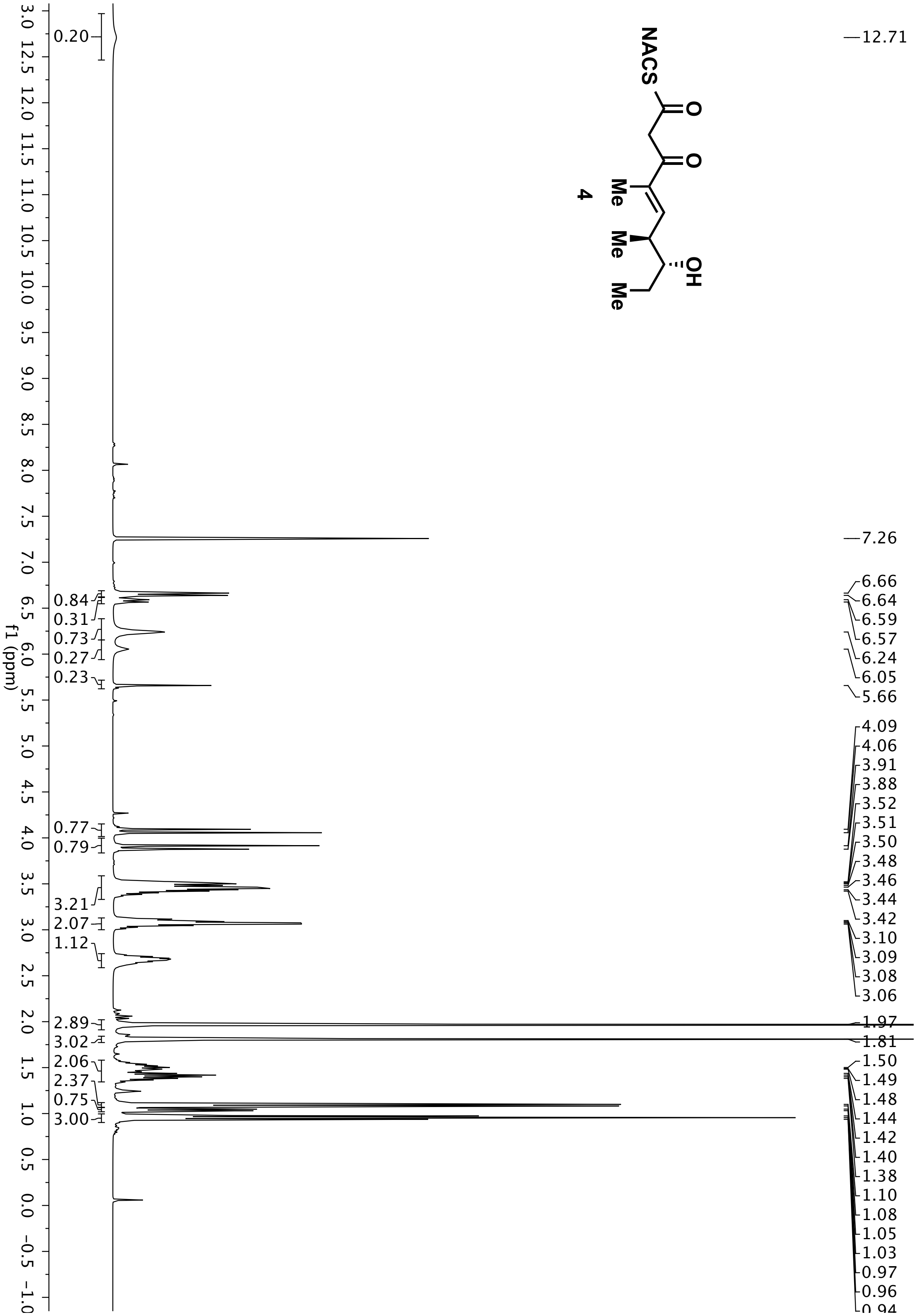
NACS
O
O
Me
Me
OH
Me
4
f1 (ppm)
12.71
7.26
6.66
6.64
6.59
6.57
6.24
6.05
5.66
4.09
4.06
3.91
3.88
3.52
3.51
3.50
3.48
3.46
3.44
3.42
3.10
3.09
3.08
3.06
1.97
1.81
1.50
1.49
1.48
1.44
1.42
1.40
1.38
1.10
1.08
1.05
1.03
0.97
0.96
0.20
0.84
0.31
0.73
0.27
0.23
0.77
0.79
3.21
2.07
1.12
2.89
3.02
2.06
2.37
0.75
3.00

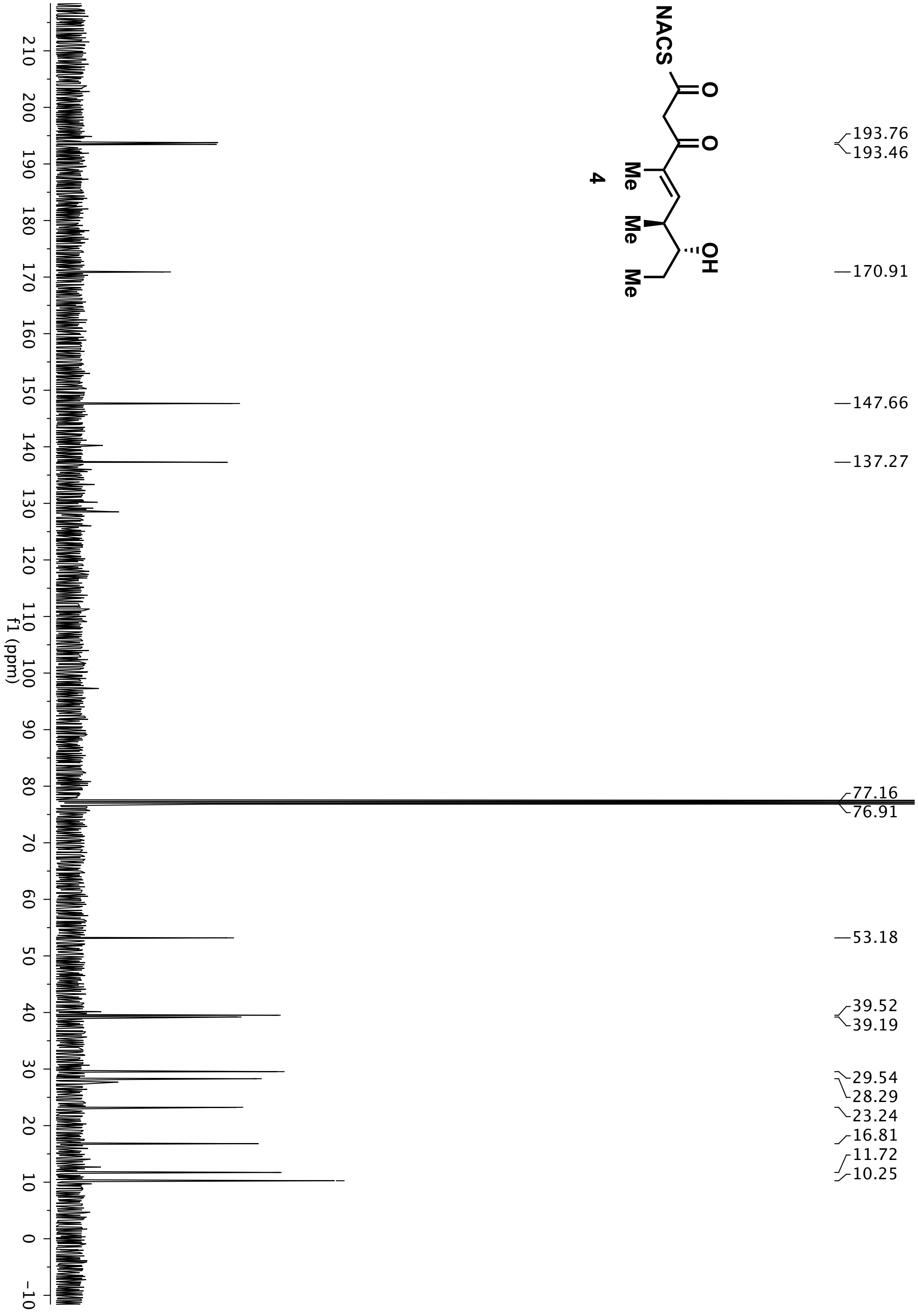

NACS
O
O
Me
Me
OH
Me
4
193.76
193.46
170.91
147.66
137.27
77.16
76.91
53.18
39.52
39.19
29.54
28.29
23.24
16.81
11.72
10.25
f1 (ppm)

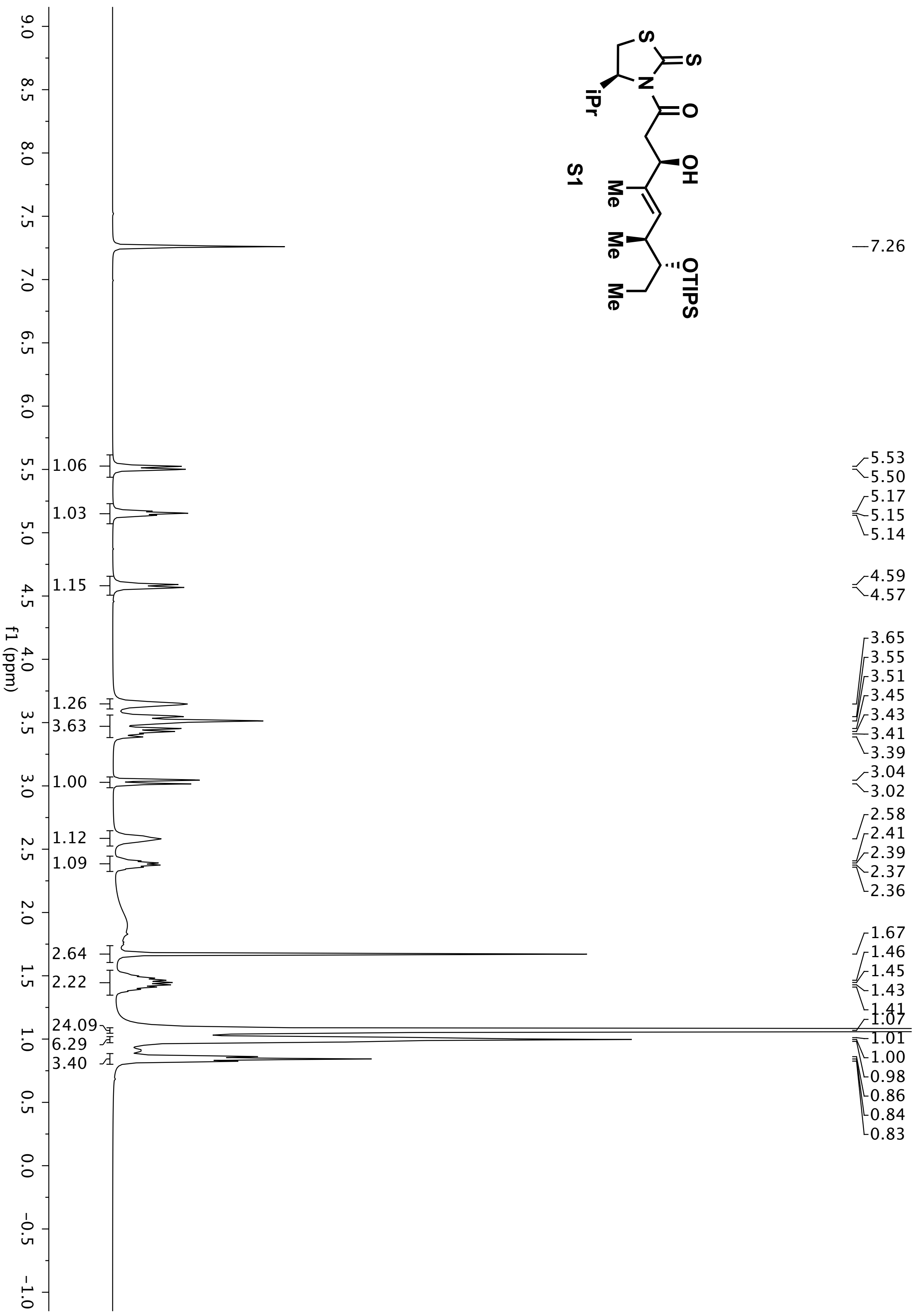
S1
OH
OTIPS
Me
Me
Me
iPr
O
S
N
f1 (ppm)
9.0 8.5 8.0 7.5 7.0 6.5 6.0 5.5 5.0 4.5 4.0 3.5 3.0 2.5 2.0 1.5 1.0 0.5 0.0 −0.5 −1.0
1.06 1.03 1.15 1.26 3.63 1.00 1.12 1.09 2.64 2.22 24.09 6.29 3.40
7.26
5.53 5.50 5.17 5.15 5.14
4.59 4.57
3.65 3.55 3.51 3.45 3.43 3.41 3.39
3.04 3.02
2.58 2.41 2.39 2.37 2.36
1.67 1.46 1.45 1.43 1.41 1.07
1.01 1.00 0.98 0.86 0.84 0.83

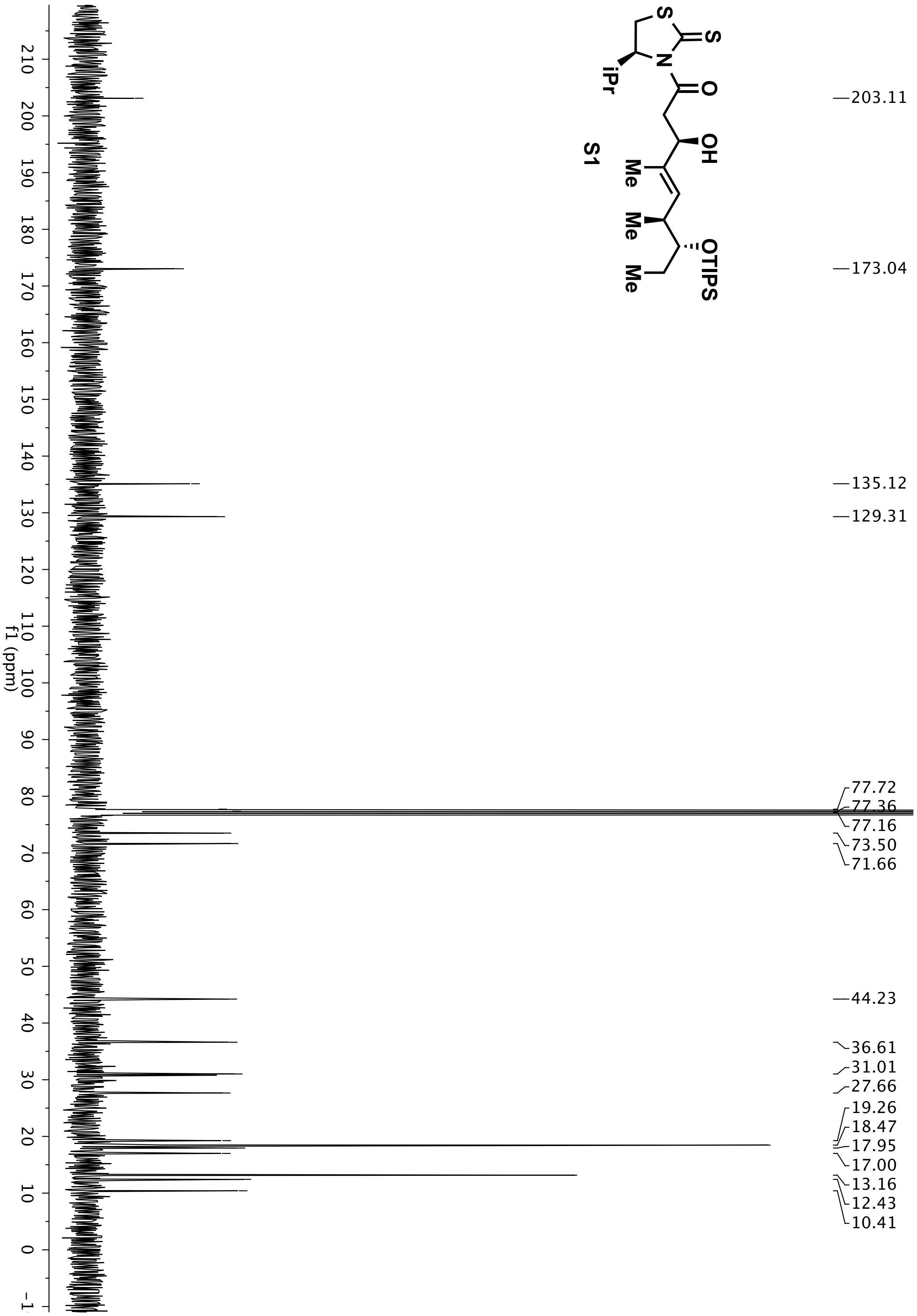

S1
OH
OTIPS
iPr
Me
203.11
173.04
135.12
129.31
77.72
77.36
77.16
73.50
71.66
44.23
36.61
31.01
27.66
19.26
18.47
17.95
17.00
13.16
12.43
10.41
f1 (ppm)

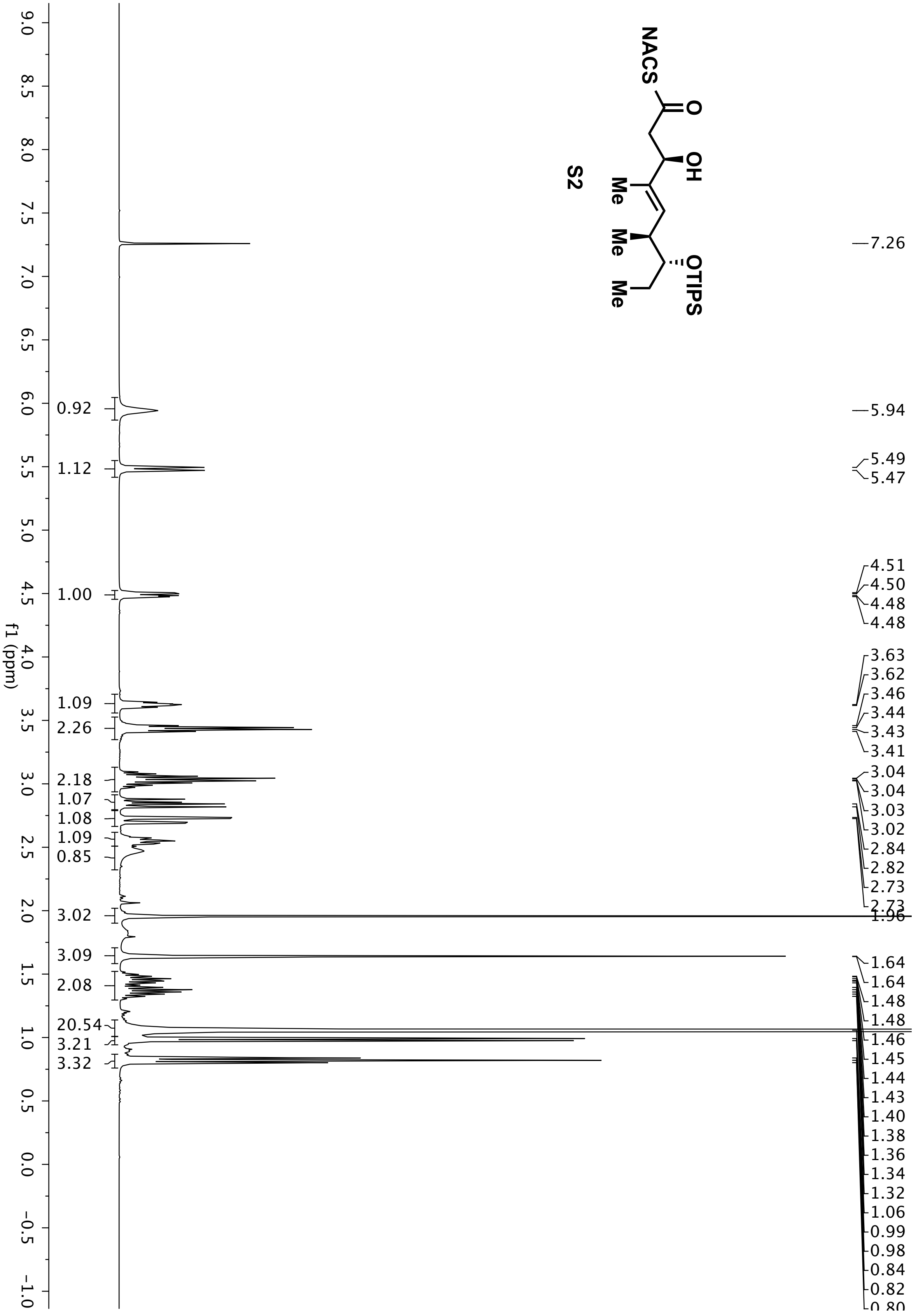
NACS
O
OH
OTIPS
Me
Me
Me
S2
7.26
5.94
5.49
5.47
4.51
4.50
4.48
4.48
3.63
3.62
3.46
3.44
3.43
3.41
3.04
3.04
3.03
3.02
2.84
2.82
2.73
2.73
1.96
1.64
1.64
1.48
1.48
1.46
1.45
1.44
1.43
1.40
1.38
1.36
1.34
1.32
1.06
0.99
0.98
0.84
0.82
0.92
1.12
1.00
1.09
2.26
2.18
1.07
1.08
1.09
0.85
3.02
3.09
2.08
20.54
3.21
3.32
f1 (ppm)

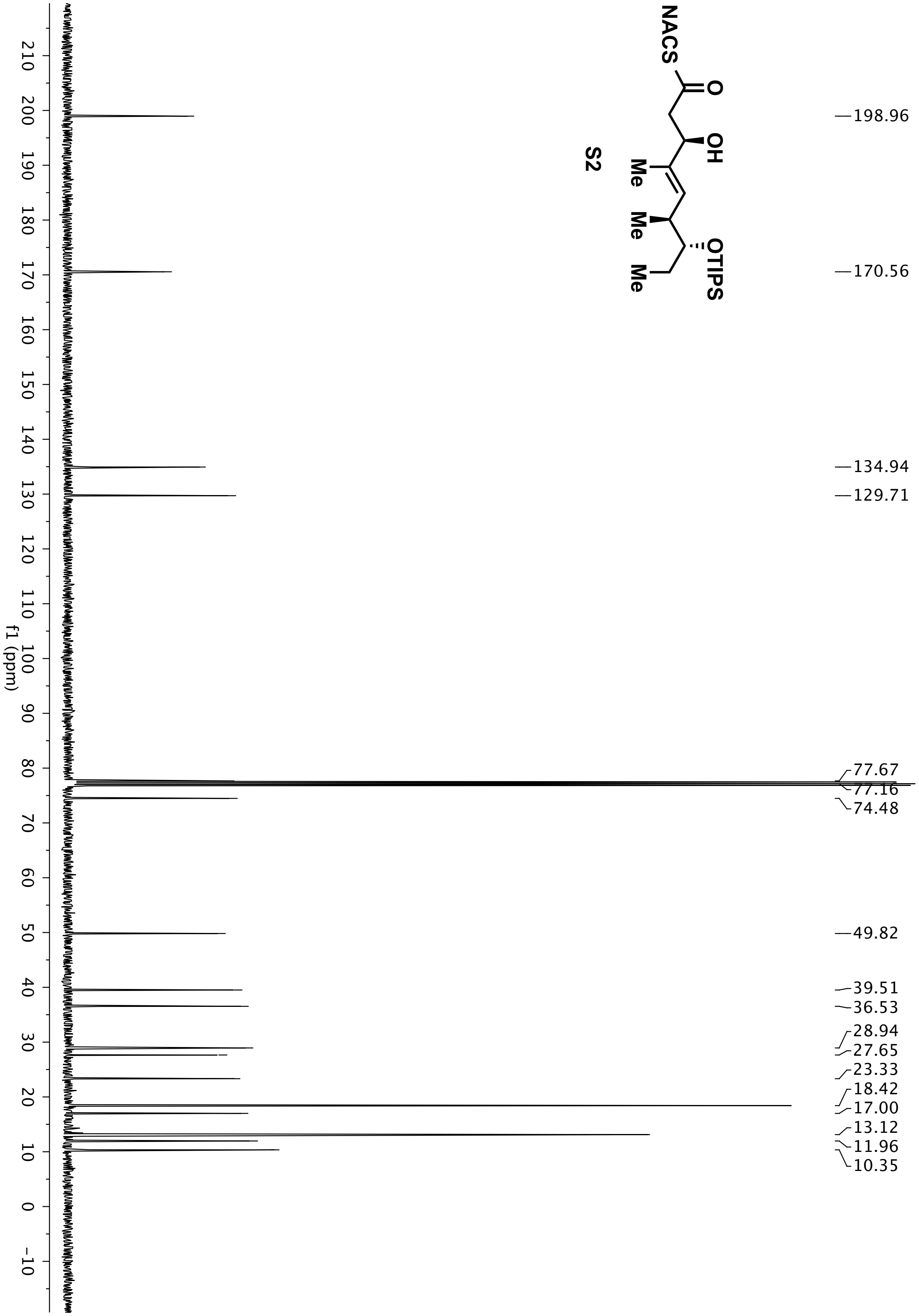
NACS
O
OH
OTIPS
Me
Me
Me
S2
198.96
170.56
134.94
129.71
77.67
77.16
74.48
49.82
39.51
36.53
28.94
27.65
23.33
18.42
17.00
13.12
11.96
10.35
f1 (ppm)

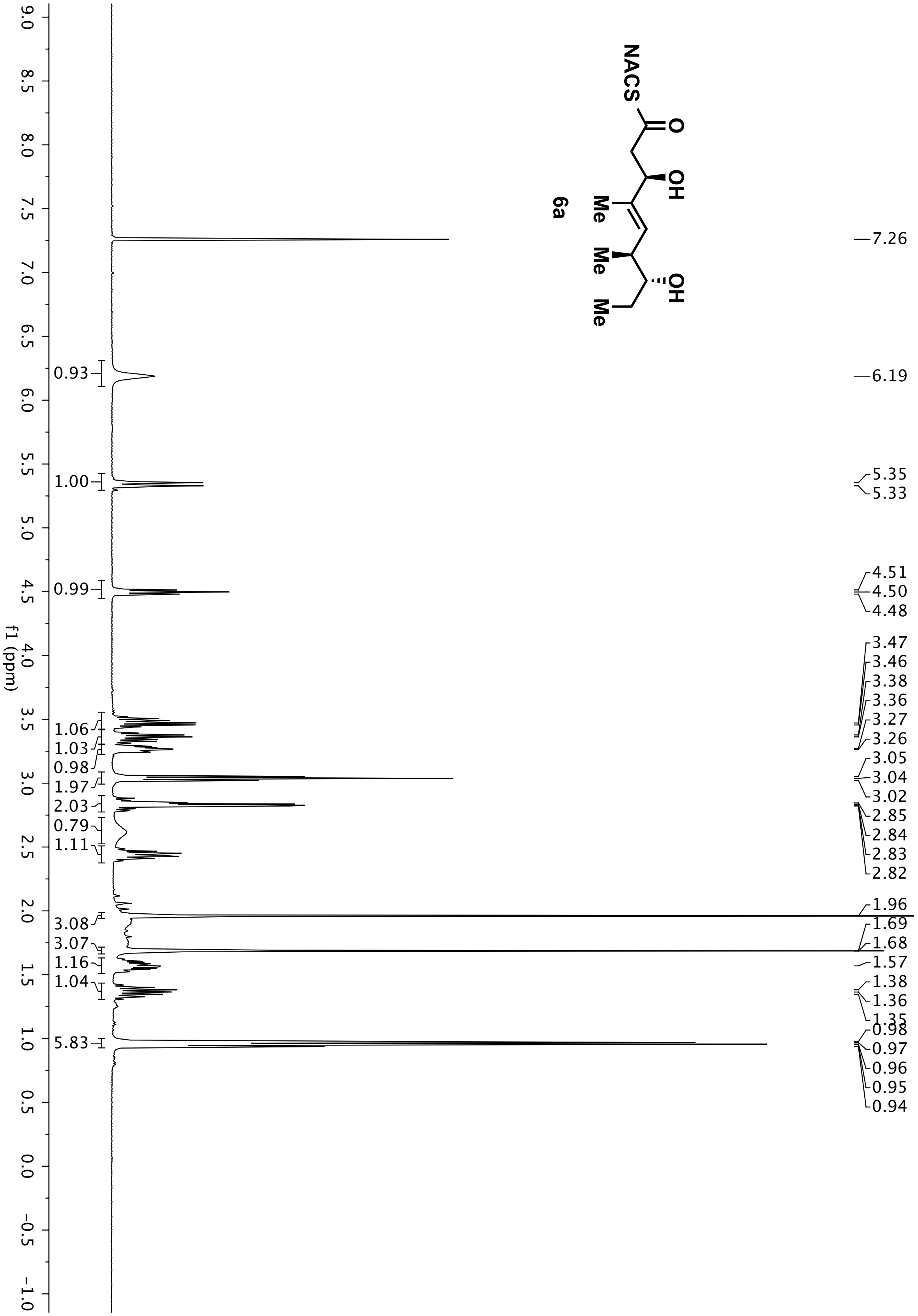
NACS
O
OH
OH
Me
Me
Me
6a
f1 (ppm)
7.26
6.19
5.35
5.33
4.51
4.50
4.48
3.47
3.46
3.38
3.36
3.27
3.26
3.05
3.04
3.02
2.85
2.84
2.83
2.82
1.96
1.69
1.68
1.57
1.38
1.36
1.35
0.98
0.97
0.96
0.95
0.94
0.93
1.00
0.99
1.06
1.03
0.98
1.97
2.03
0.79
1.11
3.08
3.07
1.16
1.04
5.83

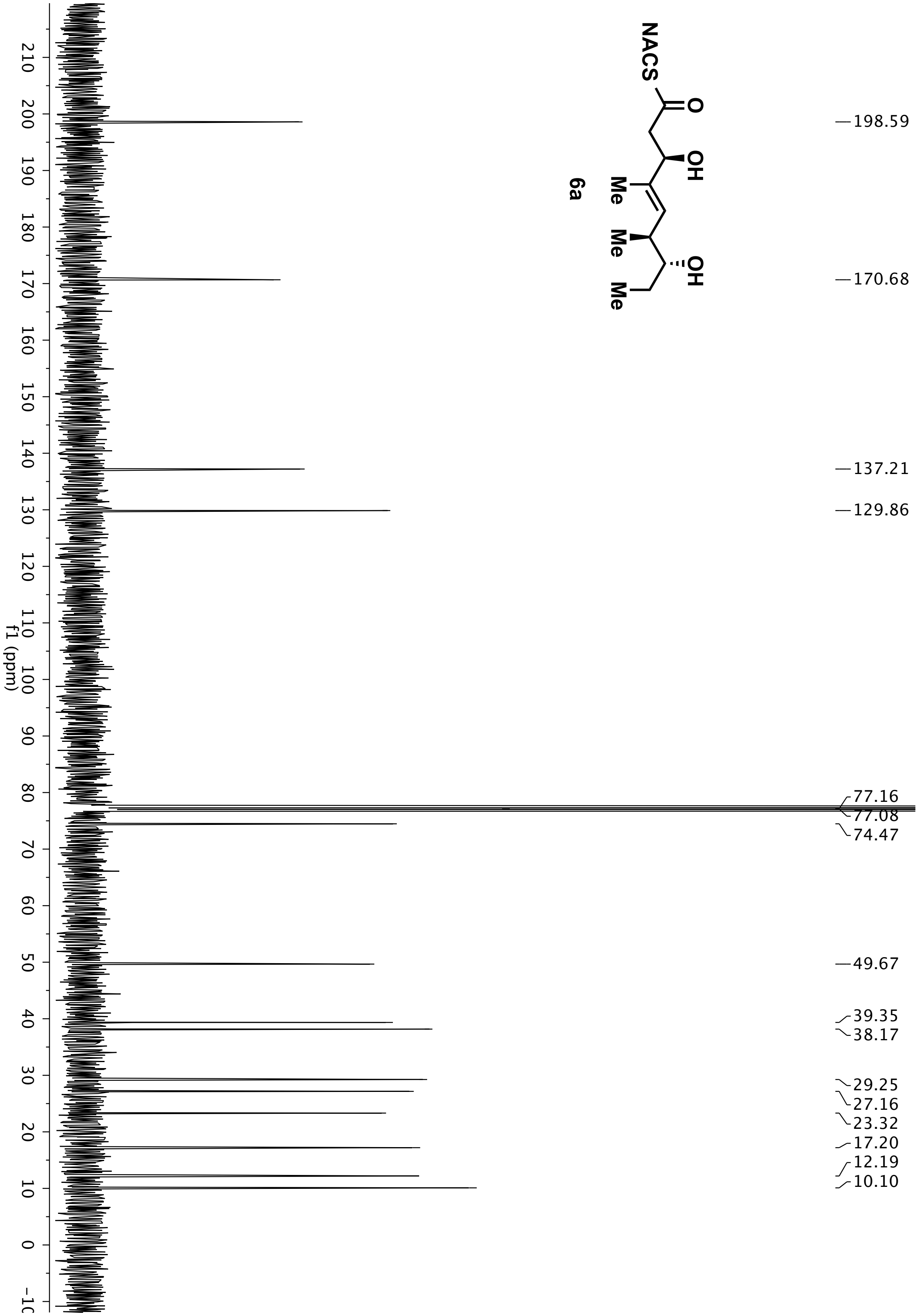
NACS
O
OH
OH
Me
Me
Me
6a
f1 (ppm)
198.59
170.68
137.21
129.86
77.16
77.08
74.47
49.67
39.35
38.17
29.25
27.16
23.32
17.20
12.19
10.10

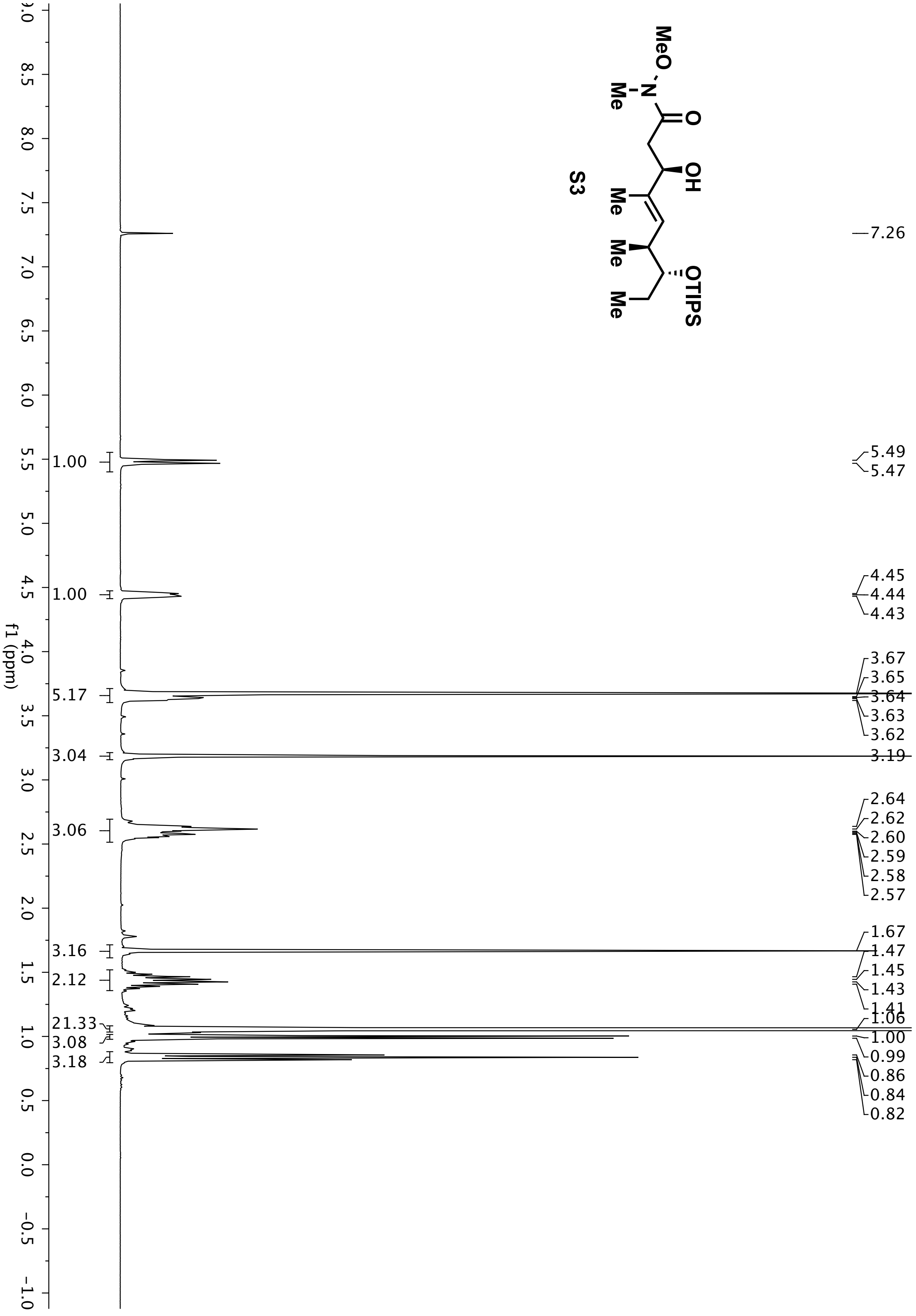
S3
MeO
N
Me
O
OH
Me
Me
OTIPS
Me
7.26
5.49
5.47
4.45
4.44
4.43
3.67
3.65
3.64
3.63
3.62
3.19
2.64
2.62
2.60
2.59
2.58
2.57
1.67
1.47
1.45
1.43
1.41
1.06
1.00
0.99
0.86
0.84
0.82
1.00
1.00
5.17
3.04
3.06
3.16
2.12
21.33
3.08
3.18
f1 (ppm)

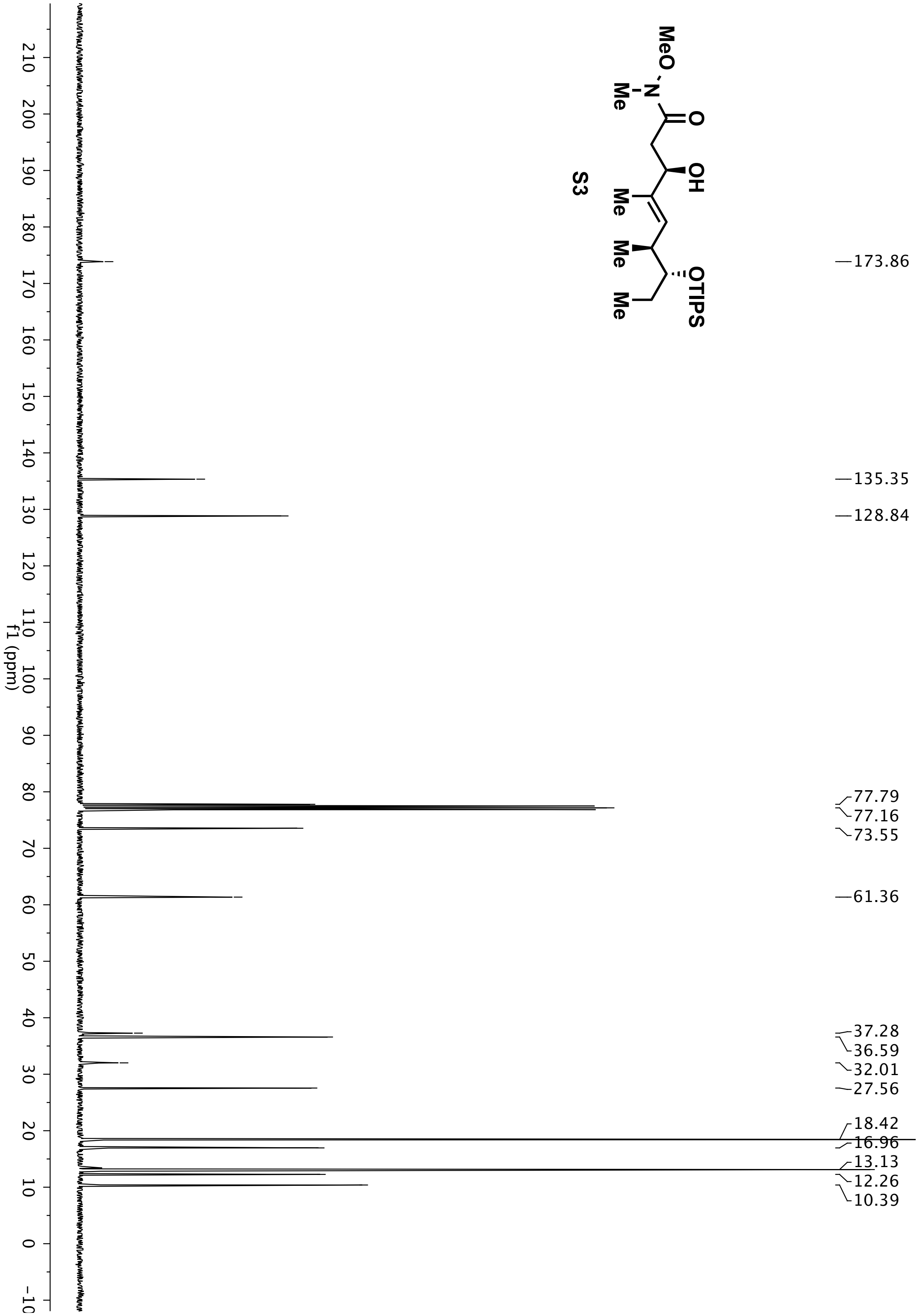
S3
MeO
N
Me
O
OH
Me
Me
OTIPS
Me
173.86
135.35
128.84
77.79
77.16
73.55
61.36
37.28
36.59
32.01
27.56
18.42
16.96
13.13
12.26
10.39
f1 (ppm)

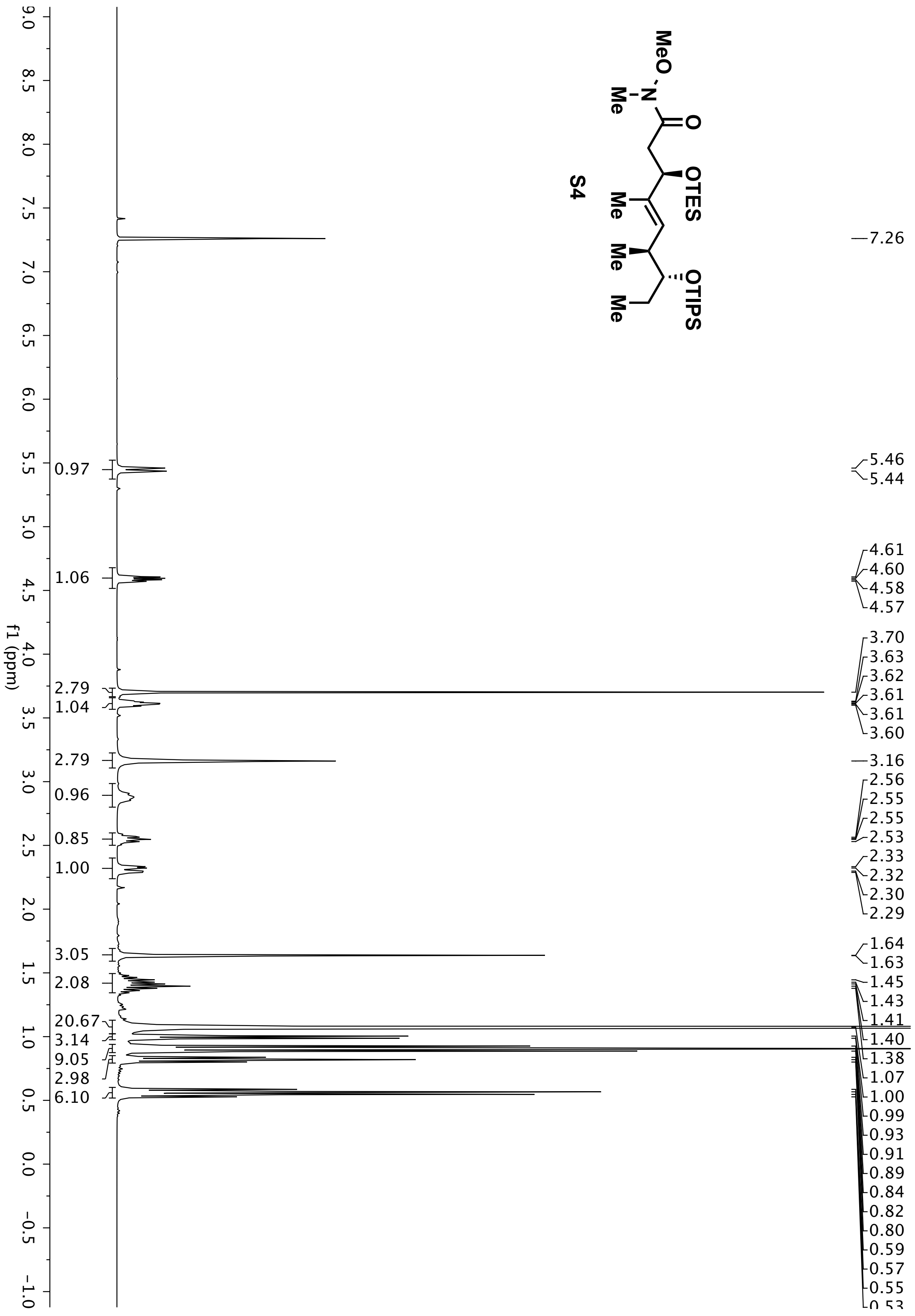
S4
MeO
N
Me
O
OTES
Me
Me
OTIPS
Me
f1 (ppm)

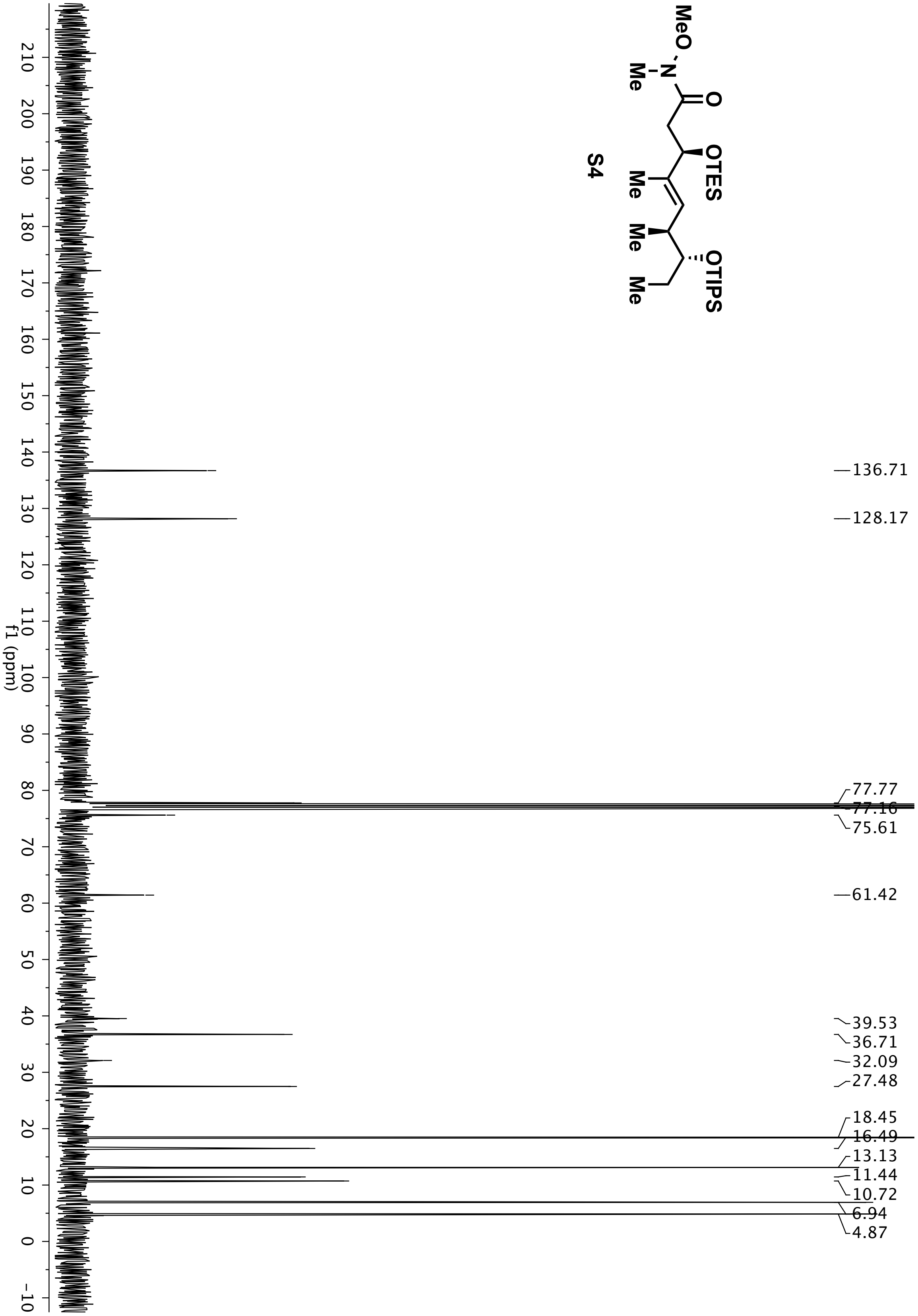
MeO
N
Me
O
OTES
Me
Me
OTIPS
Me
S4
136.71
128.17
77.77
77.16
75.61
61.42
39.53
36.71
32.09
27.48
18.45
16.49
13.13
11.44
10.72
6.94
4.87
f1 (ppm)

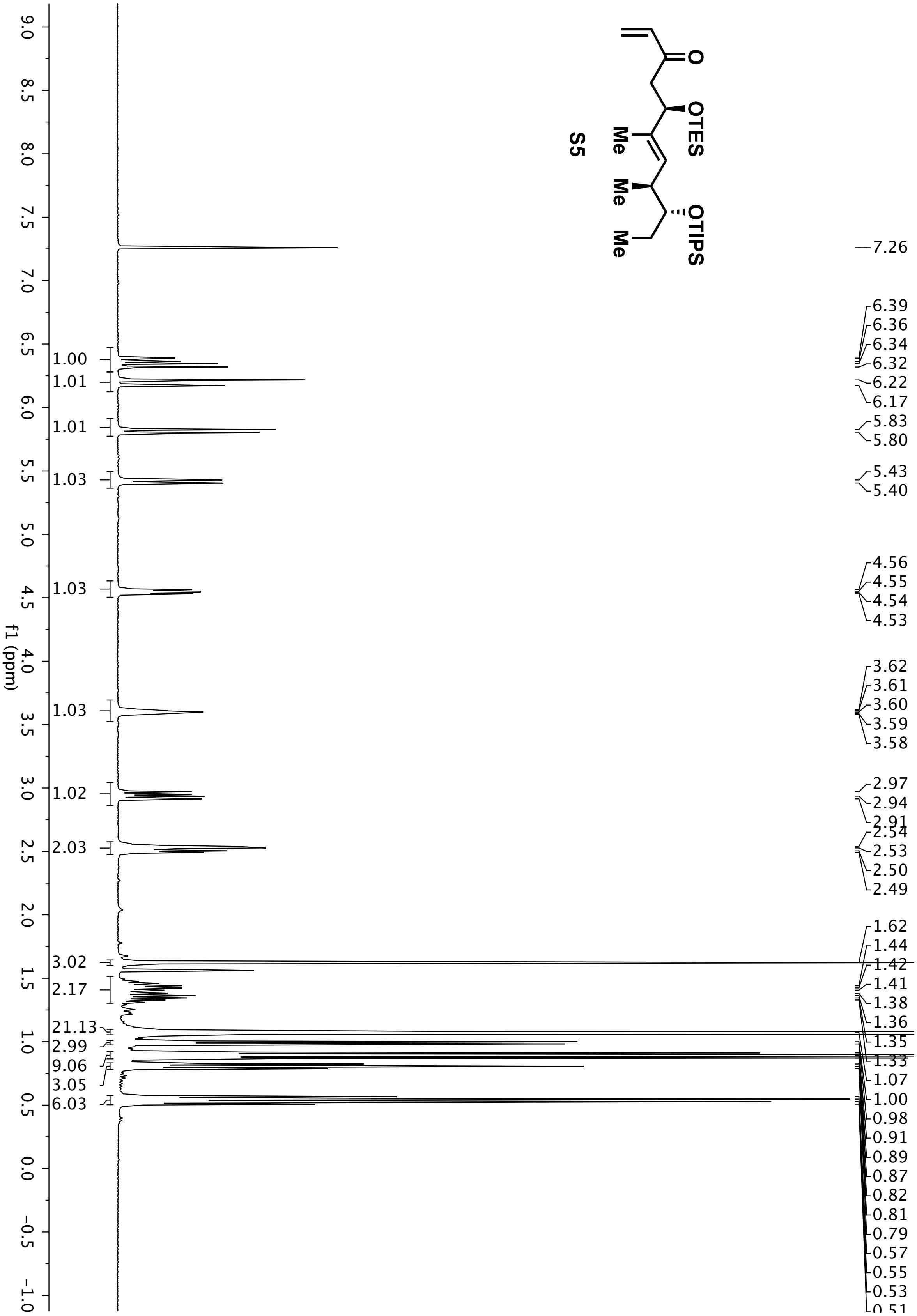
S5
OTES
OTIPS
O
Me
Me
Me
f1 (ppm)
9.0
8.5
8.0
7.5
7.0
6.5
6.0
5.5
5.0
4.5
4.0
3.5
3.0
2.5
2.0
1.5
1.0
0.5
0.0
−0.5
−1.0
1.00
1.01
1.01
1.03
1.03
1.03
1.02
2.03
3.02
2.17
21.13
2.99
9.06
3.05
6.03
7.26
6.39
6.36
6.34
6.32
6.22
6.17
5.83
5.80
5.43
5.40
4.56
4.55
4.54
4.53
3.62
3.61
3.60
3.59
3.58
2.97
2.94
2.91
2.54
2.53
2.50
2.49
1.62
1.44
1.42
1.41
1.38
1.36
1.35
1.33
1.07
1.00
0.98
0.91
0.89
0.87
0.82
0.81
0.79
0.57
0.55
0.53

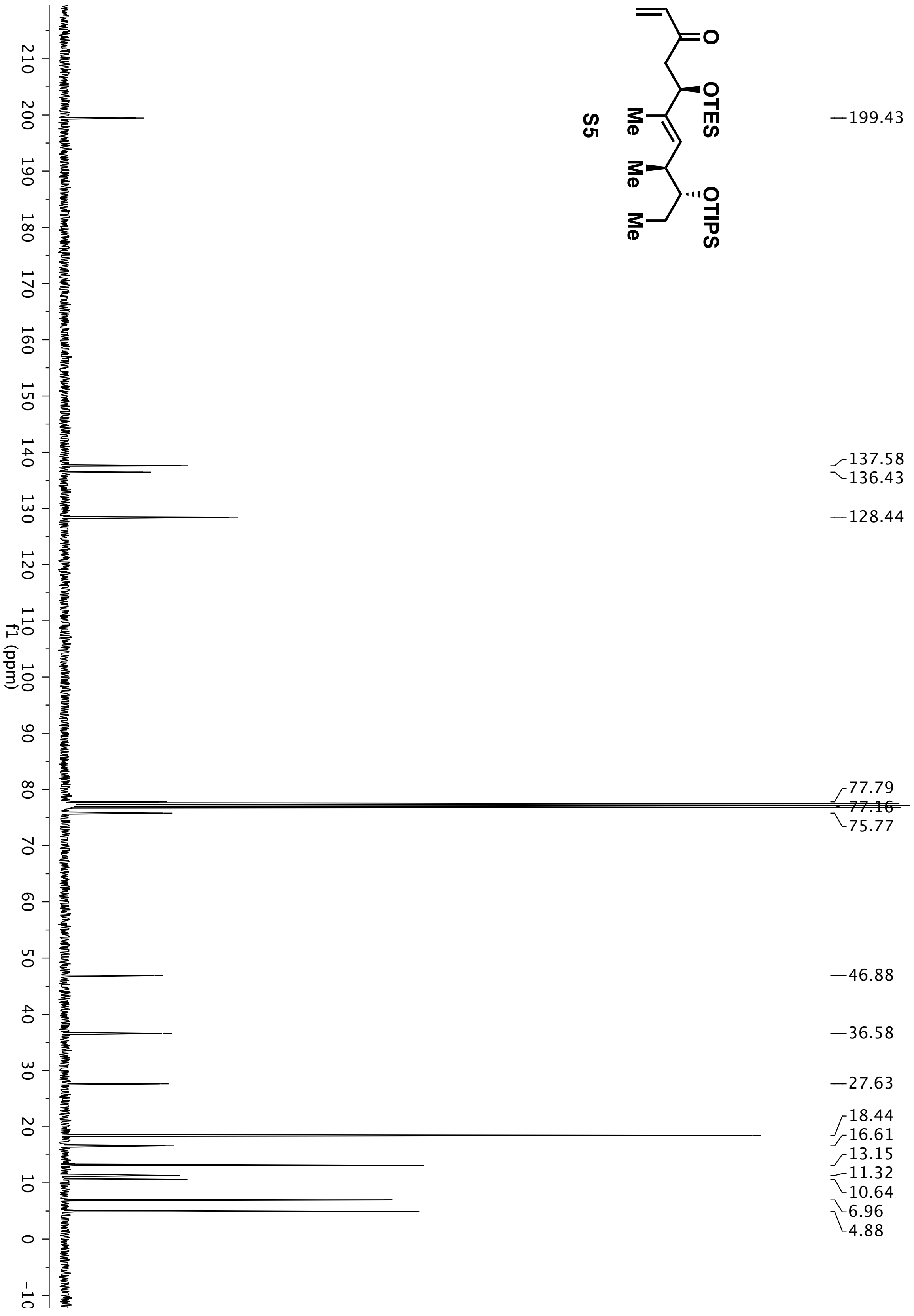

O
OTES
OTIPS
Me
Me
Me
S5
199.43
137.58
136.43
128.44
77.79
77.16
75.77
46.88
36.58
27.63
18.44
16.61
13.15
11.32
10.64
6.96
4.88
f1 (ppm)

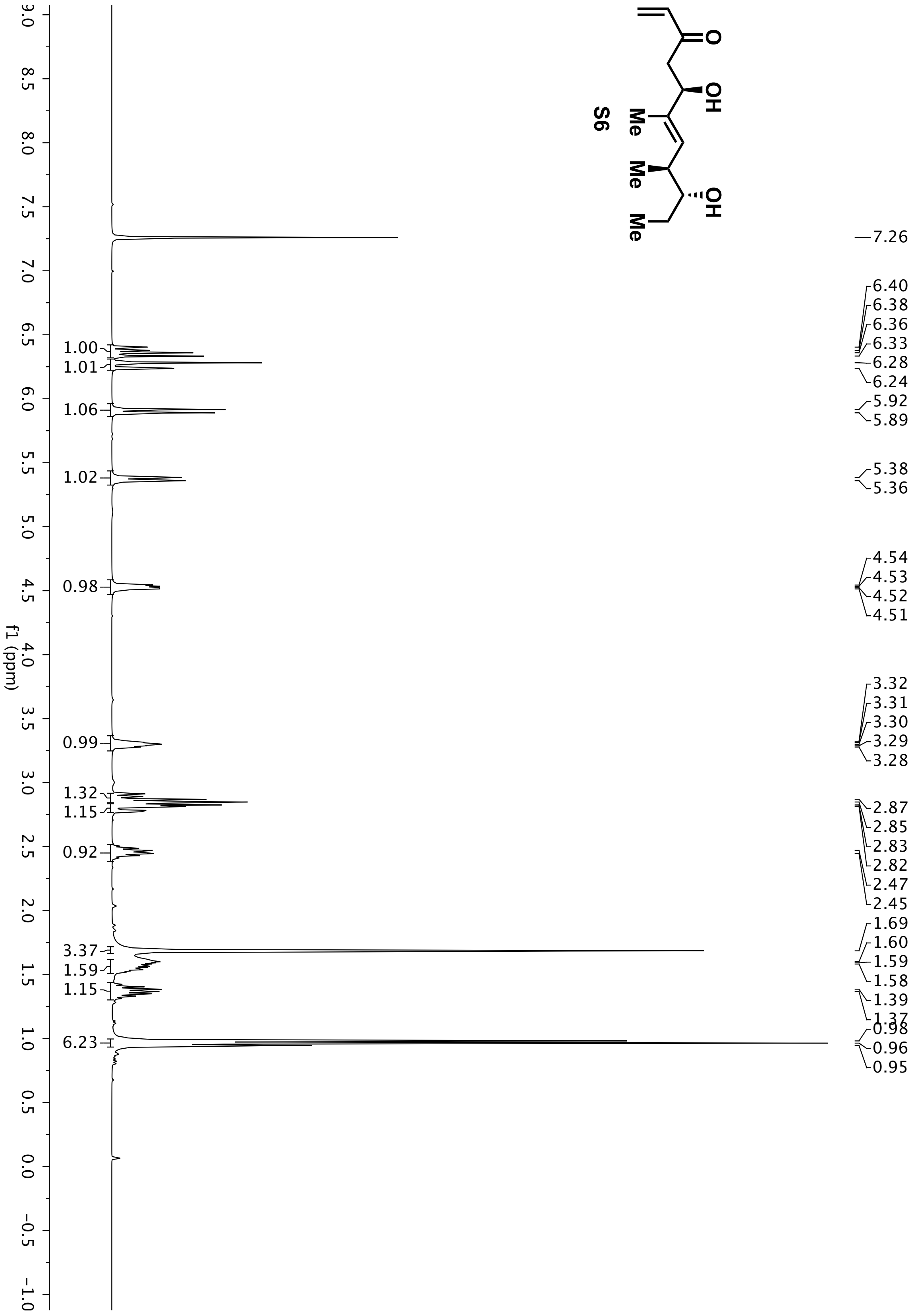
S6
f1 (ppm)
7.26
6.40
6.38
6.36
6.33
6.28
6.24
5.92
5.89
5.38
5.36
4.54
4.53
4.52
4.51
3.32
3.31
3.30
3.29
3.28
2.87
2.85
2.83
2.82
2.47
2.45
1.69
1.60
1.59
1.58
1.39
1.37
0.98
0.96
0.95
1.00
1.01
1.06
1.02
0.98
0.99
1.32
1.15
0.92
3.37
1.59
1.15
6.23

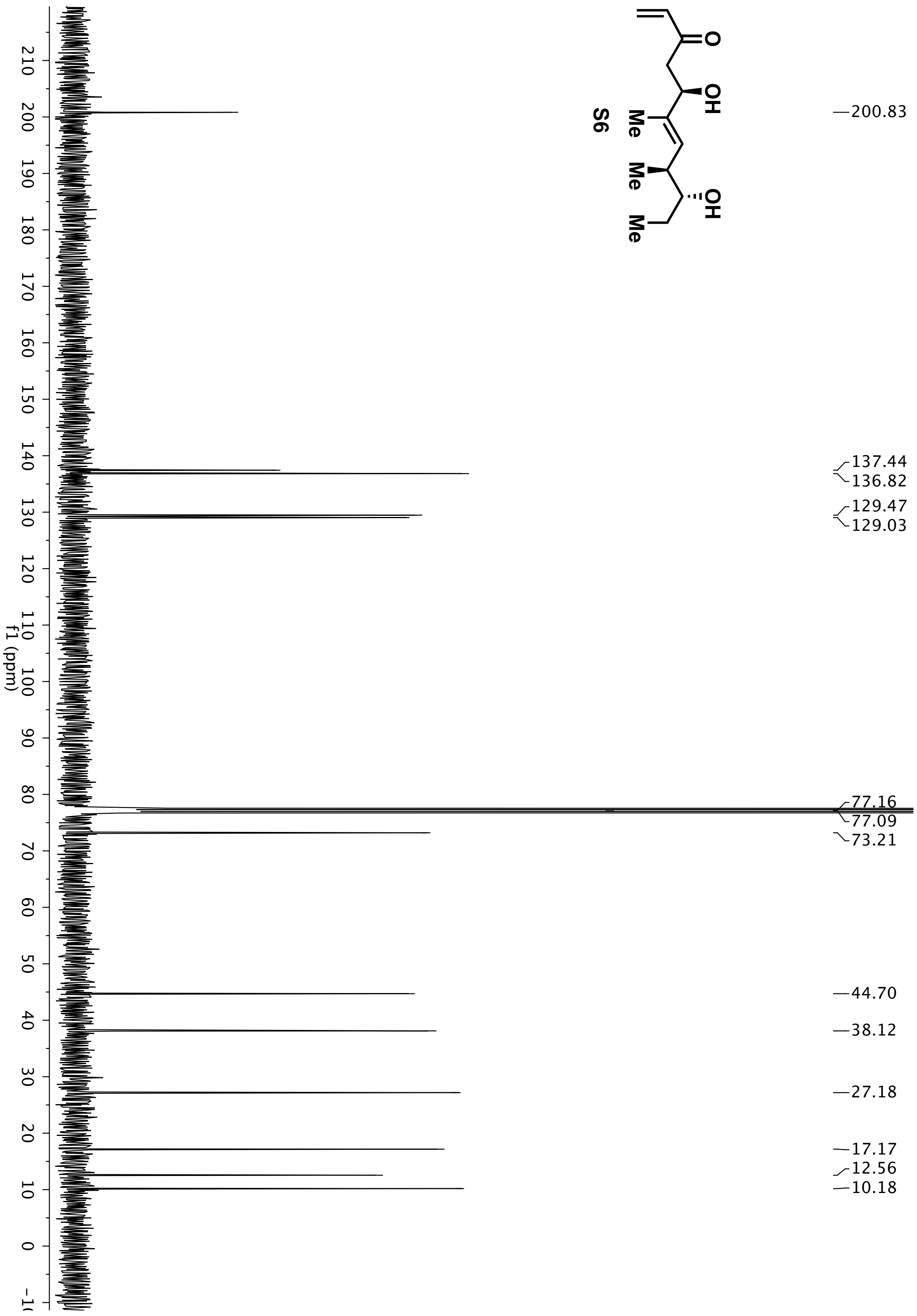
O
OH
OH
Me
Me
Me
S6
200.83
137.44
136.82
129.47
129.03
77.16
77.09
73.21
44.70
38.12
27.18
17.17
12.56
10.18
f1 (ppm)

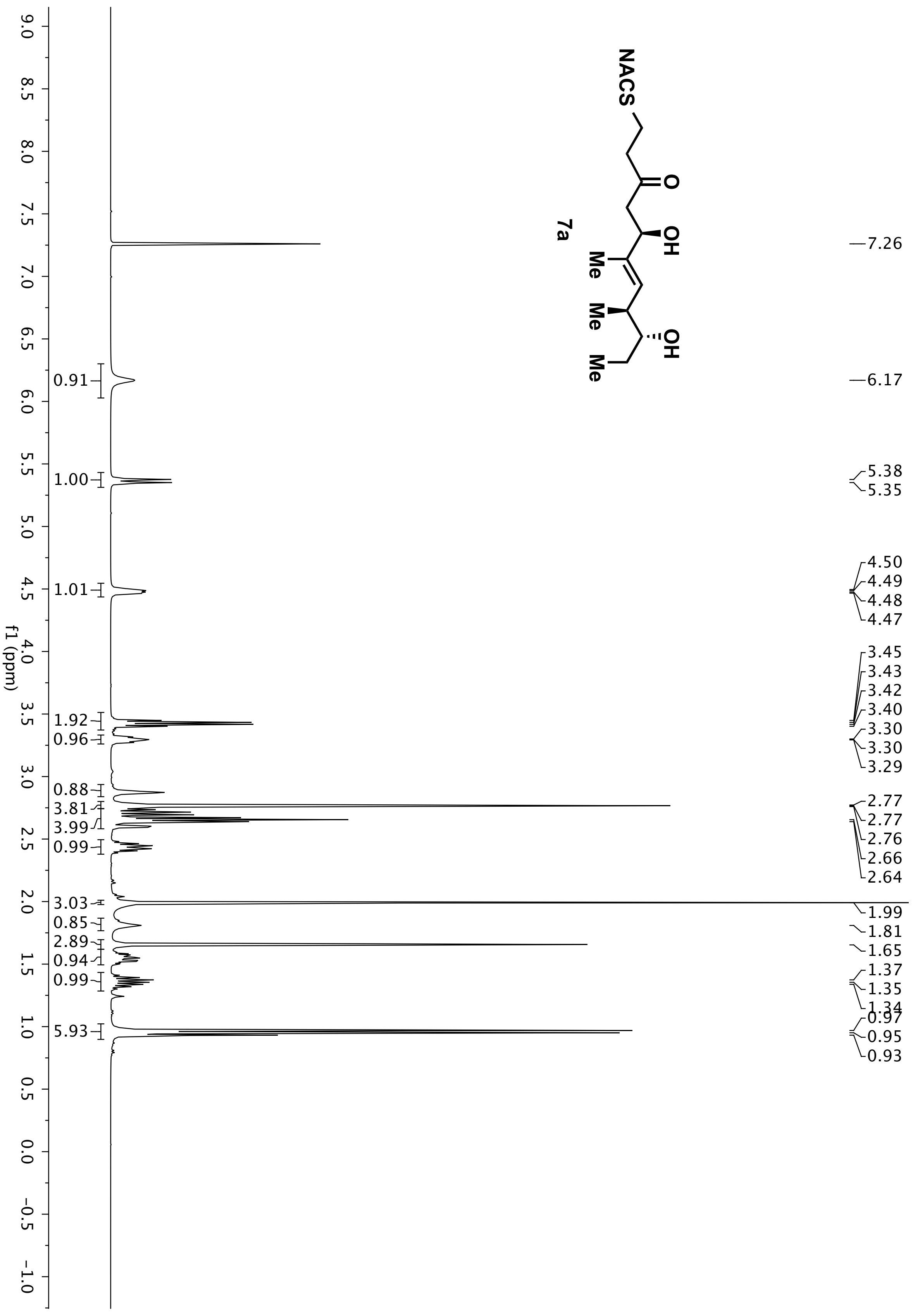
NACS
O
OH
OH
Me
Me
Me
7a
f1 (ppm)
9.0 8.5 8.0 7.5 7.0 6.5 6.0 5.5 5.0 4.5 4.0 3.5 3.0 2.5 2.0 1.5 1.0 0.5 0.0 −0.5 −1.0
0.91
1.00
1.01
1.92
0.96
0.88
3.81
3.99
0.99
3.03
0.85
2.89
0.94
0.99
5.93
7.26
6.17
5.38
5.35
4.50
4.49
4.48
4.47
3.45
3.43
3.42
3.40
3.30
3.30
3.29
2.77
2.77
2.76
2.66
2.64
1.99
1.81
1.65
1.37
1.35
1.34
0.97
0.95
0.93

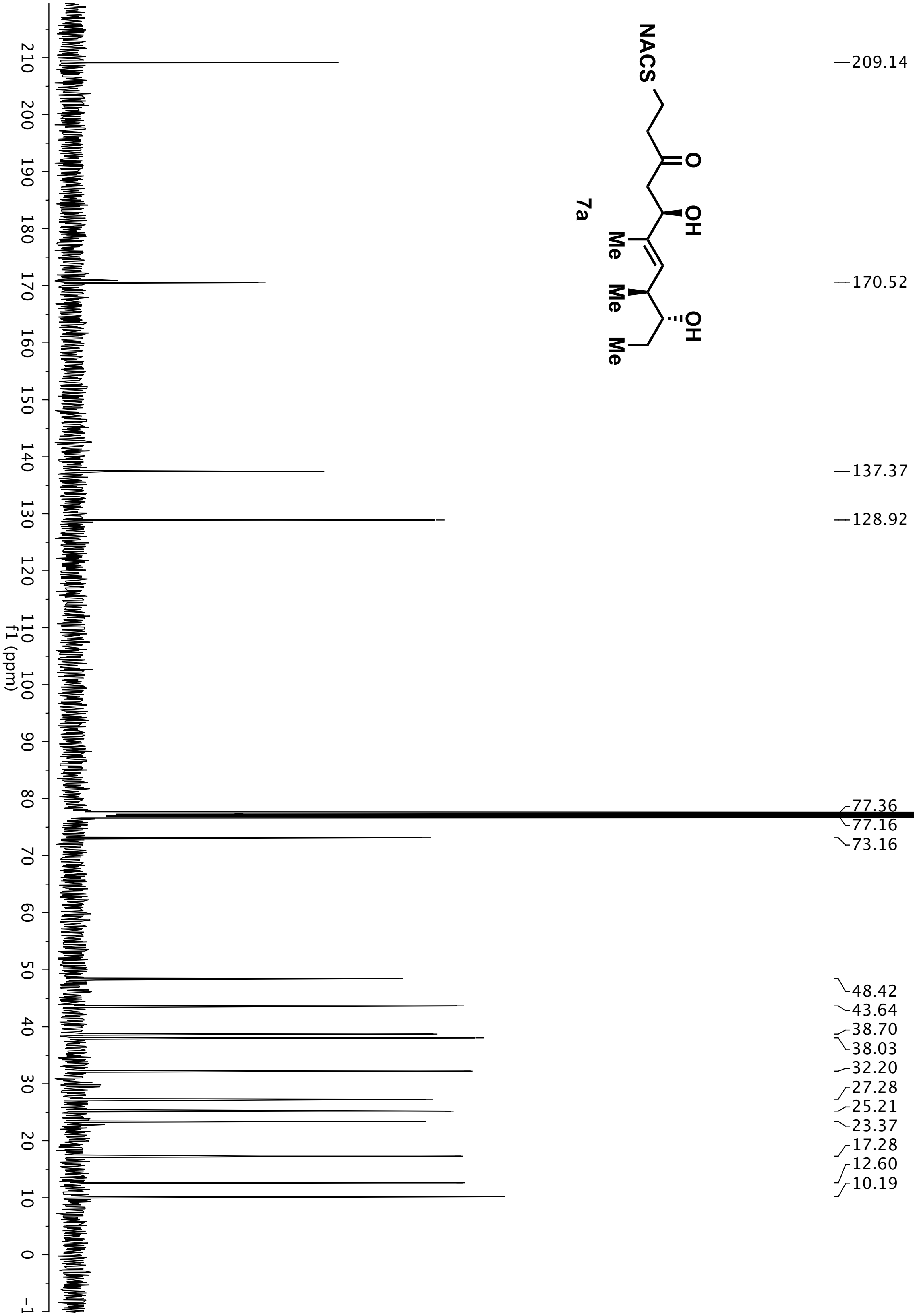
NACS
O
OH
OH
Me
Me
Me
7a
209.14
170.52
137.37
128.92
77.36
77.16
73.16
48.42
43.64
38.70
38.03
32.20
27.28
25.21
23.37
17.28
12.60
10.19
f1 (ppm)
210
200
190
180
170
160
150
140
130
120
110
100
90
80
70
60
50
40
30
20
10
0
−1

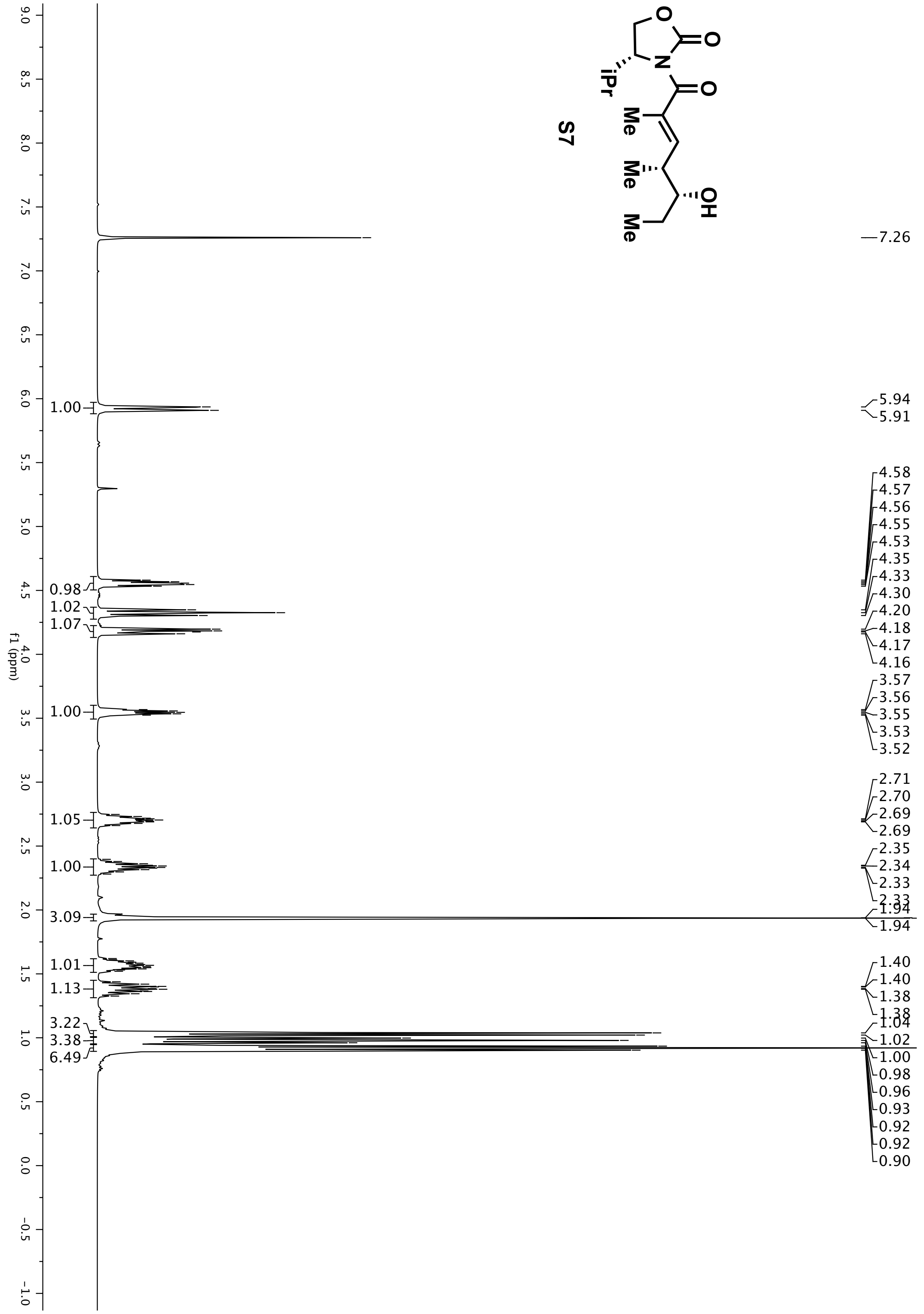
S7
iPr
Me
OH
f1 (ppm)
7.26
5.94
5.91
4.58
4.57
4.56
4.55
4.53
4.35
4.33
4.30
4.20
4.18
4.17
4.16
3.57
3.56
3.55
3.53
3.52
2.71
2.70
2.69
2.69
2.35
2.34
2.33
2.33
1.94
1.94
1.40
1.40
1.38
1.38
1.04
1.02
1.00
0.98
0.96
0.93
0.92
0.92
0.90
1.00
0.98
1.02
1.07
1.00
1.05
1.00
3.09
1.01
1.13
3.22
3.38
6.49

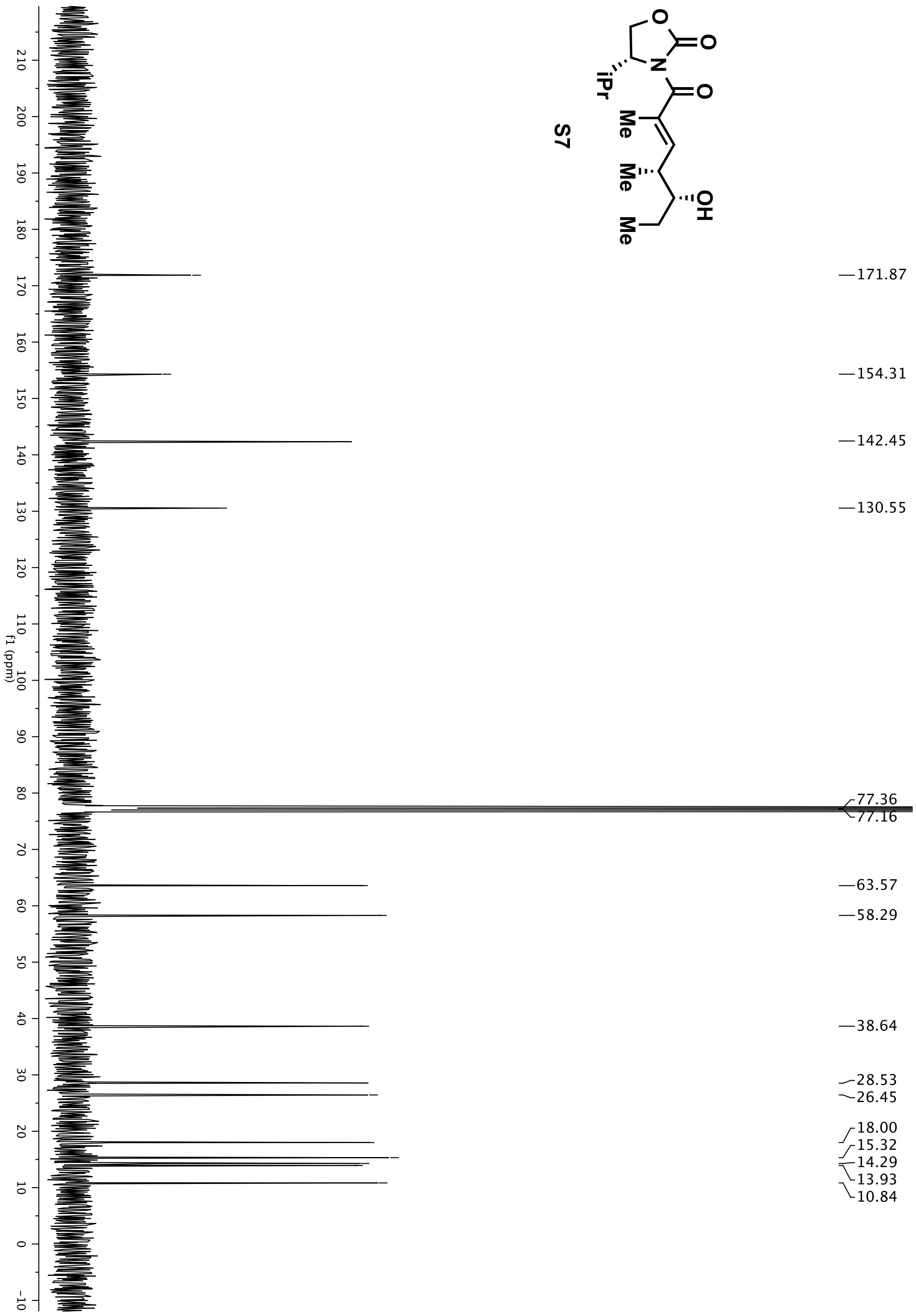
S7
OH
Me
iPr
O
N
171.87
154.31
142.45
130.55
77.36
77.16
63.57
58.29
38.64
28.53
26.45
18.00
15.32
14.29
13.93
10.84
f1 (ppm)

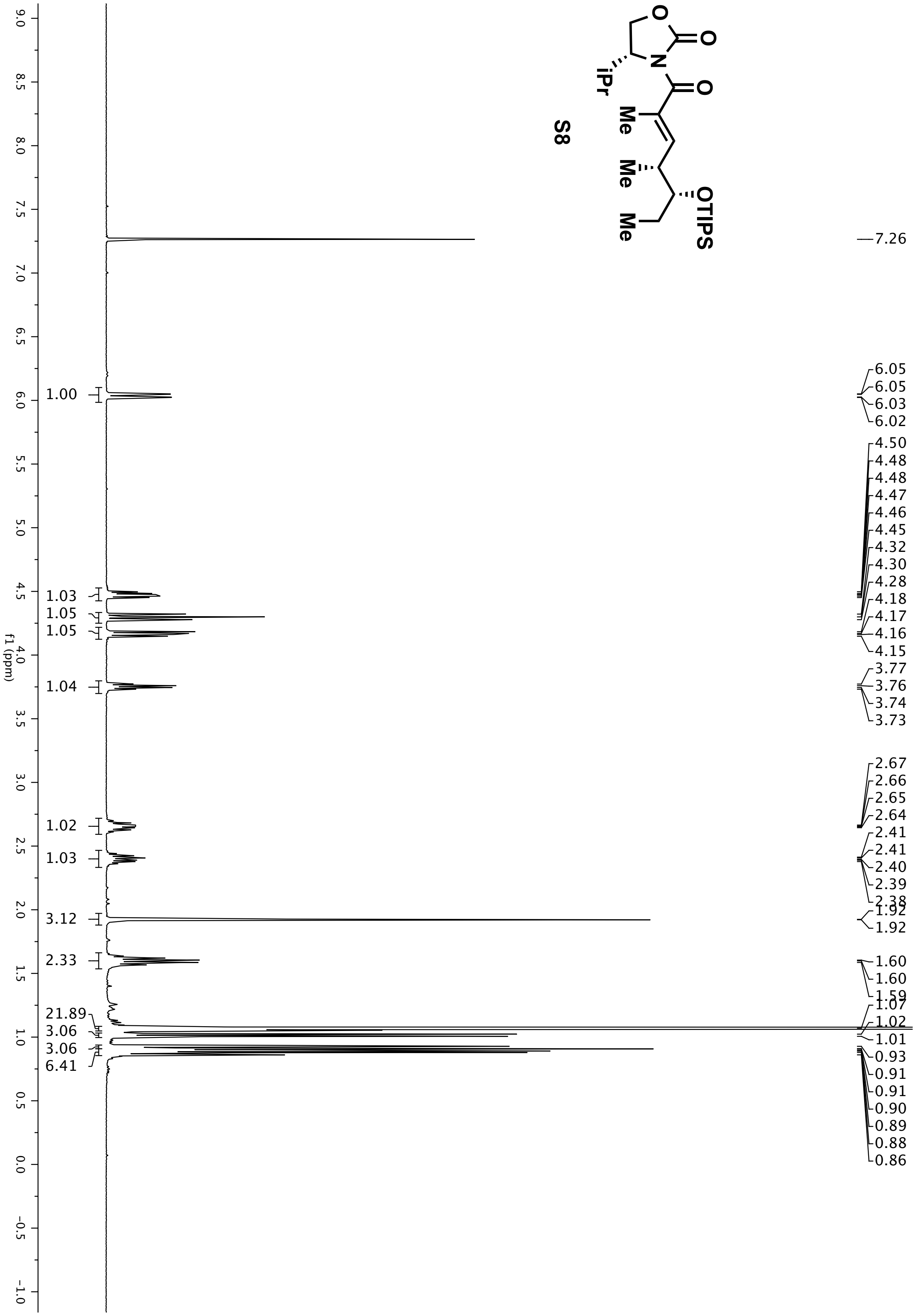
S8

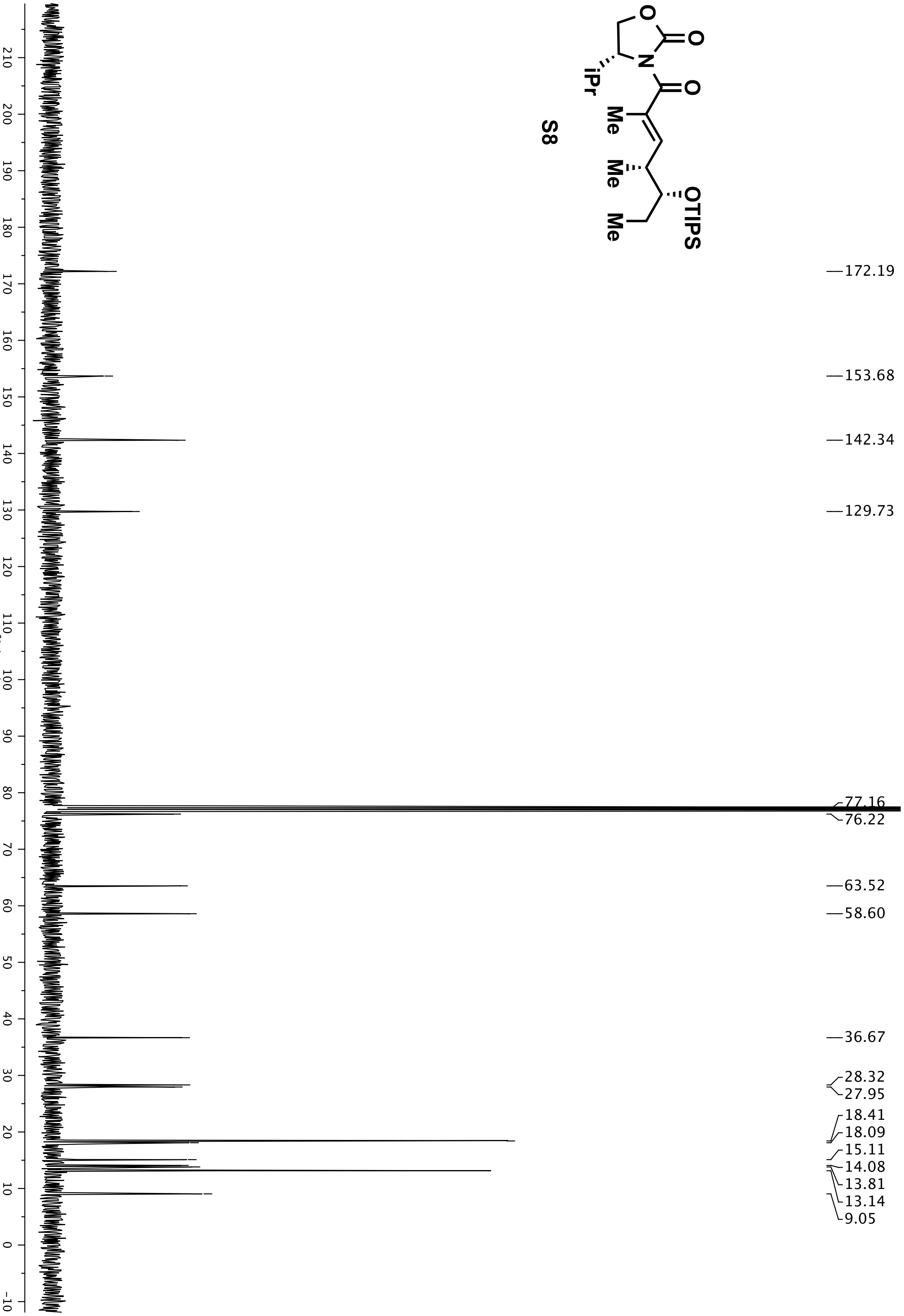

S8
OTIPS
Me
Me
Me
iPr
172.19
153.68
142.34
129.73
77.16
76.22
63.52
58.60
36.67
28.32
27.95
18.41
18.09
15.11
14.08
13.81
13.14
9.05
f1 (ppm)

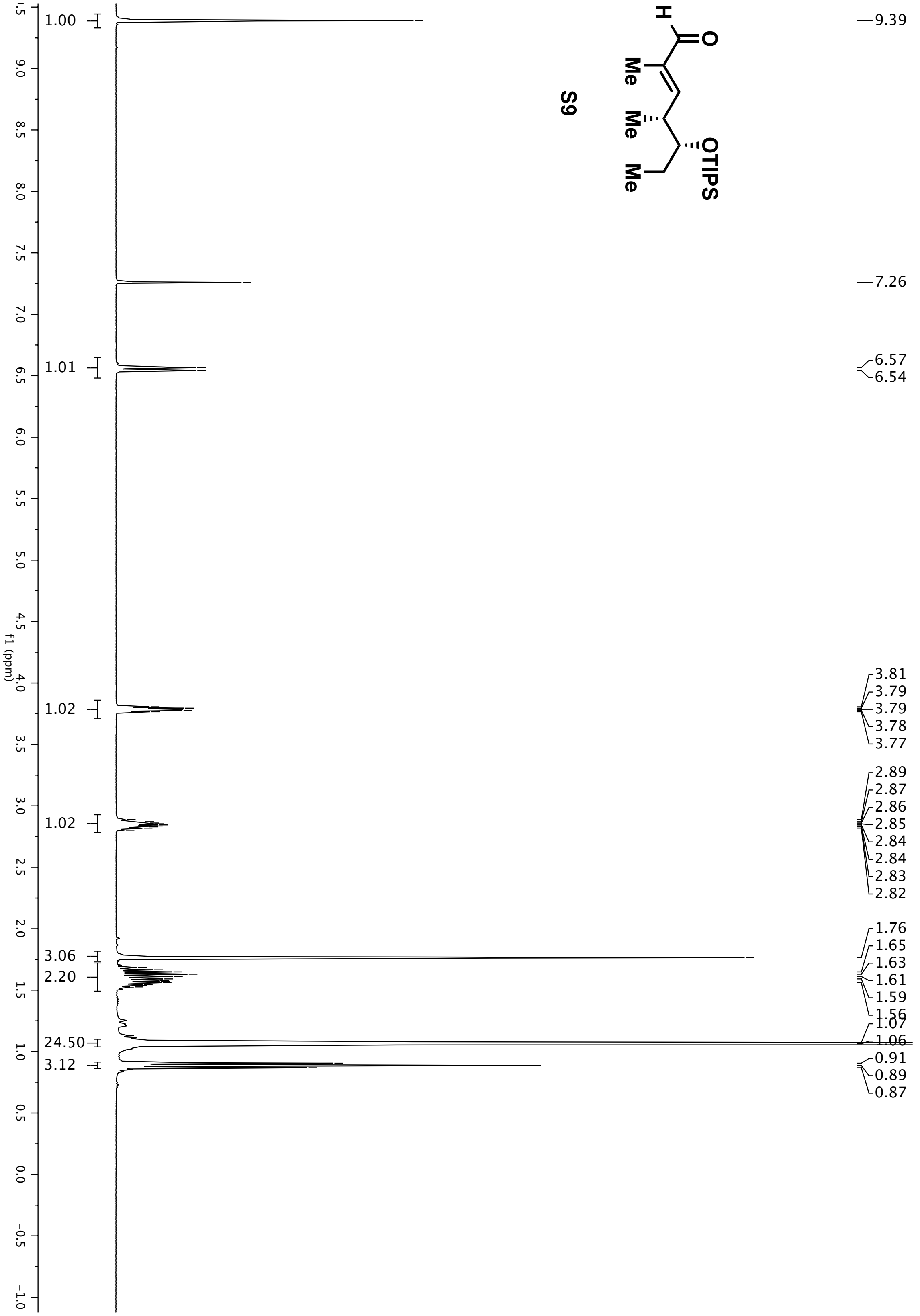

OTIPS
Me
Me
Me
H
O
S9
9.39
7.26
6.57
6.54
3.81
3.79
3.79
3.78
3.77
2.89
2.87
2.86
2.85
2.84
2.84
2.83
2.82
1.76
1.65
1.63
1.61
1.59
1.56
1.07
1.06
0.91
0.89
0.87
1.00
1.01
1.02
1.02
3.06
2.20
24.50
3.12
f1 (ppm)

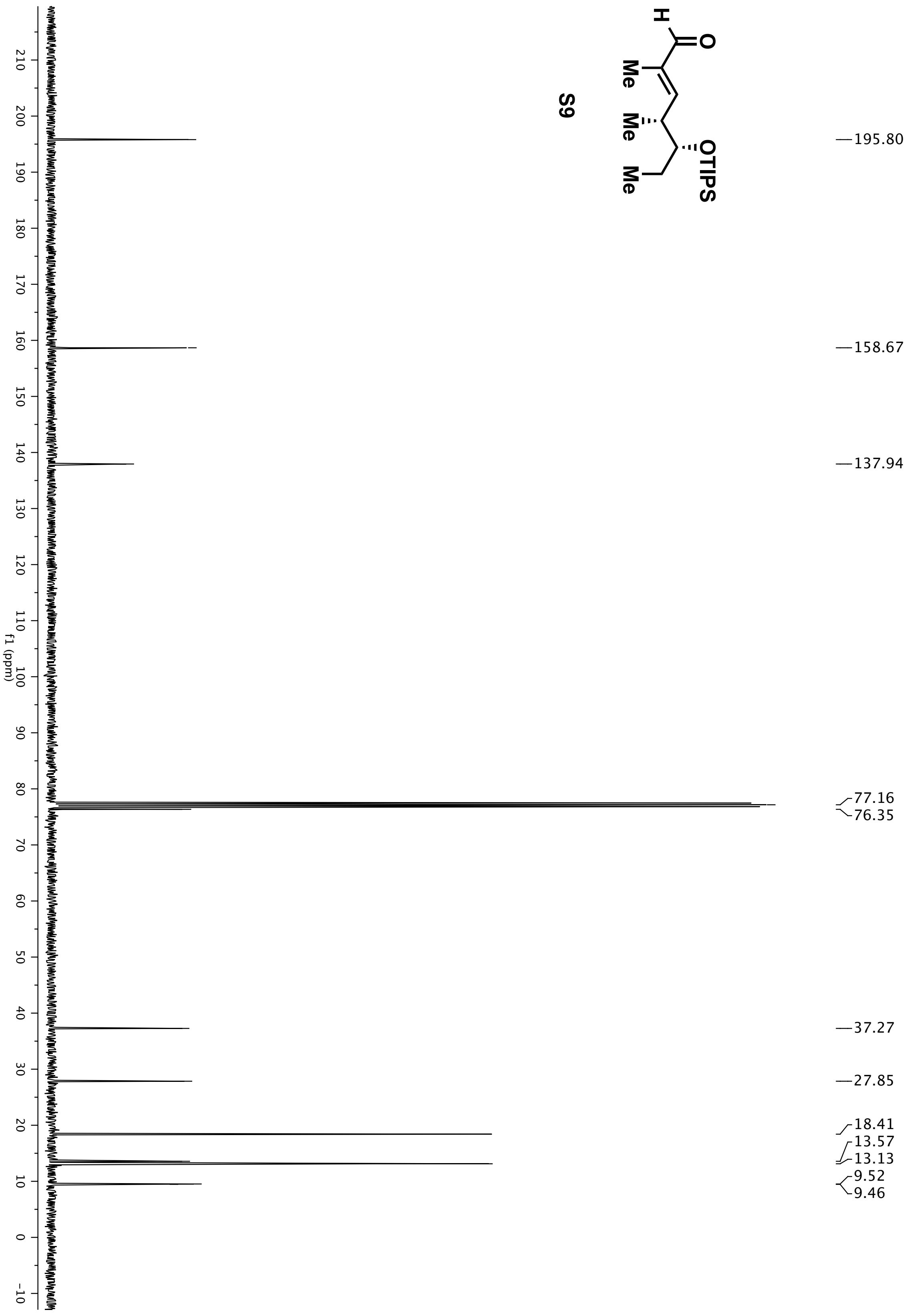
S9
OTIPS
Me
Me
Me
H
O
195.80
158.67
137.94
77.16
76.35
37.27
27.85
18.41
13.57
13.13
9.52
9.46
f1 (ppm)

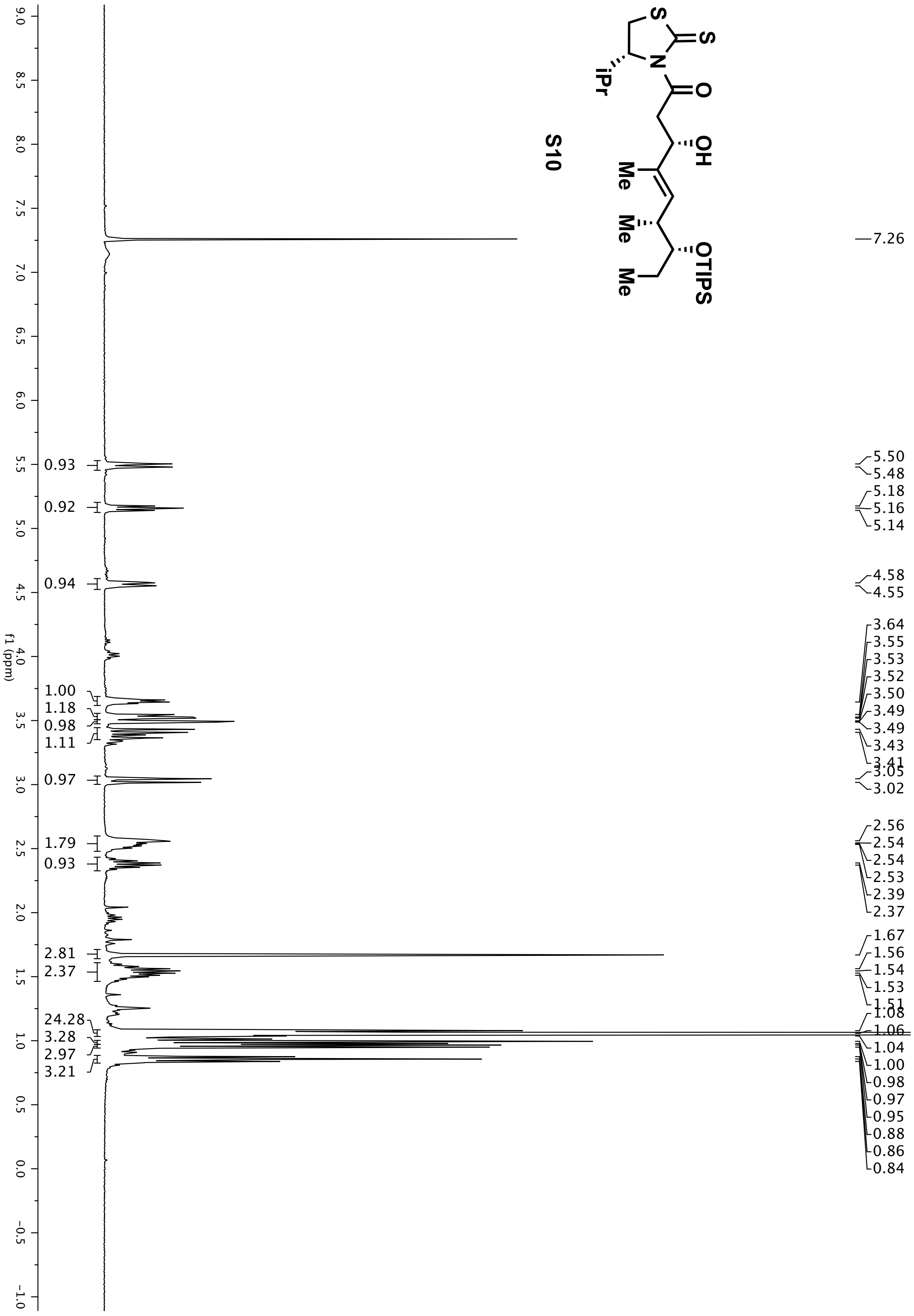
S10
OH
OTIPS
Me
Me
Me
iPr
S
S
N
O
f1 (ppm)
7.26
5.50
5.48
5.18
5.16
5.14
4.58
4.55
3.64
3.55
3.53
3.52
3.50
3.49
3.49
3.43
3.41
3.05
3.02
2.56
2.54
2.54
2.53
2.39
2.37
1.67
1.56
1.54
1.53
1.51
1.08
1.06
1.04
1.00
0.98
0.97
0.95
0.88
0.86
0.84
0.93
0.92
0.94
1.00
1.18
0.98
1.11
0.97
1.79
0.93
2.81
2.37
24.28
3.28
2.97
3.21

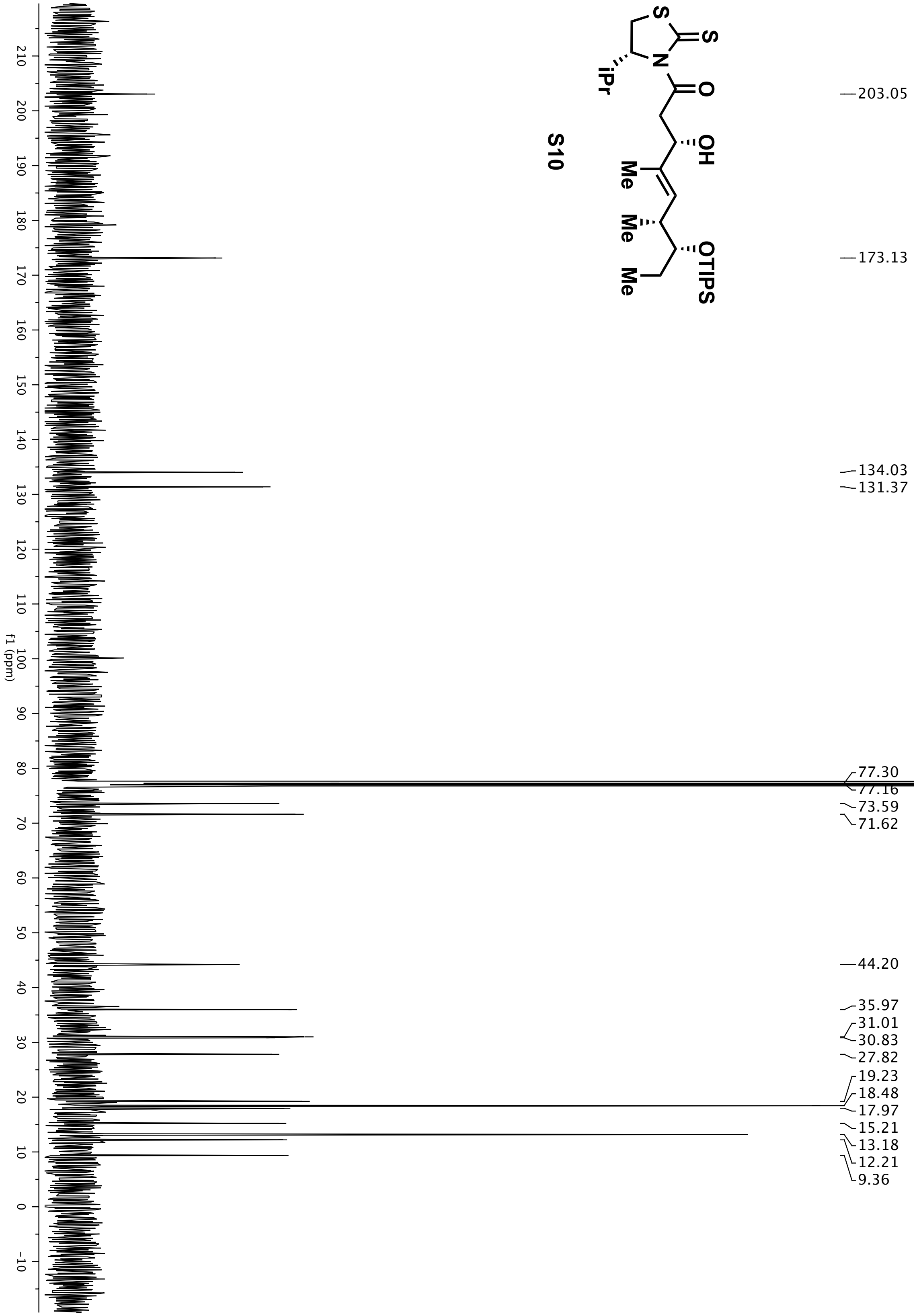
S10
S
S
N
O
OH
OTIPS
iPr
Me
Me
Me
203.05
173.13
134.03
131.37
77.30
77.16
73.59
71.62
44.20
35.97
31.01
30.83
27.82
19.23
18.48
17.97
15.21
13.18
12.21
9.36
f1 (ppm)

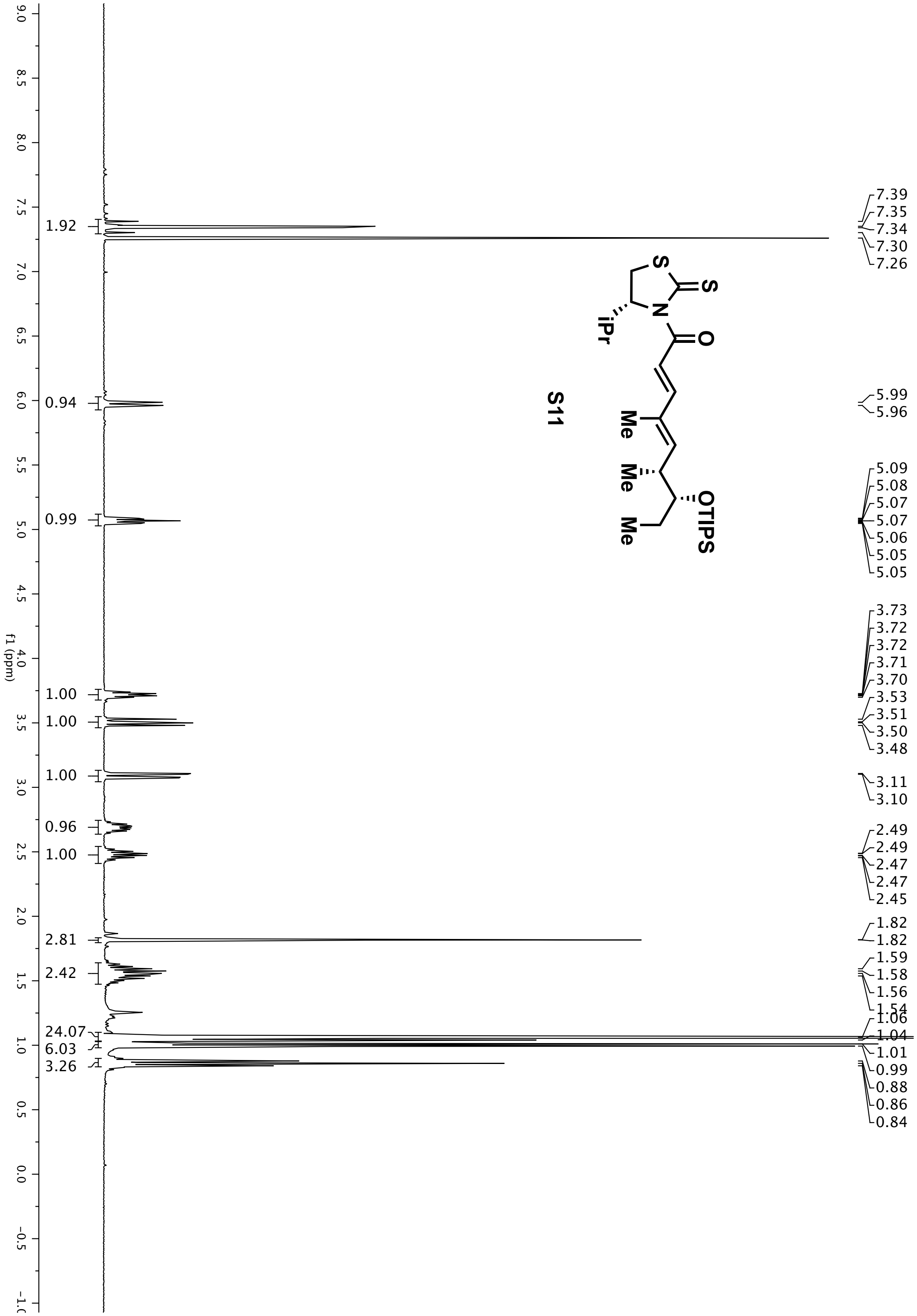
S11
iPr
Me
Me
Me
OTIPS
f1 (ppm)
9.0 8.5 8.0 7.5 7.0 6.5 6.0 5.5 5.0 4.5 4.0 3.5 3.0 2.5 2.0 1.5 1.0 0.5 0.0 -0.5
1.92
0.94
0.99
1.00
1.00
1.00
0.96
1.00
2.81
2.42
24.07
6.03
3.26
7.39 7.35 7.34 7.30 7.26
5.99 5.96
5.09 5.08 5.07 5.07 5.06 5.05 5.05
3.73 3.72 3.72 3.71 3.70 3.53 3.51 3.50 3.48
3.11 3.10
2.49 2.49 2.47 2.47 2.45
1.82 1.82
1.59 1.58 1.56 1.54
1.06 1.04 1.01 0.99 0.88 0.86 0.84

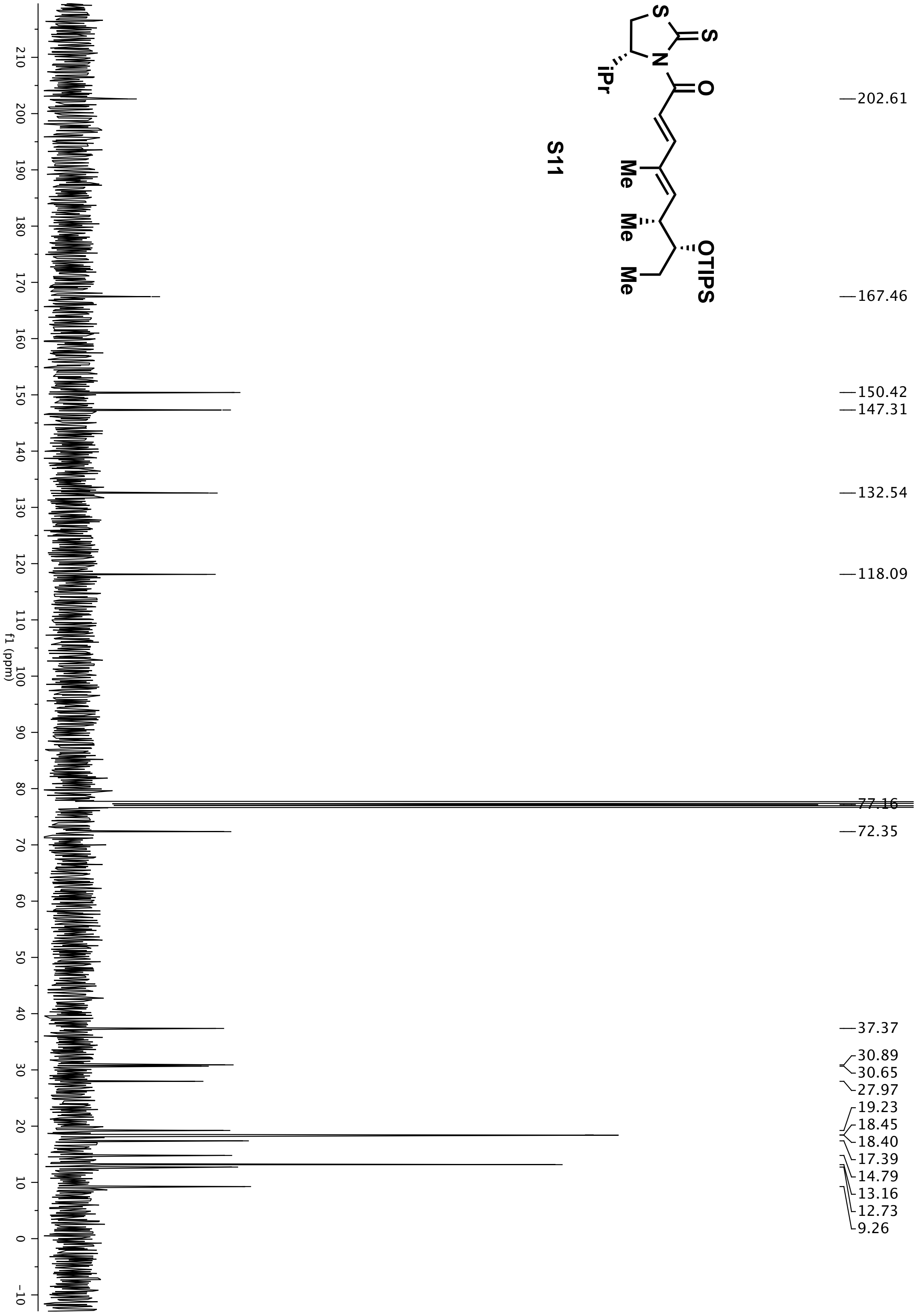
S11
iPr
OTIPS
Me
Me
Me
f1 (ppm)
202.61
167.46
150.42
147.31
132.54
118.09
77.16
72.35
37.37
30.89
30.65
27.97
19.23
18.45
18.40
17.39
14.79
13.16
12.73
9.26

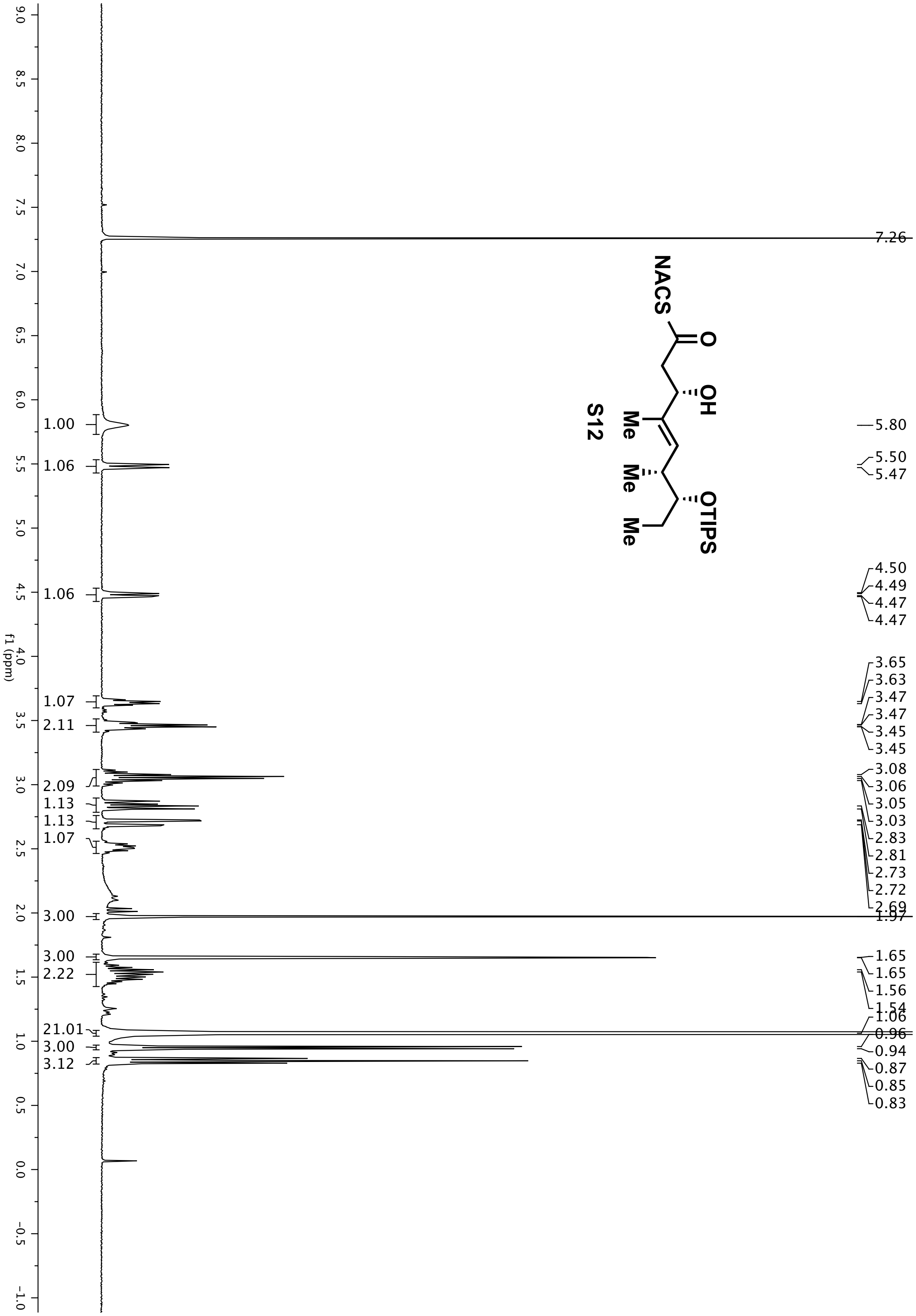
NACS
O
OH
OTIPS
Me
Me
Me
S12
f1 (ppm)
7.26
5.80
5.50
5.47
4.50
4.49
4.47
4.47
3.65
3.63
3.47
3.47
3.45
3.45
3.08
3.06
3.05
3.03
2.83
2.81
2.73
2.72
2.69
1.97
1.65
1.65
1.56
1.54
1.06
0.96
0.94
0.87
0.85
0.83
1.00
1.06
1.06
1.07
2.11
2.09
1.13
1.13
1.07
3.00
3.00
2.22
21.01
3.00
3.12

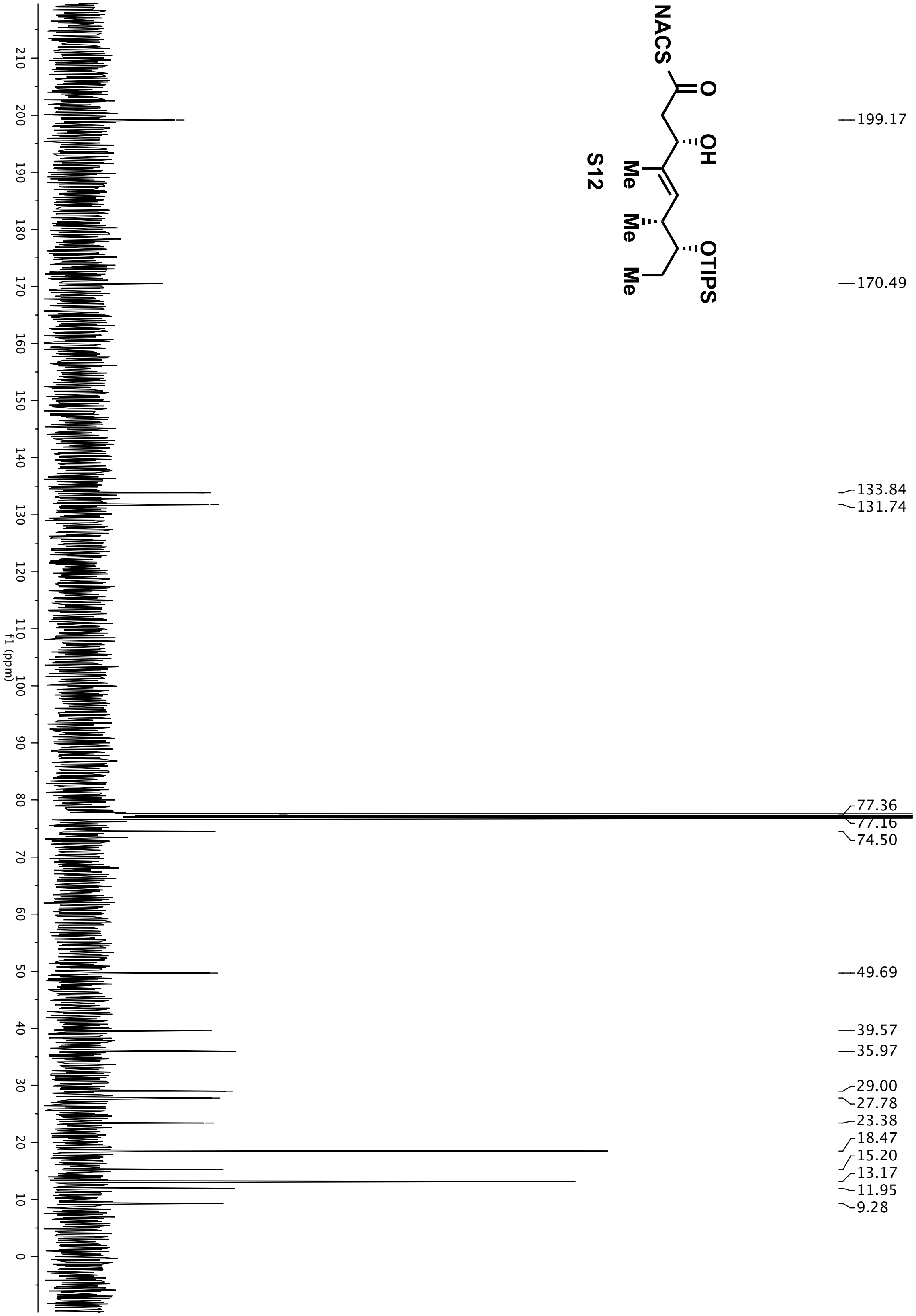

NACS
O
OH
OTIPS
Me
Me
Me
S12
199.17
170.49
133.84
131.74
77.36
77.16
74.50
49.69
39.57
35.97
29.00
27.78
23.38
18.47
15.20
13.17
11.95
9.28
f1 (ppm)

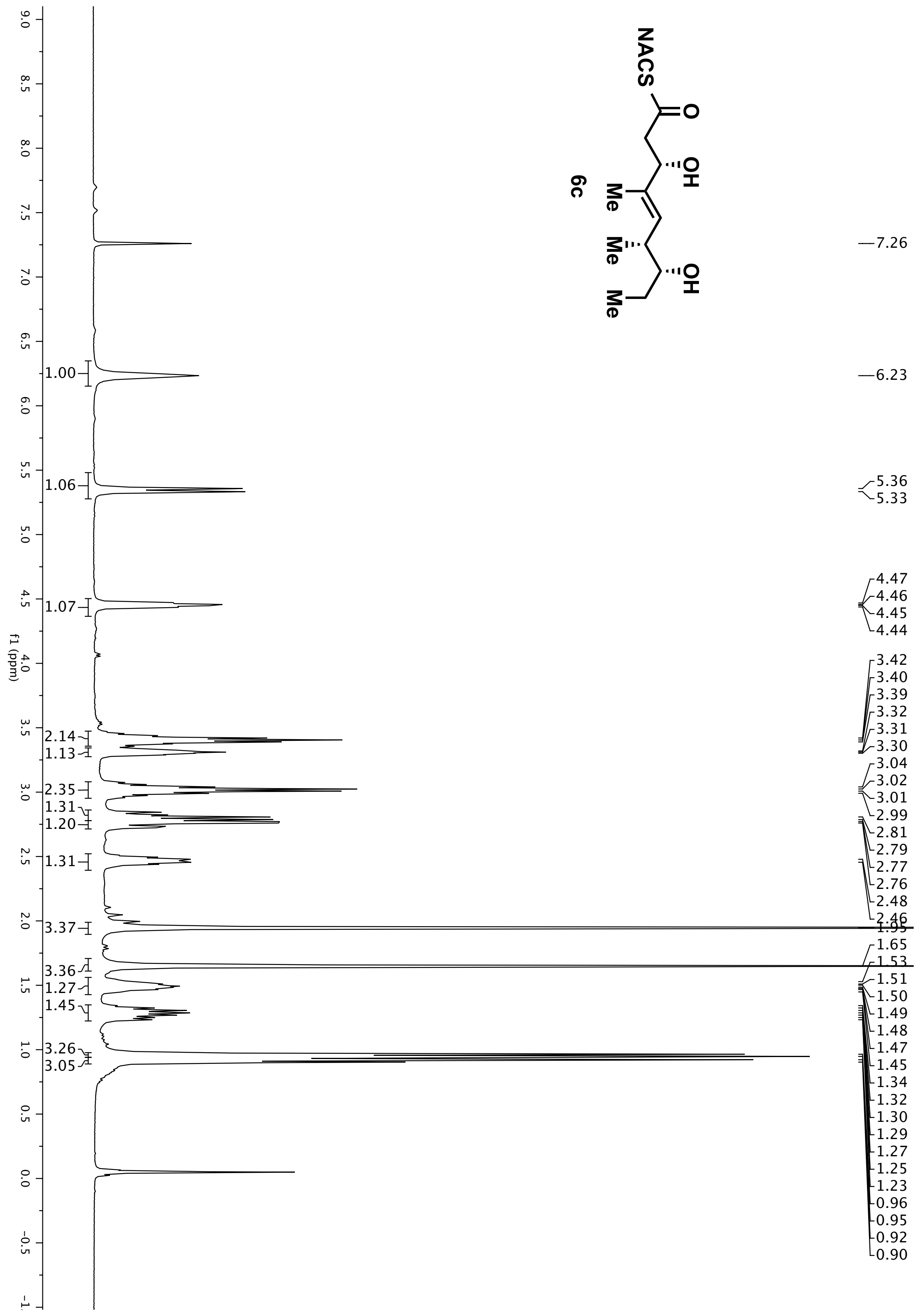
NACS
O
OH
OH
Me
Me
Me
6c
7.26
6.23
5.36
5.33
4.47
4.46
4.45
4.44
3.42
3.40
3.39
3.32
3.31
3.30
3.04
3.02
3.01
2.99
2.81
2.79
2.77
2.76
2.48
2.46
1.95
1.65
1.53
1.51
1.50
1.49
1.48
1.47
1.45
1.34
1.32
1.30
1.29
1.27
1.25
1.23
0.96
0.95
0.92
0.90
1.00
1.06
1.07
2.14
1.13
2.35
1.31
1.20
1.31
3.37
3.36
1.27
1.45
3.26
3.05
f1 (ppm)

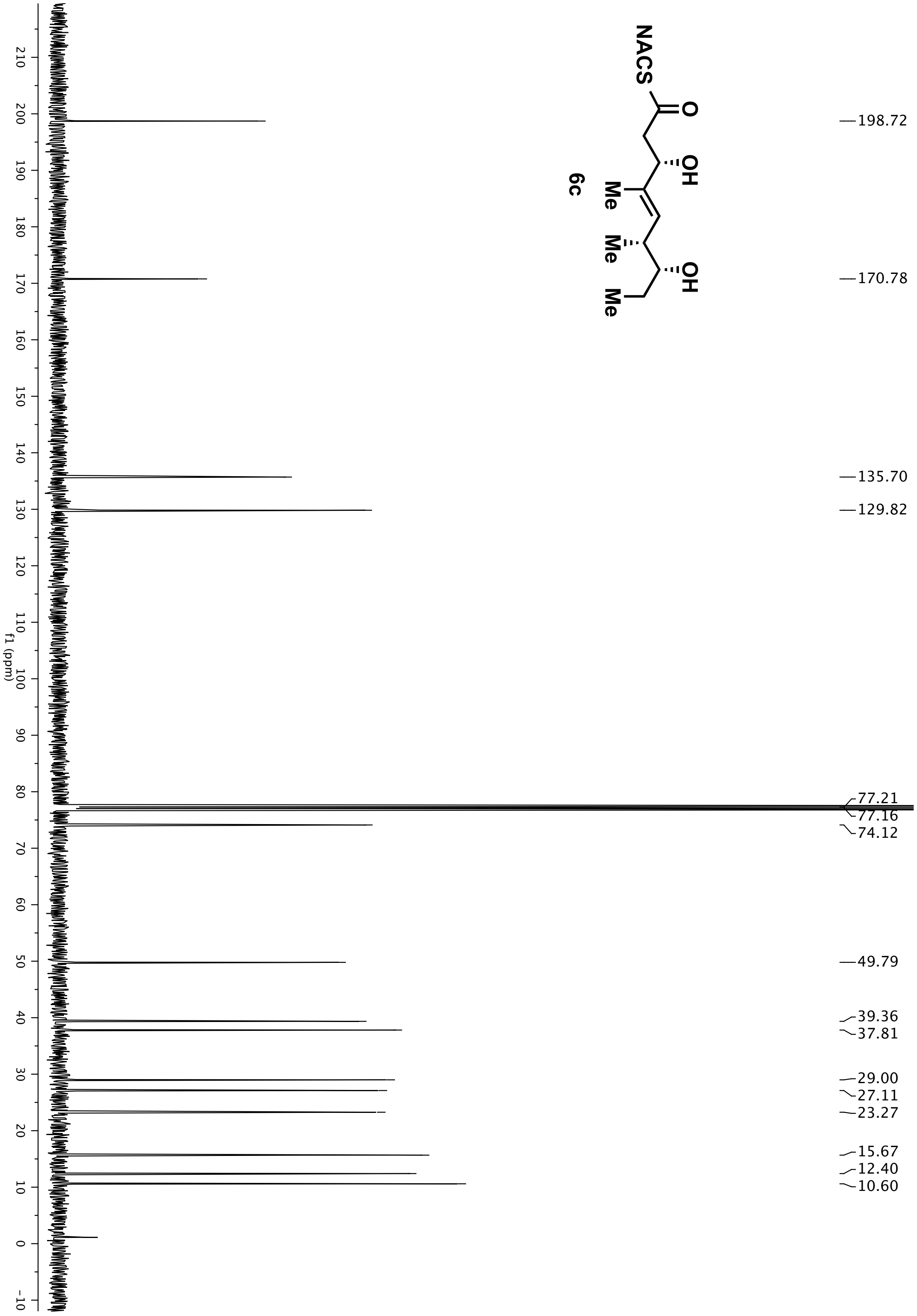
NACS
O
OH
Me
OH
Me
Me
6c
198.72
170.78
135.70
129.82
77.21
77.16
74.12
49.79
39.36
37.81
29.00
27.11
23.27
15.67
12.40
10.60
f1 (ppm)

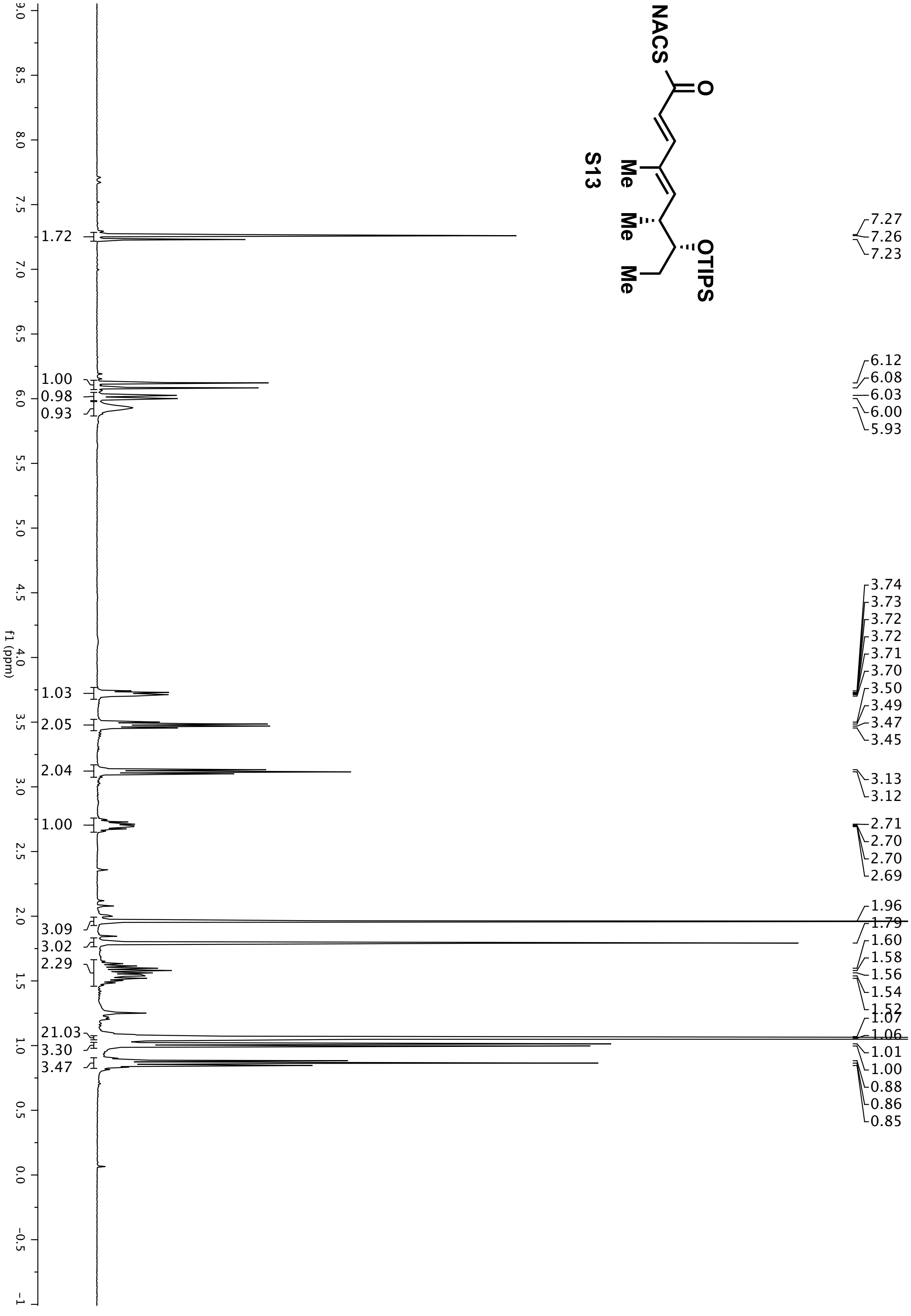
NACS
O
Me
Me
Me
OTIPS
S13
f1 (ppm)
7.27
7.26
7.23
6.12
6.08
6.03
6.00
5.93
3.74
3.73
3.72
3.72
3.71
3.70
3.50
3.49
3.47
3.45
3.13
3.12
2.71
2.70
2.70
2.69
1.96
1.79
1.60
1.58
1.56
1.54
1.52
1.07
1.06
1.01
1.00
0.88
0.86
0.85
1.72
1.00
0.98
0.93
1.03
2.05
2.04
1.00
3.09
3.02
2.29
21.03
3.30
3.47

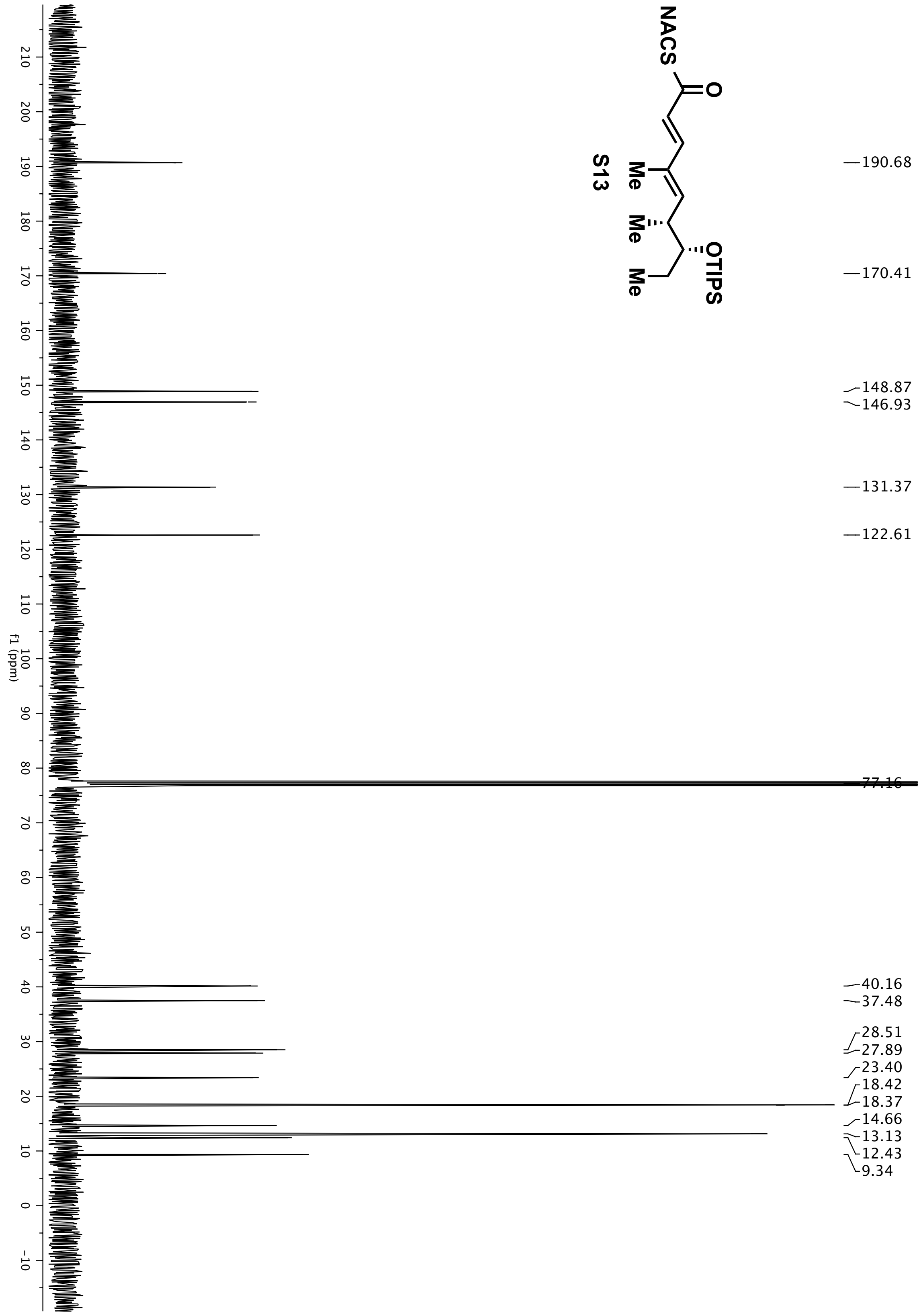

NACS
O
OTIPS
Me
Me
Me
S13
190.68
170.41
148.87
146.93
131.37
122.61
77.16
40.16
37.48
28.51
27.89
23.40
18.42
18.37
14.66
13.13
12.43
9.34
f1 (ppm)

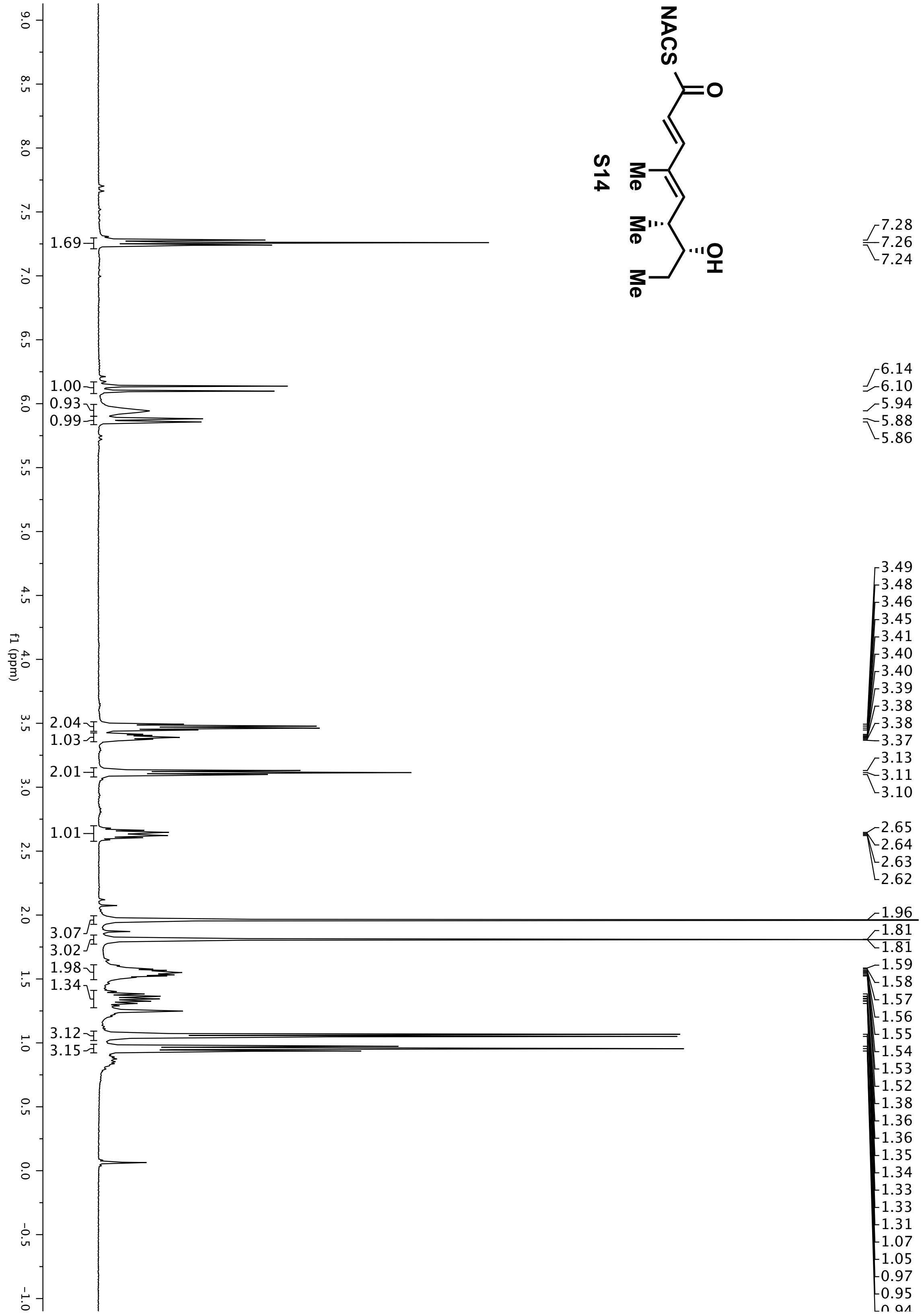

NACS
O
Me
Me
OH
Me
S14
f1 (ppm)
1.69
1.00
0.93
0.99
2.04
1.03
2.01
1.01
3.07
3.02
1.98
1.34
3.12
3.15
7.28
7.26
7.24
6.14
6.10
5.94
5.88
5.86
3.49
3.48
3.46
3.45
3.41
3.40
3.40
3.39
3.38
3.38
3.37
3.13
3.11
3.10
2.65
2.64
2.63
2.62
1.96
1.81
1.81
1.59
1.58
1.57
1.56
1.55
1.54
1.53
1.52
1.38
1.36
1.36
1.35
1.34
1.33
1.33
1.31
1.07
1.05
0.97
0.95

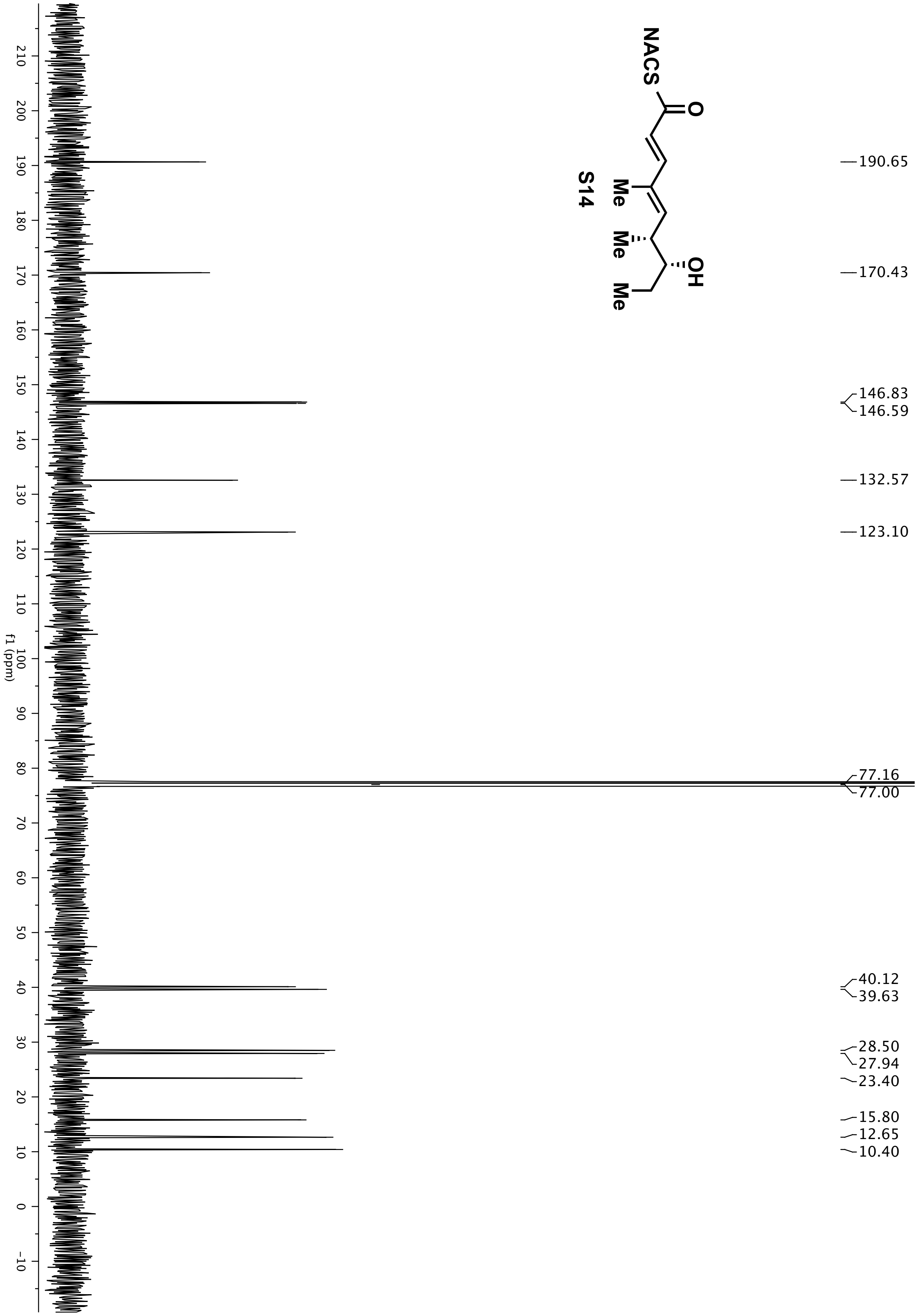
NACS
O
OH
Me
Me
Me
S14
190.65
170.43
146.83
146.59
132.57
123.10
77.16
77.00
40.12
39.63
28.50
27.94
23.40
15.80
12.65
10.40
f1 (ppm)

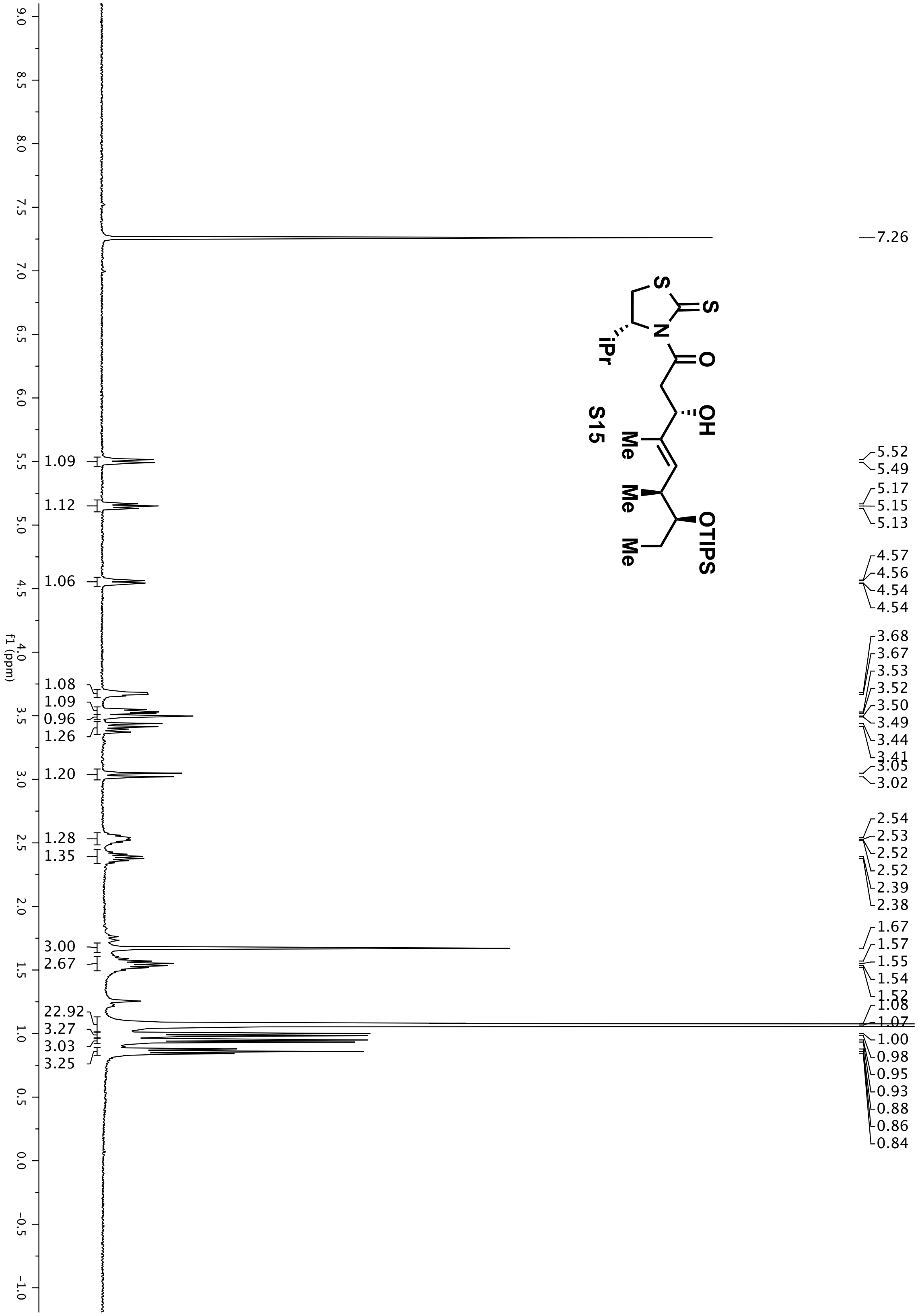
S15
OH
O
S
N
S
iPr
Me
Me
Me
OTIPS
f1 (ppm)
9.0
8.5
8.0
7.5
7.0
6.5
6.0
5.5
5.0
4.5
4.0
3.5
3.0
2.5
2.0
1.5
1.0
0.5
0.0
-0.5
-1.0
1.09
1.12
1.06
1.08
1.09
0.96
1.26
1.20
1.28
1.35
3.00
2.67
22.92
3.27
3.03
3.25
7.26
5.52
5.49
5.17
5.15
5.13
4.57
4.56
4.54
4.54
3.68
3.67
3.53
3.52
3.50
3.49
3.44
3.41
3.05
3.02
2.54
2.53
2.52
2.52
2.39
2.38
1.67
1.57
1.55
1.54
1.52
1.08
1.07
1.00
0.98
0.95
0.93
0.88
0.86
0.84

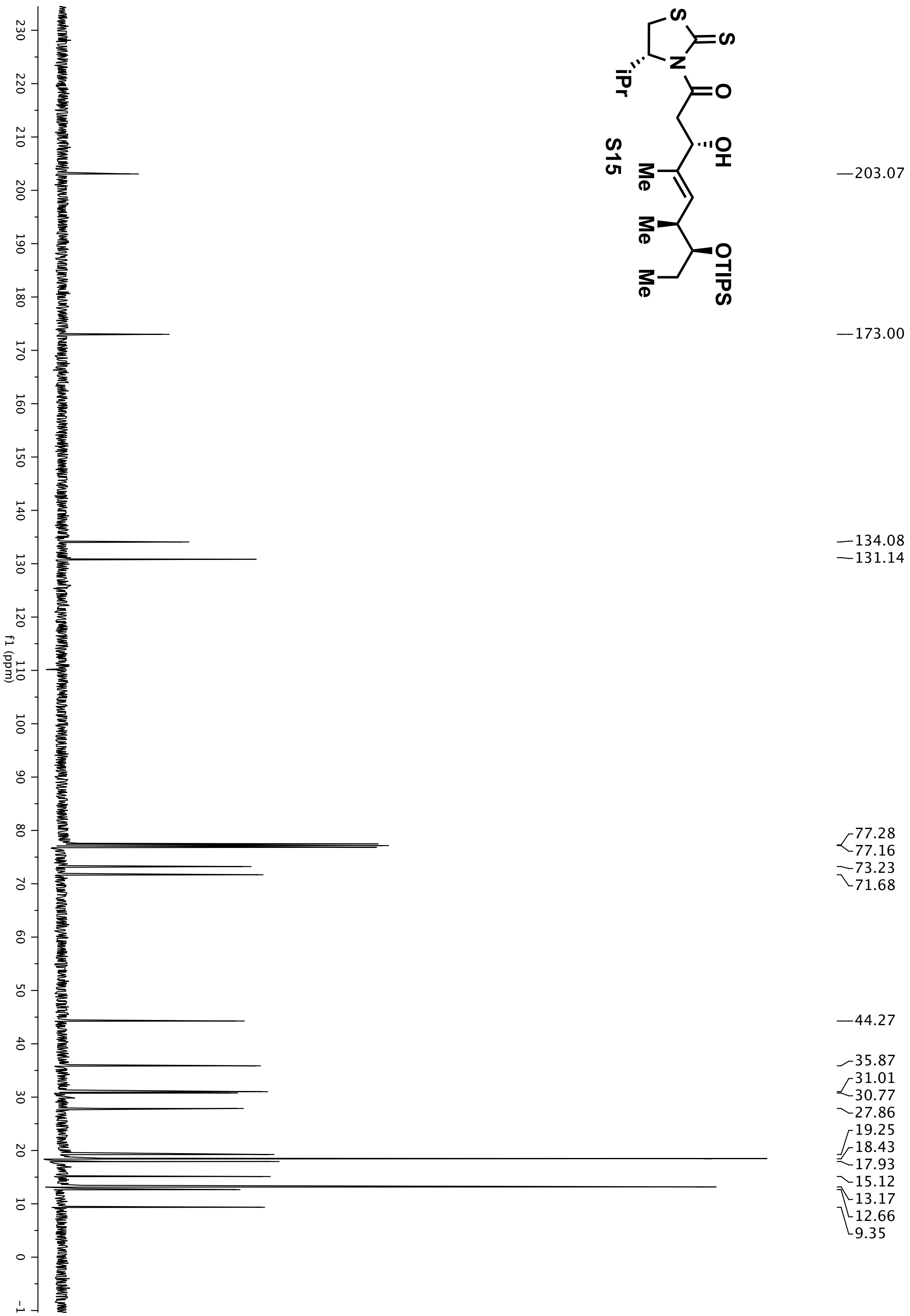
S15
OH
OTIPS
Me
Me
Me
iPr
S
S
N
O
203.07
173.00
134.08
131.14
77.28
77.16
73.23
71.68
44.27
35.87
31.01
30.77
27.86
19.25
18.43
17.93
15.12
13.17
12.66
9.35
f1 (ppm)

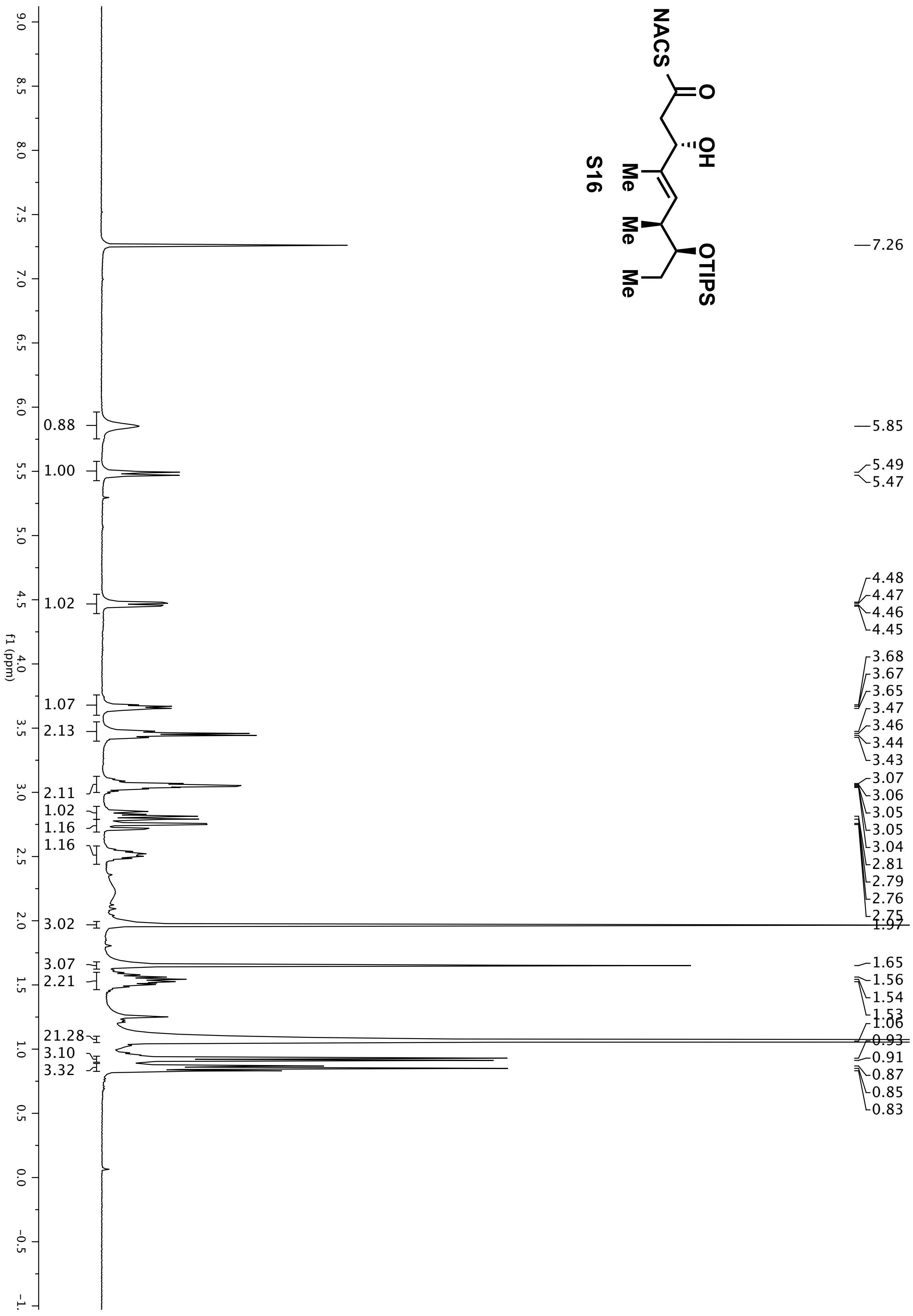
NACS
O
OH
OTIPS
Me
Me
Me
S16
f1 (ppm)
7.26
5.85
5.49
5.47
4.48
4.47
4.46
4.45
3.68
3.67
3.65
3.47
3.46
3.44
3.43
3.07
3.06
3.05
3.05
3.04
2.81
2.79
2.76
2.75
1.97
1.65
1.56
1.54
1.53
1.06
0.93
0.91
0.87
0.85
0.83
0.88
1.00
1.02
1.07
2.13
2.11
1.02
1.16
1.16
3.02
3.07
2.21
21.28
3.10
3.32

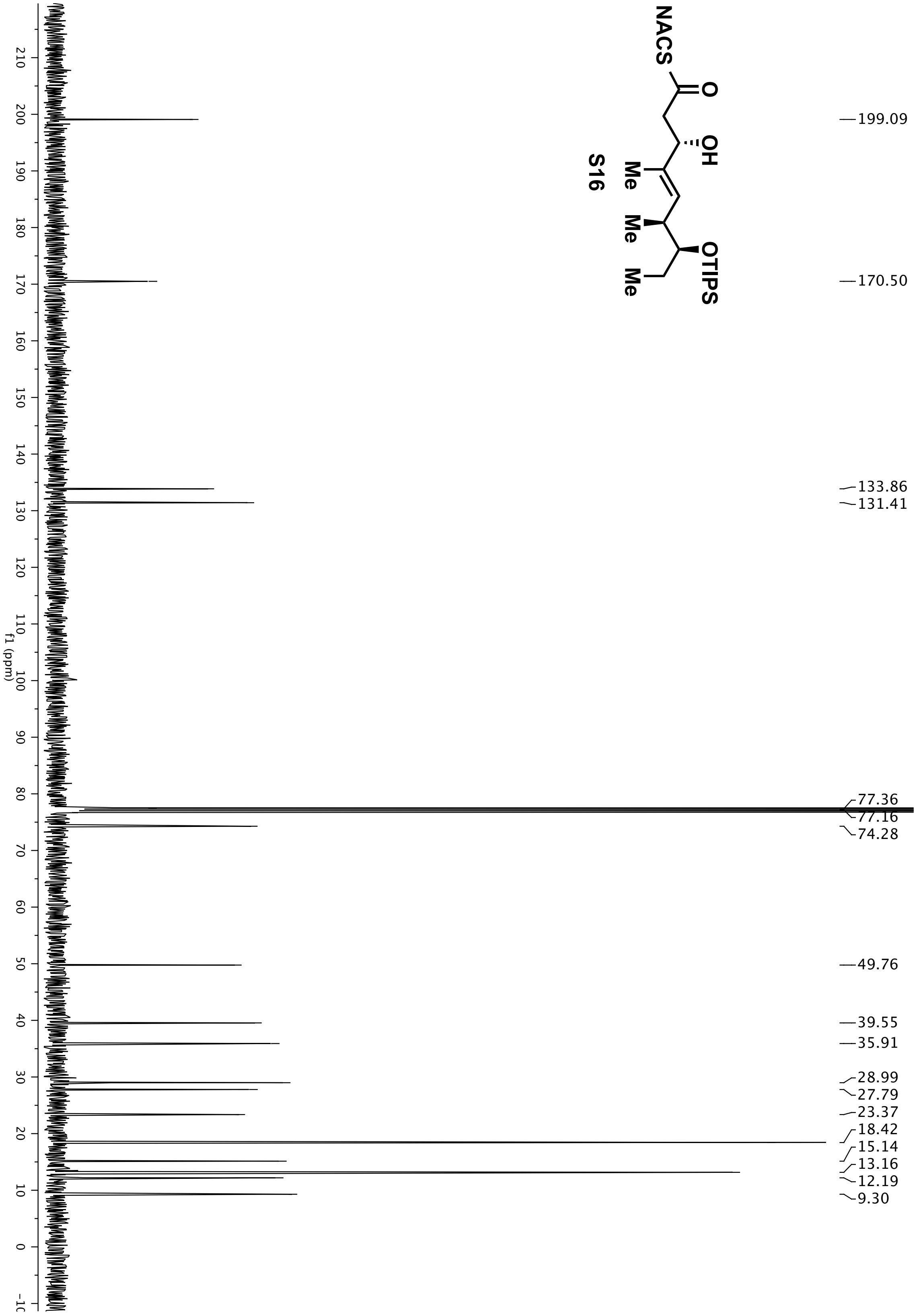
NACS
O
OH
Me
Me
OTIPS
Me
S16
199.09
170.50
133.86
131.41
77.36
77.16
74.28
49.76
39.55
35.91
28.99
27.79
23.37
18.42
15.14
13.16
12.19
9.30
f1 (ppm)

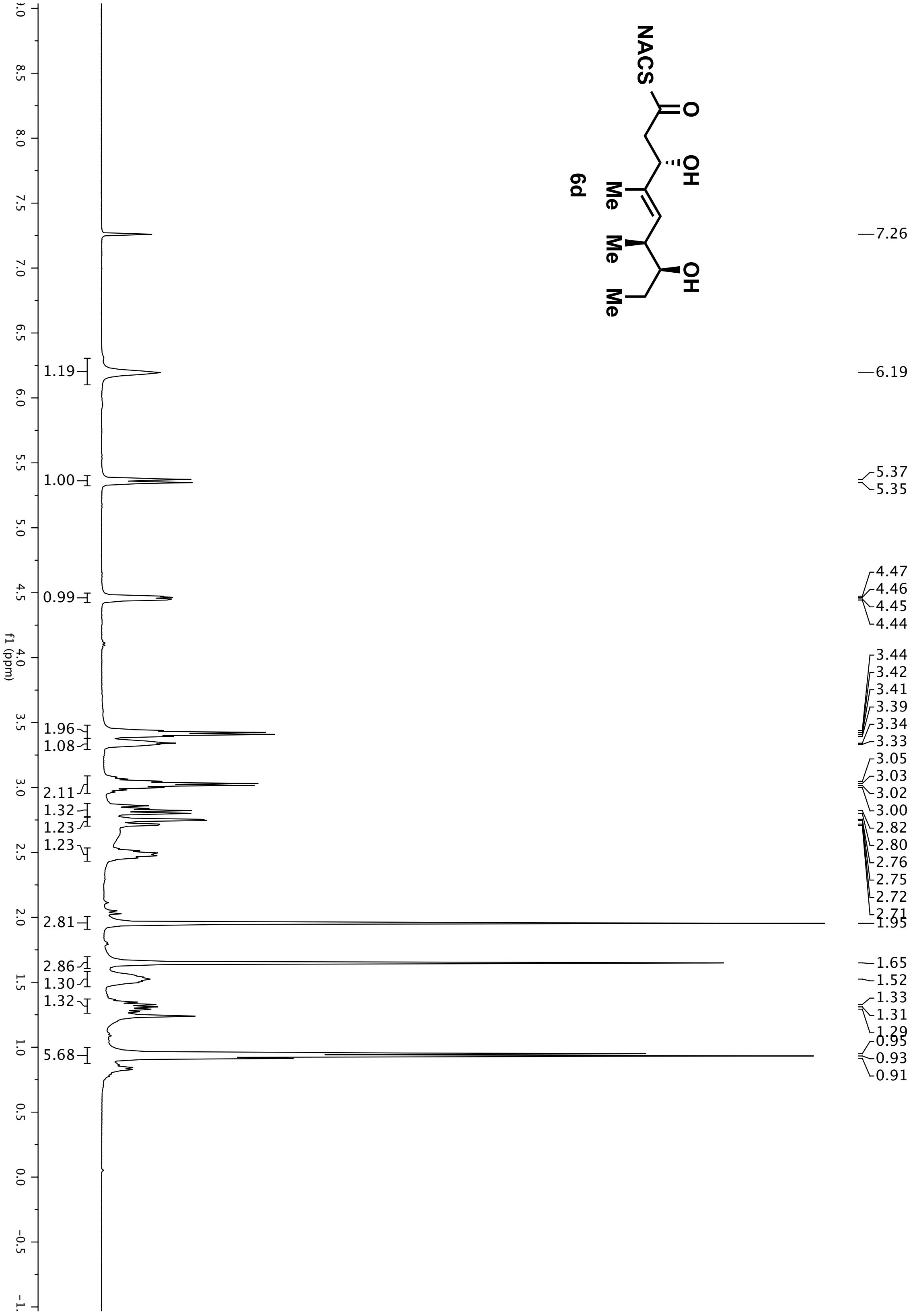
NACS
O
OH
Me
Me
OH
Me
6d
f1 (ppm)
7.26
6.19
5.37
5.35
4.47
4.46
4.45
4.44
3.44
3.42
3.41
3.39
3.34
3.33
3.05
3.03
3.02
3.00
2.82
2.80
2.76
2.75
2.72
2.71
1.95
1.65
1.52
1.33
1.31
1.29
0.95
0.93
0.91
1.19
1.00
0.99
1.96
1.08
2.11
1.32
1.23
1.23
2.81
2.86
1.30
1.32
5.68

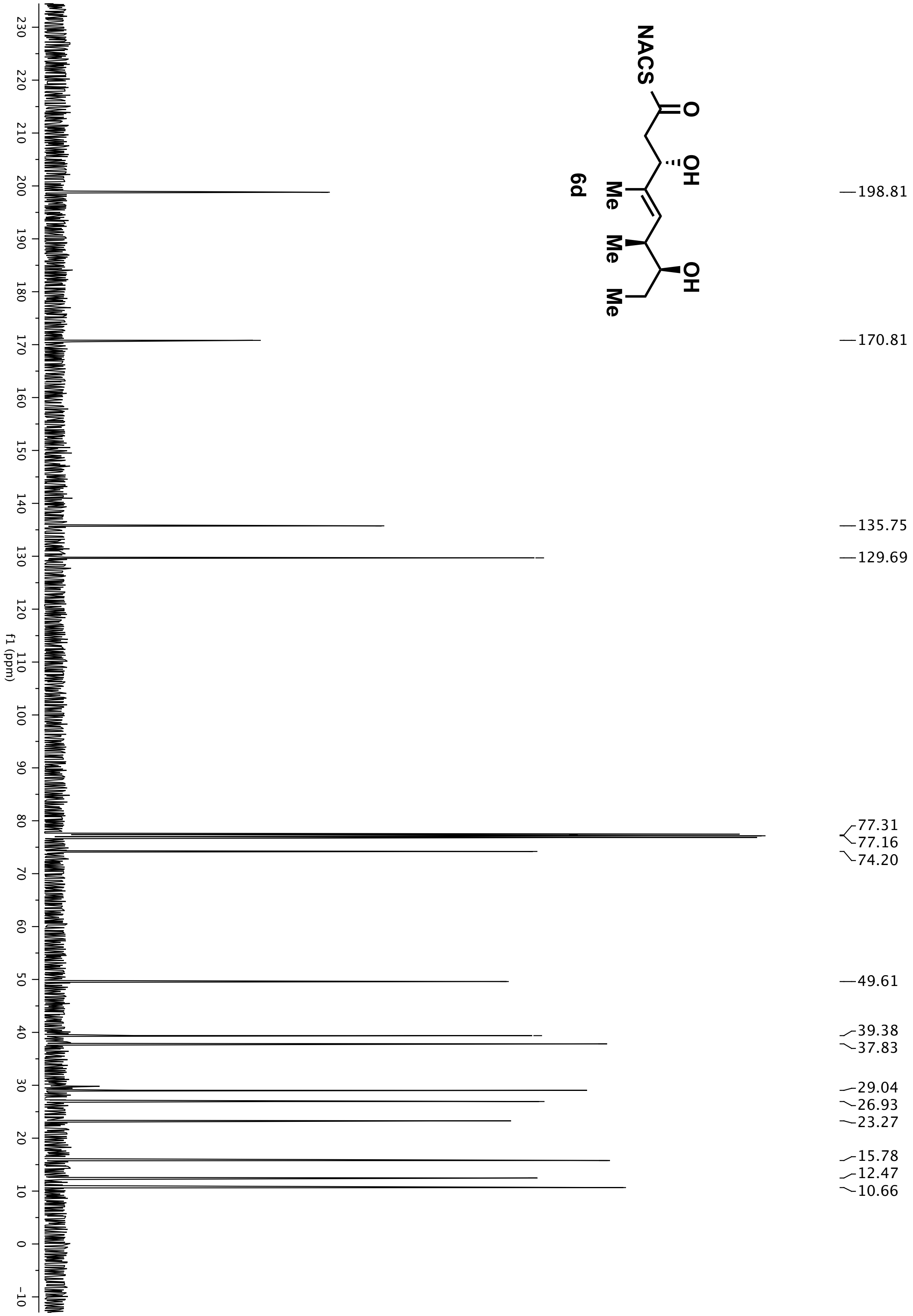
NACS
O
OH
OH
Me
Me
Me
6d
198.81
170.81
135.75
129.69
77.31
77.16
74.20
49.61
39.38
37.83
29.04
26.93
23.27
15.78
12.47
10.66
f1 (ppm)

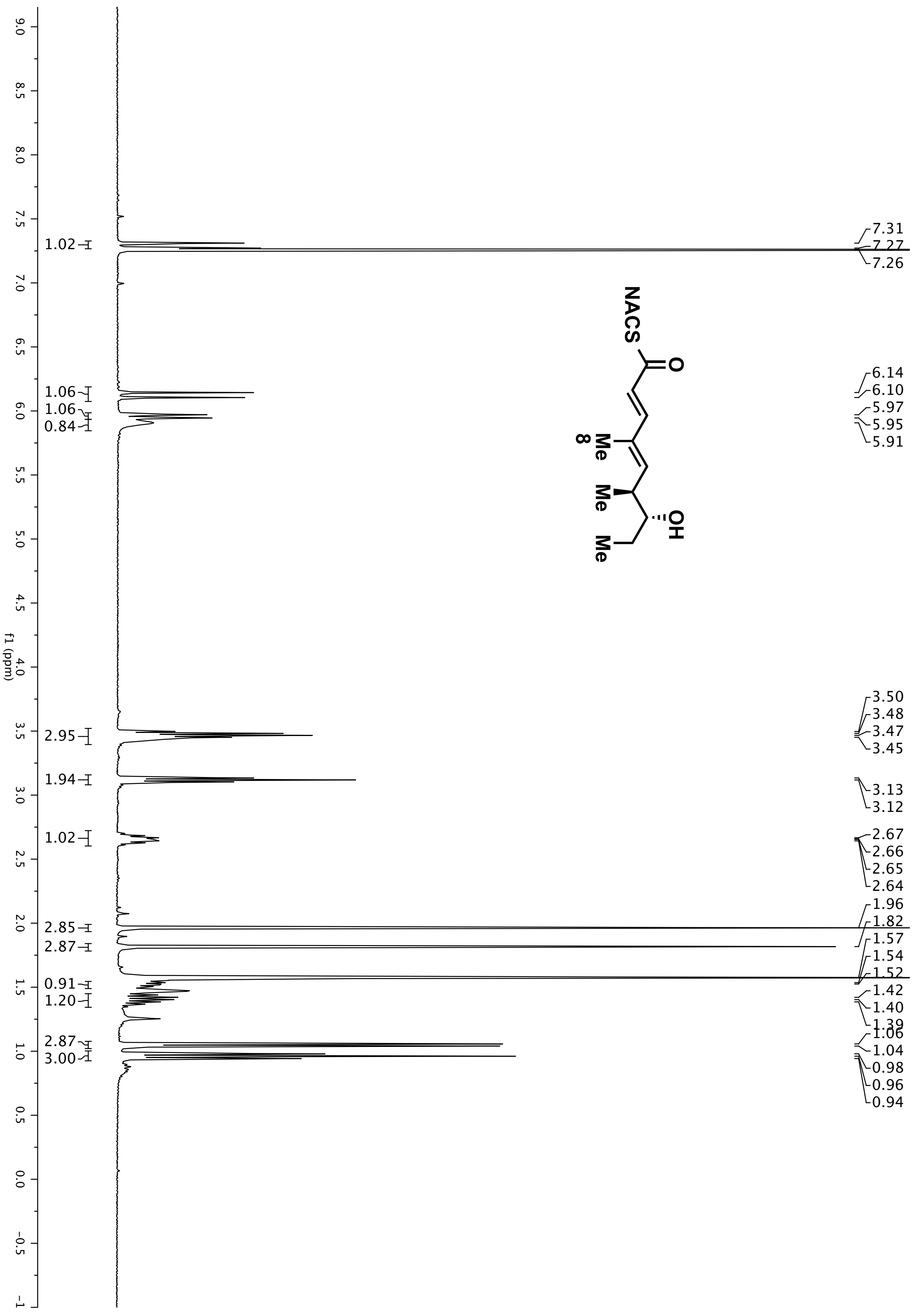
NACS
O
OH
Me
Me
Me
8
f1 (ppm)
9.0
8.5
8.0
7.5
7.0
6.5
6.0
5.5
5.0
4.5
4.0
3.5
3.0
2.5
2.0
1.5
1.0
0.5
0.0
−0.5
−1
1.02
1.06
1.06
0.84
2.95
1.94
1.02
2.85
2.87
0.91
1.20
2.87
3.00
7.31
7.27
7.26
6.14
6.10
5.97
5.95
5.91
3.50
3.48
3.47
3.45
3.13
3.12
2.67
2.66
2.65
2.64
1.96
1.82
1.57
1.54
1.52
1.42
1.40
1.39
1.06
1.04
0.98
0.96
0.94

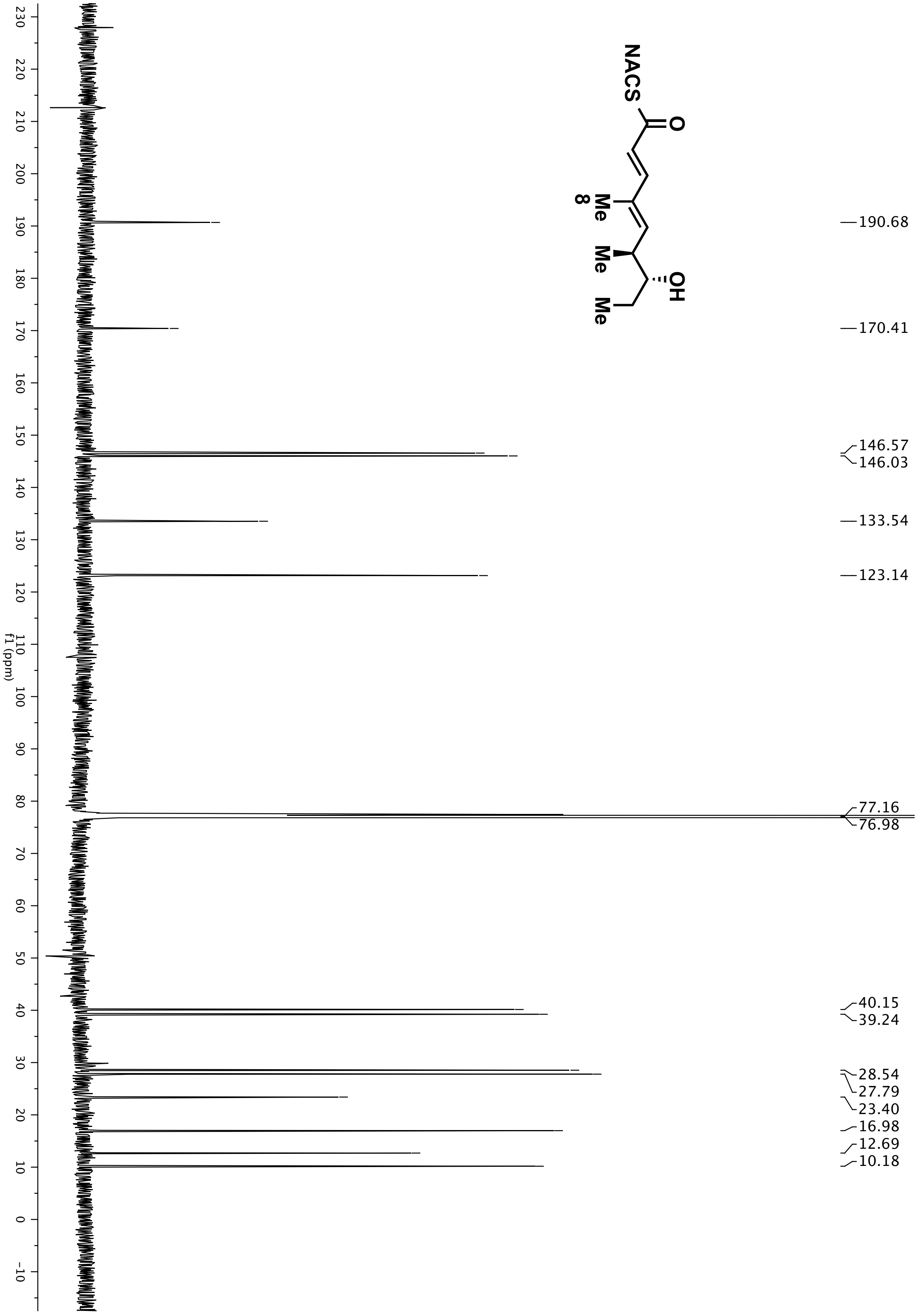
NACS
O
Me
8
Me
OH
Me
190.68
170.41
146.57
146.03
133.54
123.14
77.16
76.98
40.15
39.24
28.54
27.79
23.40
16.98
12.69
10.18
f1 (ppm)

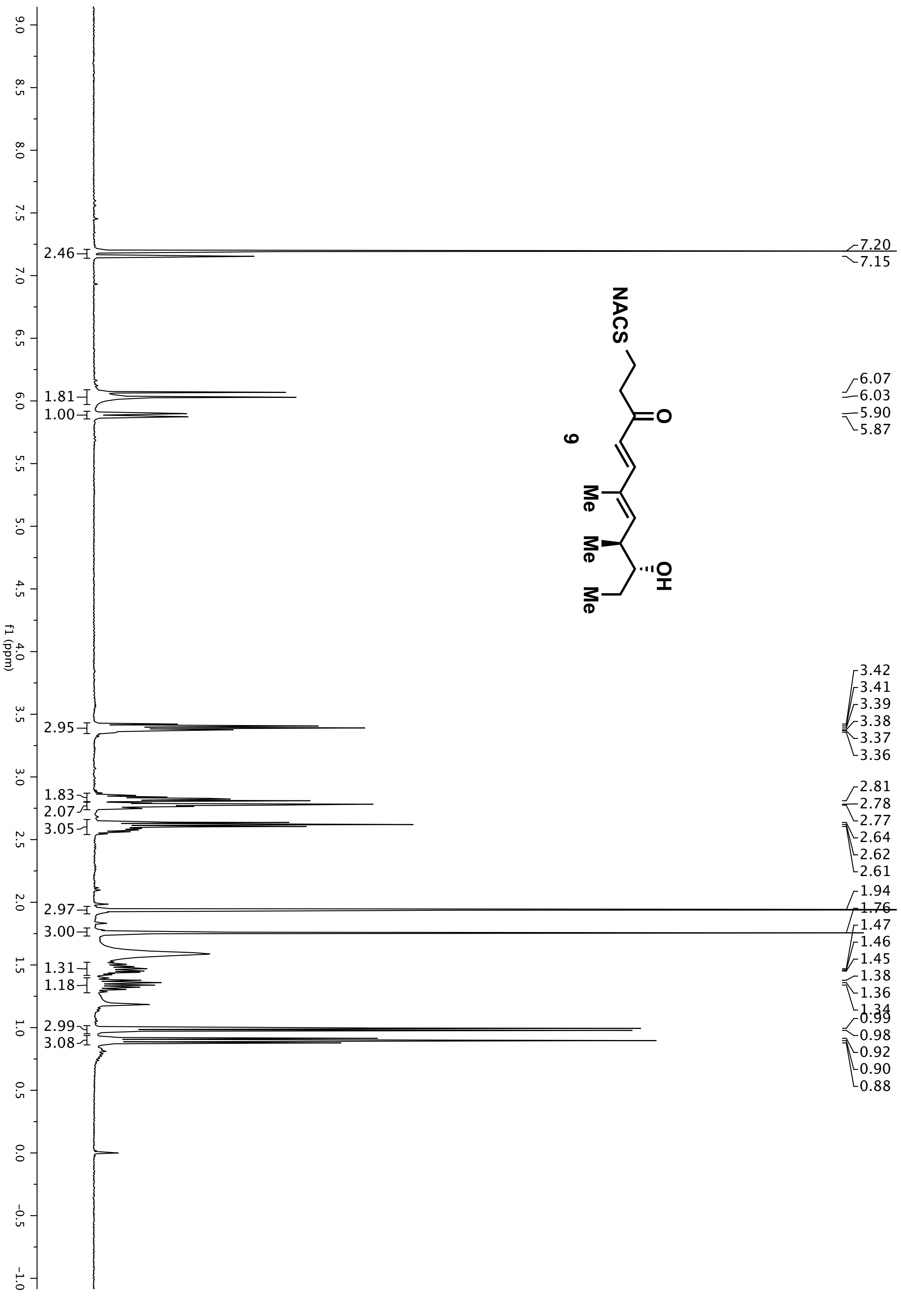

NACS
O
9
Me
Me
Me
OH
7.20
7.15
6.07
6.03
5.90
5.87
3.42
3.41
3.39
3.38
3.37
3.36
2.81
2.78
2.77
2.64
2.62
2.61
1.94
1.76
1.47
1.46
1.45
1.38
1.36
1.34
0.99
0.98
0.92
0.90
0.88
2.46
1.81
1.00
2.95
1.83
2.07
3.05
2.97
3.00
1.31
1.18
2.99
3.08
f1 (ppm)

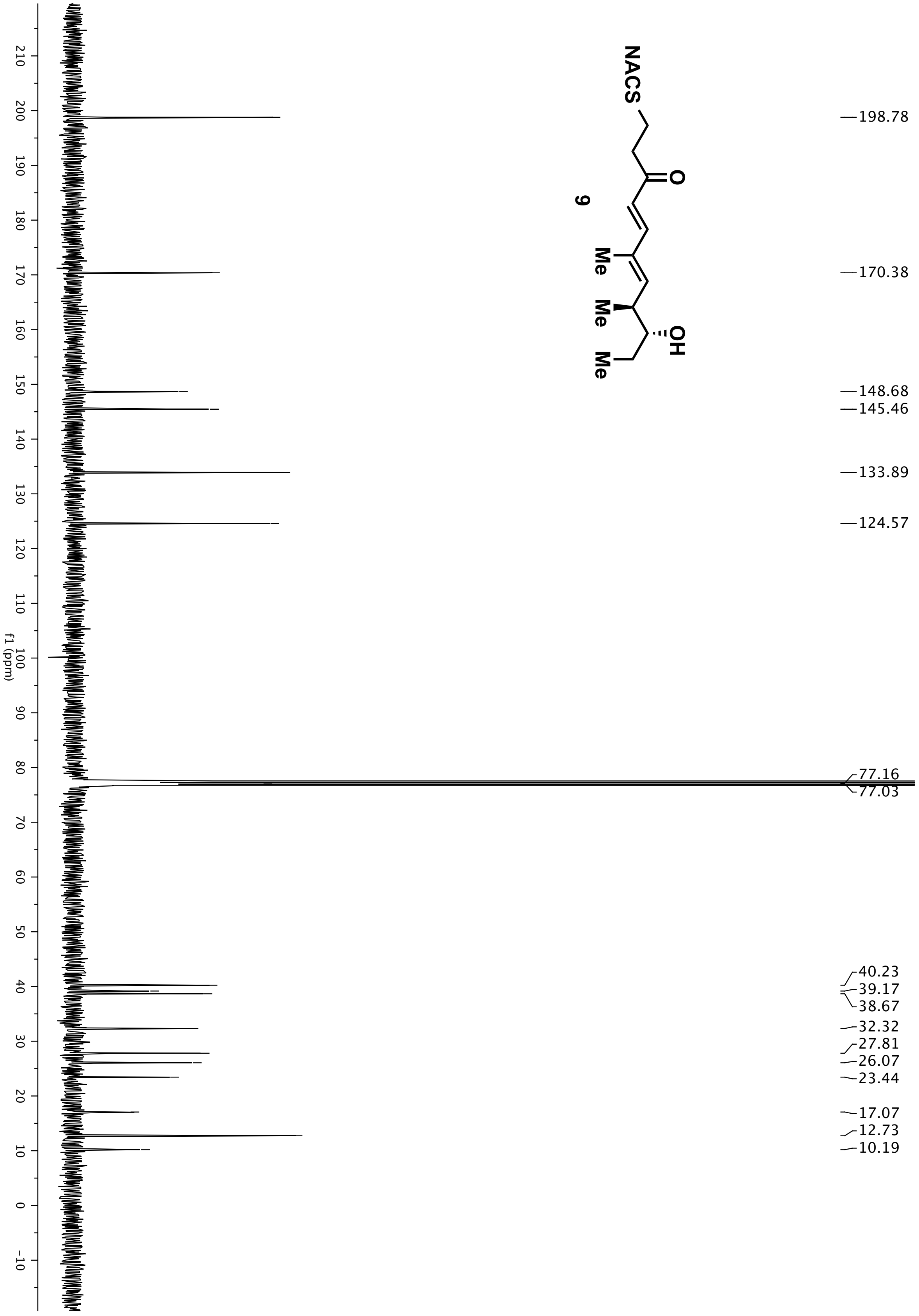
NACS
O
OH
Me
Me
Me
9
198.78
170.38
148.68
145.46
133.89
124.57
77.16
77.03
40.23
39.17
38.67
32.32
27.81
26.07
23.44
17.07
12.73
10.19
f1 (ppm)
